# Design and Discovery of MRTX0902, a Potent, Selective, Brain-Penetrant, and Orally Bioavailable Inhibitor of the SOS1:KRAS Protein–Protein Interaction

**DOI:** 10.1021/acs.jmedchem.2c00741

**Published:** 2022-07-14

**Authors:** John M. Ketcham, Jacob Haling, Shilpi Khare, Vickie Bowcut, David M. Briere, Aaron C. Burns, Robin J. Gunn, Anthony Ivetac, Jon Kuehler, Svitlana Kulyk, Jade Laguer, J. David Lawson, Krystal Moya, Natalie Nguyen, Lisa Rahbaek, Barbara Saechao, Christopher R. Smith, Niranjan Sudhakar, Nicole C. Thomas, Laura Vegar, Darin Vanderpool, Xiaolun Wang, Larry Yan, Peter Olson, James G. Christensen, Matthew A. Marx

**Affiliations:** Mirati Therapeutics, 3545 Cray Court, San Diego, California 92121, United States

## Abstract

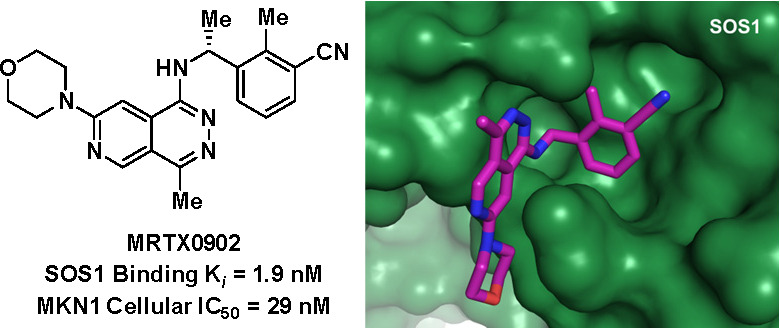

SOS1 is one of the major guanine nucleotide exchange factors that regulates the ability of KRAS to cycle through its “on” and “off” states. Disrupting the SOS1:KRAS^G12C^ protein–protein interaction (PPI) can increase the proportion of GDP-loaded KRAS^G12C^, providing a strong mechanistic rationale for combining inhibitors of the SOS1:KRAS complex with inhibitors like MRTX849 that target GDP-loaded KRAS^G12C^. In this report, we detail the design and discovery of MRTX0902—a potent, selective, brain-penetrant, and orally bioavailable SOS1 binder that disrupts the SOS1:KRAS^G12C^ PPI. Oral administration of MRTX0902 in combination with MRTX849 results in a significant increase in antitumor activity relative to that of either single agent, including tumor regressions in a subset of animals in the MIA PaCa-2 tumor mouse xenograft model.

## Introduction

Activating mutations of *KRAS* that lead to aberrant signaling and hyperactivation within the MAPK pathway are among the most common driver mutations in human cancers.^1,2^ These *KRAS* mutations, a majority of which are single codon mutations (G12, G13, Q61, etc.), show a high occurrence in some of the most aggressive cancer types: non-small-cell lung cancer (NSCLC), colorectal cancer (CRC), and pancreatic cancers.^1,2^ The prevalence of *KRAS* mutations has made KRAS a prime target for oncology drug discovery programs for several decades. KRAS, however, was thought to be undruggable until the recent clinical success of the KRAS^G12C^ inhibitors adagrasib (MRTX849)^3−5^ and sotorasib (AMG510).^6,7^

KRAS is a small GTPase that cycles between the GTP-loaded “on” state and the GDP-loaded “off” state—a process that is crucial for normal cell proliferation and survival. One of the major regulators of this process is the Son of Sevenless (SOS) protein, a guanine nucleotide exchange factor (GEF) that acts as a key activator for KRAS function.^8−10^ The binding between KRAS and SOS proteins helps facilitate the turnover of GDP-loaded KRAS into its GTP-loaded state. While two homologs of SOS exist (SOS1 and SOS2) that impart GEF activity onto KRAS, various studies have demonstrated a dominant role for SOS1 over SOS2.^11,12^ Moreover, SOS1 is the only isoform that is reported to participate in a negative feedback loop within the KRAS pathway.^13,14^ Additionally, functional genomic screens have identified that several cancer cell lines addicted to KRAS signaling are particularly sensitive to genetic perturbation of SOS1.^15^ Functionally, KRAS mutations lead to a reduction in GTPase activity, resulting in a higher population of GTP-bound KRAS and increased RAS-pathway signaling.^16−18^ GTP-bound KRAS also binds to an allosteric site on SOS1, leading to an increase in SOS1 GEF activity, thereby ensuring a high population of active KRAS.^19^

Activating mutations of SOS1 are found in ∼1% of lung adenocarcinomas and uterine carcinomas and at lower frequencies in other cancer types.^20^ Furthermore, these mutations have been reported in other RASopathies such as hereditary gingival fibromatosis (HGF)^21,22^ and Noonan’s syndrome.^23−25^ The catalytic site of SOS1 has a well-defined binding pocket adjacent to the KRAS:SOS1 interface; thus, disrupting the SOS1:KRAS protein–protein interaction (PPI) with an SOS1 binder is a compelling strategy to help treat KRAS-driven cancers.

The clinical stage KRAS^G12C^ inhibitor, adagrasib, has garnered much attention recently based on its promising clinical activity across several cancer types.^3−5^ Since adagrasib is an irreversible inhibitor that targets GDP-loaded KRAS^G12C^, we envisioned a combination strategy with an SOS1 binder that could disrupt the KRAS:SOS1 PPI and increase the concentration of adagrasib-susceptible GDP-loaded KRAS^G12C^, potentially leading to an increased response rate and/or more durable clinical responses relative to single agent adagrasib. Additionally, this combination strategy could be leveraged to target other *KRAS* mutant-driven cancers with the appropriate KRAS^mut^ inhibitor combination partner (KRAS^G12D^, KRAS^G12V^, etc.).

**Figure 1:**
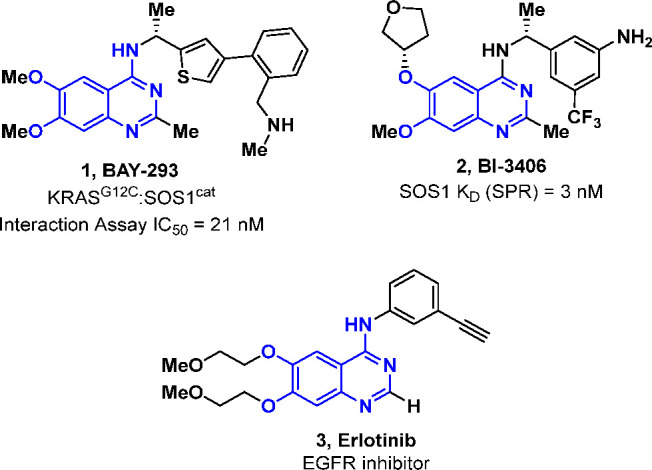
Comparison of representative SOS1 literature compounds and erlotinib.

While several reports have detailed the discovery of small-molecule agonists of SOS1,^26−33^ fewer literature reports have described the use of compounds that disrupt the SOS1:KRAS complex^34−37^ or degrade SOS1.^38^ However, several patent applications have been published describing the use of SOS1 binders to target *KRAS* mutant cancers.^39−54^ Early SOS1 binders such as BAY-293 (**1**)^35^ and BI-3406 (**2**)^36,37^ were discovered from independent high-throughput screening campaigns and serendipitously share the same quinazoline scaffold (Figure 1), similar to many EGFR inhibitors, such as erlotinib (**3**, Figure 1). The 2-methyl substituent was installed to preclude binding to the hinge region of EGFR and achieve selectivity for SOS1.^35,55^ In these leads, the 6-ether substituents extend beyond the SOS1 binding pocket to block the KRAS:SOS1 PPI and prevent the reactivation of KRAS.

**Figure 2:**
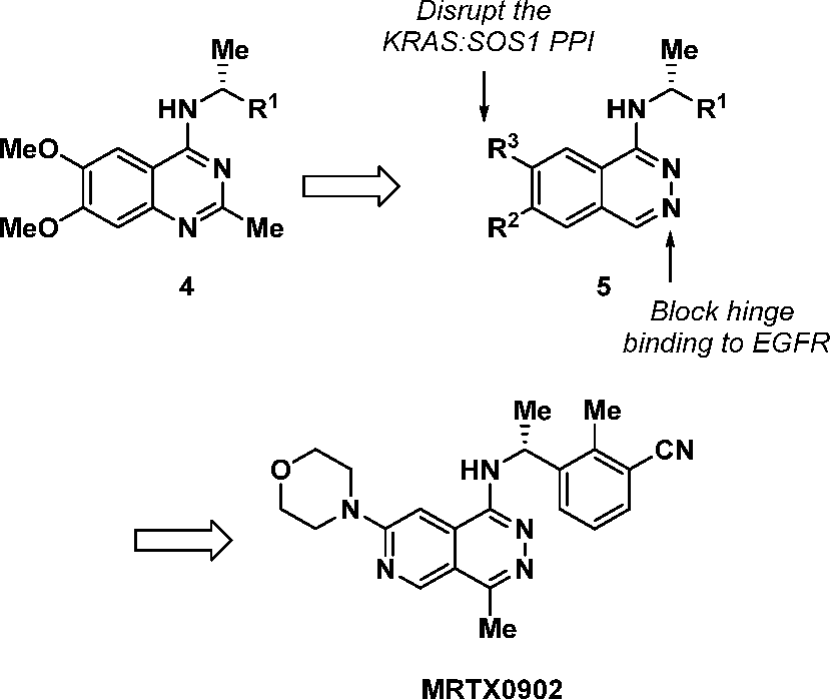
Design concept for phthalazine-based inhibitors of the SOS1:KRAS PPI leading to MRTX0902.

Herein, we report the design of a new class of phthalazine-based SOS1 binders that effectively disrupt the SOS1:KRAS protein–protein interaction (Figure 2). These molecules are highly selective for SOS1 and show no activity against EGFR. Our work has led to the identification of the clinical candidate MRTX0902, a potent and orally bioavailable inhibitor of the SOS1:KRAS complex that exhibits complete tumor regressions in mouse models when administered in combination with sub-maximal doses of our KRAS^G12C^ inhibitor adagrasib.

## Results and Discussion

Initial modeling efforts based on previously reported co-crystal structures^35^ of SOS1 suggested that the transposition of the N1-quinazoline nitrogen (**4**) to form a phthalazine core (**5**) would be well-tolerated within the binding pocket of SOS1 while also preventing binding to EGFR (Figure 2). We began with a simplified class of 6,7-dimethoxy-substituted phthalazines and screened several benzylic amino substituents to probe the hydrophobic back pocket of SOS1 (**6**–**11**, Table 1). The potency of these inhibitors was measured using an HTRF displacement assay (*K*_i_) and an In-Cell Western Assay that quantifies phosphorylated ERK1/2 (pERK) modulation in MKN1 cells (IC_50_). Gratifyingly, substitution with a simple (*R*)-α-methylbenzyl amine resulted in a compound (**6**) with an SOS1 *K*_i_ of 637 nM and no activity against EGFR (IC_50_ > 10 000 nM). To push deeper into the pocket, the phenyl substituent in **6** was replaced with a naphthyl ring (**7**), leading to an 8-fold increase in binding potency (*K*_i_ = 76 nM). However, we found that simply substituting the phenyl ring in **6** with a 3-CF_3_ substituent (**8**) led to a 12-fold boost in binding affinity (*K*_i_ = 52 nM) and measurable cellular potency in the MKN1 cell line (IC_50_ = 958 nM), while the 2-Me substituent (**9**) resulted in a 3-fold loss in binding potency (*K*_i_ = 2049 nM) when compared with compound **6**. Surprisingly, combination of the 2-Me and 3-CF_3_ substituents led to a compound (**10**) with an SOS1 *K*_i_ of 13 nM and MKN1 cellular IC_50_ of 378 nM. Presumably this boost in potency was due to the combined lipophilicity of both substituents pushing into the hydrophobic back pocket. Furthermore, installation of the synthetically more complex 2,4-substituted thiophene (**11**), inspired by BAY-293 (**1**),^35^ led to a 2–3-fold boost in both SOS1 *K*_i_ and cellular potency (*K*_i_ = 3.9 nM and MKN1 IC_50_ = 165 nM) when compared to those of compound **10**. The initial structure–activity relationship (SAR) of these phthalazines proved that the phthalazine core provided a new class of highly potent SOS1 binders not previously reported in the literature. With this new scaffold in hand, we then focused on designing a bioavailable compound for in vivo profiling.

**Table 1:**
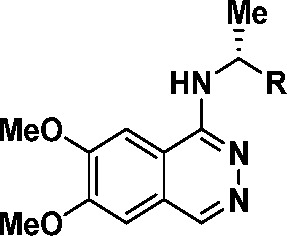

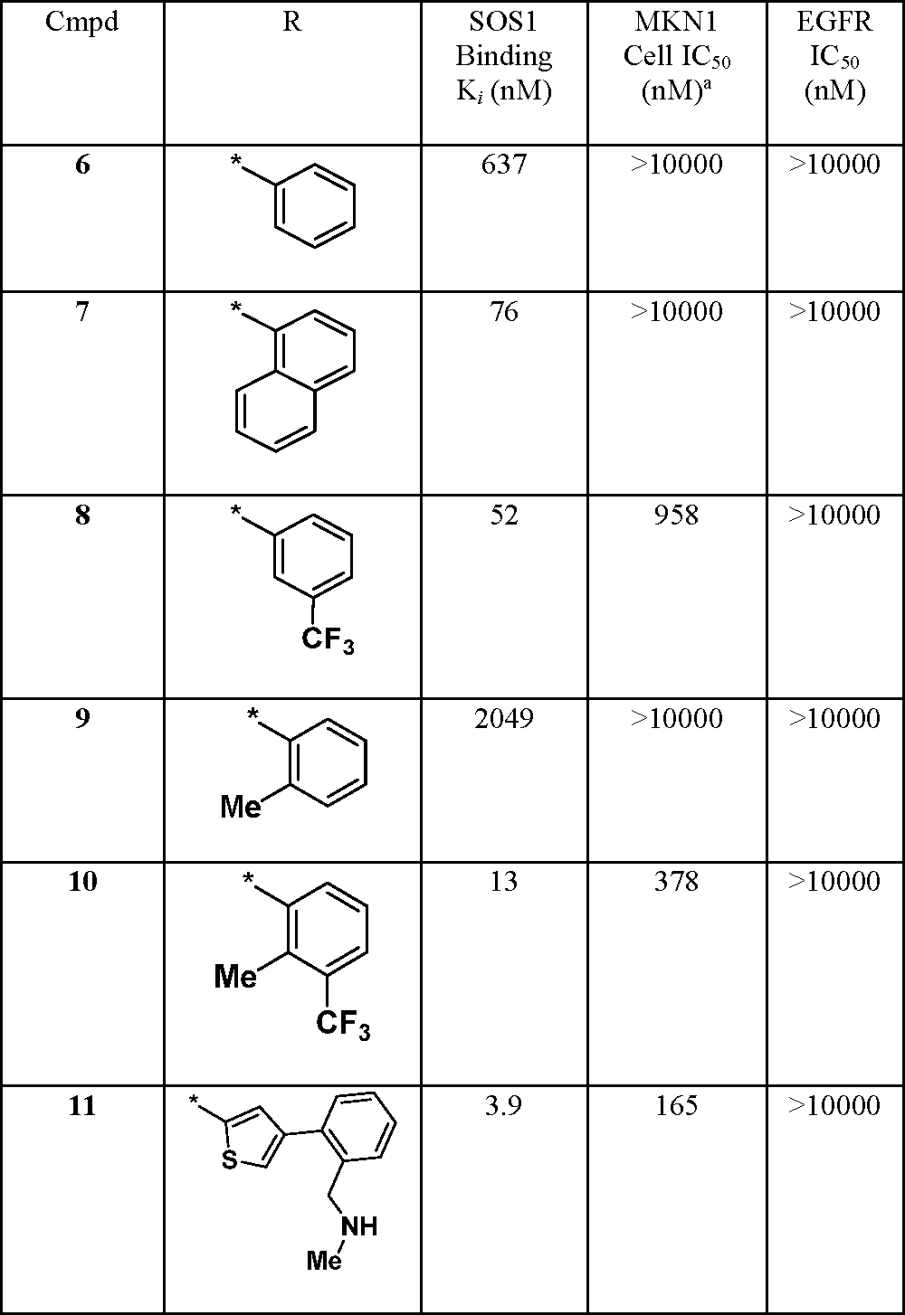
Initial Phthalazine Binders of SOS1

a In-Cell Western Assay measuring pERK.

Early in vitro ADME profiling revealed that the C4-position of the phthalazine scaffold is highly susceptible to metabolism by aldehyde oxidase (AO) (Table 2).^56,57^ When tested in human liver S9 fractions, **11** was rapidly metabolized, leading to a *t*_1/2_ of only 14 min. However, in the presence of the known AO inhibitor raloxifene, the *t*_1/2_ of **11** was dramatically increased to >180 min.

**Table 2:**
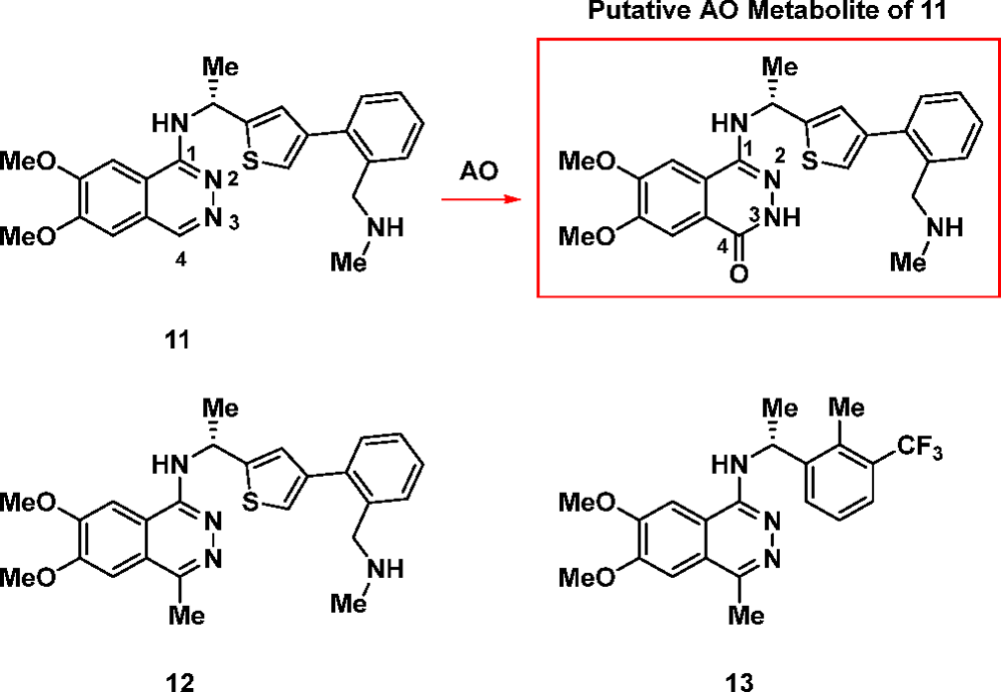
C4-Methyl Substitution Blocks Aldehyde Oxidase-Mediated Metabolism in Human Liver S9 Fractions

			*t*_1/2_ (min)	
Cmpd	SOS1 binding *K*_i_ (nM)	MKN1 cell IC_50_ (nM)	human liver S9	human liver S9 + 25 μM raloxifene
**11**	3.9	165	14	>180
**12**	0.5	249	>180	>180
**13**	2.6	195	>180	>180

**Figure 3:**
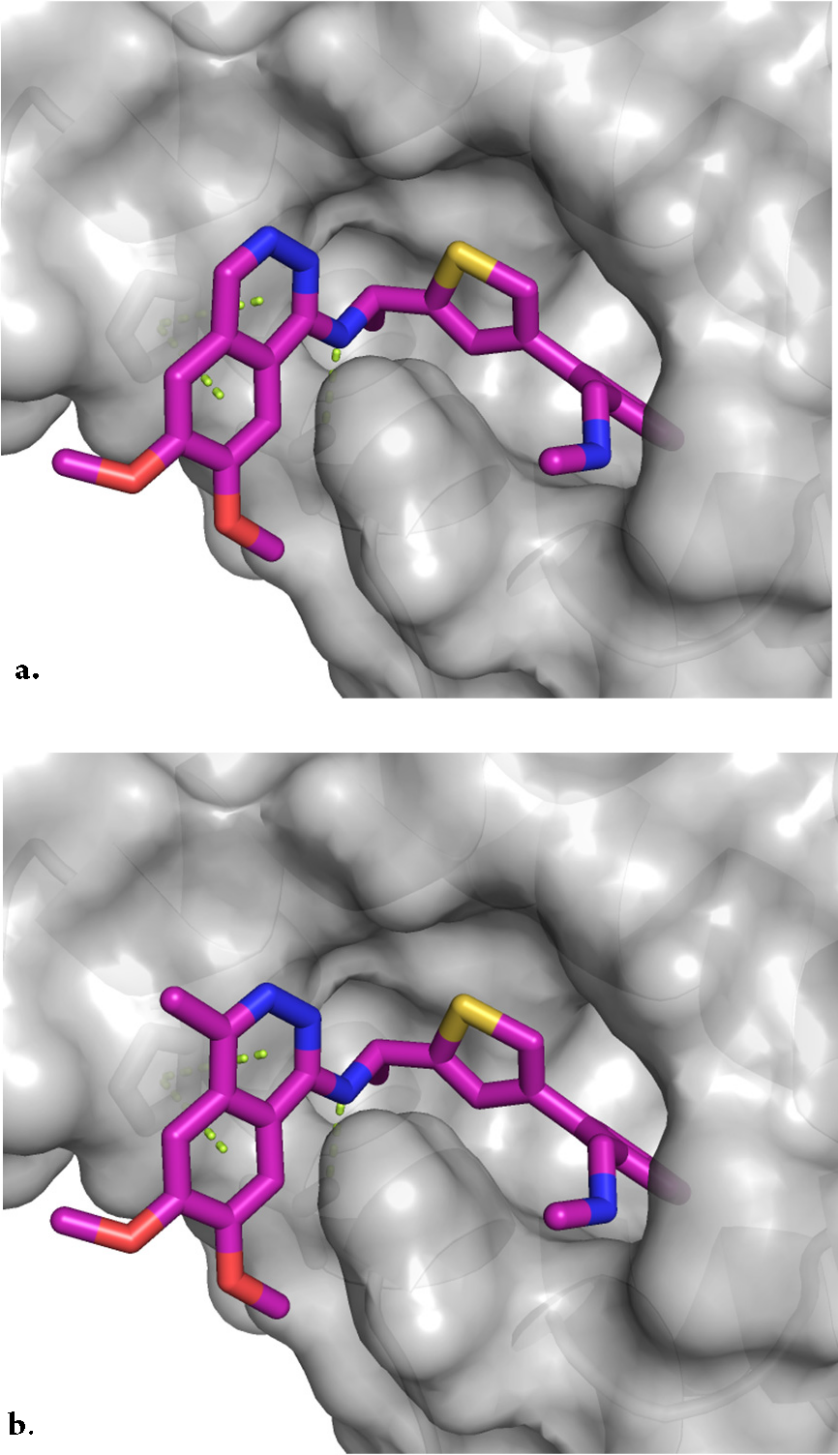
(a) Modeled structure of **11** bound to SOS1 (b) Modeled structure of **12** bound to SOS1 (modeled with PDB 5OVI).

Molecular modeling (using MOE software^58^) of **11** in SOS1 (Figure 3a, modeled with PDB 5OVI) indicated that the C4-methyl analog **12** (Figure 3b) should bind to SOS1 effectively; moreover, this C4-methyl may also block AO-mediated oxidation. Gratifyingly, installation of the C4-methyl in **12** increased the SOS1 *K*_i_ by 8-fold (SOS1 *K*_i_ = 0.5 nM) while effectively blocking AO metabolism (*t*_1/2_ > 180 min ± raloxifene, **12**, Table 2). This increase in metabolic stability was further demonstrated with the simplified (*R*)-1-(2-methyl-3-(trifluoromethyl)phenyl)ethan-1-amine analog **13** without a loss in binding potency when compared to **11**, and with cellular potency comparable to that of **11** and **12**.

Having resolved the oxidative metabolism liability of the original core, we next focused our efforts on exploring the SAR around the C7-substituent. The ideal C7-substituent should extend from the SOS1 binding pocket into the interface of the KRAS:SOS1 protein–protein interaction between the Arg73 of KRAS and Asn879/Tyr884 of SOS1, thereby disrupting the PPI, while also providing favorable drug-like properties. For synthetic ease and reduction of molecular weight, the simplified C1-substituent (*R*)-1-(2-methyl-3-(trifluoromethyl)phenyl)ethan-1-amine was held constant while probing the C7-vector (Table 3). Further molecular modeling suggested that the C6-substituent would have little effect on the binding of these phthalazines. Thus, the C6-methoxy substituent was removed, and a simple dimethylamine group was installed in the C7-position to provide an inhibitor (**14**) with high binding affinity and cellular potency (*K*_i_ = 2.2 nM, MKN1 IC_50_ = 57 nM). Furthermore, replacing the dimethylamine with a piperazine (**15**) led to a ∼7-fold increase in binding affinity for SOS1 and similar cellular potency when compared with **14**. However, swapping out the C7-nitrogen linkage with a carbon to form piperidine **16** was met with a 10-fold loss in binding potency. This observation indicated that the electronics of the phthalazine core and the conformation of the C7-substituent could play significant roles in the SOS1 activity (*vide infra*). Decreasing the basicity of **15** with piperazinone **17** resulted in a similar cellular potency, suggesting that a wide range of neutral and basic substituents could be tolerated at this position.

**Table 3:**
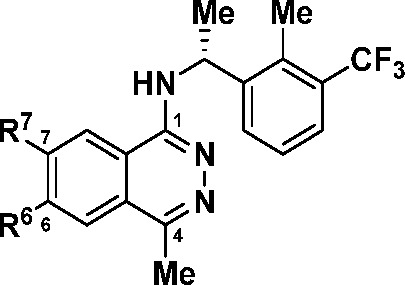

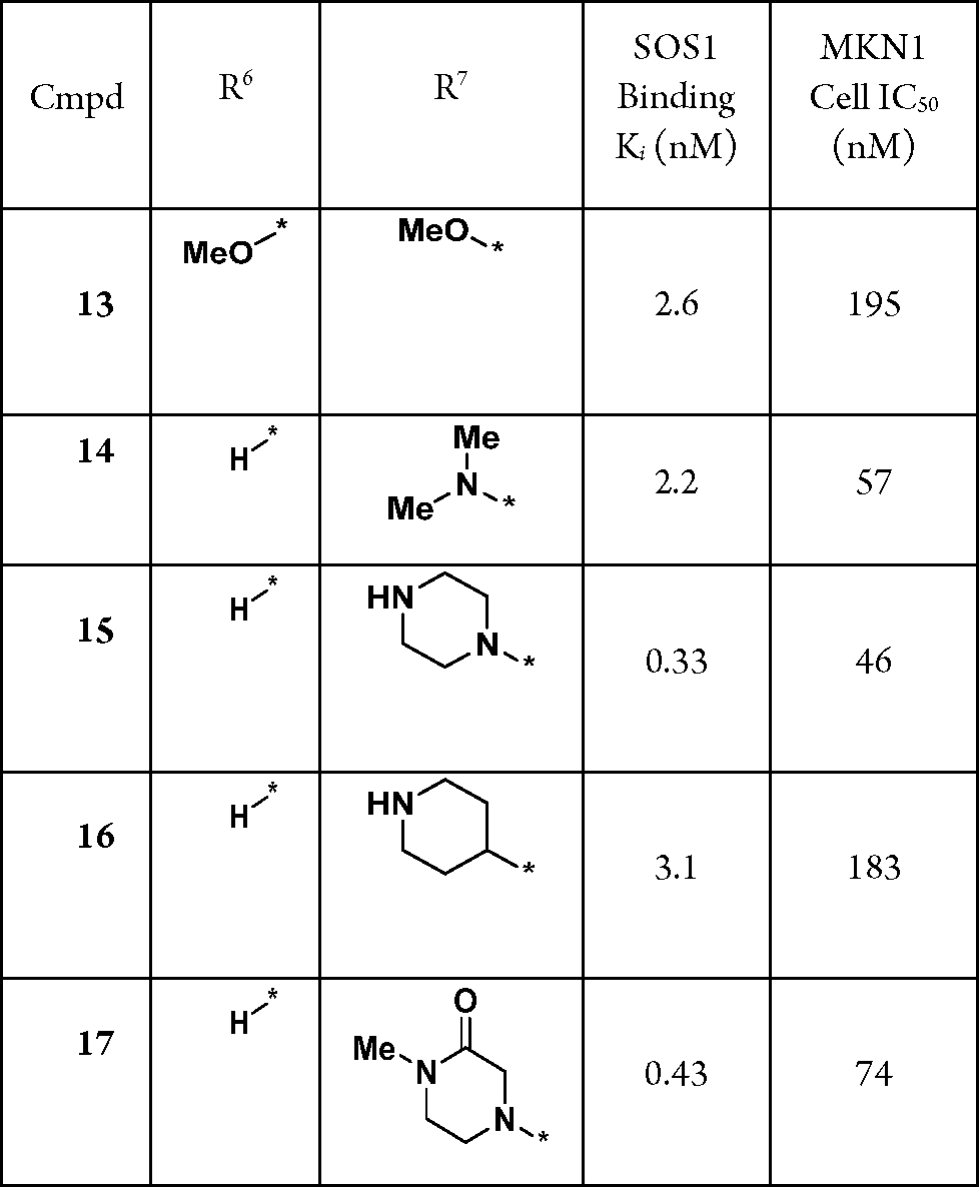
Initial SAR Data of the C7-Substituent

**Figure 4:**
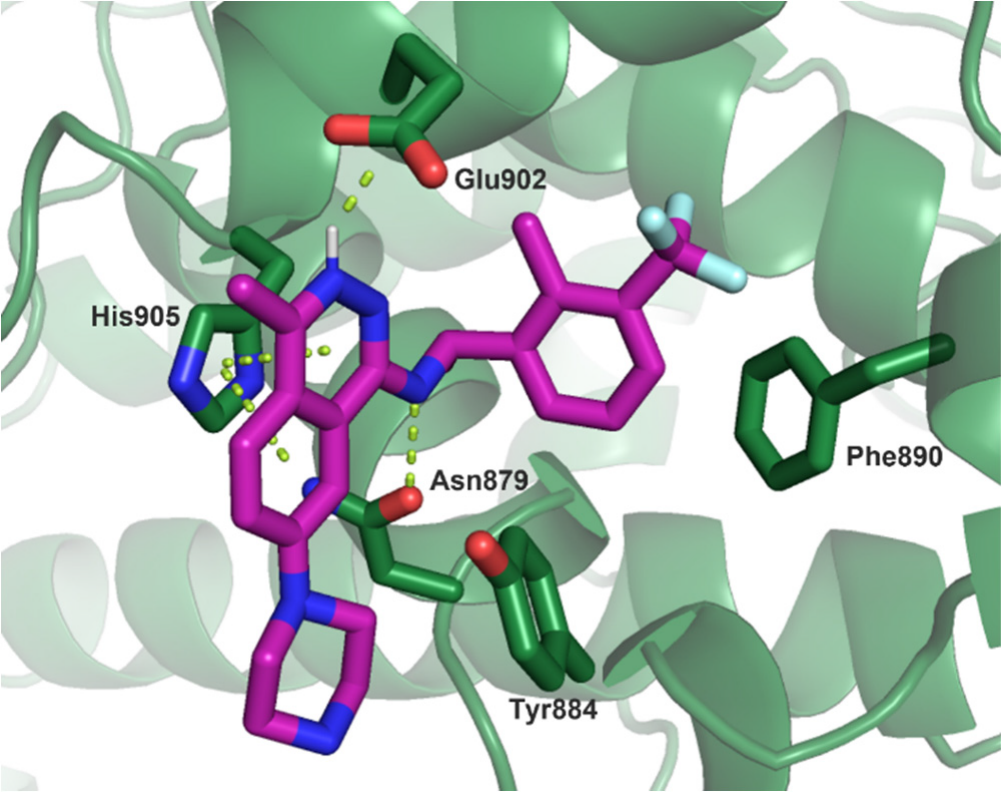
X-ray co-crystal structure of **15** bound to SOS1 (PDB 7UKS).

With the potent piperazine **15** in hand, we next obtained an X-ray co-crystal structure of it bound to SOS1 (Figure 4, PDB 7UKS). As expected from our modeling efforts, the phthalazine core makes a π-stacking interaction with His905, and the N–H from the C1-benzyl amine substituent creates a crucial hydrogen bond with Asn879. The chiral α-methyl on the benzylic amine fills a small cavity in the pocket and positions the phenyl group for an edge-to-face interaction with Phe890. The C7-piperazine protrudes out of the binding pocket and into the region where the SOS1:KRAS PPI occurs, driving the disruption of the SOS1:KRAS complex. Interestingly, based on atomic distances in the crystal structure, the nitrogen on the 3-position of the phthalazine core is clearly protonated and makes a salt bridge with the carboxylate of Glu902. This is in stark contrast to the previously reported quinazoline-based SOS1 binders that require a methyl group in this position and are unable to utilize this interaction.^34−37^ Finally, the C4-methyl substituent protrudes into the solvent front, which indicated that this vector could be used as an additional opportunity for growth and further tuning of the physicochemical properties of our phthalazine scaffold.

To probe the SAR of the C4-position and tune the electronics of the core structure, we first tested both electron-withdrawing and electron-donating groups such as trifluoromethyl and methoxy substituents, **18** and **19**, respectively (Table 4). Unfortunately, while both substituents modeled well in the crystal structure, these changes were met with a >100-fold loss in binding affinity and a complete loss of cellular potency for compounds **18** and **19**. Furthermore, replacing the C4-methyl with a dimethylamine (**20**) resulted in a >3-fold loss in both the binding and cellular potency, and oxidation of the phthalazine scaffold to phthalazinones **21** and **22** also proved detrimental. Sterically, these changes should be accommodated, thus providing further evidence that the electronics of the phthalazine core are crucial for SOS1 binding.

**Table 4:**
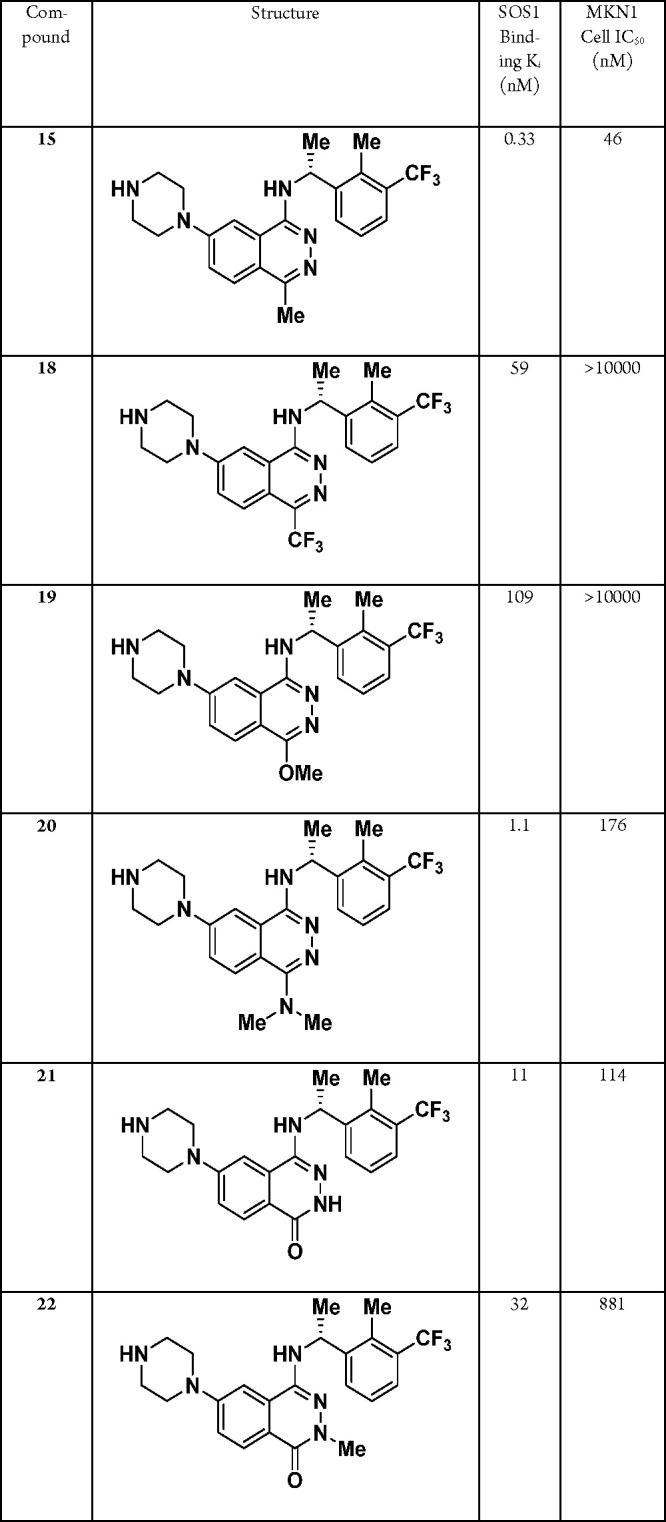
Evaluation of the C4-Substituent SAR

Based on the high affinity for SOS1 and ability to modulate pERK in the MKN1 cellular assay, **15** was dosed in female CD-1 mice to determine if its pharmacokinetics (PK) profile supported use of this compound as an in vivo tool for pharmacodynamics (PD) studies in mice (Table 5). Unfortunately, although the intrinsic clearance in human liver microsomes was moderate, **15** had a high intravenous (IV) clearance (Cl = 85 mL/min/kg) and low bioavailability (%F = 11) in mice. To reduce the clearance of **15**, the 6-position carbon on the phthalazine core was replaced with a nitrogen to lower lipophilicity and form the pyridopyridazine core in **23**. Fortunately, the pyridopyridazine **23** was equipotent to **15** and showed moderate clearance (Cl = 52 mL/min/kg) after IV bolus administration, with a significantly higher oral (PO) exposure and bioavailability (%F = 44). Furthermore, replacing the basic C7-piperazine substituent with a neutral morpholine substituent (**24**) significantly decreased the clearance (Cl = 17 mL/min/kg) and provided exceptionally high PO exposure (AUC_0-last_ = 130 318 ng·h/mL) and bioavailability (%F ≈ 100) in mice. This result was somewhat surprising, as studies with human liver microsomes predicted this compound to have high clearance, demonstrating a clear species difference with these compounds and a potential disconnect for in vitro/in vivo results.

**Table 5:**
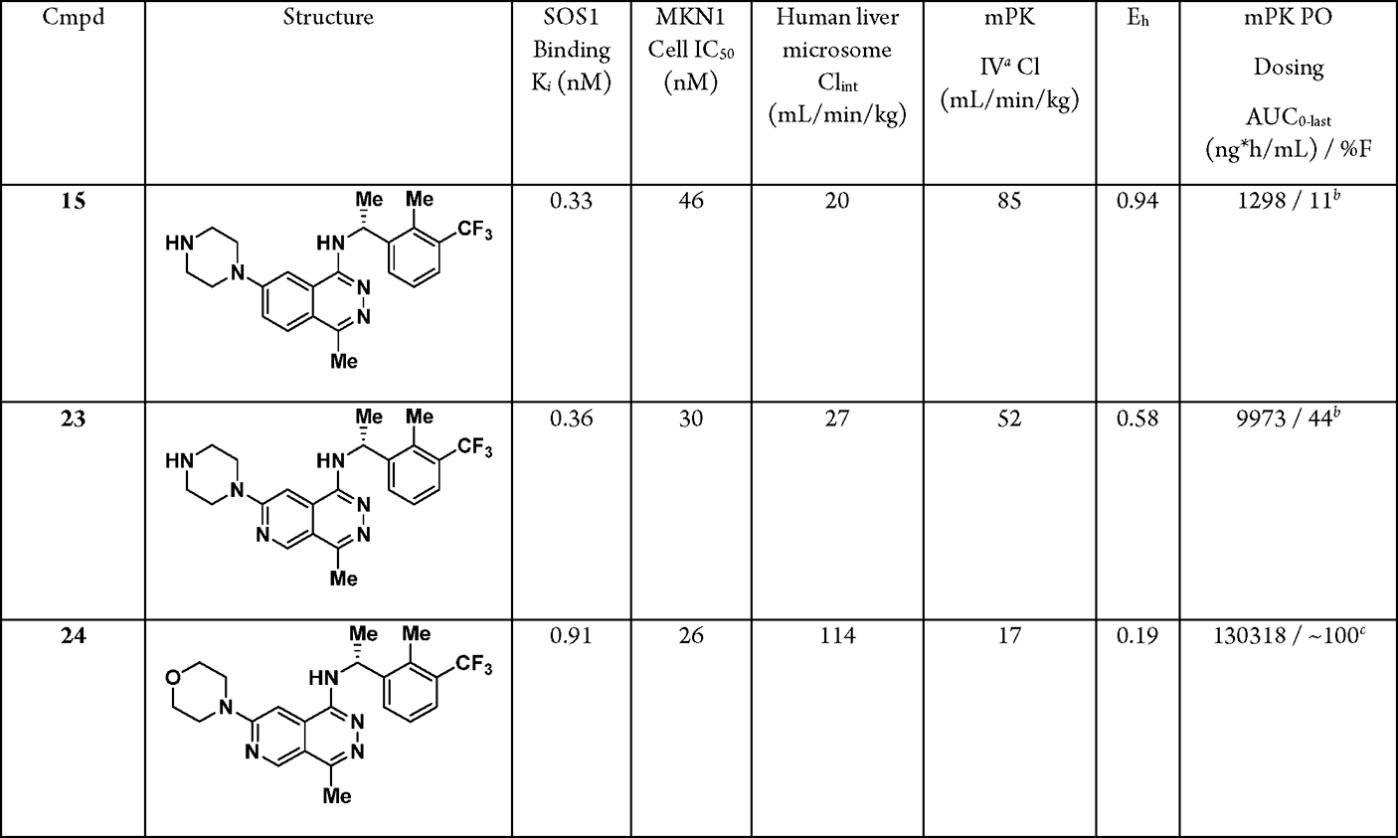
Mouse PK Profiles of Compounds **15**, **23**, and **24**

a IV dosing in CD-1 mice (3 mg/kg, 20% SBE-β-CD/50 mM citric acid pH 5.0).

b PO dosing in CD-1 mice (100 mg/kg, 20% SBE-β-CD/50 mM citric acid pH 5.0).

c PO dosing in CD-1 mice (100 mg/kg, 0.5% MC (4000 cps)/0.2% Tween80 in water).

**Figure 5:**
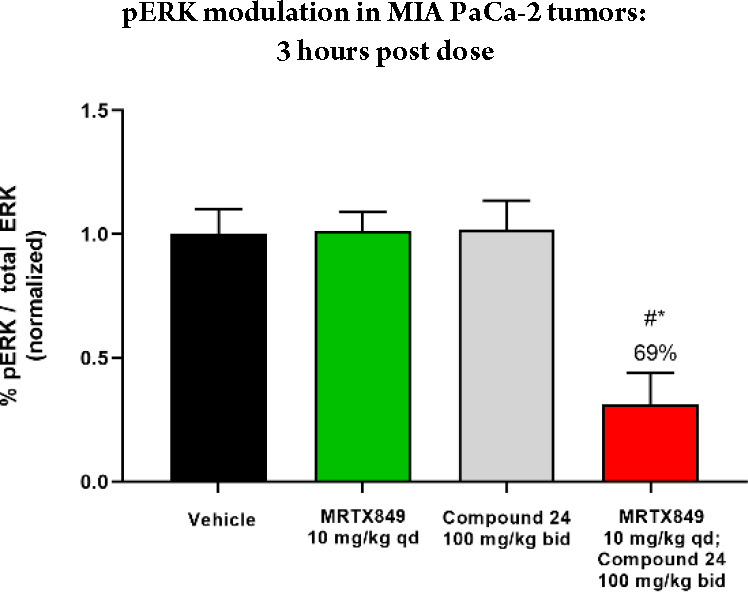
pERK modulation in tumors from mice dosed orally with compound **24**, MRTX849, and their combination. “#” indicates drug treated-tumor pERK levels were significantly different compared to vehicle-treated cohorts by two-tailed Student’s *t* test (GraphPad Prism v.8.2.0; *p*-value < 0.05). “*” indicates drug treated-tumor pERK levels were significantly different compared to MRTX849-treated cohorts by two-tailed Student’s *t* test (GraphPad Prism v.8.2.0; *p*-value < 0.05).

Based on the combined potency and mouse PK profile, compound **24** was selected as an exploratory tool for a PD study conducted in the *KRAS*^*G12C*^ mutant MIA PaCa-2 (human tumor cell line) mouse xenograft model (Figure 5). As a proof of concept for the combination of an SOS1 binder with a KRAS^G12C^ inhibitor, a dose of MRTX849 (10 mg/kg, dosed PO) that elicits approximate tumor stasis was administered to better observe the impact of **24** on the RAS/MAPK pathway. After 21 days of dosing, tumor pERK levels were determined 3 h after the last dose of orally administered **24** at 100 mg/kg bid (twice daily), MRTX849 at 10 mg/kg qd (once daily), and the combination of **24** and MRTX849. No modulation was observed with either single agent, whereas the combination showed a 69% reduction in pERK in the MIA PaCa-2 model. These results demonstrate that the combination of a KRAS^G12C^ inhibitor with an SOS1 binder more effectively inhibited the RAS/MAPK pathway and provided early proof of concept for the program.

While **24** served as an acceptable in vivo tool compound, it suffered from CYP3A4 inhibition (IC_50_ = 640 nM) and was not advanced further. To decrease the CYP inhibition of these phthalazine inhibitors, we further focused on designing inhibitors with decreased cLogP compared to the highly lipophilic **24** (cLogP = 4.4). Further modeling using the co-crystal structure of compound **15** suggested that a small hydrophilic hole existed in the back pocket of the protein and incorporation of more polar substituents on the phenyl ring may be tolerated. Simple replacement of the 2-methyl on the phenyl ring with a 2-fluoro substituent (**25**) was well tolerated for potency; however, this modification only slightly changed the cLogP and increased the human liver microsome Cl_int_ by 2-fold compared to that of **24** (Table 6). Swapping out the 2-fluoro for a nitrogen to form the 6-CF_3_-pyridyl **26** resulted in a loss in both binding and cellular potency. In hopes to target the buried Met878 and increase the potency for these pyridyl substituents, the 4-amino substituent was installed on pyridyl **27**, which resulted in a 3-fold increase in binding potency compared to the parent **26** but showed no improvement in the cellular potency. Transposition of the nitrogen to form the 2-methyl-pyridyl **28** or replacement of the pyridyl ring with 3,4-substituted pyrazoles (**29** and **30**) resulted in a lower Cl_int_ in human liver microsomes, albeit with a significant loss in potency. Pushing out from the 3-position of the phenyl ring with a bromo substituent was well tolerated for SOS1 binding (**31**), and further introduction of the 3-cyano substituent resulted in the highly potent inhibitor of the SOS1:KRAS PPI, MRTX0902 (**32**), with a reduced lipophilicity (cLogP = 3.4). Replacement of the 3-cyano with a methyl sulfone (**33**, MKN1 IC_50_ = 242 nM) or deletion of the 2-methyl substituent (**34**, MKN1 IC_50_ = 333 nM) resulted in a significant drop in cellular potency when compared to that of MRTX0902 (**32**).

**Table 6:**
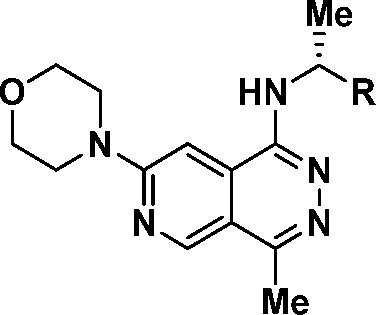

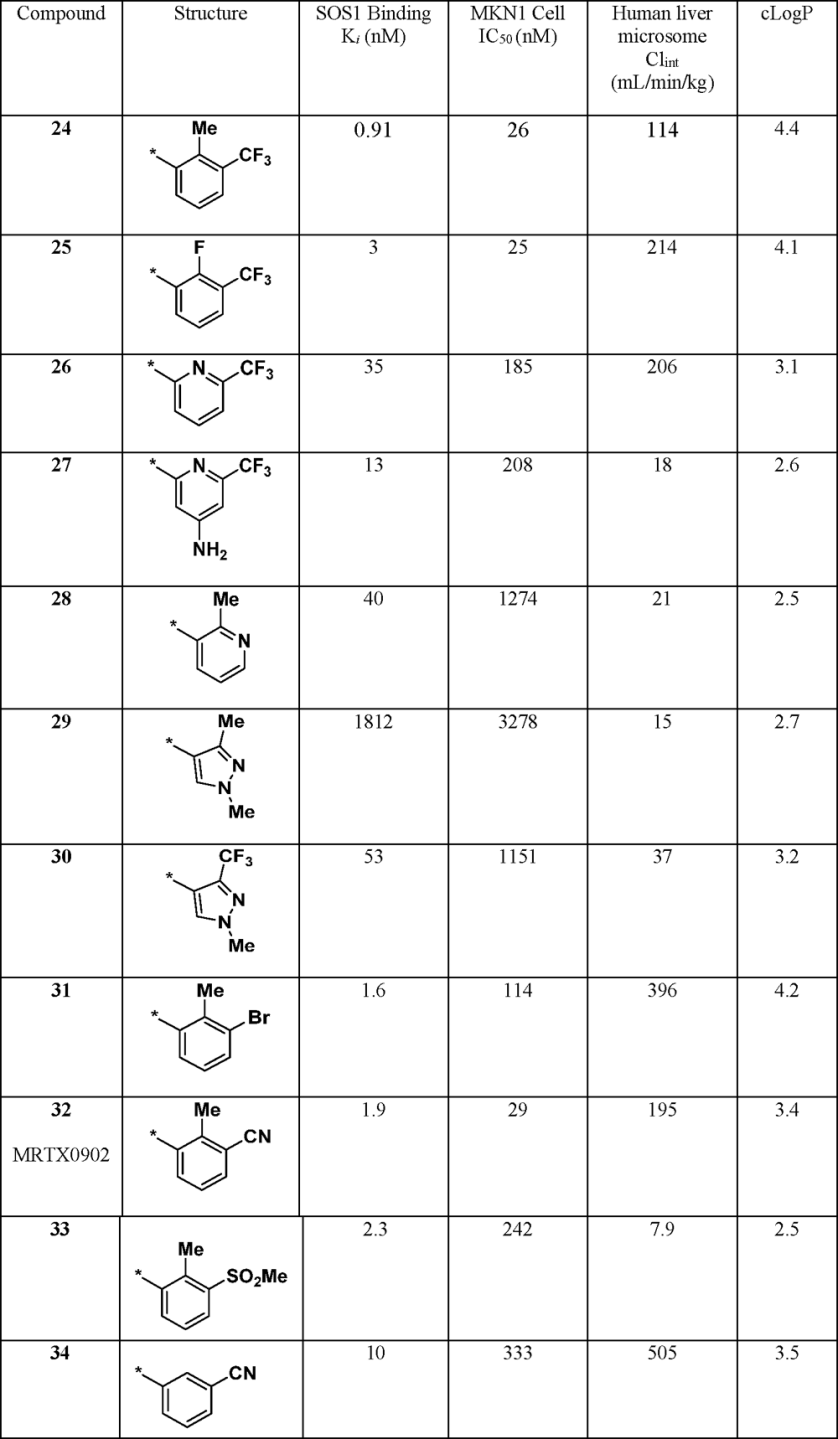
Selected SAR Data for Modifications of the C1-Benzyl Amine

**Figure 6:**
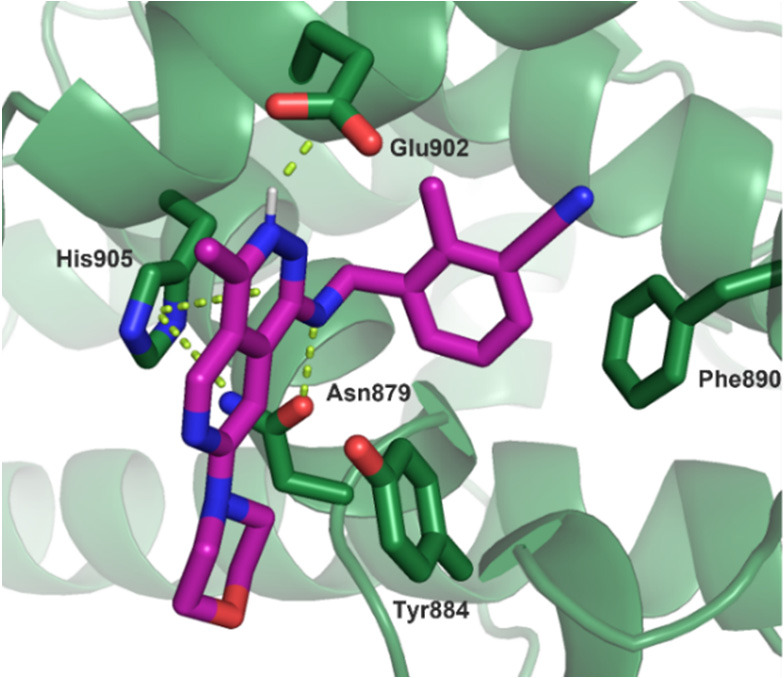
X-ray co-crystal structure of MRTX0902 (**32**) (PDB 7UKR).

A co-crystal structure of MRTX0902 (**32**) bound to SOS1 (Figure 6, PDB 7UKR) was obtained that showed key interactions similar to those of the previous co-crystal structure of compound **15**, including the salt bridge between the phthalazine core and Glu902. Based on the p*K*_a_ of MRTX0902 (measured p*K*_a_ = 6.7, ∼17% ionized at pH 7.4), the phthalazine is not highly protonated under physiological conditions; however, the local acidic environment of the protein likely helps drive protonation of the phthalazine core. The structure further revealed that the newly installed 3-cyano substituent on the phenyl ring presumably enhances the edge-to-face interaction with Phe890 due to the electron-withdrawing nature of the 3-cyano substituent. The substituted phenyl ring also provides shape complementary with the back pocket of SOS1, although the cyano substituent creates no discernible interactions with the protein as captured in the static structure. The SAR of the benzyl amine modifications show that the 3-cyano substituent in **32** provided the most effective combination of potency and lipophilicity and led to a nearly 6-fold decrease in CYP3A4 inhibition (IC_50_ = 3.6 μM) when compared to that of the 3-trifluoromethyl-substituted phenyl **24**. Furthermore, the co-crystal structure of MRTX0902 (**32**) suggested that the C6-position on the phthalazine could be used to further tune the electronics of the core structure. To this end, several electron-withdrawing substituents (-F, -Cl, -CN, -CF_3_) were installed onto the C6-position of the phthalazine ring; however, all modifications were met with a significant loss in both binding affinity and cellular potency (Table 7, **35**–**38**).

**Table 7:**
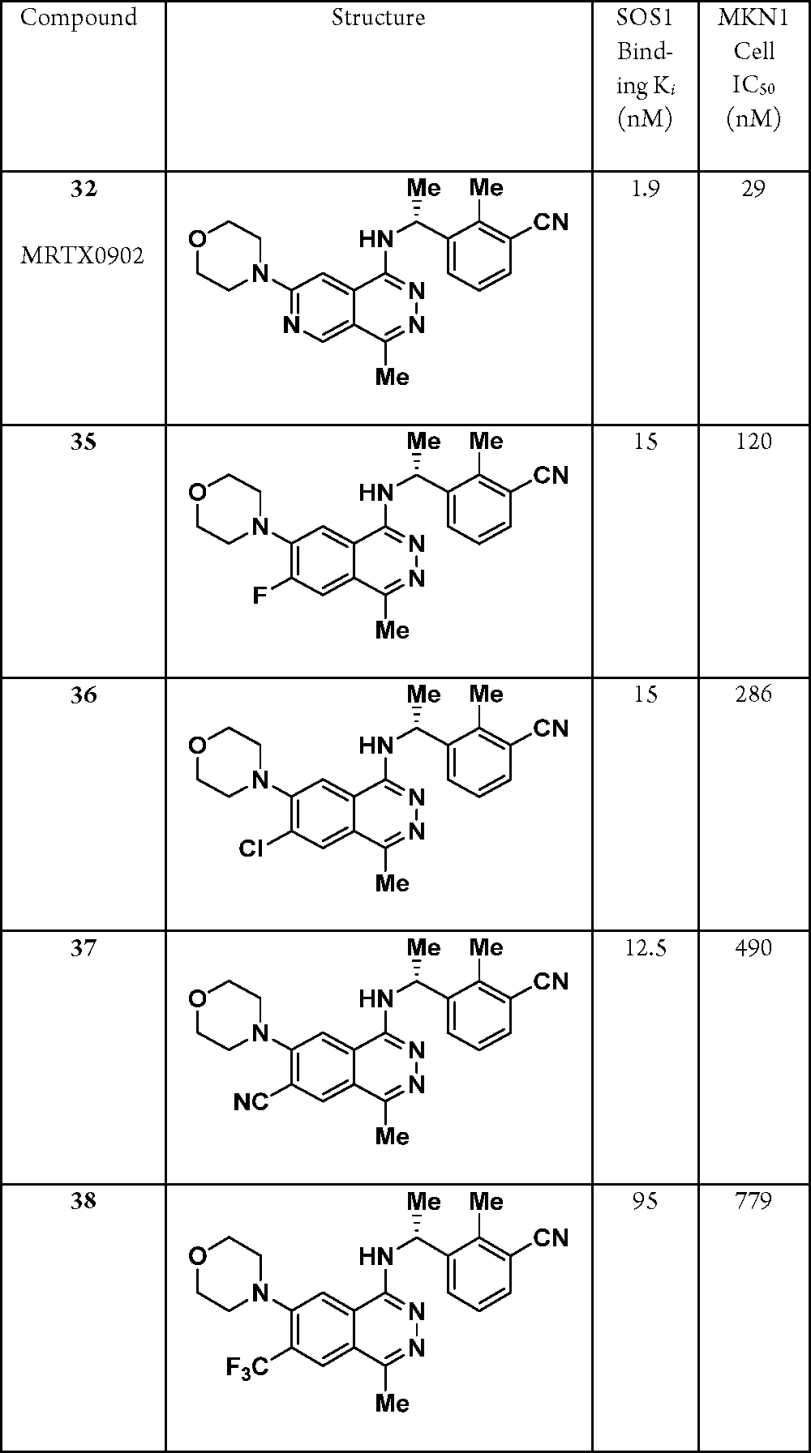
SAR Data of the C6-Position on the Phthalazine Core

Further in vitro profiling showed that MRTX0902 (**32**) was highly selective for SOS1 when compared to SOS2 and showed no inhibition of EGFR (Table 8). Additionally, a safety panel comprised of 78 protein targets revealed that MRTX0902 is highly selective for SOS1 (EC/IC_50_ > 10 μM for 74 targets; see Supporting Information for additional details). The PK properties of MRTX0902 after IV and PO dosing were evaluated in CD-1 mice, Sprague–Dawley rats, and beagle dogs (Table 9). Upon IV administration across species, MRTX0902 displayed a low clearance (4.4–14.6 mL/min/kg), low volume of distribution (0.28–0.48 L/kg), and a short half-life (0.62–1.3 h). PO administration of MRTX0902 as a homogeneous suspension in 0.5% MC (4000 cps)/0.2% Tween80 in water led to moderate to high bioavailability (38–83%F) in mice, rats, and dogs.

**Table 8:** In Vitro Profile of MRTX0902 (**32**)

assay	activity
SOS1 binding *K*_i_ (nM)	2
MKN1 cell IC_50_ (nM)	29
SOS2 KRAS^WT^ GDP exchange IC_50_ (nM)	>10 000
EGFR IC_50_ (nM)	>10 000
MW/cLogP/PSA	388.5/3.4/86.9
Caco-2 P_app_ A-to-B (10^–6^ cm/s)/efflux ratio^a^	32.3/1.5

a Caco-2 membrane permeability at 10 μM substrate concentration and pH 7.4.

**Table 9:** PK Parameters for MRTX0902 (**32**) across Species

PK parameters	mouse	rat	dog
Cl (mL/min/kg)	4.4	14.6	7.6
*V*_d,ss_ (L/kg)	0.28	0.28	0.48
IV *t*_1/2_ (h)	1.3	0.62	0.86
*F* (%)	69	83	38
dose, IV/PO (mg/kg)	3/30	1/10	2/10

Disease progression for *KRAS* mutant-driven cancers can lead to brain metastases;^59,60^ therefore, the concentrations of MRTX0902 (**32**) were measured in a central nervous system (CNS) mouse PK study. Both the total (mean brain, ng/g) and free (cerebral spinal fluid (CSF), nM) concentrations of MRTX0902 were measured after PO dosing in female CD-1 mice (Table 10), and the results demonstrated full coverage of the MKN1 cellular IC_50_ (29 nM) and efficacious free *C*_avg_ (25 nM, *vide infra*) in the CSF for up to 8 h. Importantly, drug exposure in the CSF is commonly used as a surrogate for unbound drug in the brain,^61^ and the observed free drug exposure in the brain as well as the efflux ratio in the Caco-2 assay (ER = 1.5; Table 8) appear to support the investigation of MRTX0902 in patients harboring *KRAS* mutant brain metastases.

**Table 10:** CNS Mouse PK Profile of MRTX0902 (**32**)^a^

time (h)	mean free plasma concn (*C*_p,u_, nM)	mean brain concn (ng/g)	mean CSF concn (nM)	efficacious free *C*_avg_ (nM)^b^	CSF:*C*_p,u_ (*K*_p,uu_)
1	134	1388	209		1.56
8	35	388	36		1.03
-				25	

a PO dosing: 100 mg/kg, single dose (*n* = 3).

b The efficacious free *C*_avg_ of MRTX0902 was calculated from the AUC_0–24_ (MRTX0902) of the 50 mg/kg bid dose in combination with MRTX849 in the MIA PaCa-2 PD study after 6 days of dosing (Figure 7).

The brain penetrance, low clearance, and high bioavailability of MRTX0902 (**32**) across species compelled its advancement to an in vivo antitumor efficacy study in the MIA PaCa-2 mouse model (*n* = 5 animals/group, Figure 7). Based on the short half-life of the compound in mice (*t*_1/2_ = 1.3 h), bid dosing was selected for these studies. After 25 days of PO dosing as a single agent, MRTX0902 resulted in 41% and 53% tumor growth inhibition (TGI) at 25 and 50 mg/kg bid, respectively. A sub-maximally efficacious dose of the KRAS^G12C^ inhibitor MRTX849 (10 mg/kg, qd) demonstrated nearly complete TGI (94%) as a single agent; however, this treatment did not result in tumor regression after 25 days of dosing. The combination of MRTX849 (10 mg/kg, qd) and MRTX0902 at 25 mg/k bid resulted in −54% regression, while nearly complete tumor regression (−92%) and two tumor-free animals were observed when MRTX0902 was dosed at 50 mg/kg bid with MRTX849 (10 mg/kg, qd). PD studies following 6 days of dosing demonstrated that the combination MRTX849 and MRTX0902 led to increased pERK modulation in tumors collected 4 h after the last dose. Additionally, the plasma concentration of MRTX849 remained unchanged when dosed in combination with MRTX0902.

**Figure 7:**
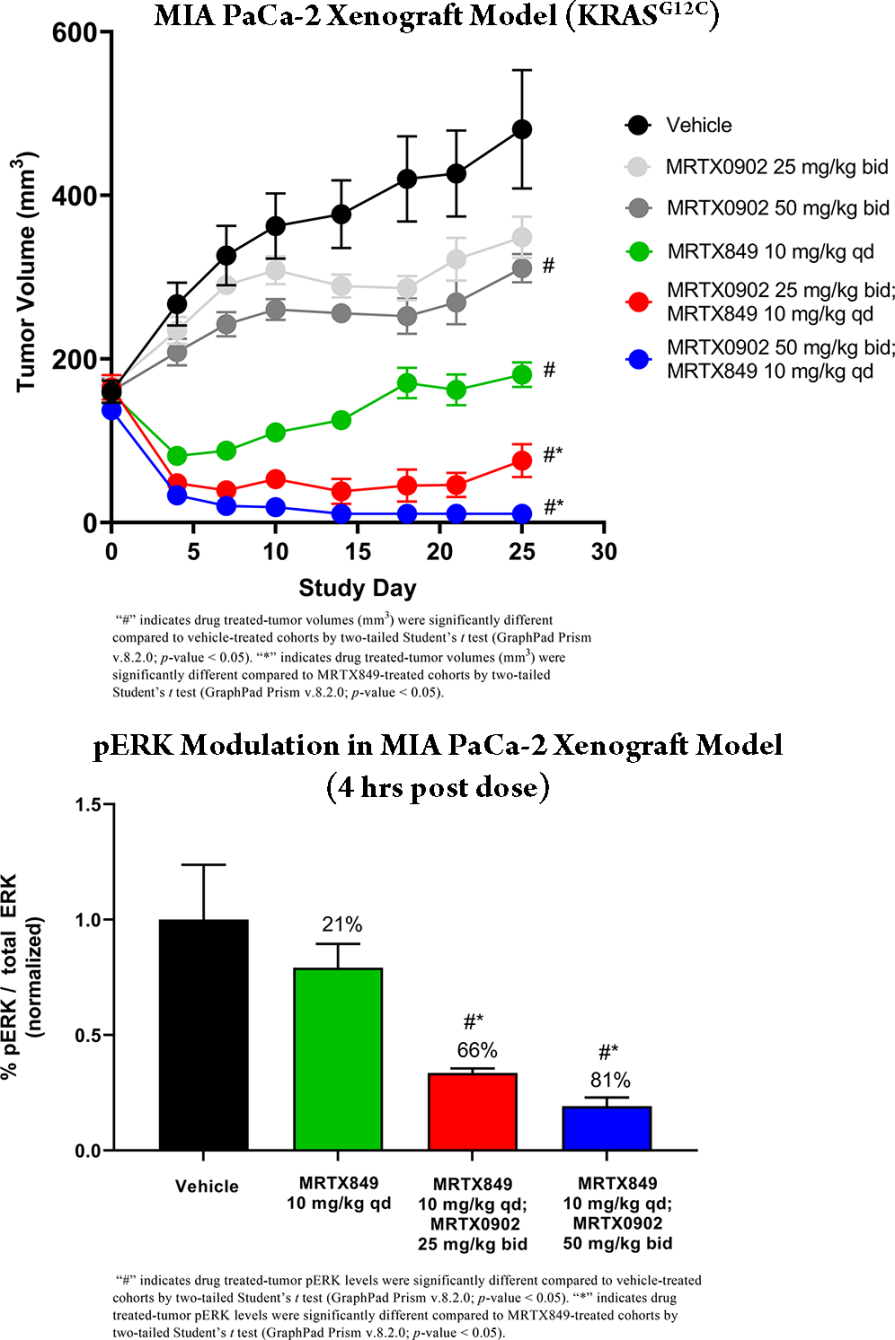
MRTX0902 (**32**) in vivo efficacy and PD in the MIA PaCa-2 mouse model.

### Chemistry

**Scheme 1:**
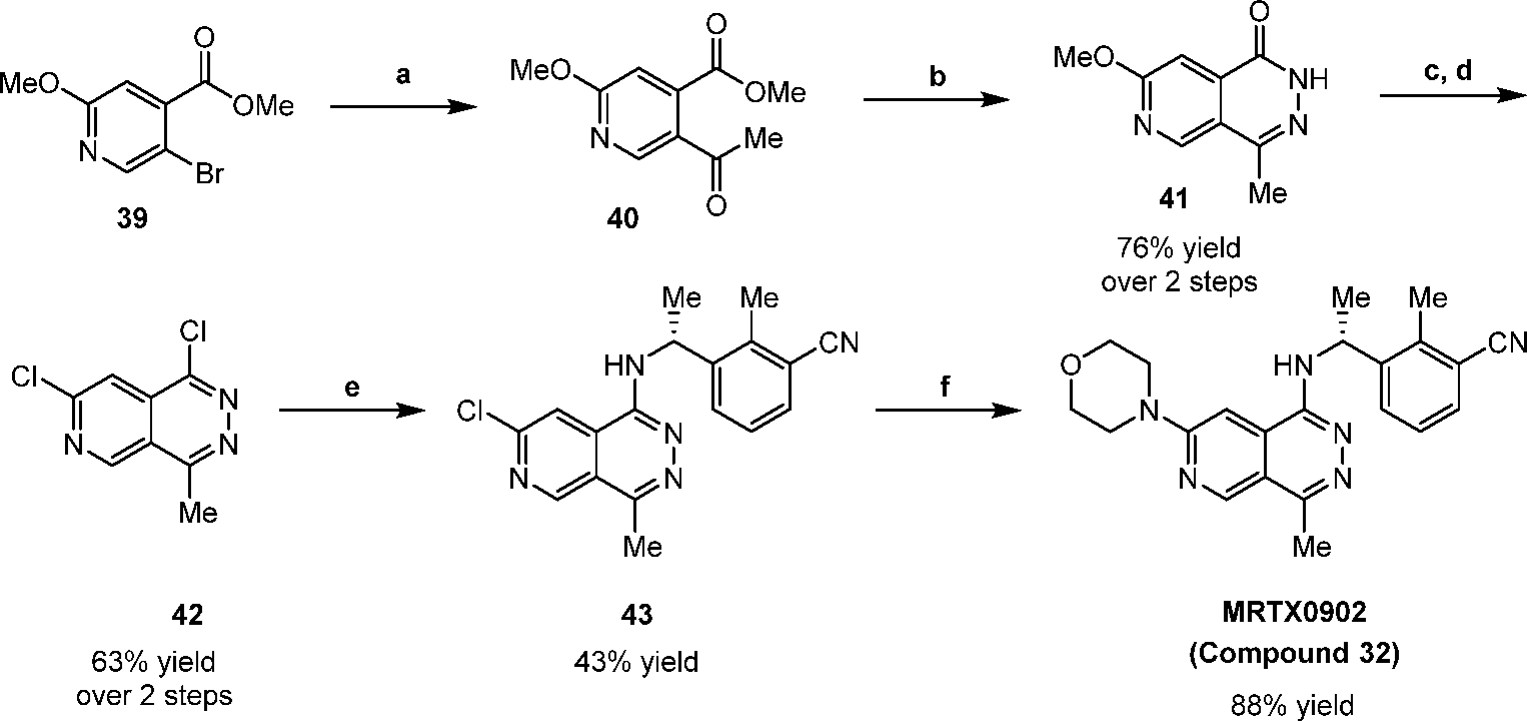
Synthesis of MRTX0902 (**32**) Reagents and conditions: (a) 1-(vinyloxy)butane, P(*t*-Bu)_3_Pd G2, *N,N*-dicyclohexylamine, dioxane, 85 °C, 10 h, then 4 N HCl, 40 °C, 2 h; (b) hydrazine monohydrate, EtOH, 70 °C, 10 h, 76% yield (over 2 steps); (c) 12 N HCl, 80 °C, 6 h; (d) POCl_3_, 100 °C, 5 h, 63% yield (over 2 steps); (e) (*R*)-3-(1-aminoethyl)-2-methylbenzonitrile, CsF, DMSO, 130 °C, 2 h, 43% yield; (f) morpholine, 110 °C, 1 h, 88% yield.

The synthesis of compound **32** (MRTX0902, Scheme 1) began with a Heck coupling between **39** and 1-(vinyloxy)butane to form the vinyl ether, followed by direct hydrolysis of the vinyl ether using hydrochloric acid to reveal methyl ketone **40**. The 6,6-core system was then constructed by cyclization of **40** with hydrazine hydrate to provide pyridopyridazinone **41**. Methyl ether cleavage of **41** followed by subsequent chlorination with POCl_3_ resulted in the formation of 1,7-dichloro-4-methylpyrido[3,4-*d*]pyridazine **42**. To complete the synthesis of **32**, an iterative S_N_Ar process was performed by first installing (*R*)-3-(1-aminoethyl)-2-methylbenzonitrile to form **43**, followed by heating **43** in morpholine to provide final compound **32** (MRTX0902).

## Conclusions

We have designed a novel phthalazine series of potent SOS1 binders that disrupt the protein–protein interaction between SOS1 and KRAS. X-ray co-crystal structures of these inhibitors reveal a unique salt bridge between the phthalazine core and Glu902 of SOS1 that has not been reported with previous SOS1 binders. Through rational design, the binding affinity (*K*_i_) of our initial lead **6** was increased more than 300-fold. Early ADME screening and molecular modeling efforts led to the introduction of a C4-methyl substituent to block AO-mediated metabolism, and the installation of the nitrogen at the 6-position of the core increased the bioavailability. These improvements provided the early in vivo tool compound **24**. Further optimization of the physiochemical properties led to the non-obvious introduction of the aryl nitrile and the discovery of MRTX0902 (**32**), a potent, selective, brain-penetrant, and orally bioavailable inhibitor of the SOS1:KRAS complex. In combination with our KRAS^G12C^ inhibitor MRTX849, MRTX0902 provides enhanced inhibition of the MAPK pathway and displays complete tumor regression in the MIA PaCa-2 tumor xenograft model. MRTX0902 has completed Investigational New Drug (IND)-enabling studies, and further profiling will be reported in due course.

## Experimental Section

### General Procedures

All final compounds were purified to ≥95% purity by either high-performance liquid chromatography (HPLC) or supercritical fluid chromatography (SFC). Purity was determined by HPLC, and additional structural characterization was performed by proton NMR, carbon NMR, and high-resolution mass spectrometry (HRMS) as described below. All chemicals were purchased from commercial suppliers and used as received unless otherwise indicated. Proton nuclear magnetic resonance (^1^H NMR) spectra were recorded on Bruker Avance 400 MHz spectrometers. Chemical shifts are expressed in δ parts per million (ppm) and are calibrated to the residual solvent peak: proton (e.g., CDCl_3_, 7.27 ppm). Coupling constants (*J*), when given, are reported in hertz. Multiplicities are reported using the following abbreviations: s = singlet, d = doublet, dd = doublet of doublets, t = triplet, q = quartet, m = multiplet (range of multiplet is given), br = broad signal, dt = doublet of triplets. Carbon nuclear magnetic resonance (^13^C NMR) spectra were recorded using a Bruker Avance HD spectrometer at 100 MHz. Chemical shifts are reported in δ ppm and are calibrated to the solvent peak: carbon (CDCl_3_, 77.23 ppm). The purity of test compounds was determined by HPLC on a Shimadzu LC-20AB instrument. HPLC conditions were as follows: Kinetex EVO C18 3.0 × 50 mm, 2.6 μm, 10%–80% ACN (0.0375% TFA) in water (0.01875% TFA), 3–10 min runs, flow rate 1.2 mL/min, UV detection (λ = 220, 215, 254 nm), or Kinetex C18 LC column 4.6 mm × 50 mm, 5 μm, 10%–80% ACN (0.0375% TFA) in water (0.01875% TFA), 4–10 min runs, flow rate 1.5 mL/min, UV detection (λ = 220, 215, 254 nm), or XBridge C18, 2.1 mm × 50 mm, 5 μm, 10%–80% ACN in water buffered with 0.025% ammonia, 4–10 min runs, flow rate 0.8 mL/min, UV detection (λ = 220, 215, 254 nm). The mass spectra were obtained using liquid chromatography–mass spectrometry (LC-MS) on a Shimadzu LCMS-2020 instrument using electrospray ionization (ESI). LC-MS conditions were as follows: Kinetex EVO C18 30 mm × 2.1 mm, 5 μm, 5%–95% ACN (0.0375% TFA) in water (0.01875% TFA), 1.5 min run, flow rate 1.5 mL/min, UV detection (λ = 220, 254 nm), or Kinetex EVO C18 2.1 mm × 30 mm, 5 μm, 5%–95% ACN in water buffered with 0.025% ammonia, 1.5 min run, flow rate 1.5 mL/min, and UV detection (λ = 220, 254 nm). HRMS measurements were carried out on a Agilent 1290LC and 6530Q-TOF series instruments with ESI. The SFC purity was determined with a Shimadzu LC-30ADsf instrument.

### Preparation of Compound **32** (MRTX0902)

#### Methyl 5-Acetyl-2-methoxyisonicotinate (**40**)

To a solution of **39** (300 g, 1.22 mol, 1.0 equiv) in dioxane (2.1 L) were added 1-(vinyloxy)butane (244 g, 2.44 mol, 2.0 equiv), P(*t*-Bu)_3_PdG2 (9.37 g, 18.3 mmol, 1.5 mol%), and *N*,*N*-dicyclohexylamine (262 g, 1.34 mol, 1.1 equiv), and the mixture was stirred at 85 °C for 10 h. After LCMS showed that the reaction was complete, the solution was cooled to room temperature, HCl (4 M in THF, 1.46 mol, 1.2 equiv) was added, and the solution was warmed to 40 °C and stirred for 2 h. The reaction mixture was diluted with H_2_O (500 mL), the pH of the mixture was adjusted to pH 8 with saturated NaHCO_3_ (in water), then the mixture was extracted with dichloromethane (1.0 L × 2). The combined organic layers were washed with brine, dried over Na_2_SO_4_, filtered, and concentrated under reduced pressure to give **40** (250 g, crude) as a yellow solid which was used without further purification.

#### 7-Methoxy-4-methylpyrido[3,4-*d*]pyridazin-1(2*H*)-one (**41**)

To a mixture of **40** (500 g, 2.39 mol, 1 equiv, crude) in EtOH (2.0 L) was added N_2_H_4_·H_2_O (169 g, 2.87 mol, 164 mL, 85% purity, 1.2 equiv) in one portion at 25 °C under a nitrogen atmosphere. The mixture was then heated to 70 °C and stirred for 10 h. After this time, the mixture was cooled to 25 °C and filtered, and the filter cake was collected and dried under reduced pressure to give **41** (350 g, 1.77 mol, 76% yield over 2 steps, 100% purity) as a black-brown solid. LCMS [M+1]^+^ = 192.2.

#### 1,7-Dichloro-4-methylpyrido[3,4-*d*]pyridazine (**42**)

A mixture of **41** (500 g, 2.62 mol, 1 equiv) in HCl (12 N in water, 2.0 L, 9.2 equiv) was prepared at 20 °C under a nitrogen atmosphere, and then the mixture was stirred at 80 °C for 6 h. The reaction mixture was then cooled to 25 °C and filtered, and the filter cake was concentrated under reduced pressure to give 7-hydroxy-4-methylpyrido[3,4-*d*]pyridazin-1(2*H*)-one (425 g, 2.40 mol, 92% yield, 99.8% purity) as a yellow solid. LCMS [M+1]^+^ = 178.0. ^1^H NMR (400 MHz, DMSO-*d*_6_) δ 12.53 (s, 1H), 8.41 (s, 1H), 6.92 (s, 1H), 2.35 (s, 3H). A solution of 7-hydroxy-4-methylpyrido[3,4-*d*]pyridazin-1(2*H*)-one (460 g, 2.60 mol, 1 equiv) in POCl_3_ (1.99 kg, 13.0 mol, 1.21 L, 5 equiv) was stirred at 100 °C for 5 h. After this time, the volatiles were removed via reduced pressure to give a residue. The residue was dissolved in dichloromethane (2.0 L), and the pH of the mixture was adjusted to pH 8 at 0 °C with saturated NaHCO_3_ (in water). The mixture was then filtered, and the filtrate was extracted with dichloromethane (2.0 L × 2). The combined organic layers were washed with brine, dried over Na_2_SO_4_, filtered, and concentrated under reduced pressure to give **42** (352 g, 1.64 mol, 63% yield, 94.9% purity) as an orange solid. LCMS [M+1]^+^ = 214.1. ^1^H NMR (400 MHz, DMSO-*d*_6_) δ 9.37 (s, 1H), 8.19 (s, 1H), 3.01 (s, 3H).

#### (*R*)-3-(1-((7-Chloro-4-methylpyrido[3,4-*d*]pyridazin-1-yl)amino)ethyl)-2-methylbenzonitrile (**43**)

To a solution of (*R*)-3-(1-aminoethyl)-2-methylbenzonitrile (16.0 g, 99.9 mmol, 1.00 equiv) and **42** (21.4 g, 99.9 mmol, 1.00 equiv) in DMSO (130 mL) was added cesium fluoride (22.8 g, 150 mmol, 5.52 mL, 1.50 equiv), and the mixture was stirred at 130 °C for 2 h. The reaction was cooled to 25 °C and then poured into water (200 mL). The aqueous phase was extracted with ethyl acetate (200 mL × 3). The combined organic phases were washed with brine (100 mL × 3), dried over anhydrous sodium sulfate, filtered, and concentrated under reduced pressure to give a residue. The residue was purified by prep-HPLC (Kromasil Eternity XT 250 × 80 mm × 10 μm; mobile phase A: 0.1% TFA in water, mobile phase B: acetonitrile; B%: 25%–55%). To the aqueous phase was added sodium bicarbonate to adjust the pH to 8, and then the suspension was extracted with ethyl acetate (1000 mL × 3). The combined organic phases were washed with brine (100 mL × 3), dried over anhydrous sodium sulfate, filtered, and concentrated under reduced pressure to give **43** (14.5 g, 42.9 mmol, 43% yield) as a yellow solid. ^1^H NMR (400 MHz, CDCl_3_) δ = 9.19 (d, *J* = 0.4 Hz, 1H), 7.74 (s, 1H), 7.63 (d, *J* = 8.0 Hz, 1H), 7.50 (dd, *J* = 1.2, 7.6 Hz, 1H), 7.23 (t, *J* = 7.6 Hz, 1H), 5.72 (quin, *J* = 6.8 Hz, 1H), 5.40 (br d, *J* = 6.0 Hz, 1H), 2.86 (s, 3H), 2.69 (s, 3H), 1.63 (s, 3H).

#### MRTX0902, (*R*)-2-Methyl-3-(1-((4-methyl-7-morpholinopyrido[3,4-*d*]pyridazin-1-yl)amino)ethyl)benzonitrile (**32**)

A solution of **43** (13.5 g, 40.0 mmol, 1.00 equiv) in morpholine (10.4 g, 120 mmol, 10.6 mL, 3.00 equiv) was stirred at 110 °C for 1 h. The reaction mixture was cooled to 25 °C, poured into water (15.0 mL), and stirred for 5 min. After this time the mixture was filtered, and the filter cake was dried under reduced pressure to give a residue. The residue was washed with water (15.0 mL × 3) to give MRTX0902 (**32**) (14.0 g, 35.3 mmol, 88% yield, 98.9% purity) as a yellow solid. ^1^H NMR (400 MHz, DMSO-*d*_6_) δ = 8.96 (s, 1H), 7.71 (d, *J* = 8.0 Hz, 1H), 7.63–7.48 (m, 2H), 7.38 (s, 1H), 7.30 (t, *J* = 8.0 Hz, 1H), 5.54–5.49 (m, 1H), 3.80–3.71 (m, 4H), 3.67–3.65 (m, 4H), 2.62 (s, 3H), 2.54 (s, 3H), 1.52 (d, *J* = 7.2 Hz, 3H). ^13^C NMR (101 MHz, DMSO-*d*_6_) δ = 159.79, 151.20, 149.42, 147.56, 146.39, 139.20, 131.25, 129.71, 127.41, 125.10, 119.01, 114.44, 112.69, 93.62, 66.30, 47.06, 45.49, 21.86, 18.47, 17.20. HRMS (*m*/*z*): [M+H]^+^ calcd for C_22_H_24_N_6_O, 389.2012; found, 389.2105. HPLC (0.025% NH_3_·H_2_O in water): *t*_R_ = 11.621 min (98.9% purity).

## Supporting Information Available

Synthetic experimental procedures for compounds **6**–**38**; NMR spectra and HPLC trace of final compounds; SOS1 X-ray structures for compounds **15** and **32**; biochemical and cellular assay protocols; in vivo PK, PD, and TGI studies (PDF)
Molecular formula strings (CSV)

## Abbreviations Used

bid: bis in die (twice a day)
Cmpd: compound
CNS: central nervous system
CSF: cerebral spinal fluid
EGFR: epidermal growth factor receptor
IV: intravenous
KRAS: Kirsten rat sarcoma virus
MC: methyl cellulose
PD: pharmacodynamics
PK: pharmacokinetics
PO: per os (oral)
qd: quaque die (once daily)
SAR: structure–activity relationship
SFC: supercritical fluid chromatography
SOS1: son of sevenless homolog 1
TGI: tumor growth inhibition
Tween80: polyoxyethylene sorbitan monooleate
