# Supplementary material for: Design and Discovery of MRTX0902, a Potent, Selective, Brain-Penetrant, and Orally Bioavailable Inhibitor of the SOS1:KRAS Protein–Protein Interaction
Source: J Med Chem. 2022 Jul 14;65(14):9678–90. doi: 10.1021/acs.jmedchem.2c00741 (PMC9340770; doi:10.1021/acs.jmedchem.2c00741)
Supplement: Supplementary file 1 — jm2c00741_si_001.pdf [file jm2c00741_si_001.pdf]

Supporting Information

Design and Discovery of MRTX0902, a Potent, Selective, Brain-Penetrant, and Orally Bioavailable Inhibitor of the SOS1:KRAS Protein-Protein Interaction

John M. Ketcham, Jacob Haling, Shilpi Khare, Vickie Bowcut, David M. Briere, Aaron C. Burns, Robin J. Gunn, Anthony Ivetac, Jon Kuehler, Svitlana Kulyk, Jade Laguer, J. David Lawson, Krystal Moya, Natalie Nguyen, Lisa Rahbaek, Barbara Saechao, Christopher R. Smith, Niranjan Sudhakar, Nicole C. Thomas, Laura Vegar, Darin Vanderpool, Xiaolun Wang, Larry Yan, Peter Olson, James G. Christensen, Matthew A. Marx*

AUTHORS ADDRESS: Mirati Therapeutics, 3545 Cray Court, San Diego, California 92121, United States

Corresponding author email:
John Ketcham, ketchamj@mirati.com

Table of Contents

General Experiment S2

NMR Spectra and HPLC Traces of Final Compounds....................................S66

HTRF Binding Assay....................................S99

Cellular Assays....................................S99

EGFR and SOS2 Assays....................................S100

Aldehyde Oxidase Metabolism Assay....................................S101

Tumor Pharmacodynamic and Tumor Xenograft Studies....................................S101

X-Ray Co-Crystal Structure S105

Table S2. MRTX0902 inhibition profile across DiscoverX (Eurofins) Safetyscan Panel…....S106

pKa Determination and Caco-2 Assay....................................S110

References S111

Synthesis of Compounds 6-11

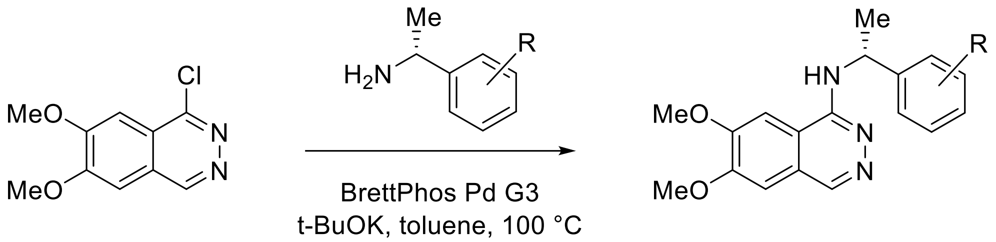

General Procedure A: A mixture of 1-chloro-6,7-dimethoxyphthalazine (1.00 *eq.*), a chiral α-methyl benzyl amine (0.70 *eq.*), BrettPhos Pd G3 (0.10 *eq.*) and potassium *tert*-butoxide (2.50 *eq.*) in toulene (3.00 mL) was degassed and purged with nitrogen 3 times, then the reaction mixture was stirred at 100 °C for 1 hour under a nitrogen atmosphere. The reaction mixture was then cooled to 25 °C, filtered, and the filtrate was concentrated under reduced pressure to give a residue. The residue was purified by prep-TLC (SiO_2, dichloromethane/methanol) or prep-HPLC (acidic, neutral, or basic conditions) to give the desired product.

The following compounds were prepared following **General Procedure A**:

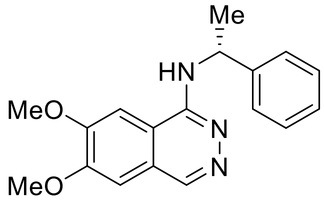

(*R*)-6,7-dimethoxy-*N*-(1-phenylethyl)phthalazin-1-amine (6)

Yellow solid, 99% purity, ^{1}H NMR (400 MHz, DMSO-d_6) δ = 8.70 (s, 1H), 7.83 (s, 1H), 7.44 (d, *J* = 7.2 Hz, 2H), 7.39 (br d, *J* = 8.0 Hz, 1H), 7.33 (s, 1H), 7.29 (t, *J* = 7.6 Hz, 2H), 7.21 - 7.13 (m, 1H), 5.60 (quin, *J* = 7.2 Hz, 1H), 3.99 (s, 3H), 3.91 (s, 3H), 1.59 (d, *J* = 7.2 Hz, 3H). ^{13}C NMR (101 MHz, DMSO-d_6) δ = 152.99, 152.93, 152.83, 146.45, 142.72, 128.54, 126.71, 126.54, 123.74, 113.26, 105.92, 102.44, 56.86, 56.52, 56.26, 49.92, 23.62, 19.01. HRMS (m/ z): [M + H]+ calcd for $C_{18}H_{19}N_3O_2$, 310.1477; found, 310.1563. HPLC (0.025% ammonium hydroxide in water): t_R = 10.788 min (99.0% purity).

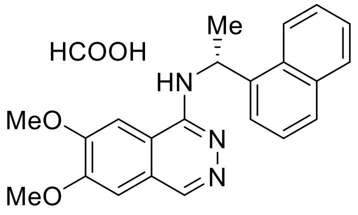

(*R*)-6,7-dimethoxy-*N*-(1-(naphthalen-1-yl)ethyl)phthalazin-1-amine formic acid salt (7)

^{1}H NMR (400 MHz, CD_3OD): δ = 8.70 (s, 1H), 8.49 (s, 1H), 8.15 (d, *J* = 8.0 Hz, 1H), 7.83 (d, *J* = 8.0 Hz, 1H), 7.78 (s, 1H), 7.73 (d, *J* = 8.0 Hz, 1H), 7.62 (d, *J* = 6.8 Hz, 1H), 7.47 - 7.35 (m, 4H), 6.22 (q, *J* = 6.4 Hz, 1H), 3.99 (s, 3H), 3.94 (s, 3H), 1.78 (d, *J* = 6.8 Hz, 3H). ^{13}C NMR (400 MHz, CD_3OD): δ ppm 155.75, 155.38, 140.83, 135.57, 132.79, 129.93, 128.90, 127.10, 126.67, 126.49, 124.59, 123.33, 115.91, 107.55, 103.51, 57.13, 56.88, 48.26, 21.46. HRMS (*m/z*): $[M + H]^+$ calcd for $C_{22}H_{21}N_3O_2$, 360.1634; found, 360.1727. HPLC (0.025% ammonium hydroxide in water): t_R = 12.406 min (97.6% purity).

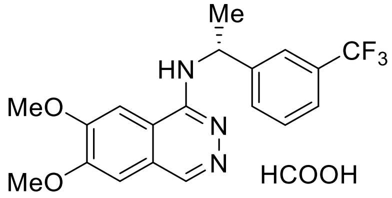

(R)-6,7-dimethoxy-N-(1-(3-(trifluoromethyl)phenyl)ethyl)phthalazin-1-amine formic acid salt (8)

^{1}H NMR (400 MHz, DMSO-d_6) δ = 8.71 (s, 1H), 8.19 (s, 1H), 7.84 - 7.73 (m, 3H), 7.59 - 7.46 (m, 3H), 7.34 (s, 1H), 5.68 - 5.56 (m, 1H), 4.00 (s, 3H), 3.91 (s, 3H), 1.62 (d, *J* = 6.8 Hz, 3H). ^{13}C NMR (101 MHz, DMSO-d_6) δ =153.06, 153.03, 152.64, 148.19, 142.99, 130.72, 129.67, 129.35 (q, *J* = 31.5 Hz, 1C), 124.86 (q, *J* = 273.1 Hz, 1C), 123.74, 123.56 (q, *J* = 3.3 Hz, 1C), 122.95 (q, *J* = 3.3 Hz, 1C), 113.23, 105.99, 102.34, 56.86, 56.29, 50.01, 23.62. HRMS (m/ z): [M + H]+ calcd for $C_{19}H_{18}F_3N_3O_2$, 378.1351; found, 378.1339. HPLC (A: 0.0375% TFA in water, B: 0.01875% TFA in Acetonitrile): t_R = 4.148 min (94.5% purity).

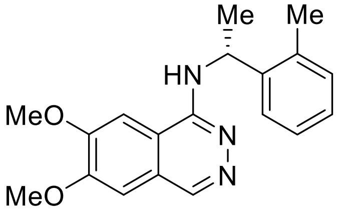

(*R*)-6,7-dimethoxy-*N*-(1-(*o*-tolyl)ethyl)phthalazin-1-amine (9)

^{1}H NMR (400 MHz, DMSO-d_6) δ = 8.68 (s, 1H), 7.84 (s, 1H), 7.48 (d, *J* = 7.2 Hz, 1H), 7.40 (d, *J* = 7.6 Hz, 1H), 7.31 (s, 1H), 7.15 - 7.04 (m, 3H), 5.69 (quin, *J* = 7.2 Hz, 1H), 3.98 (s, 3H), 3.90 (s, 3H), 2.44 (s, 3H), 1.53 (d, *J* = 6.8 Hz, 3H). ^{13}C NMR (101 MHz, DMSO-d_6) δ = 152.97, 152.90,

152.63, 144.72, 142.65, 135.49, 130.39, 126.58, 126.35, 124.94, 123.68, 113.27, 105.90, 102.46, 56.87, 56.25, 46.79, 22.23, 19.26. HRMS (m/ z): [M + H] + calcd for $C_{19}H_{21}N_3O_2$, 324.1634; found, 324.1722. HPLC (A: 0.0375% TFA in water (v/v), B: 0.01875% TFA in Acetonitrile (v/v)): t_R =3.823 min (97.6% purity).

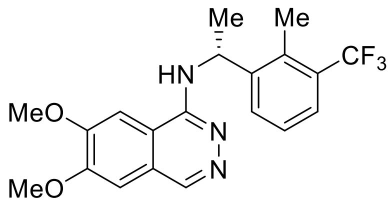

(*R*)-6,7-dimethoxy-*N*-(1-(2-methyl-3-(trifluoromethyl)phenyl)ethyl)phthalazin-1-amine (10)

^{1}H NMR (400 MHz, $CDCl_3$) δ = 8.80 (s, 1H), 7.64 (d, *J* = 7.6 Hz, 1H), 7.52 (d, *J* = 8.0 Hz, 1H), 7.20 (t, *J* = 7.6 Hz, 1H), 7.08 (s, 1H), 7.05 (s, 1H), 5.95 - 5.91 (m, 1H), 5.22 (d, *J* = 6.4 Hz, 1H), 4.04 (s, 3H), 3.95 (s, 3H), 2.53 (s, 3H), 1.64 (d, *J* = 6.8 Hz, 3H). ^{13}C NMR (101 MHz, DMSO-d_6): δ ppm 153.04, 152.99, 152.45, 147.57, 142.91, 134.02, 129.24, 128.01 (q, *J* = 28.7 Hz, 1C), 126.60, 125.32 (q, *J* = 274.11 Hz, 1C), 124.22 (q, *J* = 5.7 Hz, 1C), 123.69, 113.22, 105.96, 102.39, 56.88, 56.27, 46.77, 22.23, 14.60 (d, *J* = 2.5 Hz, 1C). HRMS (m/ z): [M + H]+ calcd for $C_{20}H_{20}F_3N_3O_2$, 392.1508; found, 392.1596. HPLC (0.025% ammonium hydroxide in water): t_R = 13.129 min (96.4 % purity).

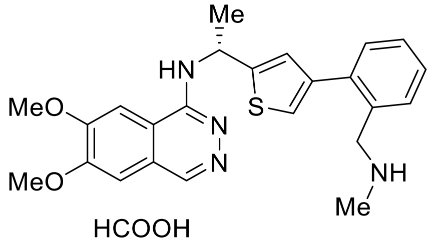

(*R*)-6,7-dimethoxy-*N*-(1-(4-(2-((methylamino)methyl)phenyl)thiophen-2-yl)ethyl)phthalazin-1-amine formic acid salt (11)

^{1}H NMR (400 MHz, DMSO-d_6) δ = 8.78 (s, 1H), 8.25 (s, 2H), 7.77 (s, 1H), 7.53 (br d, *J* = 4.0 Hz, 2H), 7.42 (s, 1H), 7.40 - 7.29 (m, 4H), 7.26 (s, 1H), 5.99 (br s, 1H), 3.96 (s, 3H), 3.93 (s, 3H), 3.83 (s, 2H), 2.35 (s, 3H), 1.75 (d, *J* = 6.8 Hz, 3H).^{13}C NMR (101 MHz, DMSO-d_6) δ = 165.43, 153.05, 152.81, 150.63, 143.08, 139.81, 137.32, 132.39, 130.38, 129.81, 128.69, 128.00, 125.83, 123.87, 122.41, 113.34, 105.98, 102.47, 56.82, 56.30, 50.26, 45.78, 33.70, 22.69. HRMS (m/ z): $[M + H]^+$:

calcd for $C_{24}H_{26}N_4O_2S$, 435.1776; found, 435.1865. HPLC (A: 0.0375% TFA in water, B: 0.01875% TFA in Acetonitrile): t_R = 3.022 min (95.6% purity).

(*R*)-6,7-dimethoxy-4-methyl-*N*-(1-(4-(2-((methylamino)methyl)phenyl)thiophen-2-yl)ethyl)phthalazin-1-amine (12)

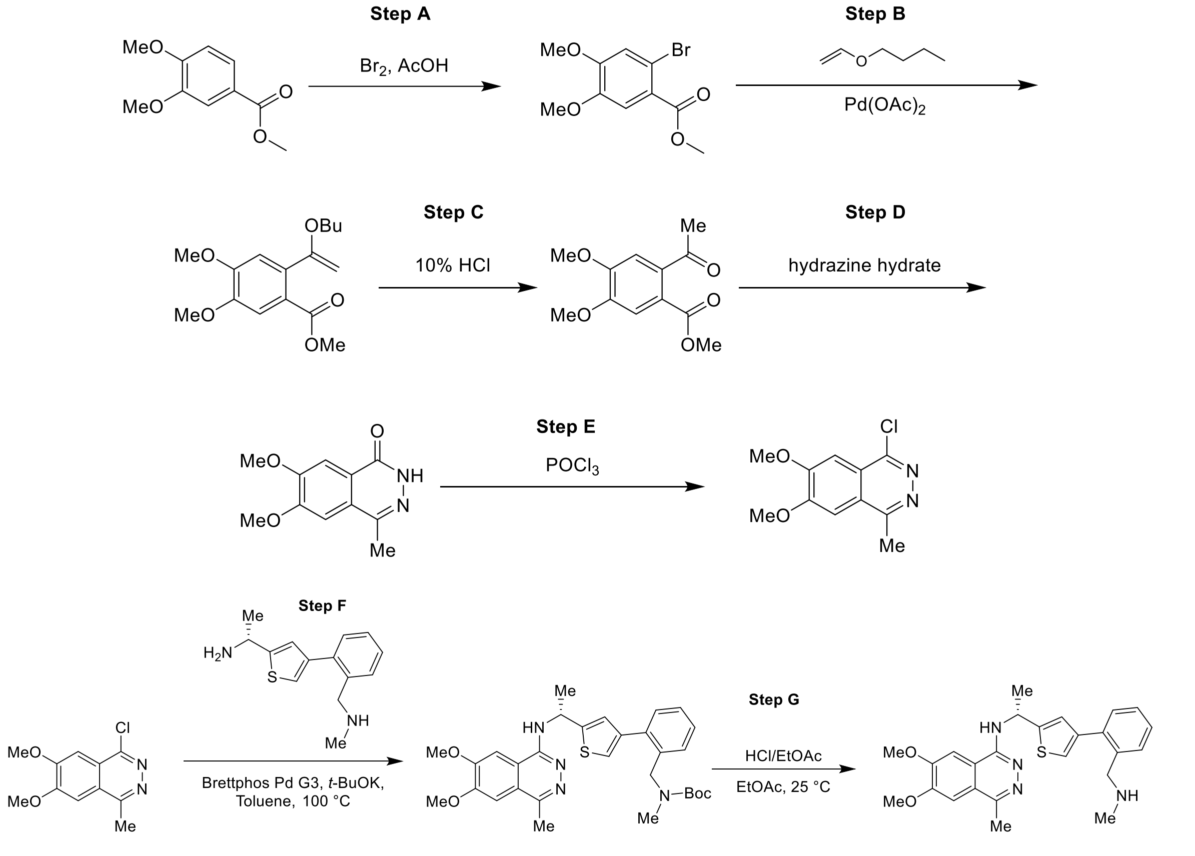

Compound 12

Step A: To a mixture of methyl 3,4-dimethoxybenzoate (10.0 g, 51.0 mmol, 1.00 *eq.*) in acetic acid (50.0 mL) was added bromine (8.96 g, 56.1 mmol, 2.89 mL, 1.10 *eq.*) in acetic acid (50.0 mL) at 0 °C over 1.5 hours. The mixture was then slowly brought to room temperature and stirred for 45 minutes. Upon completion, the reaction was quenched by pouring into water (700 mL) and stirred for 30 minutes, then stirring was stopped and the mixture was filtered after 1 hr of sitting. The collected solid was washed with water (100 mL) and washed with sodium sulfite aqueous solution (100 mL). The solid was partially dried, dissolved in hot methanol (300 mL), and the resultant solution was cooled. The cool methanolic solution was treated with water (200 mL) to give a suspension, the suspension was filtered, the filter cake was collected and dried *in vacuo* to

give methyl 2-bromo-4,5-dimethoxybenzoate (9.00 g, 32.7 mmol, 64.2% yield) as a white powder. LCMS [M+1]: 275.3.

^{1}H NMR (400 MHz, DMSO-d_6) δ = 7.36 (s, 1H), 7.24 (s, 1H), 3.84 (s, 3H), 3.82 (s, 3H), 3.79 (s, 3H).

Step B: A mixture of methyl 2-bromo-4,5-dimethoxy-benzoate (6.00 g, 21.8 mmol, 1.00 eq.), 1-(vinyloxy)butane (10.9 g, 109 mmol, 14.0 mL, 5.00 eq.), $Pd(OAc)_2$ (490 mg, 2.18 mmol, 0.10 eq.), triphenylphosphine (1.14 g, 4.36 mmol, 0.20 eq.) and triethylamine (2.65 g, 26.2 mmol, 3.64 mL, 1.20 eq.) in acetonitrile (60.0 mL) was degassed and purged with nitrogen 3 times, and then the reaction mixture was stirred at 100 °C for 16 hours under a nitrogen atmosphere. The mixture was then cooled to 25 °C, filtered, and the filtrate concentrated under reduced pressure to give methyl methyl 2-(1-butoxyvinyl)-4,5-dimethoxybenzoate (6.00 g, crude) was obtained as a yellow oil which was used in the next step directly.

Step C: A mixture of methyl 2-(1-butoxyvinyl)-4,5-dimethoxybenzoate (6.00 g, 20.4 mmol, 1.00 eq.) in hydrochloric acid (10% in water, 61.2 g, 168 mmol, 60.0 mL, 8.23 eq.) and THF (60.0 mL) was stirred at 20 °C for 1 hour. The reaction mixture was diluted with water (100 mL) and extracted with ethyl acetate (50.0 mL × 3). The combined organic layers were brought to pH = 7 with a saturated sodium bicarbonate aqueous solution, then the organic layers were washed with brine (100 mL), dried over anhydrous sodium sulfate, filtered, and concentrated under reduced pressure to give a residue. The residue was triturated with petroleum ether/ethyl acetate = 5/１ (50.0 mL) at 20 °C for 20 minutes to give a suspension, the suspension was filtered, the filter cake was collected and dried in vacuo to give methyl 2-acetyl-4,5-dimethoxybenzoate (3.00 g, 12.6 mmol, 61.8% yield) as a white solid.

^{1}H NMR (400 MHz, DMSO-d6) δ = 7.26 (s, 1H), 7.17 (s, 1H), 3.86 (s, 3H), 3.84 (s, 3H), 3.77 (s, 3H), 2.46 (s, 3H).

Step D: To a solution of methyl 2-acetyl-4,5-dimethoxybenzoate (3.00 g, 12.6 mmol, 1.00 eq.) in ethanol (30.0 mL) was added hydrazine hydrate (2.22 g, 37.8 mmol, 2.16 mL, 3.00 eq.) at room temperature, and then the reaction mixture was stirred at 95 °C for 30 minutes. The reaction mixture was diluted with water (100 mL) and extracted with ethyl acetate several times. The combined organic layers were washed with brine (100 mL), dried over anhydrous sodium sulfate,

filtered, and concentrated under reduced pressure to give a residue. The residue was triturated with ethyl acetate (50.0 mL) at 20 °C for 20 minutes to give a suspension, the suspension was filtered, the filter cake was collected and dried in vacuo to give 6,7-dimethoxy-4-methylphthalazin-1(2H)-one (2.00 g, 9.08 mmol, 72.1% yield) as a off-white solid. LCMS [M+1]: 221.4.

^{1}H NMR (400 MHz, DMSO-d6) δ = 12.25 (s, 1H), 7.58 (s, 1H), 7.21 (s, 1H), 3.96 (s, 3H), 3.92 (s, 3H), 2.48 (s, 3H).

Step E: A mixture of 6,7-dimethoxy-4-methylphthalazin-1(2H)-one (1.30 g, 5.90 mmol, 1.00 eq.) in phosphorus (V) oxychloride (13.0 mL) was stirred at 120 °C for 12 hours. The reaction mixture was concentrated under reduced pressure to give 1-chloro-6,7-dimethoxy-4-methylphthalazine (1.20 g, crude) as a yellow solid. LCMS [M+1]: 239.0.

^{1}H NMR (400 MHz, DMSO-d6) δ = 7.80 (s, 1H), 7.64 (s, 1H), 4.13 (s, 3H), 4.12 (s, 3H), 3.08 (s, 3H).

Step F: A mixture of 1-chloro-6,7-dimethoxy-4-methylphthalazine (150 mg, 481.40 µmol, 1.00 *eq.*), tert-butyl (*R*)-(2-(5-(1-aminoethyl)thiophen-3-yl)benzyl)(methyl)carbamate (133 mg, 385 µmol, 0.80 *eq.*), potassium *tert*-butoxide (1.00 M, 1.44 mL, 3.00 *eq.*) and BrettPhos Pd G3 (43.6 mg, 48.1 µmol, 0.10 *eq.*) in toluene (4.00 mL) was degassed and purged with nitrogen 3 times, then the reaction mixture was stirred at 100 °C for 12 hours under a nitrogen atmosphere. The reaction mixture was cooled to 20 °C. The reaction mixture was then filtered and concentrated under reduced pressure to give a residue. The residue was purified by prep-TLC (SiO_2, dichloromethane/methanol = 10/1) to give *tert*-butyl (*R*)-(2-(5-(1-((6,7-dimethoxy-4-methylphthalazin-1-yl)amino)ethyl)thiophen-3-yl)benzyl)(methyl)carbamate (100 mg, 182 µmol, 37.9% yield) as a yellow solid. LCMS $[M+1]^+$: 549.3.

Step G: A solution of *tert*-butyl (*R*)-(2-(5-(1-((6,7-dimethoxy-4-methylphthalazin-1-yl)amino)ethyl)thiophen-3-yl)benzyl)(methyl)carbamate (60.0 mg, 109 µmol, 1.00 *eq.*) in ethyl acetate (0.50 mL) was added dropwise hydrochloric acid (4.00 M in ethyl acetate, 2.00 mL, 73.2 *eq.*), and the reaction mixture was stirred at 20 °C for 15 minutes. The reaction mixture was then quenched by addition of saturated sodium bicarbonate aqueous solution (10.0 mL) at 0 °C and further extracted with ethyl acetate (10.0 mL × 6). The combined organic layers were washed with brine (20.0 mL), dried over anhydrous sodium sulfate, filtered, and concentrated under reduced

pressure to give a residue. The residue was purified by prep-HPLC (column: Waters Xbridge BEH C18 100 × 25 mm × 5 µm; mobile phase: phase A: 10mM NH_4HCO_3 in water, phase B: acetonitrile; B%: 25% - 55%) to give 6,7-dimethoxy-4-methyl-*N*-[(1*R*)-1-[4-[2-(methylaminomethyl)phenyl]-2-thienyl]ethyl] phthalazin-1-amine (7.72 mg, 17.2 µmol, 16% yield, 98.6% purity) as a white solid.

^{1}H NMR (400 MHz, CD_3OD) δ = 7.69 (s, 1H), 7.39 (d, *J* = 6.8 Hz, 1H), 7.32 - 7.26 (m, 4H), 7.14 (s, 2H), 5.90 (q, *J* = 6.8 Hz, 1H), 4.03 (s, 3H), 4.01 (s, 3H), 3.70 (s, 2H), 2.70 (s, 3H), 2.20 (s, 3H), 1.80 (d, *J* = 6.8 Hz, 3H). ^{13}C NMR (101 MHz, CD_3OD) δ = 154.65, 154.39, 154.21, 151.45, 149.44, 142.04, 138.51, 137.59, 131.22, 130.46, 128.70, 128.51, 126.70, 124.67, 122.22, 115.54, 105.29, 103.24, 57.00, 56.68, 53.80, 47.39, 35.64, 22.96, 19.10. HRMS (m/z): [M+H]+ calcd for $C_{25}H_{28}N_4O_2S$, 449.1933; found, 449.2030. HPLC (0.025% ammonium hydroxide in water): t_R = 14.065 min (98.6% purity).

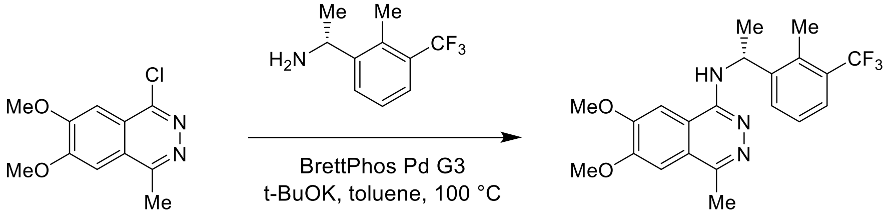

Compound 13

(*R*)-6,7-dimethoxy-4-methyl-*N*-(1-(2-methyl-3-(trifluoromethyl)phenyl)ethyl)phthalazin-1-amine (13)

A mixture of 1-chloro-6,7-dimethoxy-4-methylphthalazine (100 mg, 419 µmol, 1.00 *eq.*), (*R*)-1-(2-methyl-3-(trifluoromethyl)phenyl)ethan-1-amine (85.1 mg, 419 µmol, 1.00 *eq.*), BrettPhos Pd G3 (38.0 mg, 41.9 µmol, 0.10 *eq.*) and potassium *tert*-butoxide (1.00 M, 1.26 mL, 3.00 *eq.*) in toluene (2.00 mL) was degassed and purged with nitrogen 3 times. The reaction mixture was stirred at 100 °C for 1 hour under a nitrogen atmosphere, then cooled to 25 °C, filtered, and the filtrate was concentrated under reduced pressure to give a residue. The residue was purified by prep-HPLC (column: Waters Xbridge BEH C18 100 × 25 mm × 5 um; mobile phase: phase A: 10mM NH_4HCO_3 in water, phase B: ACN; B%: 35% - 65%) to give (*R*)-6,7-dimethoxy-4-methyl-*N*-(1-(2-methyl-3-(trifluoromethyl)phenyl)ethyl)phthalazin-1-amine, **Compound 13** (8.24 mg, 20.3 µmol, 4.84% yield, 97.7% purity) as a white solid.

^{1}H NMR (400 MHz, CD_3OD) δ = 8.49 (s, 1H), 7.94 (s, 1H), 7.71 (d, *J* = 8.0 Hz, 1H), 7.50 (d, *J* = 8.0 Hz, 1H), 7.41 (s, 1H), 7.25 (t, *J* = 8.0 Hz, 1H), 5.62 (q, *J* = 6.8 Hz, 1H), 4.12 (s, 3H), 4.04 (s, 3H), 2.73 (s, 3H), 2.62 (s, 3H), 1.66 (d, *J* = 6.8 Hz, 3H). ^{13}C NMR (101 MHz, CD_3OD) δ = 168.29, 155.49, 153.88, 152.88, 147.81, 145.59, 134.04 (d, *J* = 1.6 Hz, 1C), 128.70 (q, *J* = 28.3 Hz, 1C), 128.04, 125.76, 124.89 (q, *J* = 274.7 Hz, 1C), 123.89 (q, *J* = 6.4 Hz, 1C), 122.88, 115.39, 105.11, 102.53, 55.97, 55.53, 20.28, 15.68, 13.36 (d, *J* = 2.4 Hz, 1C). HRMS (m/ z): [M + H]+ calcd for $C_{21}H_{22}F_3N_3O_2$, 406.1664; found, 406.1761. HPLC (0.025% ammonium hydroxide in water): t_R = 13.470 min (97.7% purity).

(*R*)-N^7,N^7,4-trimethyl-N^1-(1-(2-methyl-3-(trifluoromethyl)phenyl)ethyl)phthalazine-1,7-diamine (14)

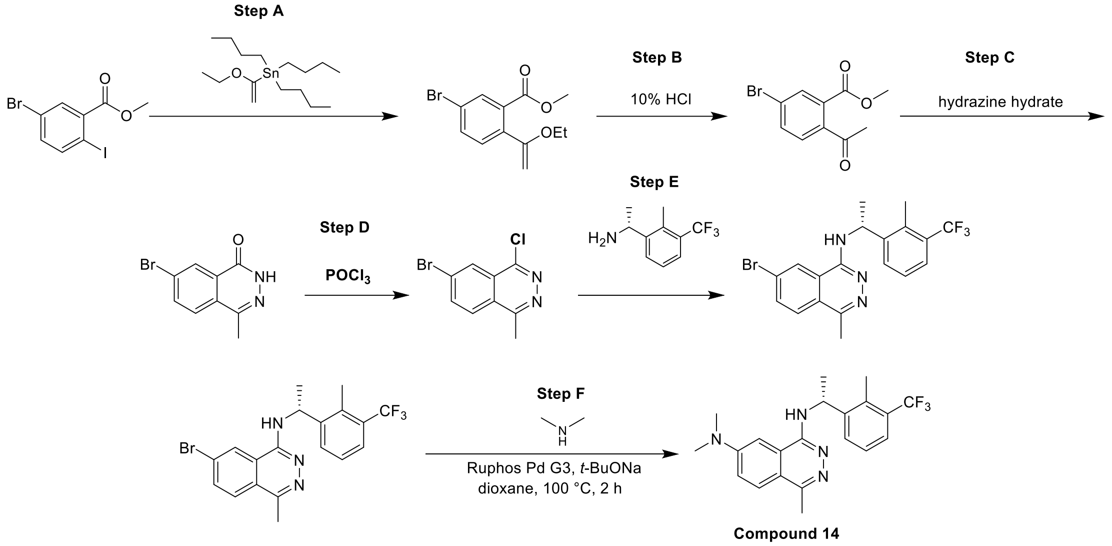

Step A: A mixture of methyl 5-bromo-2-iodobenzoate (5.00 g, 14.7 mmol, 1.00 *eq.*), tributyl(1-ethoxyvinyl)stannane (5.60 g, 15.4 mmol, 5.20 mL, 1.05 *eq.*) and $Pd(PPh_3)_2Cl_2$ (309 mg, 440 µmol, 0.03 *eq.*) in dioxane (50.0 mL) was degassed and purged with nitrogen for 3 times, and then the reaction mixture was stirred at 80 °C for 10 hours under a nitrogen atmosphere. The reaction mixture was cooled to 25 °C, quenched by addition water (50.0 mL**)**, and then extracted with ethyl acetate (50.0 mL × 3). The combined organic layers were washed with brine **(**20.0 mL × 3), dried over sodium sulfate, filtered, and concentrated under reduced pressure to give methyl 5-bromo-2-(1-ethoxyvinyl)benzoate (6.00 g, crude) as a yellow oil which was used in the next step directly.

Step B: To a solution of methyl 5-bromo-2-(1-ethoxyvinyl)benzoate (6.00 g, crude) in THF (50.0 mL) was added hydrochloric acid aqueous solution (10%, 25.0 mL). The reaction mixture was stirred at 20 °C for 1 hour. To the reaction mixture was added water (50.0 mL), and the aqueous layer was extracted with ethyl acetate (100 mL × 3). The combined organic layers were washed with brine (30.0 mL × 2), dried over sodium sulfate, filtered, and concentrated under reduced pressure to give a residue. The residue was purified by column chromatography (SiO_2, petroleum ether/ethyl acetate = 1/0 to 50/1) to give methyl 2-acetyl-5-bromobenzoate (2.50 g, 67.0% yield) as a yellow oil.

^{1}H NMR (400 MHz, $CDCl_3$) δ = 7.97 (d, *J* = 2.0 Hz, 1H), 7.70 (dd, *J* = 2.0, 8.2 Hz, 1H), 7.32 (d, *J* = 8.4 Hz, 1H), 3.91 (s, 3H), 2.53 (s, 3H).

Step C: To a solution of methyl 2-acetyl-5-bromobenzoate (1.50 g, 5.83 mmol, 1.00 *eq.*) in ethanol (30.0 mL) was added hydrazine hydrate (876 mg, 17.5 mmol, 851 µL, 3.00 *eq.*). The reaction mixture was stirred at 95 °C for 30 minutes. The reaction mixture was then cooled to 25 °C, and concentrated under reduced pressure to give a residue. The residue was triturated with ethanol for 10 minutes to give a suspension, the suspension was filtered, and the filter cake was collected and dried under vacuum to give 7-bromo-4-methylphthalazin-1(2*H*)-one (0.70 g, 2.93 mmol, 50.2% yield) as a white solid. LCMS $[M+1]^+$: 239.0.

^{1}H NMR (400 MHz, DMSO-d_6) δ = 12.57 (br s, 1H), 8.32 (d, *J* = 2.0 Hz, 1H), 8.11 (dd, *J* = 2.0, 8.4 Hz, 1H), 7.88 (d, *J* = 8.4 Hz, 1H), 2.50 (s, 3H).

Step D: A mixture of 7-bromo-4-methylphthalazin-1(2*H*)-one (4 g, 16.73 mmol, 1.00 *eq*) in $POCl_3$ (75.54 g, 492.66 mmol, 45.78 mL, 29.44 *eq*) was stirred at 20 °C under a N_2 atmosphere then heat to 100 °C and stirred for 5 hours. The mixture was cooled to 20 °C and concentrated under reduced pressure. The residue was slowly poured into water and neutralized with sat. $NaHCO_3$ until pH = 8. Then the ethyl acetate (100 mL) was added to the mixture and stirred for 30 minutes at 25 °C. The mixture was filtered and the filter cake was collected. The residue was purified using a silica gel column (0-55% petroleum ether /EtOAc) to give the 6-bromo-4-chloro-1-methylphthalazine (2.2 g, 8.54 mmol, 51.06% yield) as a yellow solid.

^{1}H NMR (500 MHz, DMSO-d_6) δ 8.41 (d, *J* = 1.5 Hz, 1H), 8.29-8.34 (m, 1H), 8.24-8.28 (m, 1H), 2.89-2.93 (m, 3H).

Step E: Note: the reaction needed to be set up in glove box under a N_2 atmosphere to avoid moisture. All of reagents including solvent (DMSO) need to be dried. To a mixture of 6-bromo-4-chloro-1-methylphthalazine (0.6 g, 2.32 mmol, 1.00 *eq*) and (*R*)-1-(2-methyl-3-(trifluoromethyl)phenyl)ethan-1-amine (472 mg, 2.32 mmol, 1.00 *eq*) in DMSO (4 mL) was added *N*, *N*-diisopropylethylamine (602 mg, 2.33 mmol, 810 µL, 2.00 *eq*) and CsF (706 mg, 4.66 mmol, 2.00 *eq*) in one portion at 20 °C under a N_2 atmosphere. The mixture was stirred at 130 °C for 3 hours. After this time the reaction was cooled to room temperature, water (50 mL) was added to the reaction mixture and extracted with ethyl acetate (3×30 mL). The combined organic layers were washed with brine (saturated, 20 mL), dried over Na_2SO_4, filtered, and concentrated to give a residue. The residue was purified using a preparative TLC plate eluting with 50% EtOAc / petroleum ether to give (*R*)-7-bromo-4-methyl-*N*-(1-(2-methyl-3-(trifluoromethyl)phenyl)ethyl)phthalazin-1-amine (0.6 g, 1414 µmol, 60.70% yield) as a yellow oil.

^{1}H NMR (400 MHz, $CDCl_3$) δ 7.97 (s, 1H), 7.87-7.93 (m, 1H), 7.79-7.85 (m, 1H), 7.66 (d, *J* = 7.6 Hz, 1H), 7.54 (d, *J* = 7.6 Hz, 1H), 7.26-7.29 (m, 1H), 5.88 (quint, *J* = 6.4 Hz, 1H), 5.12 (br s, 1H), 2.78 (s, 3H), 2.57 (s, 3H), 1.66 (d, *J* = 6.8 Hz, 3H).

Step F: A mixture of (*R*)-7-bromo-4-methyl-*N*-(1-(2-methyl-3-(trifluoromethyl)phenyl)ethyl)phthalazin-1-amine (15.0 mg, 35.4 µmol, 1.00 *eq*.), dimethylamine (2.00 M in THF, 1.50 mL, 84.9 *eq*.), RuPhos Pd G_3 (2.96 mg, 3.54 µmol, 0.10 *eq*.) and sodium *tert*-butoxide (10.2 mg, 106 µmol, 3.00 *eq*.) in THF (1.00 mL) was degassed and purged with nitrogen for 3 times, and then the mixture was stirred at 100 °C for 2 hrs under a nitrogen atmosphere. The reaction mixture was concentrated under reduced pressure to remove dioxane to give residue. The residue was diluted with water (50.0 mL) and extracted with ethyl acetate (30.0 mL × 3). The combined organic layers were washed with brine (50.0 mL × 3), dried over sodium sulfate, filtered, and concentrated under reduced pressure to give a residue. The residue was purified by prep-HPLC (column: Phenomenex Luna C18 75 × 30 mm × 3 µm; mobile phase: phase A: 0.05% HCl in water, phase B: acetonitrile; B%: 32%-52%) to give (*R*)-N^7,N^7,4-trimethyl-N^1-(1-(2-methyl-3-(trifluoromethyl)phenyl)ethyl)phthalazine-1,7-diamine (7.67 mg, 19.8 µmol, 55.9% yield, hydrochloride) as a yellow solid.

Characterization for the formic acid salt: ^{1}H NMR (400 MHz, DMSO-d_6) δ = 8.27 (s, 1H), 7.81 - 7.71 (m, 2H), 7.50 (d, *J* = 7.6 Hz, 1H), 7.39 (br s, 1H), 7.36 - 7.32 (m, 2H), 7.32 - 7.26 (m, 1H), 5.66 (br s, 1H), 3.13 (s, 6H), 2.57 (s, 3H), 2.51 (br s, 3H), 1.54 (d, *J* = 6.8 Hz, 3H). ^{13}C NMR (101 MHz, DMSO-d_6) δ ppm 164.72, 152.41, 152.07, 147.88, 147.81, 133.94, 129.16, 127.92 (q, *J* = 27.9 Hz, 1C), 126.67, 126.52, 125.35 (q, *J* = 273.9 Hz, 1C), 124.09 (q, *J* = 5.96 Hz, 1C), 120.29, 118.54, 117.93, 100.68, 46.75, 22.22, 18.87, 14.57 (s, 1C). HRMS (m/ z): [M + H]+ calcd for $C_{21}H_{23}F_3N_4$, 389.1875; found, 389.1973. HPLC (A: 0.0375% TFA in water, B: 0.01875% TFA in Acetonitrile): t_R = 4.776 min (96.6% purity).

(*R*)-4-methyl-*N*-(1-(2-methyl-3-(trifluoromethyl)phenyl)ethyl)-7-(piperazin-1-yl)phthalazin-1-amine (15)

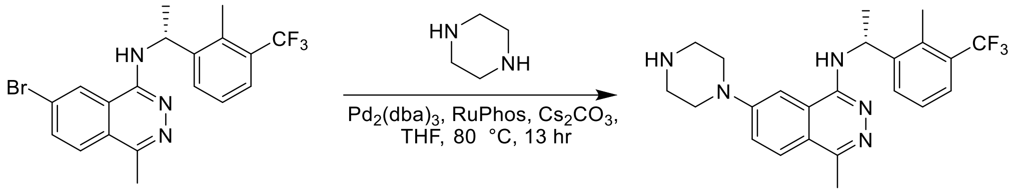

Compound 15

To a mixture of (*R*)-7-bromo-4-methyl-*N*-(1-(2-methyl-3-(trifluoromethyl)phenyl)ethyl)phthalazin-1-amine (200 mg, 471 μmol, 1.00 *eq.*) and piperazine (162 mg, 1.89 mmol, 4.00 *eq.*) in tetrahydrofuran (10.0 mL) was added $Pd_2(dba)_3$, cesium carbonate (461 mg, 1.41 mmol, 3.00 *eq.*) and RuPhos (36.6 mg, 47.1 μmol, 0.10 *eq.*) in one portion at 20 °C in a glovebox. The mixture was stirred at 80 °C for 13 hours. The suspension was filtered through a pad of celite, and the filter cake was washed with ethyl acetate (30.0 mL). The combined filtrates were concentrated, and the residue was purified by prep-HPLC (column: Agela DuraShell C18 150 × 25 mm × 5 μm, mobile phase A: 0.04% ammonium hydroxide in water + 10 mM ammonium carbonate in water, phase B: acetonitrile; Gradient: 35%-
65% B) to give (*R*)-4-methyl-*N*-(1-(2-methyl-3-(trifluoromethyl)phenyl)ethyl)-7-(piperazin-1-yl)phthalazin-1-amine (120 mg, 279 μmol, 59.3% yield) as a white solid.

^{1}H NMR (400 MHz, CD_3OD) δ = 7.86 (d, *J* = 8.8 Hz, 1H), 7.70 (d, *J* = 8.0 Hz, 1H), 7.59 - 7.53 (m, 2H), 7.47 (d, *J* = 7.6 Hz, 1H), 7.22 (t, *J* = 7.6 Hz, 1H), 5.71 (q, *J* = 6.8 Hz, 1H), 3.51 - 3.41 (m, 4H), 3.07 - 2.99 (m, 4H), 2.61 (s, 3H), 2.59 (s, 3H), 1.62 (d, *J* = 7.2 Hz, 3H). ^{13}C NMR (101 MHz, DMSO-d_6): δ ppm 152.96, 151.72, 147.46, 147.38, 133.54, 128.72, , 127.50 (q, *J* = 27.7 Hz, 1C), 126.08, 125.94, 124.92 (q, *J* = 274.9 Hz, 1C), 123.66 (q, *J* = 5.86 Hz, 1C), 120.25, 119.48,

119.13, 103.11, 56.08, 48.46, 46.25, 45.48, 21.78, 18.69, 18.58, 14.13(d, J = 2.2 Hz, 1C). [M + H]+ calcd for $C_{23}H_{26}F_3N_5$, 430.214; found, 430.2221. HPLC (0.025% ammonium hydroxide in water): t_R = 13.104 min (95.9% purity).

(*R*)-4-methyl-*N*-(1-(2-methyl-3-(trifluoromethyl)phenyl)ethyl)-7-(piperidin-4-yl)phthalazin-1-amine (16)

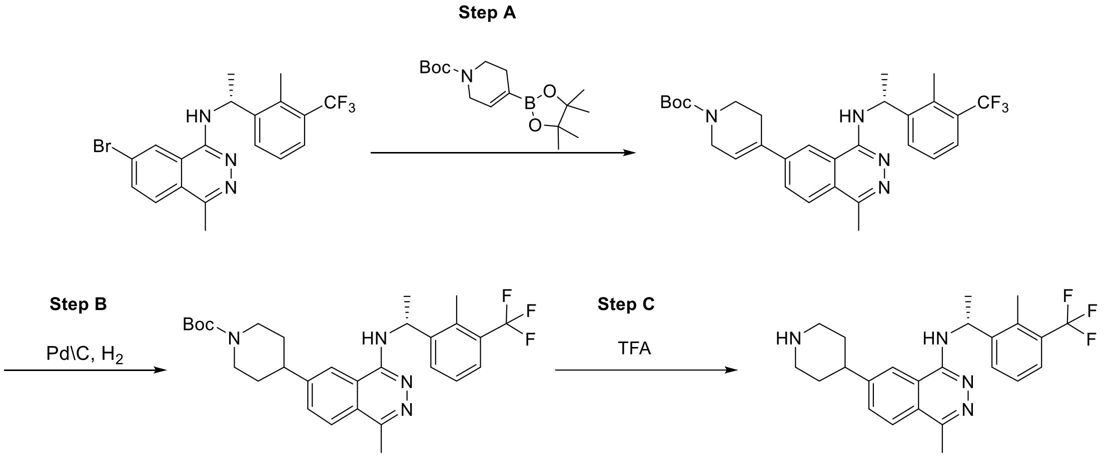

Compound 16

Step A: To a mixture of (*R*)-7-bromo-4-methyl-*N*-(1-(2-methyl-3-(trifluoromethyl)phenyl)ethyl)phthalazin-1-amine (35.0 mg, 82.5 µmol, 1.00 *eq.*) and *tert*-butyl 4-(4,4,5,5-tetramethyl-1,3,2-dioxaborolan-2-yl)-3,6-dihydropyridine-1(2*H*)-carboxylate (38.3 mg, 124 µmol, 1.50 *eq.*) in tetrahydrofuran (3.00 mL) and water (0.60 mL) was added sodium carbonate (26.2 mg, 247 µmol, 3.00 *eq.*) and Pd(dppf)Cl_2 (6.04 mg, 8.25 µmol, 0.10 *eq.*) in one portion at 20 °C under a nitrogen atmosphere. The mixture was stirred at 80 °C for 2 hours then cooled to the room temperature, and the mixture was diluted with ethyl acetate (30.0 mL), washed with water (10.0 mL × 3). The combined organic layers were washed with brine (20.0 mL), dried over sodium sulfate and filtered. The filtrate was concentrated to give the crude product as yellow oil. The yellow oil was purified by prep-TLC (SiO_2, petroleum ether / ethyl acetate = 2/1) to give *tert*-butyl (*R*)-4-(1-methyl-4-((1-(2-methyl-3-(trifluoromethyl)phenyl)ethyl)amino)phthalazin-6-yl)-3,6-dihydropyridine-1(2*H*)-carboxylate (32.0 mg, 60.8 µmol, 73.7% yield) as a yellow oil. LCMS $[M+1]^+$: 527.3.

Step B: To a solution of *tert*-butyl (*R*)-4-(1-methyl-4-((1-(2-methyl-3-(trifluoromethyl)phenyl)ethyl)amino)phthalazin-6-yl)-3,6-dihydropyridine-1(2*H*)-carboxylate

(14.0 mg, 26.6 µmol, 1.00 *eq.*) in methanol (3.00 mL) was added Pd/C (3.62 mg, 3.41 µmol, 10% purity, 0.13 *eq.*) under a nitrogen atmosphere. The suspension was degassed under vacuum and purged with hydrogen several times. The mixture was stirred under hydrogen (15.0 psi) at 25 °C for 2 hours then filtered through a pad of celite and the filter cake was washed with ethyl acetate (30.0 mL). The combined filtrates were concentrated to give *tert*-butyl (*R*)-4-(1-methyl-4-((1-(2-methyl-3-(trifluoromethyl)phenyl)ethyl)amino)phthalazin-6-yl)piperidine-1-carboxylate (14.0 mg, 26.5 µmol, 99.6% yield) as a yellow oil. The crude product was used directly in next step without further purification. LCMS $[M+1]^+$: 529.3.

Step C: A mixture of *tert*-butyl (*R*)-4-(1-methyl-4-((1-(2-methyl-3-(trifluoromethyl)phenyl)ethyl)amino)phthalazin-6-yl)piperidine-1-carboxylate (14 mg, 26.5 µmol, 1.00 *eq.*) in dichloromethane (2.00 mL) and trifluoroacetic acid (0.40 mL) was stirred at 20 °C for 2 hours then concentrated to give a residue. The residue was purified by prep-HPLC (column: Phenomenex Synergi C18 150 × 30 mm × 4 um using TFA water and acetonitrile as the eluents. mobile phase A: water (0.1%TFA), mobile phase B: acetonitrile. Gradient: 20%-50% B) to give (*R*)-4-methyl-*N*-(1-(2-methyl-3-(trifluoromethyl)phenyl)ethyl)-7-(piperidin-4-yl)phthalazin-1-amine (9.00 mg, 21.0 µmol, 79.3% yield) as a yellow solid.

^{1}H NMR (400 MHz, CD_3OD): ^{1}H NMR (400 MHz,CD_3OD) δ = 8.25 (s, 1H), 7.94 (d, *J* = 8.4 Hz, 1H), 7.79 (dd, *J* = 1.2, 8.4 Hz, 1H), 7.71 (d, *J* = 8.0 Hz, 1H), 7.46 (d, *J* = 8.0 Hz, 1H), 7.21 (t, *J* = 8.0 Hz, 1H), 5.73 (q, *J* = 6.8 Hz, 1H), 3.27 (br d, *J* = 12.4 Hz, 2H), 2.98 (tt, *J* = 3.2, 12.0 Hz, 1H), 2.92 - 2.81 (m, 2H), 2.64 (s, 3H), 2.61 (s, 3H), 2.02 - 1.94 (m, 2H), 1.86 (q, *J* = 12.4 Hz, 2H), 1.62 (d, *J* = 7.2 Hz, 3H). ^{13}C NMR (101 MHz,CD_3OD): δ ppm 154.35, 151.81, 150.20, 147.76, 135.68, 132.28, 130.20 (q, *J* = 29.3 Hz, 1C), 129.62, 127.23, 127.07, 126.60, 126.51 (q, *J* = 273.7 Hz, 1C), 125.16 (q, *J* = 6.1 Hz, 1C), 121.02, 120.38, 48.33, 47.11, 44.05, 33.95, 33.87, 21.87, 18.79, 14.95 (d, *J* = 2.5 HRMS (m/ z): [M + H]+ calcd for $C_{24}H_{27}F_3N_4$, 429.2188; found, 429.2273. HPLC (A: 0.0375% TFA in water, B: 0.01875% TFA in Acetonitrile): t_R = 3.420 min (99.0% purity).

(*R*)-1-methyl-4-(1-methyl-4-((1-(2-methyl-3-(trifluoromethyl)phenyl)ethyl)amino)phthalazin-6-yl)piperazin-2-one (17)

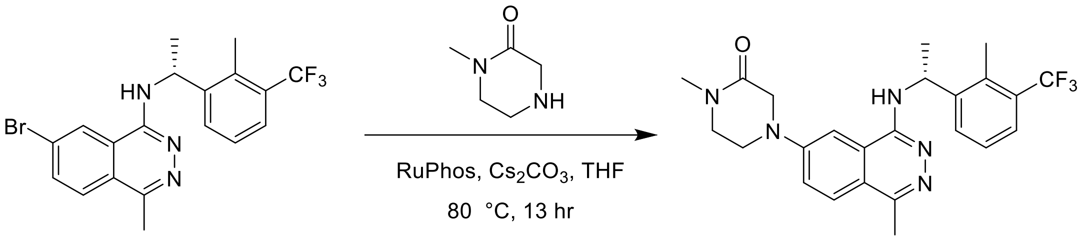

Compound 17

To a mixture of (*R*)-7-bromo-4-methyl-*N*-(1-(2-methyl-3-(trifluoromethyl)phenyl)ethyl)phthalazin-1-amine (0.03 g, 70.7 µmol, 1.00 *eq*) and 1-methylpiperazin-2-one (16.1 mg, 141 µmol, 2.00 *eq*) in THF (2.00 mL) was added cesium carbonate (69.1 mg, 212 µmol, 3.00 *eq*) and RuPhos (5.49 mg, 7.07 µmol, 0.10 *eq*) in one portion at 20 °C under a nitrogen atmosphere. The mixture was stirred at 80 °C for 13 hours under a nitrogen atmosphere. The reaction was cooled then to 25 °C, filtered through a pad of celite, and the filter cake was washed with ethyl acetate (20.0 mL). The combined filtrates were concentrated under reduced pressure to give a residue. The residue was purified by prep-HPLC (column: Phenomenex Synergi C18 100×21.2mm×4µm using, mobile phase A: 0.1%TFA in water, mobile phase B: acetonitrile; Gradient: 25%-55% B) to give (*R*)-1-methyl-4-(1-methyl-4-((1-(2-methyl-3-(trifluoromethyl)phenyl)ethyl)amino)phthalazin-6-yl)piperazin-2-one (0.02 g, 45.9 µmol, 64.9% yield) as a yellow solid.

^{1}H NMR (400 MHz, CD_3OD): δ ppm 7.75 (d, *J* = 9.2 Hz, 1H), 7.70 (d, *J* = 8.0 Hz, 1H), 7.47 - 7.42 (m, 2H), 7.38 (dd, *J* = 2.4, 9.2 Hz, 1H), 7.19 (t, *J* = 7.6 Hz, 1H), 5.69 (q, *J* = 7.2 Hz, 1H), 4.04 (s, 2H), 3.74 - 3.66 (m, 2H), 3.57 - 3.49 (m, 2H), 3.03 (s, 3H), 2.60 (s, 3H), 2.52 (s, 3H), 1.60 (d, *J* = 7.2 Hz, 3H). ^{13}C NMR (101 MHz, DMSO-d_6): δ ppm 165.19, 151.84, 150.29, 147.42, 147.30, 133.49, 128.60, 127.48 (q, *J* = 27.7 Hz, 1C), 126.12, 126.08, 124.87 (q, *J* =274.9 Hz, 1C), 123.62 (q, *J* = 5.05 Hz), 119.51, 119.38, 119.19, 102.39, 51.25, 47.39, 46.23, 43.73, 33.43, 21.77, 18.67, 14.11. HRMS (m/z): [M + H]+ calcd for $C_{24}H_{27}F_3N_5O$, 458.2089; found, 458.2184. HPLC (A: 0.0375% TFA in water, B: 0.01875% TFA in Acetonitrile): t_R = 4.356 min (99.4% purity).

(*R*)-*N*-(1-(2-methyl-3-(trifluoromethyl)phenyl)ethyl)-7-(piperazin-1-yl)-4-(trifluoromethyl)phthalazin-1-amine (18)

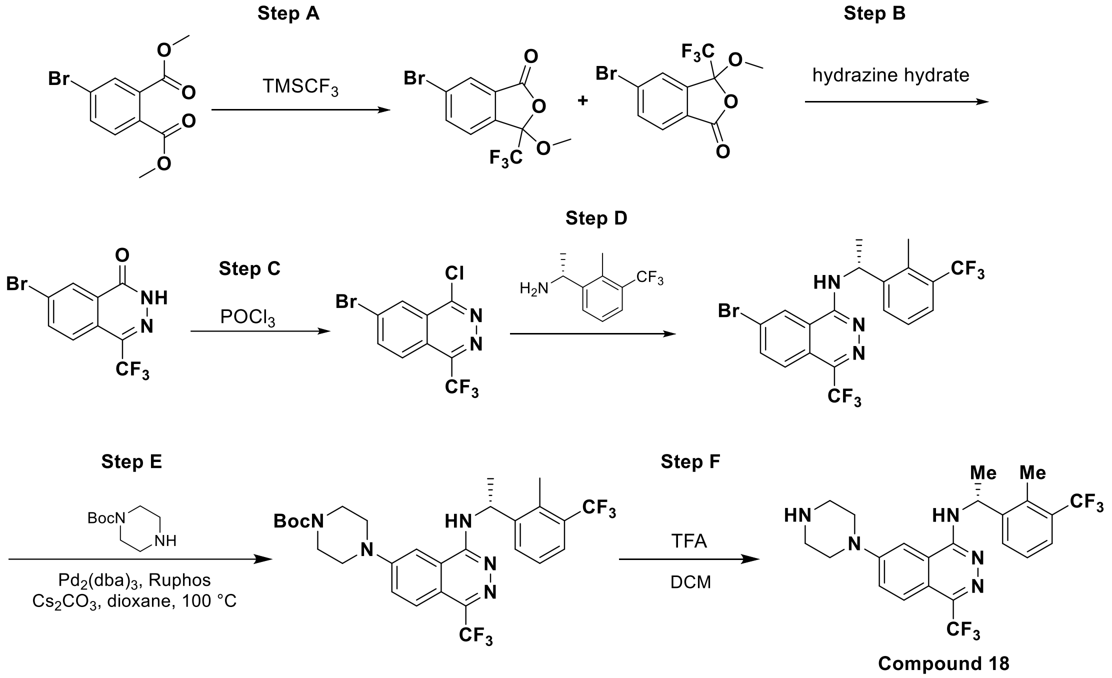

Step A: To a solution of dimethyl 4-bromophthalate (2.00 g, 7.32 mmol, 1.00 *eq.*) in 1,2-dimethoxyethane (25.0 mL) was added CsF (223 mg, 1.46 mmol, 54.0 µL, 0.20 *eq.*) and $TMSCF_3$ (1.25 g, 8.79 mmol, 1.20 *eq.*). The mixture was stirred between 0-25 °C for 1 hour. The mixture was then partitioned between ethyl acetate (1.00 mL) and water (15.0 mL). The organic phases were separated, washed with brine (15.0 mL × 3), dried over anhydrous sodium sulfate, filtered, and concentrated under reduced pressure to give a mixture of 6-bromo-3-methoxy-3-(trifluoromethyl)isobenzofuran-1(3*H*)-one and 5-bromo-3-methoxy-3-(trifluoromethyl)isobenzofuran-1(3*H*)-one (2.20 g, crude) as a colorless oil.

Step B: To a solution of 6-bromo-3-methoxy-3-(trifluoromethyl)isobenzofuran-1(3*H*)-one and 5-bromo-3-methoxy-3-(trifluoromethyl)isobenzofuran-1(3*H*)-one (2.20 g, 7.07 mmol, 1.00 *eq.*) in THF (25.0 mL) was added hydrazine hydrate (708 mg, 14.6 mmol, 688 µL, 2.00 *eq.*). The mixture was stirred at 75 °C for 18 hours then concentrated under reduced pressure to remove solvent. The residue was purified by flash silica gel chromatography (SiO_2, Petroleum ether / Ethyl acetate = 10/1 to 8/1) to give 7-bromo-4-(trifluoromethyl)phthalazin-1(2*H*)-one (680 mg, 2.32 mmol, 32.8% yield) as a white solid.

^1H NMR (400 MHz, CD_3OD) δ = 8.54 (d, *J* = 1.71 Hz, 1H), 8.15 (dd, *J* = 8.68, 2.08 Hz, 1H), 7.91 (dd, *J* = 8.80, 1.47 Hz, 1H).

Step C: To a solution of 7-bromo-4-(trifluoromethyl)phthalazin-1(2*H*)-one (200 mg, 683 µmol, 1.00 *eq.*) in $POCl_3$ (3.30 g, 21.5 mmol, 2.00 mL, 31.5 *eq.*) was added pyridine (108 mg, 1.37 mmol, 110 µL, 2.00 *eq.*) at 20 °C. The mixture was stirred at 105 °C for 1.5 hours then concentrated under reduced pressure to give 6-bromo-4-chloro-1-(trifluoromethyl)phthalazine (210 mg, crude) as a white solid.

Step D: To a solution of 6-bromo-4-chloro-1-(trifluoromethyl)phthalazine (250 mg, 802 µmol, 1.00 *eq.*), (*R*)-1-(2-methyl-3-(trifluoromethyl)phenyl)ethan-1-amine (326 mg, 1.61 mmol, 2.00 *eq.*) in DMSO (2.00 mL) was added cesium fluoride (243 mg, 1.61 mmol, 59.2 µL, 2.00 *eq.*), and the mixture was stirred at 130 °C for 2 hours. The reaction mixture was then cooled to 25 °C, diluted with ethyl acetate (10.0 mL). The organic phase was washed with brine (10.0 mL × 3), dried over anhydrous sodium sulfate, filtered, and concentrated under reduced pressure to give a residue. The residue was purified by column chromatography (SiO_2, petroleum ether/ethyl acetate =1/0 to 1/1) to give (*R*)-7-bromo-*N*-(1-(2-methyl-3-(trifluoromethyl)phenyl)ethyl)-4-(trifluoromethyl)phthalazin-1-amine (300 mg, 627 µmol, 78.2% yield) as a yellow solid. LCMS $[M+1]^+$: 478.0.

Step E: To a solution of (*R*)-7-bromo-*N*-(1-(2-methyl-3-(trifluoromethyl)phenyl)ethyl)-4-(trifluoromethyl)phthalazin-1-amine (280 mg, 585 µmol, 1.00 *eq.*), tert-butyl piperazine-1-carboxylate hydrochloride salt (260 mg, 1.17 mmol, 2.00 *eq.*) in dioxane (1.00 mL) was added $Pd_2(dba)_3$ (53.6 mg, 58.5 µmol, 0.10 *eq.*), RuPhos (27.3 mg, 58.6 µmol, 0.10 *eq.*), and cesium carbonate (572 mg, 1.76 mmol, 3.00 *eq.*) under a nitrogen atmosphere. The mixture was then stirred at 100 °C for 2 hours under a nitrogen atmosphere, then cooled to 25 °C, filtered, and concentrated under reduced pressure to give a residue. The residue was purified by column chromatography (SiO_2, petroleum ether/ethyl acetate =1/0 to 1/1) to give *tert*-butyl (*R*)-4-(4-((1-(2-methyl-3-(trifluoromethyl)phenyl)ethyl)amino)-1-(trifluoromethyl)phthalazin-6-yl)piperazine-1-carboxylate (250 mg, 517 µmol, 88.3% yield) as a yellow solid. LCMS $[M+1]^+$: 584.3.

Step F: To a solution of *tert*-butyl (*R*)-4-(4-((1-(2-methyl-3-(trifluoromethyl)phenyl)ethyl)amino)-1-(trifluoromethyl)phthalazin-6-yl)piperazine-1-carboxylate (280 mg, 579 µmol, 1.00 *eq*) in dichloromethane (1.00 mL) was added TFA (1.00 mL). The reaction mixture was stirred at 25

°C for 1 hour, then concentrated under reduced pressure to give a residue. The residue was purified by prep-HPLC (column: Phenomenex luna C18 150 × 25 mm × 10 um; mobile phase: [phase A: water (0.225% formic acid), phase B: acetonitrile; B%: 19% - 49%) to give (*R*)-*N*-(1-(2-methyl-3-(trifluoromethyl)phenyl)ethyl)-7-(piperazin-1-yl)-4-(trifluoromethyl)phthalazin-1-amine (200 mg, 413 µmol, 71.4% yield, TFA salt) as a white solid.

^{1}H NMR (400 MHz, DMSO-d_6): δ = 8.25 (d, *J* = 6.8 Hz, 1H), 7.95 - 7.65 (m, 4H), 7.53 (d, *J* = 7.6 Hz, 1H), 7.33 (t, *J* = 7.6 Hz, 1H), 5.81 (quin, *J* = 6.8 Hz, 1H), 3.82 - 3.47 (m, 4H), 3.36 - 3.00 (m, 4H), 2.58 (s, 3H), 1.60 (d, *J* = 6.8 Hz, 3H). ^{13}C NMR (101 MHz, DMSO-d_6): δ = 158.96 (q, *J* = 31.5 Hz, 1C), 154.16, 152.80, 146.80, 138.39 (q, *J* = 31.2 Hz, 1C), 134.02, 129.22, 128.10 (d, *J* = 28.2 Hz, 1C), 126.74, 125.75 (d, *J* = 172.3 Hz, 1C), 124.56, 124.46 (q, *J* = 6.6 Hz, 1C), 123.03(q, *J* = 173.9 Hz, 1C), 122.46, 119.82, 115.99, 104.21, 47.34, 45.69, 43.51, 21.96, 14.58 (d, *J* = 2.5 Hz, 1C). HRMS (m/ z): [M + H]+ calcd for $C_{23}H_{23}F_6N_5$, 484.1858; found, 484.1952. HPLC (A: 0.0375% TFA in water, B: 0.01875% TFA in Acetonitrile): t_R = 4.234 min (96.4 % purity).

(*R*)-4-methoxy-*N*-(1-(2-methyl-3-(trifluoromethyl)phenyl)ethyl)-7-(piperazin-1-yl)phthalazin-1-amine (19)

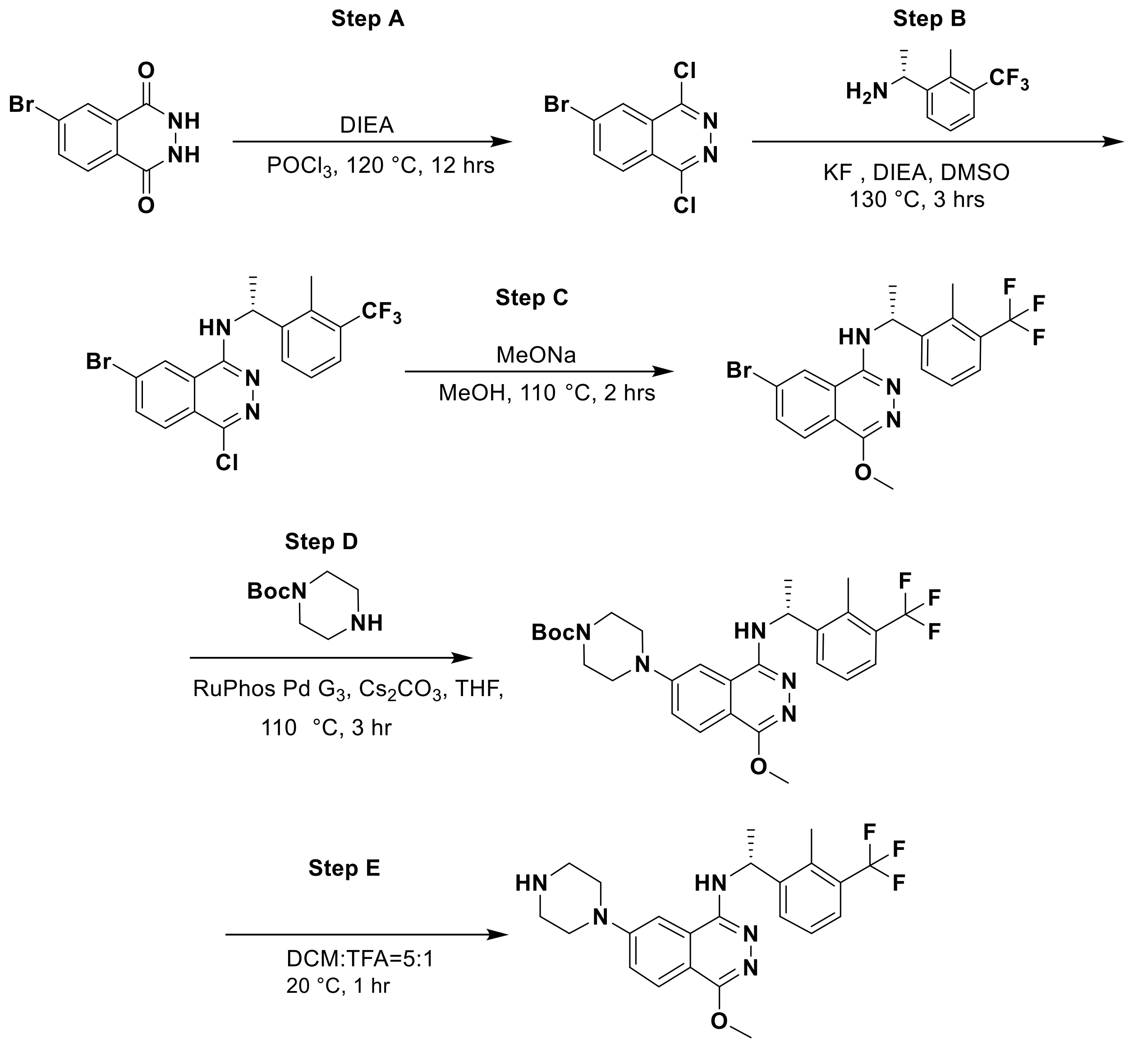

Compound 19

Step A: To a solution of 6-bromo-2,3-dihydrophthalazine-1,4-dione (3.00 g, 12.4 mmol, 1.00 *eq.*) in phosphorus oxychloride (40.0 mL) was added *N,N*-diisopropylethylamine (4.02 g, 31.1 mmol, 5.42 mL, 2.50 *eq.*) dropwise at 25 °C, then the reaction was stirred at 120 °C for 12 hours. The reaction was then cooled to 25 °C and concentrated under reduced pressure to give a residue. The

residue was poured into ice water (100 mL), and the resulting aqueous solution was adjusted to pH = 7 with saturated sodium bicarbonate aqueous solution, then extracted with DCM (50.0 mL × 2). The combined organic phases were washed with brine (30.0 mL × 2), dried over anhydrous sodium sulfate, filtered, and concentrated under reduced pressure to give 6-bromo-1,4-dichloro-phthalazine (1.20 g, 4.32 mmol, crude) as a yellow solid without further purification. LCMS $[M+3]^+$: 279.0.

Step B: To a mixture of 6-bromo-1,4-dichloro-phthalazine (500 mg, 1.80 mmol, 1.00 *eq.*) and (*R*)-1-(2-methyl-3-(trifluoromethyl)phenyl)ethan-1-amine (365 mg, 1.80 mmol, 1.00 *eq.*) in DMSO (10.0 mL) was added potassium fluoride (313 mg, 5.40 mmol, 126 µL, 3.00 *eq.*) and *N,N*-diisopropylethylamine (465 mg, 3.60 mmol, 627 µL, 2.00 *eq.*) under a nitrogen atmosphere. The reaction mixture was then stirred at 130 °C for 3 hours then cooled to 25 °C. The mixture was then diluted with ethyl acetate (20.0 mL), washed with brine (5.00 mL × 2), dried over anhydrous sodium sulfate, filtered, and concentrated under reduced pressure to give a residue. The residue was purified by prep-TLC (petroleum ether/ethyl acetate = 3/1 to give (*R*)-7-bromo-4-chloro-N-(1-(2-methyl-3-(trifluoromethyl)phenyl)ethyl)phthalazin-1-amine (360 mg, 769 µmol, 42.7% yield, white solid) as the second eluting isomer.

^{1}H NMR (400 MHz, $CDCl_3$) δ = 8.15 - 8.01 (m, 2H), 7.99 - 7.79 (m, 1H), 7.63 (d, J = 8.0 Hz, 1H), 7.56 - 7.50 (m, 1H), 7.23 (s, 1H), 5.91 - 5.77 (m, 1H), 5.45 (br d, J = 6.4 Hz, 1H), 2.55 (s, 3H), 1.65 (d, J = 6.8 Hz, 3H). LCMS $[M+3]^+$: 446.1.

Step C: To a mixture of (*R*)-7-bromo-4-chloro-N-(1-(2-methyl-3-(trifluoromethyl)phenyl)ethyl)phthalazin-1-amine (330 mg, 742 µmol, 1.00 *eq.*) in methanol (5.00 mL) was added sodium methoxide (200 mg, 3.71 mmol, 5.00 *eq.*) under a nitrogen atmosphere. The reaction mixture was stirred at 110 °C and stirred for 2 hours in the microwave. The mixture was then cooled to 25 °C and concentrated under reduced pressure to give a residue. The residue was purified by column chromatography (SiO_2, petroleum ether/ethyl acetate = 50/1 to 1/1) to give (*R*)-7-bromo-4-methoxy-*N*-(1-(2-methyl-3-(trifluoromethyl)phenyl)ethyl)phthalazin-1-amine (281 mg, 638 µmol, 86.0% yield) as a white solid.

Step D: To a solution of (*R*)-7-bromo-4-methoxy-*N*-(1-(2-methyl-3-

(trifluoromethyl)phenyl)ethyl)phthalazin-1-amine (240 mg, 545 μmol, 4.00 *eq.*) and *tert*-butyl piperazine-1-carboxylate (25.4 mg, 136 μmol, 1.00 *eq.*) in dioxane (5.00 mL) was added RuPhos Pd G3 (5.70 mg, 6.81 μmol, 0.05 *eq.*) and cesium carbonate (178 mg, 545 μmol, 4.00 *eq.*) in one portion at 20 °C under a nitrogen atmosphere. The mixture was stirred at 110 °C for 3 hours, then the suspension was filtered through a pad of celite, and the filter cake was washed with ethyl acetate (30.0 mL). The combined filtrates were concentrated to give a residue. The residue was purified by prep-HPLC (column: Phenomenex Synergi C18 150 × 30 mm × 4 μm, mobile phase A: water (0.1%TFA), phase B: acetonitrile; 49%-69% B) to give *tert*-butyl (*R*)-4-(1-methoxy-4-((1-(2-methyl-3-(trifluoromethyl)phenyl)ethyl)amino)phthalazin-6-yl)piperazine-1-carboxylate (70.0 mg, 128 μmol, 94.1% yield) as a white solid. LCMS $[M+1]^+$: 546.3.

Step E: To a mixture of *tert*-butyl (*R*)-4-(1-methoxy-4-((1-(2-methyl-3-(trifluoromethyl)phenyl)ethyl)amino)phthalazin-6-yl)piperazine-1-carboxylate (50.0 mg, 91.6 μmol, 1.00 *eq.*) in dichloromethane (1.00 mL) was added trifluoroacetic acid (0.20 mL). The mixture was stirred at 20 °C for 1 hour, then concentrated under reduced pressure to give a residue. The residue was purified by prep-HPLC (column: Phenomenex Synergi C18 100 × 21.2 mm × 4 μm, mobile phase A: water (0.1%TFA), mobile phase B: acetonitrile; 14%-44% B) to give (*R*)-4-methoxy-*N*-(1-(2-methyl-3-(trifluoromethyl)phenyl)ethyl)-7-(piperazin-1-yl)phthalazin-1-amine (35.0 mg, 78.6 μmol, 85.7% yield) as a white solid.

^{1}H NMR (400 MHz, CD_3OD): δ = 8.18 (d, *J* = 9.2 Hz, 1H), 8.08 (d, *J* = 2.0 Hz, 1H), 7.83 (dd, *J* = 2.8, 9.6 Hz, 1H), 7.75 (d, *J* = 7.6 Hz, 1H), 7.68 (d, *J* = 7.6 Hz, 1H), 7.41 (t, *J* = 8.0 Hz, 1H), 5.44 (q, *J* = 6.8 Hz, 1H), 4.08 (s, 3H), 3.86 - 3.79 (m, 4H), 3.46 - 3.39 (m, 4H), 2.52 (s, 3H), 1.81 (d, *J* = 6.8 Hz, 3H). ^{13}C NMR (101 MHz, DMSO-d_6): δ = 158.27 (q, *J* = 31.4 Hz, 1C), 155.25, 153.01, 148.43, 134.98, 128.68, 128.30 (q, *J* =27.7 Hz, 1C), 126.43, 125.41, 124.71 (d, *J* = 276.7 Hz, 1C), 124.99 (q, *J* = 2.93 Hz, 1C), 123.51, 122.56, 117.12 (q, *J* = 299.67 Hz), 113.08, 106.65, 54.75, 48.91, 44.12, 42.33, 20.68, 14.55. HRMS (m/ z): [M + H]+ calcd for $C_{23}H_{27}F_3N_5O$, 446.2089; found, 446.2164. HPLC (A: 0.0375% TFA in water, B: 0.01875% TFA in Acetonitrile): t_R = 3.482 min (95.7% purity).

(*R*)-N^1,N^1-dimethyl-N^4-(1-(2-methyl-3-(trifluoromethyl)phenyl)ethyl)-6-(piperazin-1-yl)phthalazine-1,4-diamine (20)

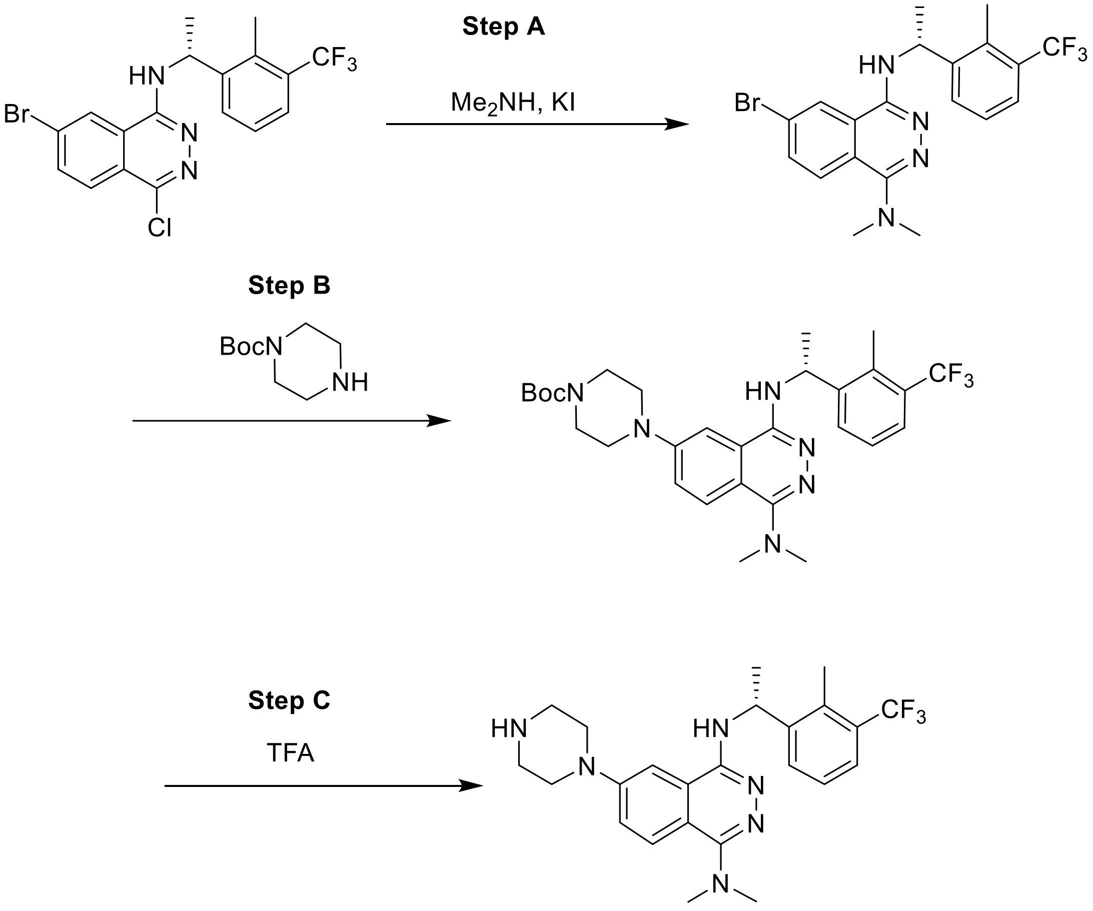

Compound 20

Step A: A mixture of (*R*)-7-bromo-4-chloro-*N*-(1-(2-methyl-3-(trifluoromethyl)phenyl)ethyl)phthalazin-1-amine (310 mg, 697 µmol, 1.00 *eq.*), dimethylamine (2.0 M in THF, 3.00 mL, 8.60 *eq.*), triethylamine (70.5 mg, 697 µmol, 97.0 µL, 1.00 *eq.*) and a catalytic amount of potassium iodide (57.9 mg, 349 µmol, 0.50 *eq.*) in *n*-butanol (3.00 mL) in a sealed pressure tube was heated at 110 °C for 14 hours. The reaction mixture was cooled to 25 °C and directly concentrated in vacuum to give a residue. The residue was purified by column chromatography (SiO_2, Petroleum ether/ethyl acetate=5/1 to 2/1) to give (*R*)-6-bromo-N^1,N^1-dimethyl-N^4-(1-(2-methyl-3-(trifluoromethyl)phenyl)ethyl)phthalazine-1,4-diamine (268 mg, 591 µmol, 84.8% yield) as a white solid.

^{1}H NMR (400 MHz,CD_3OD) δ = 8.65 (s, 1H), 8.13 - 7.90 (m, 2H), 7.69 (br d, *J* = 8.0 Hz, 1H), 7.48 (br d, *J* = 7.6 Hz, 1H), 7.23 (br t, *J* = 8.0 Hz, 1H), 5.64 (q, *J* = 6.8 Hz, 1H), 2.87 (s, 6H), 2.60 (s, 3H), 1.59 (d, *J* = 6.8 Hz, 3H)

Step B: To a mixture of (*R*)-6-bromo-N^1,N^1-dimethyl-N^4-(1-(2-methyl-3-(trifluoromethyl)phenyl)ethyl)phthalazine-1,4-diamine (40.0 mg, 88.2 μmol, 1.00 *eq.*) and *tert*-butyl piperazine-1-carboxylate (16.4 mg, 88.2 μmol, 1.00 *eq.*) in dioxane (2.00 mL) was added RuPhos Pd G3 (3.69 mg, 4.41 μmol, 0.05 *eq.*) and cesium carbonate (86.3 mg, 264 μmol, 3.00 *eq.*) in one portion at 20 °C under a nitrogen atmosphere. The mixture was stirred at 110 °C for 3 hours then cooled to 25 °C. The suspension was filtered through a pad of celite and the filter cake was washed with ethyl acetate (30.0 mL). The combined filtrates were concentrated under reduced pressure to give a residue. The residue was purified by prep-HPLC (column: Phenomenex Synergi C18 150 × 30 mm × 4 μm, mobile phase A: water (0.1%TFA), mobile phase B: acetonitrile; Gradient: 49%-69% B) to give *tert*-butyl (*R*)-4-(1-(dimethylamino)-4-((1-(2-methyl-3-(trifluoromethyl)phenyl)ethyl)amino)phthalazin-6-yl)piperazine-1-carboxylate (20.0 mg, 35.8 μmol, 40.6% yield) as a white solid. LCMS $[M+1]^+$: 559.4.

Step C: To a mixture of *tert*-butyl (*R*)-4-(1-(dimethylamino)-4-((1-(2-methyl-3-(trifluoromethyl)phenyl)ethyl)amino)phthalazin-6-yl)piperazine-1-carboxylate (20.0 mg, 35.8 μmol, 1.00 *eq.*) in trifluoroacetic acid (0.50 mL) and dichloromethane (2.50 mL) was stirred at 20 °C for 1 hour. The mixture was then concentrated under reduced pressure to give a residue. The residue was purified by prep-HPLC (column: Phenomenex Synergi C18 100 × 21.2 mm × 4 μm, mobile phase A: water (0.1%TFA), mobile phase B: acetonitrile; Gradient: 14%-44% B) to give (*R*)-N^1,N^1-dimethyl-N^4-(1-(2-methyl-3-(trifluoromethyl)phenyl)ethyl)-6-(piperazin-1-yl)phthalazine-1,4-diamine (10.0 mg, 21.8 μmol, 60.9% yield) as a white solid.

^{1}H NMR (400 MHz, DMSO-d_6) δ = 7.81 (d, *J* = 9.2 Hz, 1H), 7.77 (d, *J* = 8.0 Hz, 1H), 7.57 (s, 1H), 7.54 - 7.42 (m, 2H), 7.30 (t, *J* = 7.6 Hz, 1H), 7.21 (d, *J* = 6.8 Hz, 1H), 5.64 (quin, *J* = 6.8 Hz, 1H), 3.35 (s, 4H), 2.90 (br s, 4H), 2.76 (s, 6H), 2.56 (s, 3H), 1.52 (d, *J* = 6.8 Hz, 3H). ^{13}C NMR (101 MHz, DMSO-d_6) δ = 155.41, 153.10, 150.67, 148.02, 133.79, 129.13, 128.02 (q, *J* = 27.7 Hz), 126.55, 126.31, 125.35 (q, *J* = 274.9 Hz), 124.09 (q, *J* = 5.8 Hz), 122.33, 119.96, 115.08, 104.03 48.77, 46.81, 45.89, 43.53, 22.36, 14.56. HRMS (m/ z): $[M + H]^+$ calcd for $C_{24}H_{29}F_3N_6$,

459.2406; found, 459.2501. HPLC (A: 0.0375% TFA in water, B: 0.01875% TFA in Acetonitrile): t_R = 3.61 min (98.9% purity).

(*R*)-4-((1-(2-methyl-3-(trifluoromethyl)phenyl)ethyl)amino)-6-(piperazin-1-yl)phthalazin-1(2*H*)-one (21)

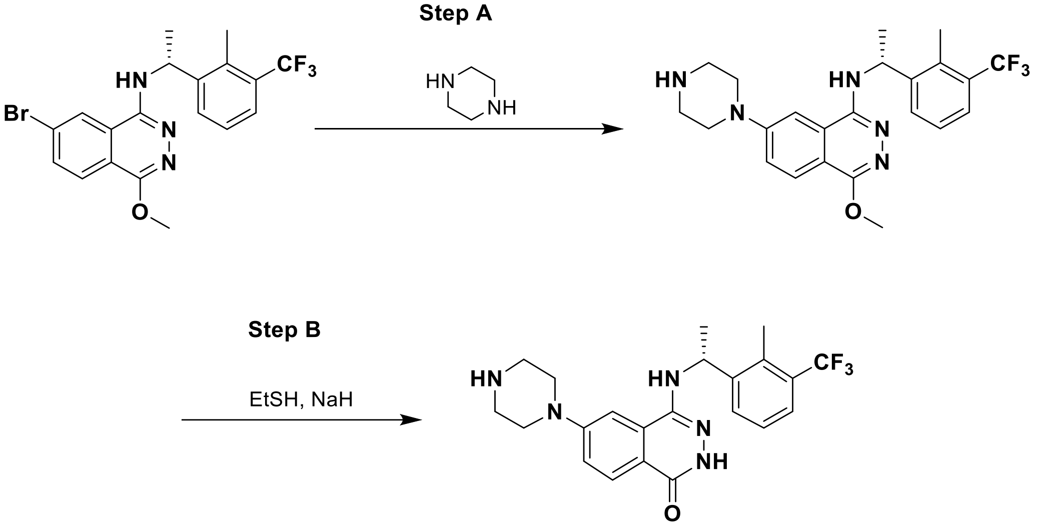

Compound 21

Step A: A mixture of (*R*)-7-bromo-4-methoxy-*N*-(1-(2-methyl-3-(trifluoromethyl)phenyl)ethyl)phthalazin-1-amine (80.0 mg, 182 µmol, 1.00 *eq.*), piperazine (62.6 mg, 727 µmol, 4.00 *eq.*), Cs_2CO_3 (178 mg, 545 µmol, 3.00 *eq.*), RuPhos (17.0 mg, 36.3 µmol, 0.20 *eq.*) and $Pd_2(dba)_3$ (16.6 mg, 18.2 µmol, 0.10 *eq.*) in dioxane (2.00 mL) was degassed and purged with nitrogen 3 times, and then the mixture was stirred at 100 °C for 1 hour under a nitrogen atmosphere. The reaction mixture was then cooled to 25 °C, filtered, and concentrated under reduced pressure to give a residue. The residue was purified by prep-TLC (SiO_2, DCM/MeOH = 10/1) to give (*R*)-4-methoxy-*N*-(1-(2-methyl-3-(trifluoromethyl)phenyl)ethyl)-7-(piperazin-1-yl)phthalazin-1-amine (70.0 mg, 157 µmol, 86.5% yield) as a yellow solid. LCMS $[M+1]^+$: 446.2.

Step B: To a solution of ethanethiol (123 mg, 1.98 mmol, 146 µL, 11.0 *eq.*) in DMF (3.00 mL) was added sodium hydride (71.8 mg, 1.80 mmol, 60% purity, 10.0 *eq.*) at 30 °C, and the mixture was stirred at this temperature for 15 minutes. After this time, (*R*)-4-methoxy-*N*-(1-(2-methyl-3-(trifluoromethyl)phenyl)ethyl)-7-(piperazin-1-yl)phthalazin-1-amine (80.0 mg, 180 µmol, 1.00 *eq.*) in DMF (1.00 mL) was added to the mixture, and the mixture was heated to 120 °C and stirred

for 1 hour. The reaction mixture was then cooled to 25 °C, diluted with ethyl acetate (20.0 mL) and washed with brine (10.0 mL × 2). The organic phases were dried over sodium sulfate, filtered, and concentrated under reduced pressure to give a residue. The residue was purified by prep-HPLC (column: Phenomenex luna C18 150 × 25mm × 10um; mobile phase: phase A: water (0.225% formic acid), phase B: acetonitrile; B%: 13%-43%) to give (*R*)-4-((1-(2-methyl-3-(trifluoromethyl)phenyl)ethyl)amino)-6-(piperazin-1-yl)phthalazin-1(2*H*)-one (52.0 mg, 109 µmol, 60.5% yield, 99.7% purity, formic acid salt) as a white solid.

^{1}H NMR (400 MHz, CD_3OD) δ = 8.51 (s, 1H), 8.14 (d, *J* = 9.2 Hz, 1H), 7.70 (d, *J* = 8.0 Hz, 1H), 7.56 (d, *J* = 2.0 Hz, 1H), 7.49 (d, *J* = 7.6 Hz, 1H), 7.46 - 7.41 (m, 1H), 7.25 (t, *J* = 8.0 Hz, 1H), 5.37 - 5.28 (m, 1H), 3.77 - 3.67 (m, 4H), 3.39 - 3.33 (m, 4H), 2.57 (s, 3H), 1.56 (d, *J* = 6.8 Hz, 3H). ^{13}C NMR (101 MHz, DMSO-d_6): δ ppm 165.75, 158.26, 154.16, 147.47, 143.69, 133.84, 129.08, 128.18, 127.99 (q, *J* = 28.2 Hz, 1C), 126.74, 125.32 (q, *J* = 274.7 Hz, 1C), 126.59, 124.20 (q, *J* = 5.9 Hz, 1C), 119.40, 118.77, 105.96, 46.92, 44.34, 22.25, 14.52. HRMS (m/ z): [M + H]+ calcd for $C_{22}H_{24}F_3N_5O$, 432.1944; found, 432.2023. HPLC (A: 0.0375% TFA in water, B: 0.01875% TFA in Acetonitrile): t_R = 4.012 min (97.9% purity).

(*R*)-2-methyl-4-((1-(2-methyl-3-(trifluoromethyl)phenyl)ethyl)amino)-6-(piperazin-1-yl)phthalazin-1(2*H*)-one (22)

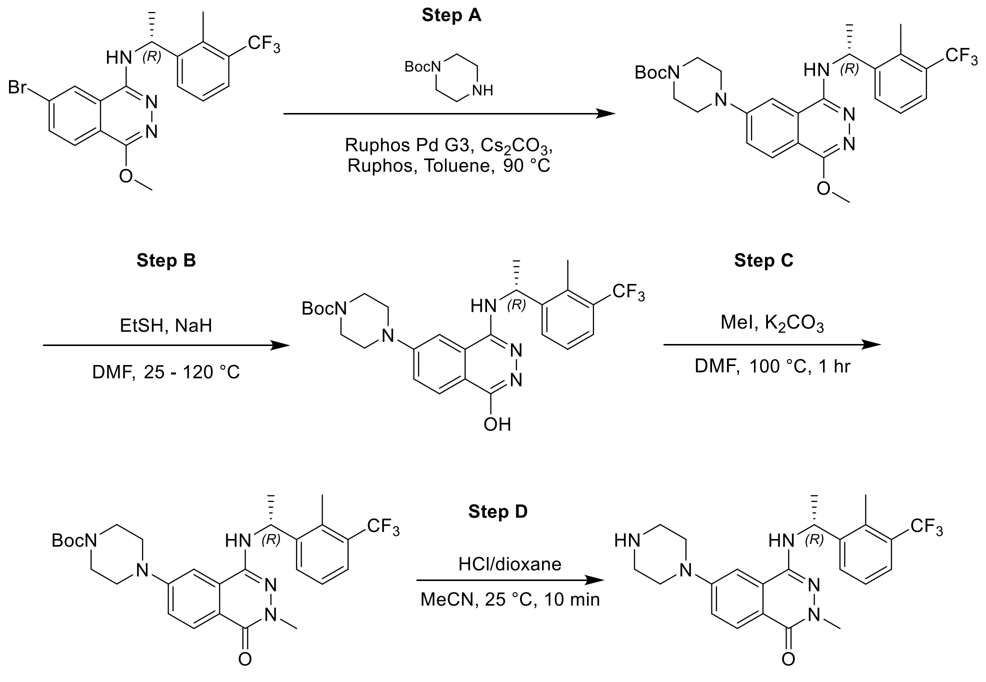

Compound 22

Step A: To a mixture of (*R*)-7-bromo-4-methoxy-*N*-(1-(2-methyl-3-(trifluoromethyl)phenyl)ethyl)phthalazin-1-amine (100 mg, 227 µmol, 1.00 *eq.*) and *tert*-butyl piperazine-1-carboxylate (254 mg, 1.36 mmol, 6 *eq.*) in toluene (3.00 mL) was added RuPhos (21.2 mg, 45.4 µmol, 0.20 *eq.*), RuPhos Pd G_3 (19.0 mg, 22.7 µmol, 0.1 *eq.*) and cesium carbonate (222 mg, 681 µmol, 3.00 *eq.*) in one portion under a nitrogen atmosphere. The mixture was stirred at 90 °C for 6 hours then cooled to 25 °C. Then mixture was then diluted with water (10.0 mL) and extracted with ethyl acetate (10.0 mL × 3). The combined organic layers were washed with brine (10.0 mL × 1), dried over sodium sulfate, filtered, and concentrated under reduced pressure to give a residue. The residue was purified by column chromatography (SiO_2, petroleum ether/ethyl acetate=20/1 to 0/1) to give *tert*-butyl (*R*)-4-(1-methoxy-4-((1-(2-methyl-3-(trifluoromethyl)phenyl)ethyl)amino)phthalazin-6-yl)piperazine-1-carboxylate (86.0 mg, 158 µmol, 69% yield) as a white solid. LCMS $[M+1]^+$: 546.7.

Step B: NaH (55.0 mg, 1.37 mmol, 60% purity, 10.0 *eq.*) dissolved in DMF (1.00 mL) was added to a suspension of ethanethiol (171 mg, 2.75 mmol, 203 µL, 20.0 *eq.*) under an atmosphere of nitrogen. The mixture was stirred for 15 minutes at 25 °C, then a solution of *tert*-butyl (*R*)-4-(1-methoxy-4-((1-(2-methyl-3-(trifluoromethyl)phenyl)ethyl)amino)phthalazin-6-yl)piperazine-1-carboxylate (75.0 mg, 137 µmol, 1.00 *eq.*) in dry DMF (1.00 mL) was added to the reaction mixture. The mixture was stirred at 120 °C for 3 hours then cooled to 25 °C. The mixture was then diluted with ethyl acetate (30.0 mL) washed with brine (10.0 mL × 3), dried over sodium sulfate, filtered, and concentrated under reduced pressure to give a residue. The residue was purified by column chromatography (SiO_2, petroleum ether/ethyl acetate=20/1 to 0/1) to give *tert*-butyl (*R*)-4-(1-hydroxy-4-((1-(2-methyl-3-(trifluoromethyl)phenyl)ethyl)amino)phthalazin-6-yl)piperazine-1-carboxylate (60.0 mg, 113 µmol, 82% yield) as a yellow solid. LCMS $[M+1]^+$: 532.2.

Step C: To a mixture of *tert*-butyl (*R*)-4-(1-hydroxy-4-((1-(2-methyl-3-(trifluoromethyl)phenyl)ethyl)amino)phthalazin-6-yl)piperazine-1-carboxylate (10.0 mg, 18.8 µmol, 1.00 *eq.*) and iodomethane (10.7 mg, 75.2 µmol, 4.68 µL, 4.00 *eq.*) in DMF (1.00 mL) was added potassium carbonate (13.0 mg, 94.1 µmol, 5.00 *eq.*) under a nitrogen atmosphere. The mixture was stirred at 100 °C for 1 hour, then cooled to room temperature. The reaction mixture was diluted with ethyl acetate (30.0 mL) and washed with brine (20.0 mL × 3), dried over sodium sulfate, filtered, and concentrated under reduced pressure to give a residue. The residue was

purified by prep-HPLC (column: Phenomenex Gemini-NX C18 75 × 30 mm × 3 µm; mobile phase A: water (0.05% ammonia hydroxide v/v), mobile phase B: acetonitrile; B%: 50% - 80%) to give *tert*-butyl (*R*)-4-(2-methyl-4-((1-(2-methyl-3-(trifluoromethyl)phenyl)ethyl)amino)-1-oxo-1,2-dihydrophthalazin-6-yl)piperazine-1-carboxylate (14.0 mg, 25.7 µmol, 45% yield) as a white solid. LCMS $[M+1]^+$: 546.3.

Step D: To a mixture of *tert*-butyl (*R*)-4-(2-methyl-4-((1-(2-methyl-3-(trifluoromethyl)phenyl)ethyl)amino)-1-oxo-1,2-dihydrophthalazin-6-yl)piperazine-1-carboxylate (10 mg, 18.3 µmol, 1 *eq.*) in acetonitrile (0.50 mL) was added HCl in dioxane (4 M, 0.50 mL, 109 *eq.*) under a nitrogen atmosphere. The mixture was stirred at 25 °C for 10 minutes then concentrated under reduced pressure to give a residue. The residue was purified by prep-HPLC (column: Phenomenex luna C 18 150 × 25 mm × 10 µm; mobile phase A: [water(0.05%HCl), mobile phase B: acetonitrile; B%: 21% - 51%) to give (*R*)-2-methyl-4-((1-(2-methyl-3-(trifluoromethyl)phenyl)ethyl)amino)-6-(piperazin-1-yl)phthalazin-1(2*H*)-one (4.21 mg, 8.72 µmol, 47% yield, hydrochloric acid salt) as a white solid.

Tabulated data for Free base of Compound 22: ^{1}H NMR (400 MHz, DMSO-d_6) δ = 7.96 (d, *J* = 9.2 Hz, 1H), 7.75 (d, *J* = 7.6 Hz, 1H), 7.50 (d, *J* = 7.2 Hz, 1H), 7.46 (d, *J* = 2.0 Hz, 1H), 7.36 (dd, *J* = 2.0, 9.2 Hz, 1H), 7.34 - 7.27 (m, 1H), 7.11 (br d, *J* = 6.8 Hz, 1H), 5.21 (quin, *J* = 6.8 Hz, 1H), 3.36 - 3.34 (m, 4H), 3.30 (s, 3H), 2.95 - 2.78 (m, 4H), 2.57 (s, 3H), 1.50 (d, *J* = 7.2 Hz, 3H). ^{13}C NMR (101 MHz, DMSO-d_6) δ = 157.08, 154.45, 147.90, 143.07, 133.90, 129.14, 128.32, 127.61 (q, *J* = 27.9 Hz), 126.62, 126.25, 125.35 (q, *J* = 275.0 Hz), 124.08 (q, *J* = 6.6 Hz), 118.60, 118.52, 105.29, 48.55, 46.67, 45.93, 38.04, 22.33, 14.52. HRMS (m/ z): $[M + H]^{+:}$ calcd for $C_{23}H_{26}F_3N_5O$, 446.2089; found, 446.2178. HPLC (A: 0.0375% TFA in water, B: 0.01875% TFA in Acetonitrile): t_R = 4.44 min (99.8% purity).

(*R*)-4-methyl-*N*-(1-(2-methyl-3-(trifluoromethyl)phenyl)ethyl)-7-(piperazin-1-yl)pyrido[3,4-*d*]pyridazin-1-amine (23)

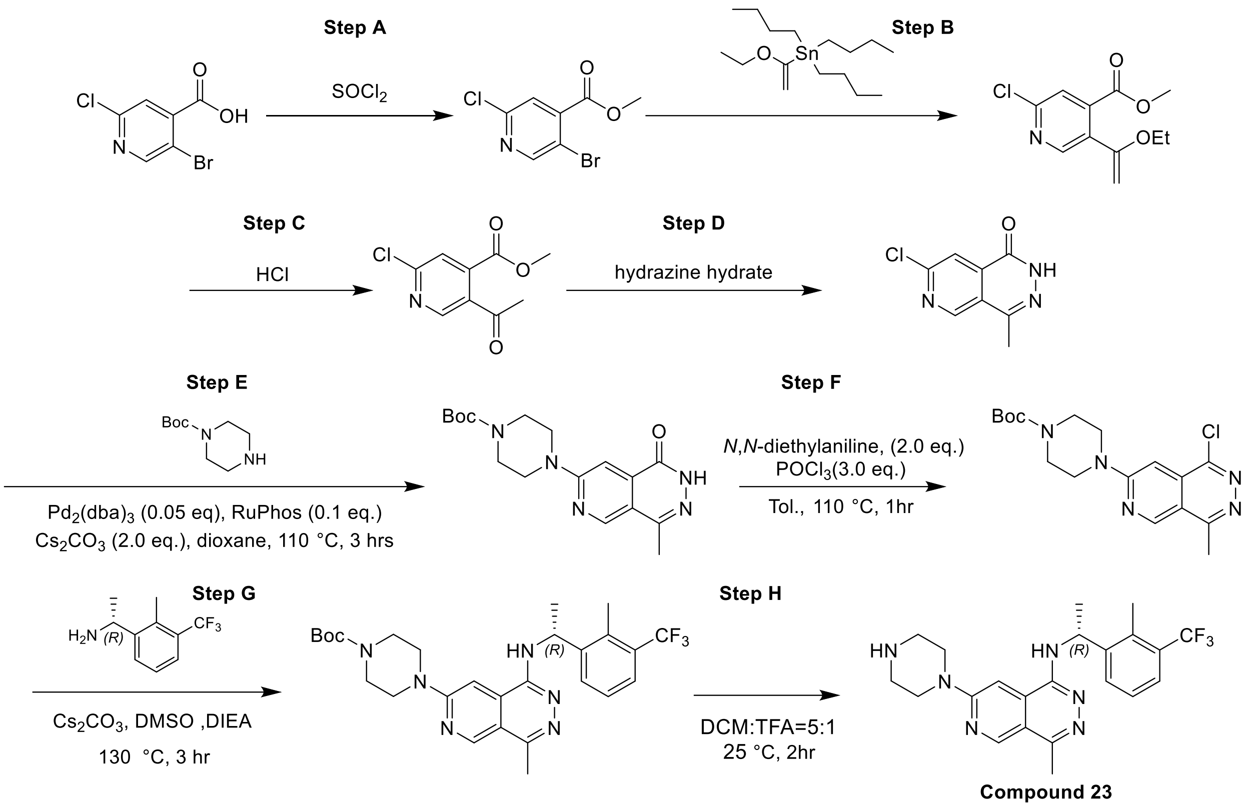

Step A: To a solution of 5-bromo-2-chloroisonicotinic acid (12.0 g, 50.6 mmol, 1.00 eq) in MeOH (100 mL) was added $SOCl_2$ (7.25 g, 60.9 mmol, 4.42 mL, 1.20 eq) dropwise, then the mixture was heated to 75 °C and stirred for 8 hours. The reaction mixture was then concentrated under reduced pressure to give a residue. The residue was diluted with EtOAc (100 mL), washed with saturated $NaHCO_3$ (100 mL), dried over Na_2SO_4, filtered, and concentrated under reduced pressure to give methyl 5-bromo-2-chloroisonicotinate (12.0 g, crude) as a yellow oil. LCMS $[M+1]^+$: 252.0.

^{1}H NMR (400 MHz, DMSO-d_6) δ =8.78 (s, 1H), 7.89 (s, 1H), 3.91 (s, 3H).

Step B: A solution of methyl 5-bromo-2-chloroisonicotinate (11.0 g, 43.92 mmol, 1.00 eq), tributyl(1-ethoxyvinyl)stannane (16.7 g, 46.1 mmol, 15.6 mL, 1.05 eq) and $Pd(PPh_3)_2Cl_2$ (1.23 g, 1.76 mmol, 0.04 eq) in dioxane (110 mL) was degassed and purged with N_2 3 times, and then the mixture was heated at 80 °C for 16 hours under a N_2 atmosphere. The reaction mixture was then

quenched by addition water (400 mL), and then extracted with EtOAc (150 mL × 3). The combined organic layers were washed with brine (200 mL), dried over Na_2SO_4, filtered, and concentrated under reduced pressure to give methyl 2-chloro-5-(1-ethoxyvinyl)isonicotinate (10.6 g, crude) was obtained as a yellow oil.

Step C: To a solution of methyl 2-chloro-5-(1-ethoxyvinyl)isonicotinate (10.6 g, 43.9 mmol, 1.00 eq) in THF (100 mL) was added HCl (102 g, 280 mmol, 100 mL, 10% purity in water, 6.38 eq) dropwise, and the mixture was stirred at 20 °C for 16 hours. The reaction mixture was then quenched by addition of $NaHCO_3$ (300 mL) at 0°C, and then extracted with EtOAc (100 mL × 3). The combined organic layers were washed with brine (100 mL), dried over Na_2SO_4, filtered, and concentrated under reduced pressure to give a residue. The residue was purified by column chromatography (SiO_2, Petroleum ether/Ethyl acetate=1/0 to 3/1) to give methyl 5-acetyl-2-chloroisonicotinate (4.50 g, 21.1 mmol, 48.0% yield) was obtained as a white solid.

^{1}H NMR (400 MHz, DMSO-d_6) δ = 8.98 (s, 1H), 7.85 (s, 1H), 3.84 (s, 3H), 2.61 (s, 3H).

Step D: To a solution of methyl 5-acetyl-2-chloroisonicotinate (1.00 g, 4.68 mmol, 1.00 eq) in EtOH (15.0 mL) was added hydrazine hydrate (703 mg, 14.0 mmol, 683 μL, 3.00 eq), the mixture was stirred at 95 °C for 30 minutes. The reaction mixture was then filtered and the filter cake was concentrated under reduced pressure to give a residue to give 7-chloro-4-methylpyrido[3,4-*d*]pyridazin-1(2*H*)-one (0.85 g, crude) was obtained as a white solid. LCMS $[M+1]^+$: 196.1.

^{1}H NMR (400 MHz, DMSO-d_6) δ = 9.20 (s, 1H), 8.10 (s, 1H), 2.58 (s, 3H).

Step E: To a solution of *tert*-butyl piperazine-1-carboxylate (761 mg, 4.09 mmol, 2.00 *eq*.) and 7-chloro-4-methylpyrido[3,4-*d*]pyridazin-1(2*H*)-one (400 mg, 2.04 mmol, 1.00 *eq*.) in dioxane (7.00 mL) was added t-BuOK (1.00 M in THF, 4.09 mL, 2.00 *eq*.) RuPhos (95.4 mg, 204 μmol, 0.10 *eq*.) and $Pd_2(dba)_3$ (93.6 mg, 102 μmol, 0.05 *eq*.) under a nitrogen atmosphere. The mixture was stirred at 110 °C for 3 hours then cooled and water (100 mL) was added to the mixture. The mixture was extracted with ethyl acetate (5 × 60.0 mL) and the combined organic layers were washed with brine (50.0 mL), dried over Na_2SO_4, filtered, and concentrated under reduced pressure to give a residue. The residue was purified by column chromatography (SiO_2, Petroleum ether/ Ethyl acetate

= 6/1 to 1/1) to give *tert*-butyl 4-(4-methyl-1-oxo-1,2-dihydropyrido[3,4-*d*]pyridazin-7-yl)piperazine-1-carboxylate (500 mg, 1.34 mmol, 65.5% yield) as a yellow solid. LCMS [M+1] $^+$: 346.2.

^{1}H NMR (400MHz, DMSO-d_6) δ = 12.23 (s, 1H), 8.88 (s, 1H), 7.23 (s, 1H), 3.71 - 3.69 (m, 4H), 3.47 - 3.45 (s, 4H), 2.49 - 2.45 (m, 3H), 1.43 (s, 9H).

Step F: To a solution of *tert*-butyl 4-(4-methyl-1-oxo-1,2-dihydropyrido[3,4-*d*]pyridazin-7-yl)piperazine-1-carboxylate (340 mg, 984 µmol, 1.00 *eq.*) and *N*,*N*-diethylaniline (293 mg, 1.97 mmol, 314 µL, 2.00 *eq.*) in toluene (3.00 mL) was added $POCl_3$ (452 mg, 2.95 mmol, 274 µL, 3.00 *eq.*) dropwise at 0°C under a nitrogen atmosphere. The reaction mixture was warmed to 110°C and stirred at for 1 hour. The reaction was then cooled and quenched by $NaHCO_3$ (aq., 80.0 mL) slowly and then extracted with dichloromethane (3 × 40 mL). The combined organic phases were washed with brine (40.0 mL), dried over anhydrous Na_2SO_4, filtered, and concentrated under reduced pressure. The residue was purified by prep-TLC (SiO_2, Petroleum ether/ Ethyl acetate = 0/1) to give *tert*-butyl 4-(1-chloro-4-methylpyrido[3,4-d]pyridazin-7-yl)piperazine-1-carboxylate (100 mg, 274 µmol, 27.9% yield) as a yellow solid. LCMS [M+1] $^+$: 364.2.

^{1}H NMR (400MHz, DMSO-d_6) δ = 9.31 (s, 1H), 6.93 (s, 1H), 3.79 - 3.77 (m, 4H), 3.50 - 3.49 (s, 4H), 2.82 (s, 3H), 1.43 (s, 9H).

Step G: To a mixture of *tert*-butyl 4-(1-chloro-4-methylpyrido[3,4-d]pyridazin-7-yl)piperazine-1-carboxylate (100 mg, 275 µmol, 1.00 *eq.*) and (*R*)-1-(2-methyl-3-(trifluoromethyl)phenyl)ethan-1-amine (55.9 mg, 275 µmol, 1.00 *eq.*) in dimethyl sulfoxide (6.00 mL) was added cesium fluoride (83.5 mg, 550 µmol, 20.3 µL, 2.00 *eq.*) and *N,N*-diisopropylethylamine (71.0 mg, 550 µmol, 95.8 µL, 2.00 *eq.*) in one portion at 20 °C in a glove box. The mixture was stirred at 130 °C for 3 hours, then cooled to 25 °C to give suspension. The suspension was filtered through a pad of celite and the filter cake was washed with ethyl acetate (30.0 mL). The combined filtrates were concentrated to give a residue. The residue was purified by prep-HPLC (column: Phenomenex Synergi C18 150 × 30 mm × 4 µm, mobile phase A: water (0.1%TFA), mobile phase B: acetonitrile; gradient: 49%-69% B) to give *tert*-butyl (*R*)-4-(4-methyl-1-((1-(2-methyl-3-(trifluoromethyl)phenyl)ethyl)amino)pyrido[3,4-*d*]pyridazin-7-yl)piperazine-1-carboxylate (50.0 mg, 94.2 µmol, 34.3% yield) as a white solid. LCMS [M-99] $^+$: 431.3.

Step H: To a mixture of *tert*-butyl (*R*)-4-(4-methyl-1-((1-(2-methyl-3-(trifluoromethyl)phenyl)ethyl)amino)pyrido[3,4-*d*]pyridazin-7-yl)piperazine-1-carboxylate (59.0

mg, 111 μmol, 1.00 *eq.*) in dichloromethane (2.00 mL) was added trifluoroacetic acid (0.40 mL) was stirred at 25 °C for 2 hours. The mixture was then concentrated to give a residue. The residue was purified by prep-HPLC (column: Phenomenex Synergi C18 150 × 30mm × 4μm, mobile phase A: water (0.1%TFA), mobile phase B: acetonitrile; Gradient: 22%-42% B) to give (*R*)-4-methyl-*N*-(1-(2-methyl-3-(trifluoromethyl)phenyl)ethyl)-7-(piperazin-1-yl)pyrido[3,4-*d*]pyridazin-1-amine (40.0 mg, 92.9 μmol, 83.6% yield, trifluoroacetic acid salt) as a white solid.

^{1}H NMR (400 MHz, CD_3OD) δ = 9.30 (s, 1H), 7.76 (s, 1H), 7.71 (d, *J* = 8.0 Hz, 1H), 7.53 (d, *J* = 7.6 Hz, 1H), 7.28 (t, *J* = 8.0 Hz, 1H), 5.52 (q, *J* = 6.8 Hz, 1H), 4.29 - 4.21 (m, 4H), 3.48 - 3.40 (m, 4H), 2.81 (s, 3H), 2.62 (s, 3H), 1.66 (d, *J* = 6.8 Hz, 3H). ^{13}C NMR (101 MHz, CD_3OD): δ ppm 161.67(q, *J* = 34.5 Hz, 1C), 161.08, 152.85, 152.73, 149.18, 144.72, 133.96, 128.71(q, *J* = 28.0, 1C), 128.21, 128.01, 126.00, 124.82 (q, *J* = 274.2 Hz, 1C), 124.21(q, *J* = 5.8 Hz, 1C), 116.81(q, *J* = 293.1 Hz, 1C), 113.13, 94.59, 42.79, 41.53, 20.09, 13.31(q, *J* = 2.53 Hz, 1C), 13.19. HRMS (m/z): [M + H]+ calcd for $C_{22}H_{25}F_3N_6$, 431.2093; found, 431.2190. HPLC (A: 0.0375% TFA in water, B: 0.01875% TFA in Acetonitrile): t_R = 3.356 min (99.0% purity).

(*R*)-4-methyl-*N*-(1-(2-methyl-3-(trifluoromethyl)phenyl)ethyl)-7-morpholinopyrido[3,4-*d*]pyridazin-1-amine (24)

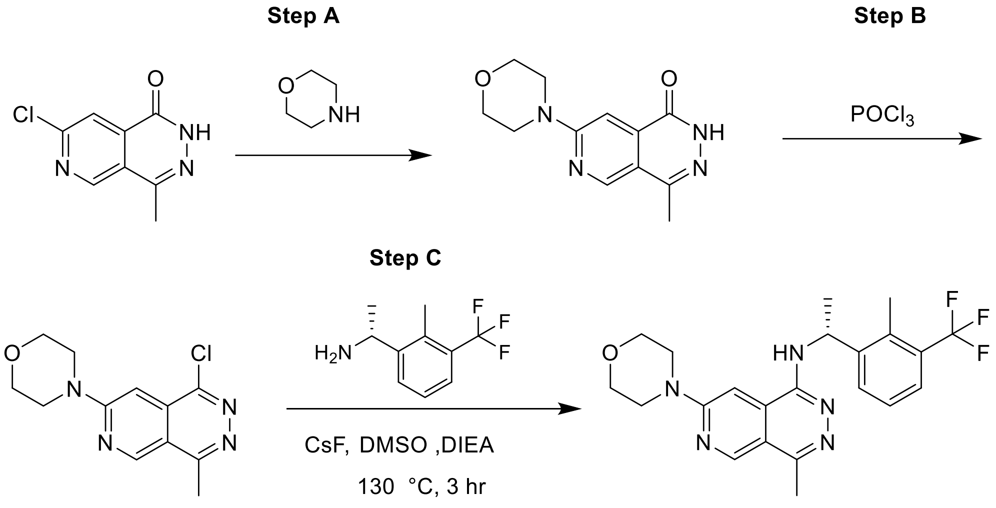

Compound 24

Step A: A solution of 7-chloro-4-methylpyrido[3,4-*d*]pyridazin-1(2*H*)-one (750 mg, 3.83 mmol, 1.00 eq), morpholine (668 mg, 7.67 mmol, 675 µL, 2.00 eq) in dioxane (10.0 mL), *t*BuOK (1.00 M in THF, 11.5 mL, 3.00 eq), RuPhos (179 mg, 383 µmol, 0.10 eq), $Pd_2(dba)_3$ (176 mg, 192 µmol, 0.05 eq) was degassed and purged with nitrogen 3 times, and the mixture was stirred at 110 °C for 3 hours under a nitrogen atmosphere. The reaction mixture was filtered, and the filtrate was concentrated under reduced pressure to give a residue. The residue was purified by prep-HPLC (column: Phenomenex luna C18 (250*70mm,15 um); mobile phase A: [water(0.05%HCl), phase B: acetonitrile; B%: 10%-40%) to give 4-methyl-7-morpholinopyrido[3,4-*d*]pyridazin-1(2*H*)-one (500 mg, 2.03 mmol, 49.2% yield) was obtained as a white solid. LCMS $[M+1]^+$: 247.0.

^{1}H NMR (400 MHz, DMSO-d_6) δ = 12.26 (s, 1H), 8.89 (s, 1H), 7.23 (s, 1H), 3.75 - 3.70 (m, 4H), 3.68 - 3.63 (m, 4H), 2.46 (s, 3H).

Step B: A solution of -methyl-7-morpholinopyrido[3,4-*d*]pyridazin-1(2*H*)-one (500 mg, 2.03 mmol, 1.00 eq) in $POCl_3$ (6.23 g, 40.6 mmol, 3.77 mL, 20.0 eq), was stirred at 110 °C for 3 hours. The reaction mixture was then concentrated under reduced pressure to remove $POCl_3$. The residue was diluted with H_2O (100 mL), and then adjusted to pH = 8 using $NaHCO_3$ solid, and then extracted with ethyl acetate (50.0 mL × 3). The combined organic layers were washed with brine (50.0 mL), dried over Na_2SO_4, filtered, and concentrated under reduced pressure to give 4-(1-

chloro-4-methylpyrido[3,4-d]pyridazin-7-yl)morpholine (500 mg, crude) was obtained as a yellow solid. LCMS $[M+1]^+$: 264.9.

^{1}H NMR (400 MHz, $CDCl_3$) δ = 9.13 (s, 1H), 6.89 (s, 1H), 3.92 - 3.86 (m, 4H), 3.81 - 3.75 (m, 4H), 2.91 (s, 3H).

Step C: To a mixture of 4-(1-chloro-4-methylpyrido[3,4-*d*]pyridazin-7-yl)morpholine (50.0 mg, 189 µmol, 1.00 *eq.*) and (*R*)-1-(2-methyl-3-(trifluoromethyl)phenyl)ethan-1-amine (38.2 mg, 188 µmol, 1.00 *eq.*) in dimethyl sulfoxide (2.00 mL) was added cesium fluoride (57.34 mg, 378 µmol, 13.9 µL, 2.00 *eq.*) and *N, N*-diisopropylethylamine (48.8 mg, 378 µmol, 65.8 µL, 2.00 *eq.*) in a glove box. The mixture was stirred at 130 °C for 3 hours then cooled to room temperature. To the mixture was added water (30.0 mL) and the reaction mixture was extracted with ethyl acetate (3 × 20.0 mL). The combined organic layers were washed with brine (saturated, 20.0 ml), dried over sodium sulfate, filtered, and concentrated under reduced pressure to give a residue. The residue was purified by prep-HPLC (column: Agela DuraShell C18 150 × 25 mm × 5 µm, mobile phase A: water (0.04% NH_3H_2O + 10 mM NH_4HCO_3), mobile phase B: acetonitrile; Gradient: 25%-55% B) to give (*R*)-4-methyl-*N*-(1-(2-methyl-3-(trifluoromethyl)phenyl)ethyl)-7-morpholinopyrido[3,4-*d*]pyridazin-1-amine (10.6 mg, 24.6 µmol, 13.3% yield) as a yellow solid. ^{1}H NMR (400 MHz, DMSO-d_6) δ = 8.97 (s, 1H), 7.72 (d, *J* = 8.0 Hz, 1H), 7.59 - 7.47 (m, 2H), 7.42 (s, 1H), 7.31 (t, *J* = 8.0 Hz, 1H), 5.65 - 5.61 (m, 1H), 3.81 - 3.74 (m, 4H), 3.71 - 3.62 (m, 4H), 2.55 (s, 6H), 1.54 (d, *J* = 6.8 Hz, 3H). ^{13}C NMR (101 MHz, DMSO-d_6): δ ppm 159.79, 151.21, 149.36, 147.45, 147.25, 134.03, 129.08, 128.01 (q, *J* = 28.4 Hz, 1C), 126.63, 125.28 (q, *J* = 273.0 Hz, 1C), 125.12, 124.26 (q, *J* = 5.9 Hz), 114.46, 93.65, 66.31, 46.72, 45.51, 22.07, 18.47, 14.56. HRMS (m/ z): [M + H]+ calcd for C22H24F3N5O, 432.1933; found, 432.2024. HPLC (0.025% $NH_3 \cdot H_2O$ in water): t_R = 13.772 min (99.8% purity).

(*R*)-*N*-(1-(2-fluoro-3-(trifluoromethyl)phenyl)ethyl)-4-methyl-7-morpholinopyrido[3,4-*d*]pyridazin-1-amine (25)

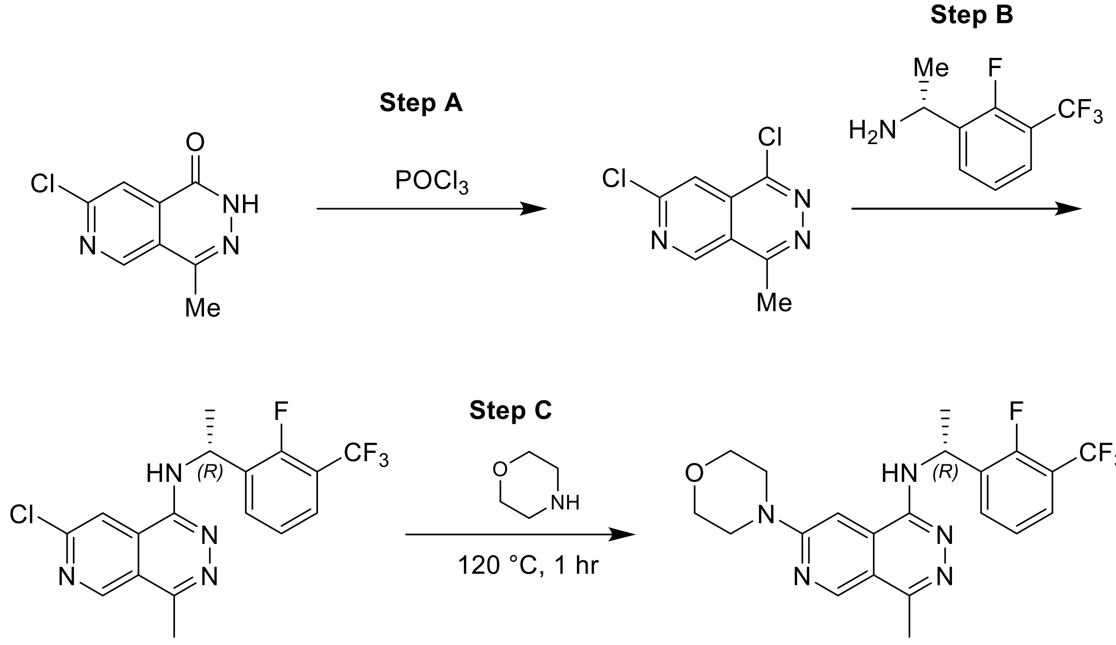

Compound 25

Step A: A solution of 7-chloro-4-methylpyrido[3,4-*d*]pyridazin-1(2*H*)-one (5.00 g, 25.6 mmol, 1.00 *eq.*) in $POCl_3$ (137 g, 893 mmol, 83.0 mL, 34.9 *eq.*) was added *N*,*N*-diisopropylethylamine (9.91 g, 76.7 mmol, 13.4 mL, 3 *eq.*) dropwise at 25 °C, then the reaction was stirred at 110 °C for 2 h. After this time the mixture was cooled to 25 °C and concentrated under vacuum to give a residue, the residue was diluted with ethyl acetate (300 mL) at 0 °C, adjusted to pH=7 with slow addition of sodium bicarbonate saturated aqueous solution. The combined organic phases were washed with brine (200 mL x 2), dried over anhydrous sodium sulfate, filtered, and concentrated under vacuum to give 1,7-dichloro-4-methylpyrido[3,4-*d*]pyridazine (4.10 g, 19.2 mmol, 74.9% yield) as a pink solid.

^{1}H NMR (400 MHz, DMSO-d_6) δ = 9.65 (s, 1H), 8.22 (s, 1H), 3.02 (s, 3H).

Step B: To a solution of 1,7-dichloro-4-methyl-pyrido[3,4-*d*]pyridazine (86.8 mg, 405 µmol, 1.20 *eq.*) and (*R*)-1-(2-fluoro-3-(trifluoromethyl)phenyl)ethan-1-amine (70.0 mg, 338 µmol, 1.00 *eq.*) in dimethyl sulfoxide (0.20 mL) was added cesium fluoride (154 mg, 1.01 mmol, 37.4 µL, 3.00 *eq.*). The mixture was stirred at 130°C for 2 hours then cooled to 25°C. The suspension was poured

into water (15.0 mL), and the aqueous phase was extracted with ethyl acetate (15.0 mL × 2). The combined organic phases were washed with brine (10.0 mL), dried over anhydrous sodium sulfate, filtered, and concentrated under reduced pressure to give a residue. The residue was purified by prep-TLC (silicon dioxide, petroleum ether/ethyl acetate = 1:1) to give (*R*)-7-chloro-*N*-(1-(2-fluoro-3-(trifluoromethyl)phenyl)ethyl)-4-methylpyrido[3,4-*d*]pyridazin-1-amine (40.0 mg, 104 µmol, 30.8% yield) as a yellow solid. LCMS $[M+1]^+$: 385.1.

Step C: To a solution of (*R*)-7-chloro-*N*-(1-(2-fluoro-3-(trifluoromethyl)phenyl)ethyl)-4-methylpyrido[3,4-*d*]pyridazin-1-amine (40.0 mg, 104 µmol, 1.00 *eq.*) and morpholine (9.06 mg, 104 µmol, 9.15 µL, 1.00 *eq.*). The mixture was stirred at 120°C for 1 hour then cooled and the reaction mixture was added water (3.00 mL) and filtered. Then the filter cake concentrated under reduced pressure to give a residue. The residue was purified by prep-HPLC (column: Waters Xbridge 150 × 25mm × 5µm; mobile phase A: water (10 mM ammonium bicarbonate), phase B: acetonitrile; B%: 44%-74%) to give (*R*)-*N*-(1-(2-fluoro-3-(trifluoromethyl)phenyl)ethyl)-4-methyl-7-morpholinopyrido[3,4-*d*]pyridazin-1-amine (14.7 mg, 33.7 µmol, 32.4% yield, 99.9% purity) as a yellow solid.

^{1}H NMR (400 MHz, CD_3OD) δ = 8.92 (s, 1H), 7.65 (t, *J* = 7.2 Hz, 1H), 7.48 (t, *J* = 7.2 Hz, 1H), 7.29 (s, 1H), 7.19 (t, *J* = 8.0 Hz, 1H), 5.67 (q, *J* = 7.2 Hz, 1H), 3.90 - 3.79 (m, 4H), 3.79 - 3.79 (m, 1H), 3.78 - 3.69 (m, 4H), 2.56 (s, 3H), 1.67 (d, *J* = 6.8 Hz, 3H). ^{13}C NMR (101 MHz, CD_3OD) δ = 161.58, 158.99 (dd, *J* = 1.4, 254.5 Hz, 1C), 152.92, 150.60, 149.59, 135.80 (d, *J* = 13.1 Hz, 1C), 132.57 (d, *J* = 4.3 Hz, 1C), 127.09, 126.44 (q, *J* = 4.4 Hz, 1C), 125.44 (d, *J* = 4.3 Hz, 1C), 124.49 (q, *J* =272.0 Hz, 1C) ,119.23 (dq, *J* = 13.1, 32.8 Hz, 1C), 115.83, 94.26, 67.80, 46.62, 46.56 (d, *J* = 4.4 Hz, 1C), 21.92, 17.91. HRMS (m/ z): [M + H]+ calcd for $C_{21}H_{21}F_4N_5O$, 436.1682; found, 436.1780. HPLC (A: 0.0375% TFA in water, B: 0.01875% TFA in Acetonitrile): t_R = 4.348 min (99.9% purity).

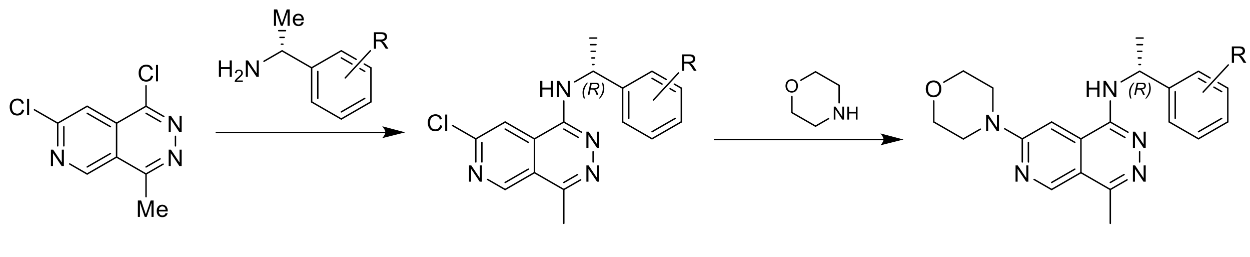

General Procedure B: *Step 1*: A mixture of 1,7-dichloro-4-methylpyrido[3,4-d]pyridazine (1.20 *eq.*), a chiral α-methyl benzyl amine (1.00 *eq.*), in dimethyl sulfoxide (0.1 M) was added cesium fluoride (3.00 – 5.00 *eq.*). The mixture was stirred at 130°C until the reaction was completed then cooled to 25°C. The suspension was poured into water, and the aqueous phase was extracted with ethyl acetate. The combined organic phases were washed with brine, dried over anhydrous sodium sulfate, filtered, and concentrated under reduced pressure to give a residue. The residue was purified by prep-TLC (SiO_2, dichloromethane/methanol) to give the desired product. *Step 2*: A solution of the requisite chloro-phthalazine (1.00 *eq.*) and morpholine (1.00-3.00 *eq.*) was stirred at 120°C until the reaction was completed. The mixture was then cooled and water was added, then the mixture was filtered and the filter cake was collected. The filter cake was further concentrated under reduced pressure to give a residue. The residue was washed with water several times, and if needed, purified by prep-HPLC to give the desired product.

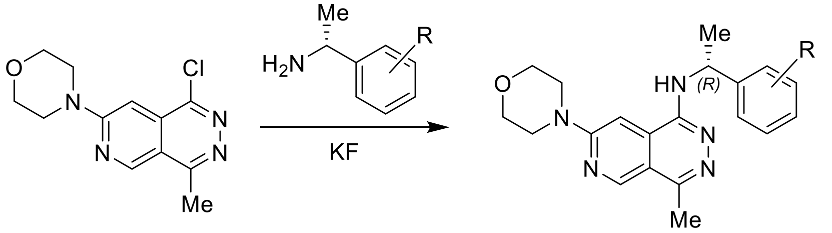

General Procedure C: To a solution of 4-(1-chloro-4-methylpyrido[3,4-d]pyridazin-7-yl)morpholine (1.00 *eq.*) and chiral α-methyl benzyl amine (1.00 *eq.*) in dimethyl sulfoxide (0.1 M) was added potassium fluoride (3.00-5.00 *eq.*), and the mixture was stirred at 130 °C until completion of the reaction. The reaction mixture was cooled to 25 °C, quenched by addition water and extracted with ethyl acetate. The combined organic layers were washed with brine, dried over sodium sulfate, filtered, and concentrated under reduced pressure to give a residue. The residue was purified by prep-HPLC to give the final compound.

The following compounds were prepared following **General Procedure B** or **General Procedure C**:

(*R*)-4-methyl-7-morpholino-*N*-(1-(6-(trifluoromethyl)pyridin-2-yl)ethyl)pyrido[3,4-*d*]pyridazin-1-amine (26)

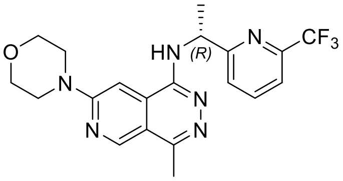

Compound 26

The following compound was made using **General Procedure C:** Yellow solid. ^{1}H NMR (400 MHz, CD_3OD) δ = 9.12 (s, 1H), 8.47 (s, 1H), 7.97 - 7.89 (m, 1H), 7.69 (d, *J* = 8.0 Hz, 1H), 7.63 (d, *J* = 8.0 Hz, 1H), 7.43 (s, 1H), 5.42 (q, *J* = 6.8 Hz, 1H), 3.85 (s, 8H), 2.70 (s, 3H), 1.72 (d, *J* = 6.8 Hz, 3H). ^{13}C NMR (101 MHz, CD_3OD) δ = 169.66, 165.83, 162.29, 153.99, 152.37, 150.09, 148.62 (d, *J* = 34.1 Hz, 1C), 139.84, 128.19, 125.25, 123.22 (q, *J* = 274.7, 1C), 119.98 (d, *J* = 3.3 Hz, 1C), 114.83, 94.75, 67.74, 53.94, 46.61, 21.42, 16.43. HRMS (m/ z): [M + H]+ calcd for $C_{20}H_{21}F_3N_6O$, 419.1729; found, 419.1814. HPLC (0.025% $NH_3 \cdot H_2O$ in water): t_R = 12.065 min (99.9% purity).

Synthesis of (*R*)-2-(1-aminoethyl)-6-(trifluoromethyl)pyridin-4-amine

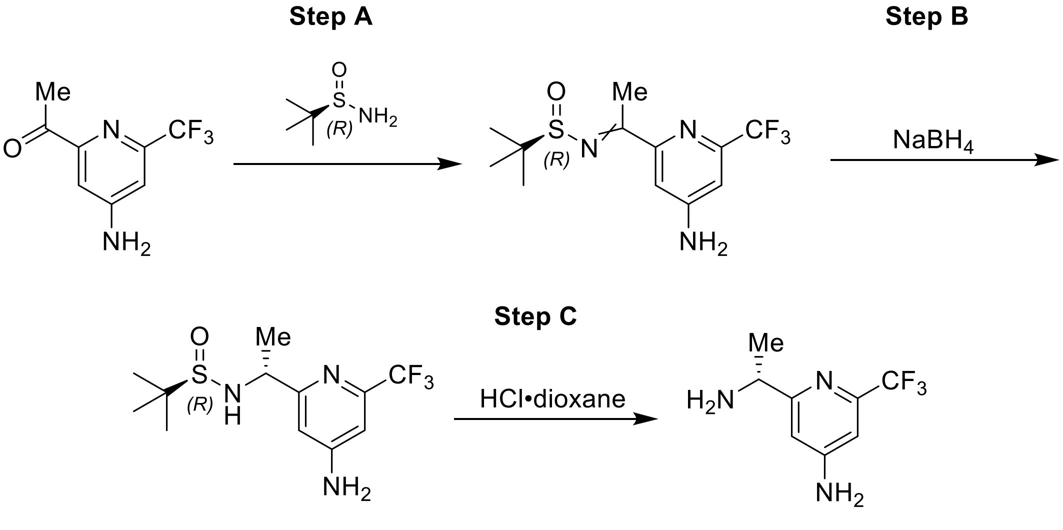

Step A: To a solution of 1-(4-amino-6-(trifluoromethyl)pyridin-2-yl)ethan-1-one (35.6 g, 175 mmol, 1.00 *eq.*) and (*R*)-2-methylpropane-2-sulfinamide (25.4 g, 209 mmol, 1.20 *eq.*) in THF (350 mL) was added titanium (IV) isopropoxide (149 g, 524 mmol, 155 mL, 3.00 *eq.*), and 1,2-

dimethoxyethane (15.7 g, 175 mmol, 18.1 mL, 1.00 *eq.*). The reaction mixture was stirred at 80 °C for 12 hours, after which point was added water (50.0 mL) to give a suspension. The suspension was filtered, the filtrate was concentrated under reduced pressure to give a residue, the residue was purified by silica gel chromatography(petroleum ether/ethyl acetate=10/1 to 1/1) to give (*R*)-*N*-(1-(4-amino-6-(trifluoromethyl)pyridin-2-yl)ethylidene)-2-methylpropane-2-sulfinamide (44.0 g, 143 mmol, 82.0% yield) as brown oil.

^{1}H NMR (400 MHz, $CDCl_3$) δ = 7.45 (d, *J* = 2.0 Hz, 1H), 6.97 (d, *J* = 2.0 Hz, 1H), 4.56 (br s, 2H), 2.82 (s, 3H), 1.33 (s, 9H).

Step B: To a solution of (*R*)-*N*-(1-(4-amino-6-(trifluoromethyl)pyridin-2-yl)ethylidene)-2-methylpropane-2-sulfinamide (44.0 g, 143 mmol, 1.00 *eq.*) in THF (400 mL) was added sodium borohydride (16.3 g, 430 mmol, 3.00 *eq.*) at 0 °C in portion wise, then the reaction was stirred at 0 °C for 1 hour. The mixture was slowly poured into water (200 mL) and stirred for 5 minutes, then extracted with ethyl acetate (300 mL×3). The combined organic phases were washed with brine (200 mL×3), dried with anhydrous sodium sulfate, filtered and concentrated under reduced pressure to give a residue. The residue was purified by column chromatography (SiO_2, petroleum ether/ethyl acetate=10/1 to 1/1) to give (*R*)-*N*-((*R*)-1-(4-amino-6-(trifluoromethyl)pyridin-2-yl)ethyl)-2-methylpropane-2-sulfinamide (24.0 g, 76.2 mmol, 53.2% yield, 98.2% purity) as a brown oil.

^{1}H NMR (400 MHz, $CDCl_3$) δ = 6.63 (d, *J* = 2.0 Hz, 1H), 6.56 (d, *J* = 2.0 Hz, 1H), 5.06 (d, *J* = 6.0 Hz, 1H), 4.69 (s, 2H), 4.46 - 4.39 (m, 1H), 1.45 (d, *J* = 6.8 Hz, 3H), 1.27 (s, 9H).

Step C: To a solution of (*R*)-*N*-((*R*)-1-(4-amino-6-(trifluoromethyl)pyridin-2-yl)ethyl)-2-methylpropane-2-sulfinamide (23.5 g, 76.0 mmol, 1.00 *eq*) in HCl/dioxane (200 mL) was stirred at 25 °C for 2 hours. The mixture was filtered, and the filter cake was washed with ethyl acetate (100 mL), then the filter cake was collected and dried under vacuum to give (*R*)-2-(1-aminoethyl)-6-(trifluoromethyl)pyridin-4-amine (hydrochloride salt) as a white solid.

^{1}H NMR (400 MHz, DMSO-d_6) δ = 8.43 (br s, 3H), 6.93 (br d, *J* = 2.0 Hz, 2H), 6.74 (d, *J* = 1.6 Hz, 1H), 4.34 - 4.27 (m, 1H), 1.45 (d, *J* = 6.8 Hz, 3H).

(*R*)-*N*-(1-(4-amino-6-(trifluoromethyl)pyridin-2-yl)ethyl)-4-methyl-7-morpholinopyrido[3,4-*d*]pyridazin-1-amine (27)

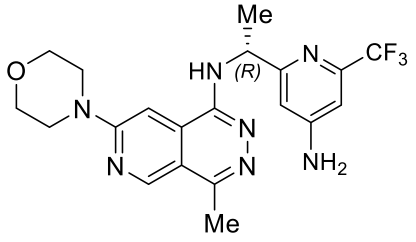

Compound 27

The following compound was made using **General Procedure B:** Yellow solid. ^{1}H NMR (400 MHz, DMSO-d_6): δ = 9.01 (s, 1H), 7.46 (s, 1H), 7.42 (br d, *J* = 7.2 Hz, 1H), 6.73 (s, 1H), 6.64 (s, 1H), 6.42 (s, 2H), 5.29-5.19 (quin, *J* = 6.8 Hz, 1H), 3.82 - 3.75 (m, 4H), 3.73 -3.65 (m, 4H), 2.58 (s, 3H), 1.56 (br d, *J* = 7.2 Hz, 3H). ^{13}C NMR (101 MHz, DMSO-d_6) δ = 165.44, 159.77, 156.46, 151.61, 149.40, 147.48, 147.0 (q, *J* = 32.1 Hz, 1C), 125.19, 122.55 (q, *J* = 275.0 Hz, 1C), 114.58, 106.28, 103.92, 93.84, 66.33, 51.82, 45.53, 21.69, 18.57. HRMS (m/ z): [M + H]+ calcd for $C_{20}H_{22}F_3N_7O$, 434.1838; found, 434.1926. HPLC (A: 0.0375% TFA in water, B: 0.01875% TFA in Acetonitrile): t_R = 3.324 min (97.0% purity).

Synthesis of (*R*)-1-(2-methylpyridin-3-yl)ethan-1-amine

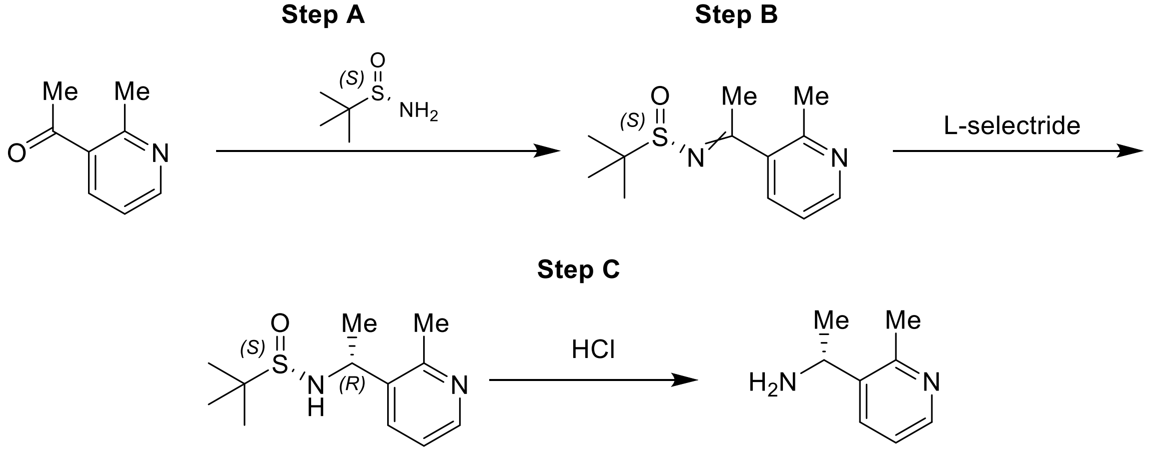

Step A: To a solution of 1-(2-methylpyridin-3-yl)ethan-1-one (800 mg, 5.92 mmol, 1.00 *eq.*) and (*S*)-2-methylpropane-2-sulfinamide (933 mg, 7.69 mmol, 1.30 *eq.*) in tetrahydrofuran (8.00 mL) was added titanium (IV) ethoxide (2.70 g, 11.8 mmol, 2.45 mL, 2.00 *eq.*) and 1,2-dimethoxyethane (533 mg, 5.92 mmol, 615 µL, 1.00 *eq.*), and the mixture was stirred at 70 °C for 16 hours. After

cooling to 25°C the mixture was concentrated under reduced pressure and purified by column chromatography (SiO_2, petroleum ether / ethyl acetate = 5/1 to 1/1) to give (*S*)-2-methyl-*N*-(1-(2-methylpyridin-3-yl)ethylidene)propane-2-sulfinamide (1.25 g, 5.24 mmol, 88.6% yield) as a yellow oil. LCMS $[M+1]^+$: 239.2.

Step B: To a solution of (*S*)-2-methyl-*N*-(1-(2-methylpyridin-3-yl)ethylidene)propane-2-sulfinamide (1.25 g, 5.24 mmol, 1.00 *eq.*) in tetrahydrofuran (7.00 mL) was added dropwise *L*-selectride (1.0 M in THF, 7.87 mL, 1.50 *eq.*) at -78 °C over 30 minutes, then stirred for an additional 1 hour at -78°C. The reaction mixture was then quenched by addition saturated ammonium chloride solution (in water, 30.0 mL) at 0 °C, and stirred for another 1 hour at 25 °C. The solution was then extracted with ethyl acetate (50.0 mL× 3), and the combined organic layers were washed with brine (30.0 mL ×2), dried over anhydrous sodium sulfate, filtered, and concentrated under reduced pressure. The residue was purified twice by column chromatography (SiO_2, petroleum ether / ethyl acetate = 5/1 to 0/1) to give (*S*)-2-methyl-*N*-((*R*)-1-(2-methylpyridin-3-yl)ethyl)propane-2-sulfinamide (600 mg, 2.50 mmol, 47.6% yield) as a white solid. LCMS $[M+1]^+$: 432.3.

^{1}H NMR (400 MHz, $CDCl_3$) δ = 8.36 (dd, *J* =1.2, 3.6 Hz, 1H), 7.64 (dd, *J* =1.6, 8.0 Hz, 1H), 7.12 (dd, *J* =4.8, 7.6 Hz, 1H), 4.81 - 4.70 (m, 1H), 2.58 (s, 3H), 1.47 (d, *J* =6.8 Hz, 3H), 1.14 (s, 9H).

SFC conditions: Column: Chiralpak AD-3 50 × 4.6 mm I.D., 3um Mobile phase: Phase A: CO_2, and Phase B: for MeOH(0.05% diethylamine); Gradient elution: MeOH (0.05% diethylamine) in CO2 from 5% to 40%f Flow rate: 3mL/min;Detector: PDA Column Temp: 35 °C; Back Pressure: 100 Bar.

Step C: A mixture of (*S*)-2-methyl-*N*-((*R*)-1-(2-methylpyridin-3-yl)ethyl)propane-2-sulfinamide (600 mg, 2.50 mmol, 1.00 *eq.*) in HCl•dioxane (3.00 mL) was stirred at 0 °C for 30 minutes under a nitrogen atmosphere. After this time, a white precipitate was formed, and the suspension was filtered. The cake was collected and dried under vacuum, and the residue was further purified by prep-HPLC [column: Waters Xbridge 150 × 25 mm × 5 um; mobile phase: phase A: water (0.05% ammonium hydroxide v/v), phase B: MeCN; B%: 3%-33%] to give (*R*)-1-(2-methylpyridin-3-yl)ethan-1-amine (370 mg, 2.23 mmol, 89.2% yield, 82% purity) as an colorless oil. LCMS $[M-16]^+$: 120.3.

(*R*)-4-methyl-*N*-(1-(2-methylpyridin-3-yl)ethyl)-7-morpholinopyrido[3,4-*d*]pyridazin-1-amine (28)

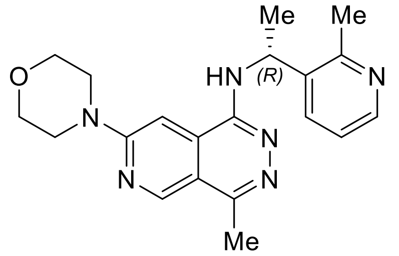

Compound 28

The following compound was made using **General Procedure C:** Yellow solid. ^{1}H NMR (400 MHz, CD_3OD) δ = 9.17 (s, 1H), 8.39 (s, 1H), 8.26 (dd, *J* = 1.2, 4.8 Hz, 1H), 7.85 (dd, *J* = 1.2, 8.0 Hz, 1H), 7.45 (s, 1H), 7.21 (dd, *J* = 4.8, 8.0 Hz, 1H), 5.42 (q, *J* = 6.8 Hz, 1H), 3.93 - 3.88 (m, 4H), 3.87 - 3.83 (m, 4H), 2.74 (s, 3H), 2.72 (s, 3H), 1.66 (d, *J* = 7.2 Hz, 3H). ^{13}C NMR (101 MHz, CD_3OD) δ = 168.74, 162.78, 156.94, 153.98, 153.57, 150.38, 147.69, 140.38, 135.03, 128.79, 123.56, 114.20, 94.94, 67.72, 46.61, 21.57, 21.36, 15.39. HRMS (m/ z): [M + H]+ calcd for $C_{20}H_{24}N_6O$, 365.2012; found 365.2083. HPLC (0.025% $NH_3{\cdot}H_2O$ in water): t_R = 9.402 min (99.3% purity).

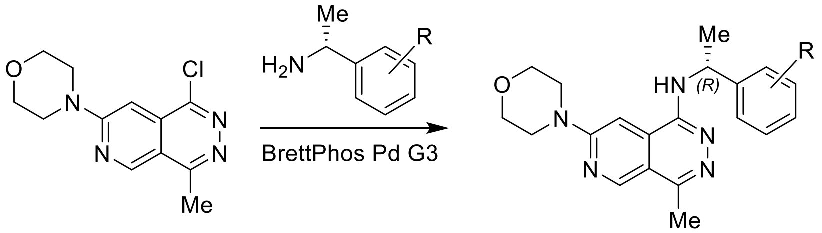

<u>**General Procedure D**</u>: A solution of 4-(1-chloro-4-methylpyrido[3,4-d]pyridazin-7-yl)morpholine (1.00 *eq.*), chiral α-methyl benzyl amine (1.00 *eq.*), *tert*-butoxide or cesium carbonate (4.00 eq.), and BrettPhos Pd G3 (0.10 *eq.*) in dioxane or toluene (0.20 M) is stirred under a nitrogen atmosphere at 100 °C until completion of the reaction. The reaction mixture was cooled to 25 °C and then concentrated to give a residue. The residue is further purified by prep-HPLC or column chromatography to give the desired product.

Synthesis of (*R*)-1-(1,3-dimethyl-1*H*-pyrazol-4-yl)ethan-1-amine

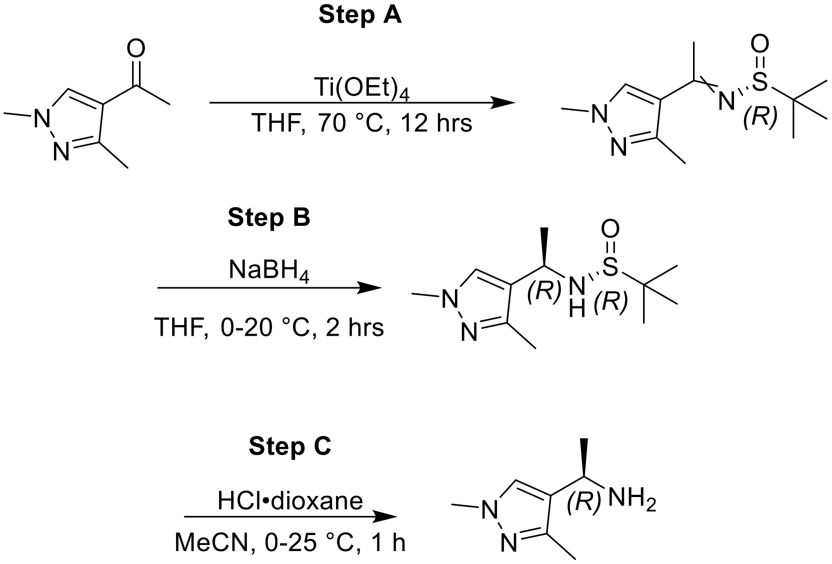

Step A: To a solution of 1-(1,3-dimethyl-1*H*-pyrazol-4-yl)ethan-1-one (1.20 g, 8.69 mmol, 1.00 *eq.*) and (*R*)-2-methylpropane-2-sulfinamide (1.58 g, 13.0 mmol, 1.50 *eq.*) in THF (10.0 mL) was added titanium (IV) ethoxide (24.7 g, 86.9 mmol, 25.6 mL, 10.0 *eq.*). The mixture was stirred at 70 °C for 12 hours, then cooled and concentrated under reduced pressure to remove any volatiles. The residue was diluted with water (50.0 mL) and extracted with ethyl acetate (30.0 mL × 3). The combined organic layers were washed with brine (50.0 mL × 3), dried over sodium sulfate, filtered, and concentrated under reduced pressure to give a residue. The residue was purified by column chromatography (SiO_2, petroleum ether / ethyl acetate = 5/1 to 2/1) to give compound (*R*)-*N*-(1-(1,3-dimethyl-1*H*-pyrazol-4-yl)ethylidene)-2-methylpropane-2-sulfinamide (700 mg, 2.90 mmol, 33.4% yield) as a yellow solid. LCMS $[M+1]^+$: 242.1.

Step B**:** To a solution of (*R*)-*N*-(1-(1,3-dimethyl-1*H*-pyrazol-4-yl)ethylidene)-2-methylpropane-2-sulfinamide (700 mg, 2.90 mmol, 1.00 *eq.*) in THF (10.0 mL) was added sodium borohydride (329 mg, 8.70 mmol, 3.00 *eq.*) and the mixture was stirred at 0-20 °C for 2 hours. The reaction mixture was quenched by addition water (20.0 mL) at 25°C, and then extracted with ethyl acetate (60.0 mL). The combined organic layers were washed with brine (10.0 mL × 3), dried over sodium sulfate, filtered, and concentrated under reduced pressure to give a residue. The residue was purified by prep-HPLC (column: 3_Phenomenex Luna C18 75 × 30 mm × 3 µm; mobile phase A: water (0.05% HCl), mobile phase B: acetonitrile; B%: 16%-36%) to give (*R*)-*N*-((*R*)-1-(1,3-dimethyl-1*H*-pyrazol-4-yl)ethyl)-2-methylpropane-2-sulfinamide (80.0 mg, 329 µmol, 11.3%

yield) as a yellow solid.

Step C: To a solution of (*R*)-*N*-((*R*)-1-(1,3-dimethyl-1*H*-pyrazol-4-yl)ethyl)-2-methylpropane-2-sulfinamide (40.0 mg, 164 μmol, 1.00 *eq.*) in acetonitrile (1.00 mL) was added HCl in dioxane (4.0 M, 1.00 mL, 24.3 *eq.*) at 0 °C. The mixture was slowly warmed to 25 °C over 1 hour. The mixture was filtered to obtain (*R*)-1-(1,3-dimethyl-1*H*-pyrazol-4-yl)ethan-1-amine (20.0 mg, 144 μmol, 87.4% yield) as a off-white solid.

(*R*)-*N*-(1-(1,3-dimethyl-1*H*-pyrazol-4-yl)ethyl)-4-methyl-7-morpholinopyrido[3,4-*d*]pyridazin-1-amine (29)

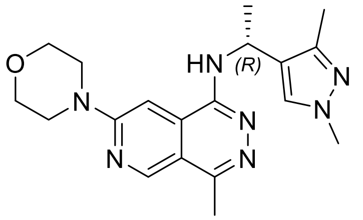

Compound 29

The following compound was made using **General Procedure D:** Yellow solid. ^{1}H NMR (400 MHz, CD_3OD): δ ppm 9.06 (s, 1H), 8.53 (br s, 1H), 7.53 (s, 1H), 7.28 (s, 1H), 5.35 (q, *J* = 6.4 Hz, 1H), 3.79 (s, 8H), 3.76 (s, 3H), 2.73 (s, 3H), 2.19 (s, 3H), 1.62 (d, *J* = 6.8 Hz, 3H). ^{13}C NMR (101 MHz, CD_3OD): δ ppm 162.28, 153.64, 152.28, 149.59, 147.73, 131.03, 128.38, 123.21, 114.86, 94.86, 67.71, 46.60, 43.61, 38.64, 21.25, 16.46, 12.09. HRMS (m/ z): $[M + H]^+$ calcd for $C_{19}H_{26}N_7O$, 368.2121; found, 368.2210. HPLC (A: 0.0375% TFA in water, B: 0.01875% TFA in Acetonitrile): t_R = 3.107 min (91.4% purity).

Synthesis of (*R*)-1-(1-methyl-3-(trifluoromethyl)-1*H*-pyrazol-4-yl)ethan-1-amine

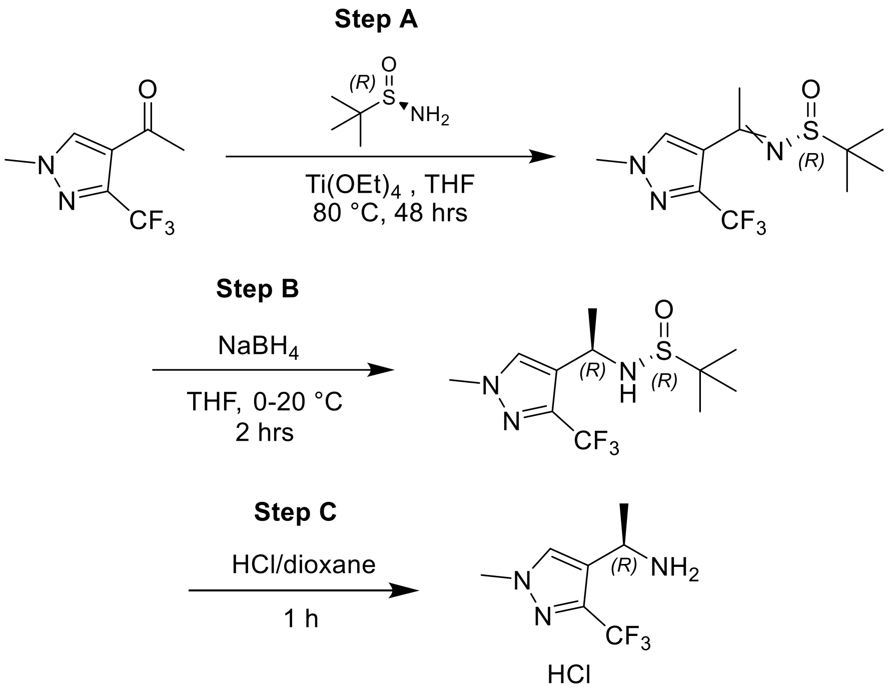

Step A: To a solution of 1-(1-methyl-3-(trifluoromethyl)-1H-pyrazol-4-yl)ethan-1-one (1.80 g, 9.37 mmol, 1.00 *eq.*) and (*R*)-2-methylpropane-2-sulfinamide (1.48 g, 12.2 mmol, 1.30 *eq.*) in THF (30.0 mL) was added titanium (IV) ethoxide (26.6 g, 93.7 mmol, 27.7 mL, 10.0 *eq.*), and the mixture was stirred at 80 °C for 48 hours. The mixture was poured into water (60.0 mL) and stirred for 5 minutes, filtered, and the aqueous phase was extracted with ethyl acetate (40.0 mL × 3). The combined organic phases were washed with brine (20.0 mL × 2), dried over anhydrous sodium sulfate, filtered, and concentrated under reduced pressure to give a residue. The residue was purified by column chromatography (SiO_2, petroleum ether / ethyl acetate = 10/1 to 4/1) to give (*R*)-2-methyl-*N*-(1-(1-methyl-3-(trifluoromethyl)-1*H*-pyrazol-4-yl)ethylidene)propane-2-sulfinamide (2.40 g, 8.13 mmol, 86.8% yield) as a yellow oil.

^{1}H NMR (400 MHz, CD_3OD) δ = 8.39 (s, 1H), 3.97 (s, 3H), 2.67 (s, 3H), 1.28 (s, 9H).

Step B: To a solution of (*R*)-2-methyl-*N*-(1-(1-methyl-3-(trifluoromethyl)-1*H*-pyrazol-4-yl)ethylidene)propane-2-sulfinamide (2.57 g, 8.70 mmol, 1.00 *eq.*) in tetrahydrofuran (30.0 mL) was added sodium borohydride (987 mg, 26.1 mmol, 3.00 *eq.*). The mixture was stirred between 0-20 °C for 2 hours. The mixture was then poured into water (10.0 mL), and the aqueous phase was extracted with ethyl acetate (30.0 mL × 3). The combined organic phases were washed with brine (30.0 mL × 3), dried over anhydrous sodium sulfate, filtered, and concentrated under reduced

pressure to give a residue. The residue was purified by column chromatography (silicon dioxide, petroleum ether / ethyl acetate = 20/1 to 2/1) to give (*R*)-2-methyl-*N*-((*R*)-1-(1-methyl-3-(trifluoromethyl)-1*H*-pyrazol-4-yl)ethyl)propane-2-sulfinamide (1.20 g, 4.04 mmol, crude) as yellow oil.

Step C: To a solution of (*R*)-2-methyl-*N*-((*R*)-1-(1-methyl-3-(trifluoromethyl)-1*H*-pyrazol-4-yl)ethyl)propane-2-sulfinamide (950 mg, 3.19 mmol, 1.00 *eq.*) was added HCl in dioxane (4.00 M, 2.40 mL, 3.00 *eq.*). The mixture was stirred at 0 °C for 1 hour then filtered and concentrated under reduced pressure to give a residue. The residue was purified by prep-HPLC (column: 3_Phenomenex Luna C18 75 × 30 mm × 3 um; mobile phase A: water(0.05%HCl), mobile phase B: acetonitrile; B%: 0%-10%) to give (*R*)-1-(1-methyl-3-(trifluoromethyl)-1*H*-pyrazol-4-yl)ethan-1-amine (250 mg, 1.29 mmol, 40.5% yield, 99.9% purity, HCl salt) as a white solid.

^{1}H NMR (400 MHz, DMSO-d_6) δ = 8.34 (br s, 3H), 8.09 (s, 1H), 4.40 (q, *J* = 6.4 Hz, 1H), 3.94 (s, 3H), 1.48 (d, *J* = 6.8 Hz, 3H).

(*R*)-4-methyl-*N*-(1-(1-methyl-3-(trifluoromethyl)-1*H*-pyrazol-4-yl)ethyl)-7-morpholinopyrido[3,4-*d*]pyridazin-1-amine (30)

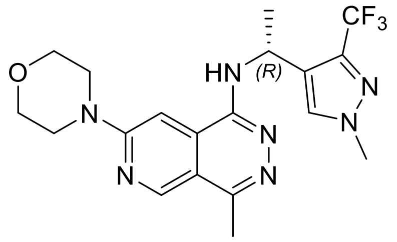

Compound 30

The following compound was made using **General Procedure D:** Yellow solid. ^{1}H NMR (400 MHz, CD_3OD) δ = 8.96 (d, *J* = 5.2 Hz, 1H), 7.71 (s, 1H), 7.17 (s, 1H), 5.59 (q, *J* = 6.8 Hz, 1H), 3.87 (s, 3H), 3.83 – 3.74 (m, 4H), 3.72 – 3.58 (m, 4H), 2.64 (d, *J* = 2.0 Hz, 3H), 1.63 (d, *J* = 6.8 Hz, 3H). ^{13}C NMR (101 MHz, CD_3OD) δ = 161.56, 153.02, 150.46, 149.36, 139.66 (q, *J* = 36.6 Hz, 1C), 132.51, 127.33, 126.00, 123.37 (q, *J* = 268.3 Hz, 1C), 115.98, 94.45, 67.78, 46.62, 43.02, 39.65, 21.94, 18.02 HRMS (m/ z): [M + H]+ calcd for $C_{19}H_{22}F_3N_7O$, 422.1838; found, 422.1929. HPLC (0.025% $NH_3 \cdot H_2O$ in water) : t_R = 10.480 min (98.9% purity).

Synthesis of (*R*)-3-(1-aminoethyl)-2-methylbenzonitrile

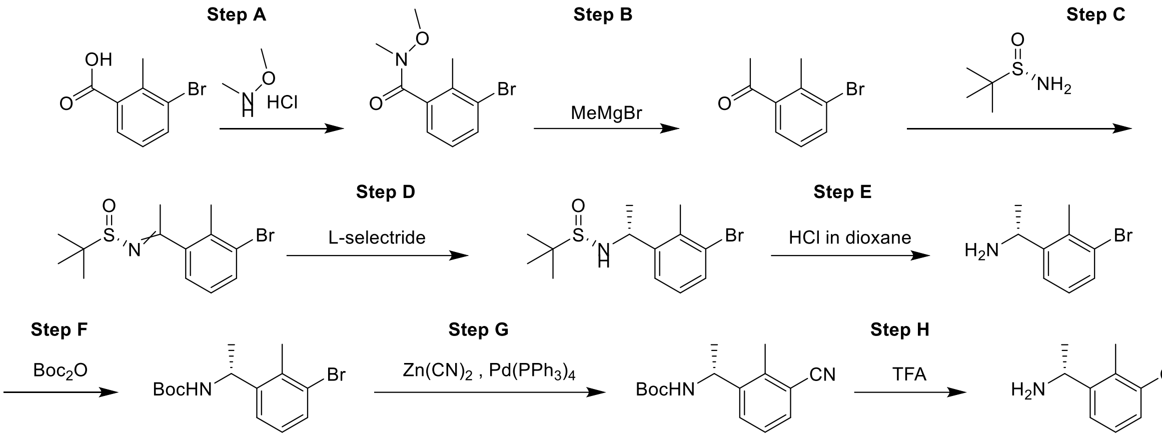

Step A: To a solution of 3-bromo-2-methylbenzoic acid (100 g, 465 mmol, 1.00 *eq.*) and *N*, O-dimethylhydroxylamine hydrochloride (68.6 g, 512 mmol, 1.10 *eq.*, HCl) in DMF (1000 mL) was added 1-[bis(dimethylamino)methylene]-1*H*-1,2,3-triazolo[4,5-*b*]pyridinium 3-oxid hexafluorophosphate (195 g, 512 mmol, 1.10 *eq.*) and *N*,*N*-diisopropylethylamine (180 g, 1.40 mol, 243 mL, 3.00 *eq.*). The mixture was stirred at 25 °C for 2 hours, then poured into water (1000 mL) and stirred for 15 minutes. The aqueous phase was extracted with ethyl acetate (1000 mL × 3). The combined organic phases were washed with brine (1000 mL × 5), dried over anhydrous sodium sulfate, filtered and concentrated *in vacuo* to give 3-bromo-*N*-methoxy-*N*,2-dimethylbenzamide (120 g, crude) as yellow oil. LCMS $[M+1]^+$: 258.0.

Step B: To a solution of 33-bromo-*N*-methoxy-*N*,2-dimethylbenzamide (120 g, 465 mmol, 1.00 *eq.*) in THF (100 mL) was added methyl magnesium bromide (3.0 M, 180 mL, 1.16 *eq.*) at 0 °C. The mixture was stirred between 0-40 °C for 3 hours, then the mixture was cooled to 0 °C and hydrochloric acid (6.0 N) (450 mL) was added dropwise, and stirred for 2 hours between 40-45 °C. Then the mixture was cooled to 25 °C and poured into a saturated ammonium chloride solution (9000 mL). The aqueous phase was extracted with ethyl acetate (1500 mL × 3). The combined organic phase was washed with brine (1000 mL × 3), dried over anhydrous sodium sulfate, filtered, and concentrated under vacuum to give 1-(3-bromo-2-methylphenyl)ethan-1-one (90.0 g, 422 mmol, 90.9% yield) as yellow oil.

^{1}H NMR (400 MHz, CD_3OD) δ = 7.70 (dd, *J* = 1.2, 8.0 Hz, 1H), 7.62 (dd, *J* = 0.8, 7.6 Hz, 1H), 7.19 (t, *J* = 8.0 Hz, 1H), 2.56 (s, 3H), 2.46 (s, 3H).

Step C: To a solution of 1-(3-bromo-2-methylphenyl)ethan-1-one (88.0 g, 413 mmol, 1.00 *eq.*) and (*S*)-2-methylpropane-2-sulfinamide (60.1 g, 496 mmol, 1.20 *eq.*) in THF (100 mL) was added titanium (IV) ethoxide (471 g, 2.07 mol, 428 mL, 5.00 *eq.*) and diglyme (55.4 g, 413 mmol, 59.1 mL, 1.00 *eq.*). The mixture was stirred at 80 °C for 2 hours then poured into water (300 mL) and stirred for 15 minutes. The mixture was then filtered and concentrated *in vacuo* to give a residue. The residue was purified by column chromatography (SiO_2, petroleum ether / ethyl acetate = 100/1 to 40/1) to give (*S*)-*N*-(1-(3-bromo-2-methylphenyl)ethylidene)-2-methylpropane-2-sulfinamide (110 g, 348 mmol, 84.2% yield) as yellow oil.

^{1}H NMR (400 MHz, CD_3OD) δ = 7.63 (br t, *J* = 6.8 Hz, 2H), 7.28 (br d, *J* = 7.6 Hz, 1H), 7.17 (t, *J* = 8.0 Hz, 2H), 7.14 - 7.02 (m, 1H), 2.67 (s, 3H), 2.50 (br d, *J* = 4.8 Hz, 3H), 2.42 (s, 3H), 2.31 (br d, *J* = 17.2 Hz, 3H), 1.31 - 1.26 (m, 9H), 1.24 - 1.16 (m, 9H)

Step D: To a solution of (*S*)-*N*-(1-(3-bromo-2-methylphenyl)ethylidene)-2-methylpropane-2-sulfinamide (109 g, 345 mmol, 1.00 *eq.*) in THF (1100 mL) was added *L*-selectride (1.0 M, 689 mL, 2.00 *eq.*) at -78 °C. The mixture was stirred at -78 °C for 2 hours then poured into a saturated aqueous solution of ammonium chloride (1000 mL) and stirred for 60 minutes at 25 °C. The aqueous phase was extracted with ethyl acetate (1000 mL × 3). The combined organic phase were washed with brine (500 mL × 3), dried over anhydrous sodium sulfate, filtered, and concentrated under vacuum to give a residue. The residue was purified by column chromatography (SiO_2, petroleum ether / ethyl acetate = 100/1 to 2/1) to give a residue. The residue was further washed with petroleum ether to give (*S*)-*N*-((*R*)-1-(3-bromo-2-methylphenyl)ethyl)-2-methylpropane-2-sulfinamide (70.0 g, 220 mmol, 63.8% yield) as a white solid. LCMS $[M+1]^+$: 318.1.

Step E: To a solution of (*S*)-*N*-((*R*)-1-(3-bromo-2-methylphenyl)ethyl)-2-methylpropane-2-sulfinamide (71.0 g, 223 mmol, 1.00 *eq.*) in an HCl/dioxane solution (300 mL) and MeOH (300 mL) was stirred at 0 °C for 30 minutes. The mixture was concentrated *in vacuo* to give a (*R*)-1-(3-bromo-2-methylphenyl)ethan-1-amine (55.0 g, crude, HCl) as a white solid. LCMS $[M+1]^+$: 214.1.

Step F: To a solution of (*R*)-1-(3-bromo-2-methylphenyl)ethan-1-amine (55.0 g, 220 mmol, 1.00 *eq.*, HCl) and Boc_2O (48.4 g, 222 mmol, 50.9 mL, 1.01 *eq.*) in dichloromethane (500 mL) was added *N*,*N*-diisopropylethylamine (56.7 g, 439 mmol, 76.5 mL, 2.00 *eq.*). The mixture was stirred

between 0-25 °C for 30 minutes, then concentrated under vacuum to give a residue. The residue was purified by column chromatography (SiO_2, petroleum ether / ethyl acetate = 1/0 to 100/1) to give a residue. The residue was further washed with petroleum ether to give *tert*-butyl (*R*)-(1-(3-bromo-2-methylphenyl)ethyl)carbamate (51.0 g, 162 mmol, 73.9% yield) as a white solid. LCMS $[M-55]^+$: 258.0.

^{1}H NMR (400 MHz, CD_3OD) δ = 7.43 (d, *J* = 8.0 Hz, 1H), 7.32 (d, *J* = 8.0 Hz, 1H), 7.10 - 7.03 (m, 1H), 4.93 (br d, *J* = 6.4 Hz, 2H), 2.45 (s, 3H), 1.41 (br s, 9H), 1.33 (d, *J* = 6.8 Hz, 3H).

Step G: To a solution of *tert*-butyl (*R*)-(1-(3-bromo-2-methylphenyl)ethyl)carbamate (51.0 g, 162 mmol, 1.00 *eq.*) in DMF (540 mL) was added zinc cyanide (22.9 g, 195 mmol, 12.4 mL, 1.20 *eq.*) and $Pd(PPh_3)_4$ (18.8 g, 16.2 mmol, 0.10 *eq.*). The mixture was stirred at 110 °C for 3 hours, then cooled to 25 °C and poured into water (500 mL). The aqueous phase was extracted with ethyl acetate (100 mL × 3). The combined organic phases were washed with brine (1000 mL × 3), dried over anhydrous sodium sulfate, filtered, and concentrated under vacuum to give a residue. The residue was purified by column chromatography (SiO_2, petroleum ether / ethyl acetate = 100/1 to 5/1) to give *tert*-butyl (*R*)-(1-(3-cyano-2-methylphenyl)ethyl)carbamate (37.0 g, 142.1 mmol, 87.6% yield) as a white solid. LCMS $[M-55]^+$: 205.0.

^{1}H NMR (400 MHz, CD_3OD) δ = 7.63 (d, *J* = 7.6 Hz, 1H), 7.54 (d, *J* = 7.2 Hz, 1H), 7.39 - 7.30 (m, 1H), 4.93 (br d, *J* = 6.8 Hz, 1H), 2.58 (s, 3H), 1.40 (br s, 9H), 1.34 (d, *J* = 7.2 Hz, 3H).

Step H: To a solution of *tert*-butyl (*R*)-(1-(3-cyano-2-methylphenyl)ethyl)carbamate (49.0 g, 188 mmol, 1.00 *eq.*) in dichloromethane (400 mL) was added TFA (133 mL). The mixture was stirred at 0 °C for 30 minutes then poured into saturated sodium bicarbonate solution (200 mL) and stirred for and additional 30 minutes. The aqueous phase was extracted with ethyl acetate (1000 mL × 3). The combined organic phases were washed with brine (200 mL × 3), dried over anhydrous sodium sulfate, filtered, and concentrated under vacuum to give (R)-3-(1-aminoethyl)-2-methylbenzonitrile (26.0 g, 162 mmol, 86.2% yield) as yellow oil. LCMS $[M-16]^+$: 144.1.

^{1}H NMR (400 MHz, DMSO-d_6) δ = 8.36 (br s, 2H), 7.86 (d, *J* = 8.0 Hz, 1H), 7.80 (dd, *J* = 0.8, 7.6 Hz, 1H), 7.51 (t, *J* = 8.0 Hz, 1H), 4.68 (q, *J* = 6.8 Hz, 1H), 2.55 (s, 3H), 1.48 (d, *J* = 6.8 Hz, 3H).

SFC conditions: Column: Chiralpak IC-3 50 × 4.6 mm I.D., 3 µm Mobile phase: Phase A for CO_2, and Phase B for MeOH (0.05% DEA); Gradient elution: MeOH (0.05% DEA) in CO_2 from 5% to 40% Flow rate: 3 mL/min; Detector: PDA Column Temp: 35 °C; Back Pressure: 100Bar.

(*R*)-*N*-(1-(3-bromo-2-methylphenyl)ethyl)-4-methyl-7-morpholinopyrido[3,4-*d*]pyridazin-1-amine (31)

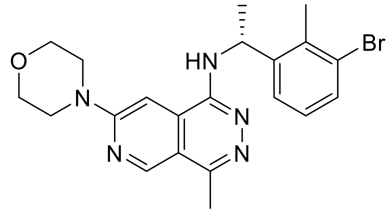

Compound 31

The following compound was made using **General Procedure B:** Yellow solid. ^{1}H NMR (400 MHz, CD_3OD) δ = 9.01 (s, 1H), 8.52 (s, 1H), 7.42 - 7.34 (m, 3H), 6.97 (t, *J* = 8.0 Hz, 1H), 5.50 (q, *J* = 6.8 Hz, 1H), 3.80 (s, 8H), 2.64 (s, 3H), 2.53 (s, 3H), 1.59 (d, *J* = 7.2 Hz, 3H). ^{13}C NMR (101 MHz, CD_3OD) δ =169.95, 162.24, 153.54, 152.16, 149.73, 146.76, 136.42, 132.22, 128.56, 128.11, 126.96, 125.04, 114.92, 94.70, 67.74, 46.60, 21.71, 18.98, 16.53. HRMS (m/ z): [M + H]+ calcd for $C_{21}H_{24}BrN_5O$, 442.1164; found, 442.1244. HPLC (A: 0.0375% TFA in water, B: 0.01875% TFA in Acetonitrile): t_R = 4.385 min (95.4% purity).

(*R*)-2-methyl-3-(1-((4-methyl-7-morpholinopyrido[3,4-*d*]pyridazin-1-yl)amino)ethyl)benzonitrile (32, MRTX0902)

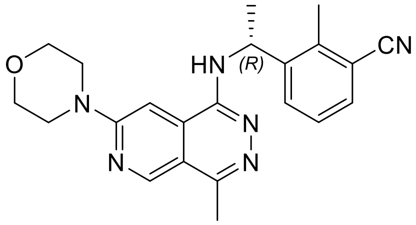

MRTX0902
(Compound 32)

Made using **General Procedure B** to give **MRTX0902 (32)**: Yellow solid. ^{1}H NMR (400 MHz, DMSO-d_6) δ = 8.96 (s, 1H), 7.71 (d, J = 8.0 Hz, 1H), 7.63 - 7.48 (m, 2H), 7.38 (s, 1H), 7.30 (t, J = 8.0 Hz, 1H), 5.54 - 5.49 (m, 1H), 3.80 - 3.71 (m, 4H), 3.67 - 3.65 (m, 4H), 2.62 (s, 3H), 2.54 (s, 3H), 1.52 (d, J = 7.2 Hz, 3H). ^{13}C NMR (101 MHz, DMSO-d_6): δ ppm 159.79, 151.20, 149.42, 147.56, 146.39, 139.20, 131.25, 129.71, 127.41, 125.10, 119.01, 114.44, 112.69, 93.62, 66.30, 47.06, 45.49, 21.86, 18.47, 17.20. HRMS (m/ z): [M + H]+ calcd for $C_{22}H_{24}N_6O$, 389.2012; found, 389.2105. HPLC (0.025% $NH_3 \cdot H_2O$ in water): t_R = 11.621 min (98.9% purity).

Synthesis of (*R*)-1-(2-methyl-3-(methylsulfonyl)phenyl)ethan-1-amine

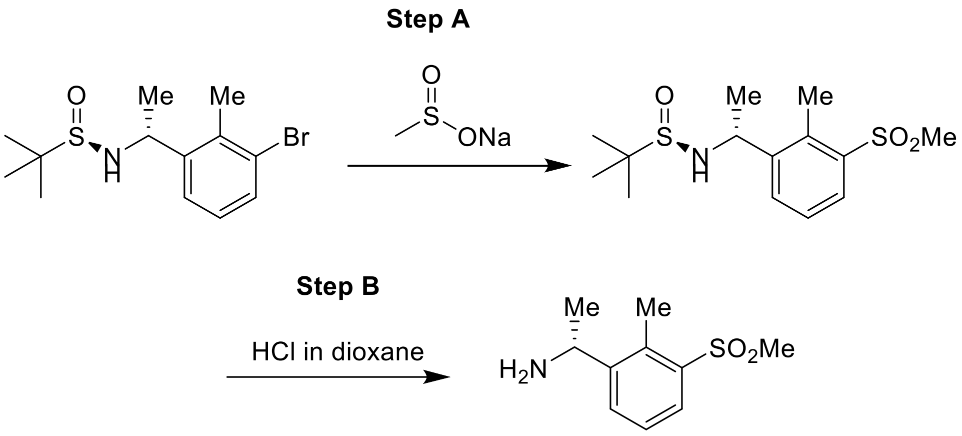

Step A: To a mixture of (*R*)-*N*-((*R*)-1-(3-bromo-2-methylphenyl)ethyl)-2-methylpropane-2-sulfinamide (250 mg, 786 µmol, 1.00 *eq*.), sodium methanesulfinate (176 mg, 1.73 mmol, 2.20 *eq*.), potassium carbonate (326 mg, 2.36 mmol, 3.00 *eq*.) and *L*-proline (18.1 mg, 157 µmol, 0.20 *eq*.) in dimethyl sulfoxide (3.00 mL) was added copper (I) iodide (15.0 mg, 78.6 µmol, 0.10 *eq*.) at 20 °C, the mixture was stirred at 130 °C for 3 hours under a nitrogen atmosphere. To the mixture was added water (15.0 mL), and the mixture was extracted with ethyl acetate (20.0 mL × 3). The combined organic phases were washed with brine (30.0 mL × 3), dried over anhydrous sodium sulfate, filtered, and concentrated under reduced pressure to give a residue. The residue was purified by prep-TLC (silica gel plate, petroleum ether / ethyl acetate = 1/1) to give (*R*)-2-methyl-*N*-((*R*)-1-(2-methyl-3-(methylsulfonyl)phenyl)ethyl)propane-2-sulfinamide (120 mg, 378 µmol, 48.1% yield) as a yellow oil. LCMS [M+1] $^{+}$: 318.1.

^{1}H NMR (400 MHz, DMSO-d_6) δ = 7.85 (dd, J = 8.0, 1.2 Hz, 1H), 7.78 (d, J = 7.6 Hz, 1H), 7.46 (t, J = 8.0 Hz, 1 H), 5.42-5.50 (m, 1H), 4.71-4.80 (m, 1H), 3.22 (s, 3H), 2.65 (s, 3H), 1.46 (d, J = 6.8 Hz, 3 H), 1.09 (s, 9H).

Step B: A mixture of (*R*)-2-methyl-*N*-((*R*)-1-(2-methyl-3-(methylsulfonyl)phenyl)ethyl)propane-2-sulfinamide (120 mg, 378 µmol, 1.00 *eq.*) in hydrochloric acid (4.0 M in dioxane, 2.00 mL, 21.2 *eq.*) was stirred at 20 °C for 1 hour. The mixture was concentrated under reduced pressure to give (*R*)-1-(2-methyl-3-(methylsulfonyl)phenyl)ethan-1-amine (91.0 mg, crude, HCl) as a white solid.

(*R*)-4-methyl-*N*-(1-(2-methyl-3-(methylsulfonyl)phenyl)ethyl)-7-morpholinopyrido[3,4-*d*]pyridazin-1-amine (33)

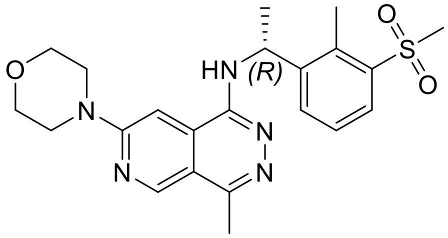

Compound 33

The following compound was made using **General Procedure B:** Yellow solid. ^{1}H NMR (400 MHz, DMSO-d_6) δ = 8.99 (s, 1H), 8.16 (s, 1H), 7.82 - 7.76 (m, 2H), 7.69 - 7.48 (m, 1H), 7.42 (s, 1H), 7.38 (t, J = 8.0, 1H), 5.64 (q, J = 6.8 Hz, 1H), 3.77 (br d, J = 4.4 Hz, 4H), 3.69 (br d, J = 4.4 Hz, 4H), 3.25 (s, 3H), 2.80 (s, 3H), 2.56 (s, 3H), 1.55 (br d, J = 6.8 Hz, 3H). ^{13}C NMR (101 MHz, DMSO-d_6) δ =159.87, 151.26, 149.60, 147.63, 147.60, 140.02, 134.97, 130.26, 127.19, 126.82, 125.25, 114.38, 93.68, 66.31, 46.82, 45.51, 44.12, 22.05, 18.30, 15.10. HRMS (m/ z): [M + H]+ calcd for $C_{22}H_{27}N_5O_3S$, 442.1835; found, 442.1926. HPLC (0.025% $NH_3{\cdot}H_2O$ in water): t_R =10.458 min (98.9% purity).

(*R*)-3-(1-((4-methyl-7-morpholinopyrido[3,4-*d*]pyridazin-1-yl)amino)ethyl)benzonitrile (34)

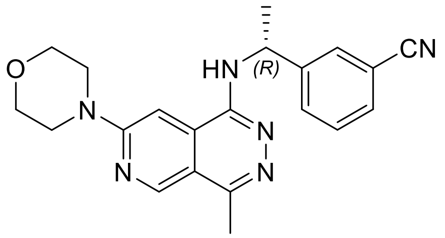

Compound 34

The following compound was made using **General Procedure C:** Yellow solid. ^{1}H NMR (400 MHz, CD_3OD) δ = 9.07 (s, 1H), 8.48 (s, 1H), 7.78 (s, 1H), 7.75 (br d, *J* = 8.0 Hz, 1H), 7.58 - 7.51 (m, 1H), 7.50 - 7.44 (m, 1H), 7.39 (s, 1H), 5.31 (q, *J* = 6.8 Hz, 1H), 3.83 (s, 8H), 2.70 (s, 3H), 1.67 (d, *J* = 7.2 Hz, 3H). ^{13}C NMR (101 MHz, CD_3OD) δ = 167.81, 160.90, 152.29, 151.19, 148.67, 146.35, 130.90, 130.36, 129.76, 129.21, 126.85, 118.39, 113.13, 111.88, 93.24, 66.19, 50.67, 45.07, 21.21, 14.62. HRMS (m/ z): [M + H]+ calcd for $C_{21}H_{22}N_6O$, 375.1855; found, 375.1970. HPLC (0.025% $NH_3{\cdot}H_2O$ in water): t_R = 10.878 min (99.4% purity).

(*R*)-3-(1-((6-fluoro-4-methyl-7-morpholinophthalazin-1-yl)amino)ethyl)-2-methylbenzonitrile (35)

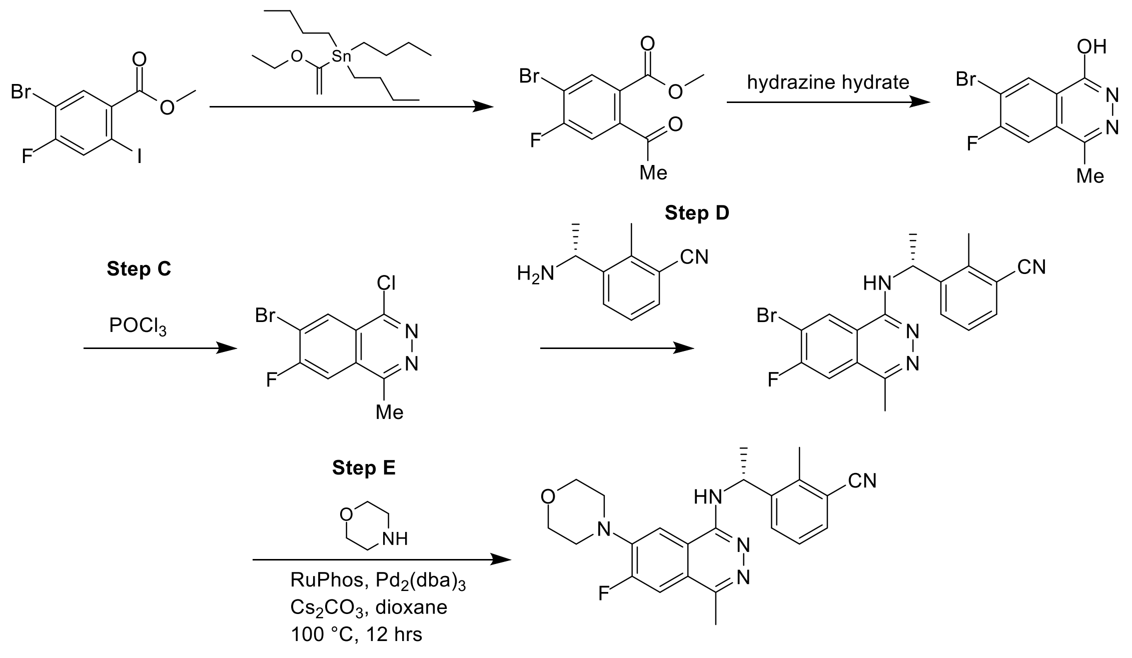

Compound 35

Step A: To a solution of methyl 5-bromo-4-fluoro-2-iodobenzoate (1.50 g, 4.18 mmol, 1.00 *eq.*) and tributyl(1-ethoxyvinyl)tin (1.52 g, 4.22 mmol, 1.42 mL, 1.01 *eq.*) in dioxane (20.0 mL) was added $Pd(PPh_3)_2Cl_2$ (60.0 mg, 0.08 mmol, 0.02 *eq.*) under a nitrogen atmosphere. The reaction mixture was stirred at 80 °C for 12 hours under a nitrogen atmosphere. The reaction mixture was cooled to 25 °C, quenched by addition of saturated aqueous potassium fluoride (100 mL**)** and extracted with ethyl acetate (200 mL × 3). The combined organic layers were washed with brine (200 mL × 3), dried over sodium sulfate, filtered, and concentrated under reduced pressure to give compound methyl 5-bromo-2-(1-ethoxyvinyl)-4-fluorobenzoate (2.00 g, crude) as a brown oil which was used in next step directly.

To a solution of methyl 5-bromo-2-(1-ethoxyvinyl)-4-fluorobenzoate (2.00 g, crude) in THF (50.0 mL) was added hydrochloric acid aqueous solution (4.00 M, 10.0 mL, 6.06 *eq*). The mixture was stirred at 25 °C for 2 hours, then diluted with water (50.0 mL**)** and extracted with ethyl acetate (50.0 mL × 3). The combined organic layers were washed with brine (20.0 mL), dried over sodium sulfate, filtered, and concentrated under reduced pressure to give a residue. The residue was purified by column chromatography (petroleum ether/ethyl acetate = 10/1 to 1/1) to give compound methyl 2-acetyl-5-bromo-4-fluorobenzoate (700 mg, 2.54 mmol, 38.6% yield) as a yellow oil.

^{1}H NMR (400 MHz, $CDCl_3$) δ = 8.14 (d, *J* = 6.4 Hz, 1H), 7.12 (d, *J* = 8.0 Hz, 1H), 3.91 (s, 3H), 2.52 (s, 3H).

Step B: To a solution of methyl 2-acetyl-5-bromo-4-fluorobenzoate (700 mg, 2.54 mmol, 1.00 *eq.*) in ethanol (10.0 mL) was added hydrazine hydrate (130 mg, 2.54 mmol, 98% purity, 1.00 *eq.*) dropwise. The reaction mixture was stirred at 95 °C for 30 minutes, then cooled to 25 °C and concentrated under reduced pressure to give 7-bromo-6-fluoro-4-methylphthalazin-1-ol (460 mg, 1.79 mmol, 70.3% yield) as a white solid.

^{1}H NMR (400 MHz, $CDCl_3$) δ = 12.62 (s, 1H), 8.46 (d, *J* = 7.2 Hz, 1H), 7.94 (d, *J* = 9.6 Hz, 1H), 2.48 (s, 3H).

Step C: A mixture of 7-bromo-6-fluoro-4-methylphthalazin-1-ol (250 mg, 0.97 mmol, 1.00 *eq.*) in phosphorus (V) oxychloride (9.52 g, 62.1 mmol, 5.77 mL, 63.8 *eq.*) was stirred at 110 °C for 2 hours. The reaction mixture was cooled to 25 °C and concentrated under reduced pressure to give

a residue. The residue was diluted with ethyl acetate (30.0 mL) and the pH was adjusted to pH=7 by slow addition of saturated sodium bicarbonate (aqueous solution). The organic phase was washed with brine (20.0 mL × 2), dried over anhydrous sodium sulfate, filtered, and concentrated under reduced pressure. The crude product was purified by prep-TLC (petroleum ether/ethyl acetate = 3/1) to give 6-bromo-4-chloro-7-fluoro-1-methylphthalazine (170 mg, 617 µmol, 63.5% yield) as a yellow solid. LCMS $[M+3]^+$: 276.7.

Step D: A mixture of 6-bromo-4-chloro-7-fluoro-1-methylphthalazine (100 mg, 0.36 mmol, 1.00 *eq.*), (*R*)-3-(1-aminoethyl)-2-methylbenzonitrile (58.0 mg, 0.36 mmol, 1.00 *eq.*), potassium fluoride (21.0 mg, 0.36 mmol, 8.50 µL, 1.00 *eq.*) in dimethyl sulfoxide (3.00 mL) was degassed and purged with nitrogen 3 times, and then the mixture was stirred at 130 °C for 12 hours under a nitrogen atmosphere. The reaction mixture was cooled to 25 °C and water (20.0 mL) was added, then the aqueous solution was extracted with ethyl acetate (20.0 mL × 3). The combined organic phases were washed with brine (20.0 mL × 3), dried over anhydrous sodium sulfate, filtered, and concentrated under reduced pressure to give a residue. The residue was purified by prep-TLC (silica gel plate, dichloromethane/methyl alcohol = 20/1) to give (*R*)-3-(1-((7-bromo-6-fluoro-4-methylphthalazin-1-yl)amino)ethyl)-2-methylbenzonitrile (60.0 mg, 0.09 mmol, 24.8% yield, 60.0% purity) as a white solid. LCMS $[M+1]^+$ = 399.0.

Step E: A mixture of (*R*)-3-(1-((7-bromo-6-fluoro-4-methylphthalazin-1-yl)amino)ethyl)-2-methylbenzonitrile (100 mg, 250 µmol, 1.00 *eq.*), morpholine (43.6 mg, 501 µmol, 44.1 µL, 2.00 *eq.*), $Pd_2(dba)_3$ (22.9 mg, 25.0 µmol, 0.10 *eq.*), RuPhos (23.4 mg, 50.1 µmol, 0.20 *eq.*) and cesium carbonate (408 mg, 1.25 mmol, 5.00 *eq.*) in dioxane (2.00 mL) was degassed and purged with nitrogen 3 times, and then the mixture was stirred at 100 °C for 12 hours under a nitrogen atmosphere. The reaction mixture was cooled to room temperature, then ethyl acetate (10.0 mL) and water (10.0 mL) were added and the layers were separated. The aqueous phase was extracted with ethyl acetate (10.0 mL × 3). The combined organic phases were washed with brine (10.0 mL), dried over anhydrous sodium sulfate, filtered, and concentrated under reduced pressure to give a residue was purified by prep-HPLC (column: Waters Xbridge 150 × 25mm × 5um; mobile phase A: water(10 mM NH_4HCO_3), mobile phase B: acetonitrile; B%: 40%-70%) to give (*R*)-3-(1-((6-fluoro-4-methyl-7-morpholinophthalazin-1-yl)amino)ethyl)-2-methylbenzonitrile (14.9 mg, 36.7 µmol, 14.7% yield) as a white solid.

^{1}H NMR (400 MHz, CD_3OD) δ = 7.78 (d, *J* = 8.0 Hz, 1H), 7.72 (d, *J* = 7.6 Hz, 1H), 7.58 (d, *J* = 13.2 Hz, 1H), 7.43 (d, *J* = 7.2 Hz, 1H), 7.19 (t, *J* = 7.6 Hz 1H), 5.59 (q, *J* = 6.8 Hz, 1H), 3.85 (t, *J* = 4.4 Hz, 4H), 3.28 - 3.19 (m, 4H), 2.67 (s, 3H), 2.56 (s, 3H), 1.59 (d, *J* = 6.8 Hz, 3H). ^{13}C NMR (101 MHz, CD_3OD) δ = 160.28, 157.76, 153.52, 149.45 (d, *J* = 4.1 Hz, 1C), 147.33, 145.96 (d, *J* = 9.8 Hz, 1C), 140.81, 132.06, 130.50, 127.89, 124.73 (d, *J* = 9.8 Hz, 1C), 119.72, 118.09 (d, *J* = 1.6 Hz, 1C), 114.24, 112.10 (d, *J* = 28.8 Hz, 1C), 112.01 (d, *J* = 1.6 Hz, 1C), 67.97, 52.04 (d, *J* = 4.1 Hz, 1C), 21.67, 18.76, 17.50 HRMS (m/ z): [M + H]+ calcd for $C_{23}H_{24}FN_5O$, 406.1965; found, 406.2056. HPLC (A: 0.0375% TFA in water, B: 0.01875% TFA in Acetonitrile): t_R = 4.088 min (99.4% purity).

(*R*)-3-(1-((6-chloro-4-methyl-7-morpholinophthalazin-1-yl)amino)ethyl)-2-methylbenzonitrile (36)

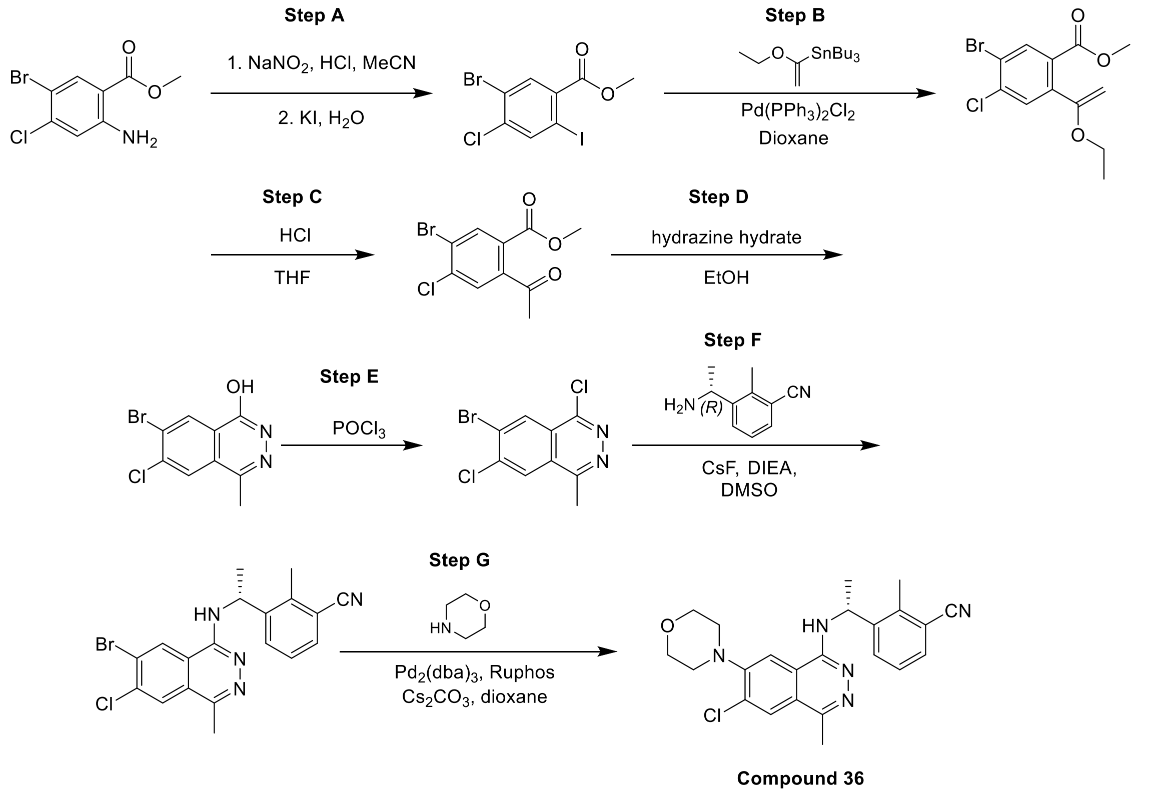

Step A: Methyl 2-amino-5-bromo-4-chloro-benzoate (9.00 g, 34.0 mmol, 1.00 *eq.*) was dissolved in a mixture of acetonitrile (60.0 mL) and hydrochloric acid in water (6 M, 170 mL, 30.0 *eq.*). The mixture was cooled to 0 °C, and a solution of sodium nitrite (3.76 g, 54.4 mmol, 1.60 *eq.*) in water

(15.0 mL) was added dropwise to the mixture at 0 °C. After stirring for 1 hour at 0 °C, potassium iodide (10.2 g, 61.3 mmol, 1.80 *eq.*) in water (15.0 mL) was added dropwise to the mixture at 0 °C, and the mixture was slowly warmed to 20 °C and stirred for an additional 12 hours. The reaction mixture was extracted with ethyl acetate (100 mL × 3), and the combined organic phases were dried over sodium sulfate, filtered, and concentrated under reduced pressure to give a residue. The residue was purified by column chromatography (SiO_2, Petroleum ether/Ethyl acetate = 50/1) to give methyl 5-bromo-4-chloro-2-iodobenzoate (11.0 g, 29.3 mmol, 86.1% yield) as a yellow solid.

^{1}H NMR (400 MHz, $CDCl_3$) δ = 8.09 (s, 1H), 8.08 (s, 1H), 3.95 (s, 3H).

Step B: A mixture of methyl 5-bromo-4-chloro-2-iodobenzoate (11.0 g, 29.3 mmol, 1.00 *eq.*), tributyl(1-ethoxyvinyl)stannane (11.6 g, 32.2 mmol, 10.9 mL, 1.10 *eq.*) and $Pd(PPh_3)_2Cl_2$ (2.06 g, 2.93 mmol, 0.10 *eq.*) in dioxane (100 mL) was degassed and purged with nitrogen 3 times, and then the mixture was stirred at 80 °C for 12 hours under a nitrogen atmosphere. The mixture was cooled to 25 °C, poured into saturated potassium fluoride aqueous solution (200 mL) and stirred for 1 hour. The aqueous phase was extracted with ethyl acetate (100 mL × 3), and the combined organic phases were washed with brine (50.0 mL × 2), dried over anhydrous sulfate, filtered, and concentrated under reduced pressure to give a crude product methyl 5-bromo-4-chloro-2-(1-ethoxyvinyl)benzoate (10.0 g, crude) as red oil which was used in the next step directly.

Step C: To a solution of methyl 5-bromo-4-chloro-2-(1-ethoxyvinyl)benzoate (10.0 g, 31.3 mmol, 1.00 *eq.*) in THF (10.0 mL) was added hydrochloric acid (4 M in THF, 15.7 mL, 2.00 *eq.*), and the mixture was stirred at 20 °C for 30 minutes. After this time, the reaction mixture was adjusted to pH=7 with saturated sodium bicarbonate aqueous solution and extracted with ethyl acetate (50.0 mL × 2). The combined organic phases were dried over sodium sulfate, filtered, and concentrated under reduced pressure to give a residue. The residue was purified by column chromatography (SiO_2, petroleum ether/ethyl acetate=30/1 to 20/1) to give methyl 2-acetyl-5-bromo-4-chlorobenzoate (7.20 g, 24.7 mmol, 78.9% yield) as a light-yellow solid.

^{1}H NMR (400 MHz, $CDCl_3$) δ = 8.14 (s, 1H), 7.47 (s, 1H), 3.92 (s, 3H), 2.52 (s, 3H).

Step D: To a solution of methyl 2-acetyl-5-bromo-4-chlorobenzoate (4.00 g, 13.7 mmol, 1.00 *eq.*) in ethanol (40.0 mL) was added hydrazine hydrate (701 mg, 13.7 mmol, 680 μL, 98% purity, 1.00 *eq.*) dropwise at 25 °C, then the mixture was stirred at 80 °C for 4 hours. The reaction

mixture was cooled to 10 °C and filtered, then the filter cake was dried under vacuum to give 7-bromo-6-chloro-4-methylphthalazin-1-ol (3.40 g, 12.4 mmol, 90.6% yield) as a white solid.

^{1}H NMR (400 MHz, DMSO-d_6) δ = 12.65 (br s, 1H), 8.46 (s, 1H), 8.19 (s, 1H), 2.50 (s, 3H).

Step E: A mixture of 7-bromo-6-chloro-4-methylphthalazin-1-ol (3.10 g, 11.3 mmol, 1.00 *eq.*) in phosphorus oxychloride (30.0 mL) was stirred at 110 °C for 1 hour. The reaction mixture was then cooled to room temperature and poured into sodium bicarbonate aqueous solution to adjust pH = 7, the resulting mixture was extracted with ethyl acetate (50.0 mL × 3), and the combined organic phases were dried over sodium sulfate, filtered, and concentrated under reduced pressure to give a residue. The residue was purified by column chromatography (SiO_2, Petroleum ether/Ethyl acetate = 5/1 to 3/1) to give 6-bromo-4,7-dichloro-1-methylphthalazine (2.70 g, 9.25 mmol, 81.6% yield) as a yellow solid. LCMS $[M+3]^+$: 293.0.

Step F: To a solution of 6-bromo-4,7-dichloro-1-methylphthalazine (3.10 g, 10.6 mmol, 1.00 *eq.*) and (*R*)-3-(1-aminoethyl)-2-methylbenzonitrile (2.30 g, 11.7 mmol, 1.10 *eq.*, HCl salt) in DMSO (25.0 mL) was added cesium fluoride (2.42 g, 15.9 mmol, 587 μL, 1.50 *eq.*) and *N,N*-diisopropylethylamine (2.74 g, 21.2 mmol, 3.70 mL, 2.00 *eq.*), the mixture was stirred at 130 °C for 4 hours. The reaction mixture was cooled to 25 °C, diluted with water (100 mL) and filtered, the filter cake was dried under vacuum to give a residue. The residue was purified by column chromatography (SiO_2, Petroleum ether/Ethyl acetate=5/1 to 3/1) to give (*R*)-3-(1-((7-bromo-6-chloro-4-methylphthalazin-1-yl)amino)ethyl)-2-methylbenzonitrile (3.10 g, 7.46 mmol, 70.2% yield) as a pale red solid.

^{1}H NMR (400 MHz, $CDCl_3$) δ = 8.15 (s, 1H), 8.03 (s, 1H), 7.65 (d, J = 8.0 Hz, 1H), 7.50 (dd, J = 1.2, 8.0 Hz, 1H), 7.26 - 7.21 (m, 1H), 5.78 - 5.71 (m, 1H), 5.21 (br s, 1H), 2.76 (s, 3H), 2.72 (s, 3H), 1.65 (d, J = 6.8 Hz, 3H). LCMS $[M+3]^+$: 417.1.

Step G**:** A mixture of (*R*)-3-(1-((7-bromo-6-chloro-4-methylphthalazin-1-yl)amino)ethyl)-2-methylbenzonitrile (150 mg, 361 μmol, 1.00 *eq.*), morpholine (34.6 mg, 396.91 μmol, 34.9 μL, 1.10 *eq.*), cesium carbonate (353 mg, 1.08 mmol, 3.00 *eq.*), RuPhos (33.7 mg, 72.2 μmol, 0.20 *eq.*) and $Pd_2(dba)_3$ (33.0 mg, 36.1 μmol, 0.10 *eq.*) in dioxane (2.00 mL) was degassed and purged with nitrogen 3 times, and then the mixture was stirred at 80 °C for 2 hours under a nitrogen atmosphere. The reaction mixture was cooled to 25 °C, filtered, and the filtrate was concentrated under reduced pressure to give a residue. The residue was purified by prep-TLC

(SiO_2, Petroleum ether/Ethyl acetate= 1/3) and then by prep-HPLC (column: Phenomenex Luna C18 150 × 25mm × 10um; mobile phase A: 0.225% formic acid in water, mobile phase B: acetonitrile; B%: 11%-41%,) to give (*R*)-3-(1-((6-chloro-4-methyl-7-morpholinophthalazin-1-yl)amino)ethyl)-2-methylbenzonitrile (22.5 mg, 52.9 μmol, 44.6% yield, 99.2% purity) as a white solid.

^{1}H NMR (400 MHz, DMSO-d_6) δ = 8.01 (br s, 1H), 7.99 (br s, 1H), 7.76 (br d, *J* = 7.6 Hz, 1H), 7.70 (br d, *J* = 6.0 Hz, 1H), 7.59 (br d, *J* = 7.6 Hz, 1H), 7.30 (br t, *J* = 7.6 Hz, 1H), 5.56 (br t, *J* = 6.4 Hz, 1H), 3.82 (br s, 4H), 3.16 (br s, 4H), 2.65 (s, 3H), 2.56 (s, 3H), 1.55 (br d, *J* = 6.8 Hz, 3H). ^{13}C NMR (101 MHz, DMSO-d_6) δ =151.85, 151.68, 147.38, 146.61, 139.16, 132.65, 131.17, 129.77, 127.36, 127.12, 123.46, 119.03, 117.95, 113.54, 112.68, 66.01, 52.10, 47.22, 21.91, 19.21, 17.21. HRMS (m/ z): [M + H]+ calcd for $C_{23}H_{24}ClN_5O$, 422.1699; found, 422.1766. HPLC (A: 0.0375% TFA in water, B: 0.01875% TFA in Acetonitrile): t_R = 4.177 min (95.5% purity).

(*R*)-1-((1-(3-cyano-2-methylphenyl)ethyl)amino)-4-methyl-7-morpholinophthalazine-6-carbonitrile (37)

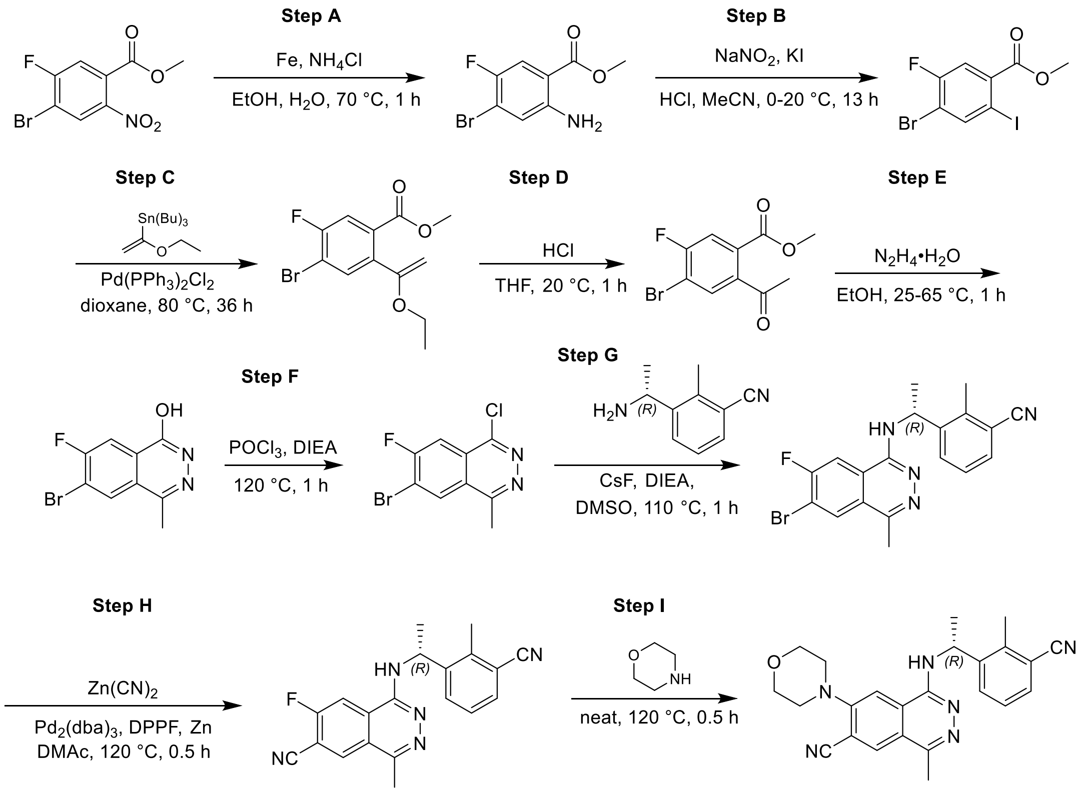

Compound 37

Step A: To a solution of methyl 4-bromo-5-fluoro-2-nitrobenzoate (15.0 g, 54.0 mmol, 1.00 *eq.*) in ethanol (180 mL) and water (60.0 mL) was added iron powder (9.04 g, 162 mmol, 3.00 *eq.*) and ammonium chloride (23.1 g, 432 mmol, 8.00 *eq.*), the mixture was stirred at 70 °C for 1 hour. The reaction mixture was cooled to 25 °C, filtered, and concentrated under reduced pressure to remove ethanol, and the resulting mixture was filtered. The precipitate was triturated with water (100 mL), filtered, and the filter cake was dried under vacuum to give a residue. The residue was purified by column chromatography (SiO_2, Petroleum ether/Ethyl acetate = 20/1) to give methyl 2-amino-4-bromo-5-fluorobenzoate (9.50 g, 38.3 mmol, 71.0% yield) as a pale red solid.

^{1}H NMR (400 MHz, $CDCl_3$) δ 7.59 (d, J = 9.6 Hz, 1H), 6.89 (d, J = 5.6 Hz, 1H), 3.88 (s, 3H)

LCMS $[M+1]^+$: 247.8.

Step B: methyl 2-amino-4-bromo-5-fluorobenzoate (2.30 g, 9.27 mmol, 1.00 *eq.*) was dissolved in acetonitrile (16.0 mL) and hydrochloric acid in water (6 M, 46.6 mL, 30.1 *eq.*), then the mixture was cooled to 0 °C, and a solution of sodium nitrite (960 mg, 13.9 mmol, 1.50 *eq.*) in water (10.0 mL) was added to the mixture dropwise at 0 °C. Solution was stirred for 1 hour, then a solution of potassium iodide (3.08 g, 18.5 mmol, 2.00 *eq.*) in water (10.0 mL) was added to the reaction mixture, and the mixture was slowly warmed to 20 °C and stirred for 12 hours. The reaction mixture was then extracted with ethyl acetate (50.0 mL × 2), and the combined organic phases were dried over sodium sulfate, filtered, and concentrated under reduced pressure to give a residue. The residue was purified by column chromatography (SiO_2, Petroleum ether/Ethyl acetate = 20/1 to 10/1) to give methyl 4-bromo-5-fluoro-2-iodobenzoate (3.10 g, 8.64 mmol, 93.2% yield) as a red solid.

^{1}H NMR (400 MHz, $CDCl_3$) δ 8.21 (d, J = 6.8 Hz, 1H), 7.63 (d, J = 8.8 Hz, 1H), 3.95 (s, 3H).

Step C: A mixture of methyl 4-bromo-5-fluoro-2-iodobenzoate (9.20 g, 25.6 mmol, 1.00 *eq.*), tributyl(1-ethoxyvinyl)stannane (10.2 g, 28.2 mmol, 9.52 mL, 1.10 *eq.*) and $Pd(PPh_3)_2Cl_2$ (1.80 g, 2.56 mmol, 0.10 *eq.*) in dioxane (100 mL) was degassed and purged with nitrogen 3 times, and then the mixture was stirred at 80 °C for 36 hours under a nitrogen atmosphere. The mixture was cooled to 25 °C, poured into saturated potassium fluoride aqueous solution (200 mL) and stirred for 1 hour. The aqueous phase was then extracted with ethyl acetate (100 mL × 3). The combined organic phases were washed with brine (100 mL × 2), dried

over anhydrous sodium sulfate, filtered, and concentrated under reduced pressure to give the crude product methyl 4-bromo-2-(1-ethoxyvinyl)-5-fluorobenzoate (9.00 g, crude) as red oil which was used directly in the next step.

Step D: To a solution of methyl 4-bromo-2-(1-ethoxyvinyl)-5-fluorobenzoate (9.00 g, 29.7 mmol, 1.00 *eq.*) in THF (40.0 mL) was added hydrochloric acid in water (3.0 M, 39.6 mL, 4.00 *eq.*) dropwise at 20 °C, and the mixture was left to stir at 20 °C for 1 hour. The reaction mixture was adjusted to pH = 7 with saturated sodium bicarbonate aqueous solution and extracted with ethyl acetate (80.0 mL × 2). The combined organic phases were dried over sodium sulfate, filtered, and concentrated under reduced pressure to give a residue. The residue was purified by column chromatography (SiO_2, Petroleum ether/Ethyl acetate = 10/1) to give methyl 2-acetyl-4-bromo-5-fluorobenzoate (6.60 g, 24.0 mmol, 80.8% yield) as yellow oil

^{1}H NMR (400 MHz, $CDCl_3$) δ 7.64 (d, J = 6.4 Hz, 1H), 7.59 (d, J = 8.4 Hz, 1H), 3.92 (s, 3H), 2.53 (s, 3H).

Step E: To a solution of methyl 2-acetyl-4-bromo-5-fluorobenzoate (7.00 g, 25.5 mmol, 1.00 *eq.*) in ethanol (50.0 mL) was added hydrazine hydrate (1.36 g, 26.7 mmol, 1.33 mL, 98% purity, 1.05 *eq.*) dropwise at 25 °C, and the mixture was then stirred at 65 °C for 1 hour. The reaction mixture was then cooled to 10 °C, filtered, the filter cake was dried under reduced pressure to give 6-bromo-7-fluoro-4-methylphthalazin-1-ol (5.20 g, 20.2 mmol, 79.5% yield) as a white solid.

^{1}H NMR (400 MHz, DMSO-d_6) δ 12.59 (s, 1H), 8.35 (d, J = 6.4 Hz, 1H), 8.04 (d, J = 8.8 Hz, 1H), 2.51 (s, 3H).

Step F: To a solution of 6-bromo-7-fluoro-4-methylphthalazin-1-ol (1.50 g, 5.84 mmol, 1.00 *eq.*) in $POCl_3$ (10.0 mL) was added *N,N*-diisopropyethylamine (2.26 g, 17.5 mmol, 3.05 mL, 3.00 *eq.*), and the mixture was stirred at 120 °C for 1 hr. The reaction mixture was then cooled to room temperature and adjusted to pH = 7 with saturated sodium bicarbonate aqueous solution. The resulting mixture was extracted with ethyl acetate (50.0 mL × 3), and the combined organic phases were dried over sodium sulfate, filtered, and concentrated under reduced pressure to give a residue. The residue was purified by column chromatography (SiO_2, Petroleum ether/Ethyl acetate = 2/1) to give 6-bromo-1-chloro-7-fluoro-4-methylphthalazine (1.00 g, 3.63 mmol, 62.2% yield) as a red solid. LCMS $[M+3]^+$: 277.0.

Step G: A mixture of 6-bromo-1-chloro-7-fluoro-4-methylphthalazine (0.90 g, 3.27 mmol, 1.00 *eq.*) and cesium fluoride (744 mg, 4.90 mmol, 181 µL, 1.50 *eq.*) in DMSO (8.00 mL) was stirred at 110 °C for 30 minutes. To this mixture was added (*R*)-3-(1-aminoethyl)-2-methylbenzonitrile (675 mg, 3.43 mmol, 1.05 *eq.*, HCl salt) and *N,N*-diisopropylethylamine (1.27 g, 9.80 mmol, 1.71 mL, 3.00 *eq.*) and the mixture was then stirred 110 °C for 30 minutes. The reaction mixture was then cooled to 25 °C, diluted with water (100 mL), filtered, and the filter cake was collected and dried under reduced pressure to give a residue. The residue was purified by column chromatography (SiO2, Petroleum ether/Ethyl acetate = 90/10 to 55/45) to give (*R*)-3-(1-((6-bromo-7-fluoro-4-methylphthalazin-1-yl)amino)ethyl)-2-methylbenzonitrile (0.30 g, 751 µmol, 23.0% yield) as a pale red solid.

^{1}H NMR (400 MHz, $CDCl_3$) δ = 8.22 (br d, *J* = 6.4 Hz, 1H), 7.92 - 7.75 (m, 1H), 7.70 (br d, *J* = 7.6 Hz, 1H), 7.47 (br d, *J* = 7.6 Hz, 1H), 7.25 - 7.14 (m, 1H), 5.91 - 5.57 (m, 2H), 2.79 (s, 3H), 2.68 (s, 3H), 1.65 (br d, *J* = 6.4 Hz, 3H). LCMS $[M+3]^+$: 401.1.

Step H: A mixture of (*R*)-3-(1-((6-bromo-7-fluoro-4-methylphthalazin-1-yl)amino)ethyl)-2-methylbenzonitrile (0.25 g, 626 µmol, 1 *eq.*), zinc cyanide (147 mg, 1.25 mmol, 79.5µL, 2.00 *eq.*), $Pd_2(dba)_3$ (57.3 mg, 62.6 µmol, 0.10 *eq.*), DPPF (69.4 mg, 125 µmol, 0.20 *eq.*) and Zn (81.9 mg, 1.25 mmol, 2.00 *eq.*) in dimethylacetamide (4.00 mL) was degassed and purged with nitrogen 3 times, and then the mixture was stirred at 120 °C for 30 minutes under a nitrogen atmosphere. The reaction mixture was cooled to 25 °C, diluted with ethyl acetate (50.0 mL) and filtered. The filtrate was washed with brine (30.0 mL × 3), dried over sodium sulfate, filtered, and concentrated under reduced pressure to give a residue. The residue was purified by column chromatography (SiO_2, petroleum ether/ethyl acetate=3/1 to 1/1) to give (*R*)-1-((1-(3-cyano-2-methylphenyl)ethyl)amino)-7-fluoro-4-methylphthalazine-6-carbonitrile (180 mg, 521 µmol, 83.2% yield) as a pale red solid. LCMS $[M+1]^+$: 346.2.

Step I: A mixture of (*R*)-1-((1-(3-cyano-2-methylphenyl)ethyl)amino)-7-fluoro-4-methylphthalazine-6-carbonitrile (50.0 mg, 145 µmol, 1.00 *eq.*) in morpholine (505 mg, 5.79 mmol, 510 µL, 40.0 *eq.*) was stirred at 120 °C for 30 minutes. The reaction mixture was cooled to 25 °C, diluted with water (30.0 mL), filtered, and the filter cake was dried under reduced pressure to give a residue. The residue was purified by prep-HPLC (Phenomenex Luna C18 150 × 25mm × 10um; mobile phase A: 0.225% formic acid in water, mobile phase B: acetonitrile; B%: 10%-

40%) to give (*R*)-1-((1-(3-cyano-2-methylphenyl)ethyl)amino)-4-methyl-7-morpholinophthalazine-6-carbonitrile (54.9 mg, 132 μmol, 91.3% yield, 99.3% purity) as a yellow solid.

^{1}H NMR (400 MHz, DMSO-d_6) δ = 8.46 (s, 1H), 7.91 (s, 1H), 7.81 - 7.72 (m, 2H), 7.59 (d, *J* = 7.6 Hz, 1H), 7.30 (t, *J* = 8.0 Hz, 1H), 5.55 (quin, *J* = 6.8 Hz, 1H), 3.87 - 3.80 (m, 4H), 3.33 - 3.28 (m, 4H), 2.64 (s, 3H), 2.58 (s, 3H), 1.55 (d, *J* = 6.8 Hz, 3H). ^{13}C NMR (101 MHz, DMSO-d_6) δ =155.28, 151.73, 147.73, 146.36, 139.20, 134.36, 131.23, 129.73, 127.38, 121.31, 121.13, 118.99, 117.76, 112.70, 111.02, 109.89, 66.46, 52.21, 47.38, 21.85, 19.14, 17.20. HRMS (m/ z): [M + H]+ calcd for $C_{24}H_{24}N_6O$, 413.2012; found, 413.2105. HPLC (A: 0.0375% TFA in water, B: 0.01875% TFA in Acetonitrile): t_R = 3.933 min (96.1% purity).

(*R*)-2-methyl-3-(1-((4-methyl-7-morpholino-6-(trifluoromethyl)phthalazin-1-yl)amino)ethyl)benzonitrile (38)

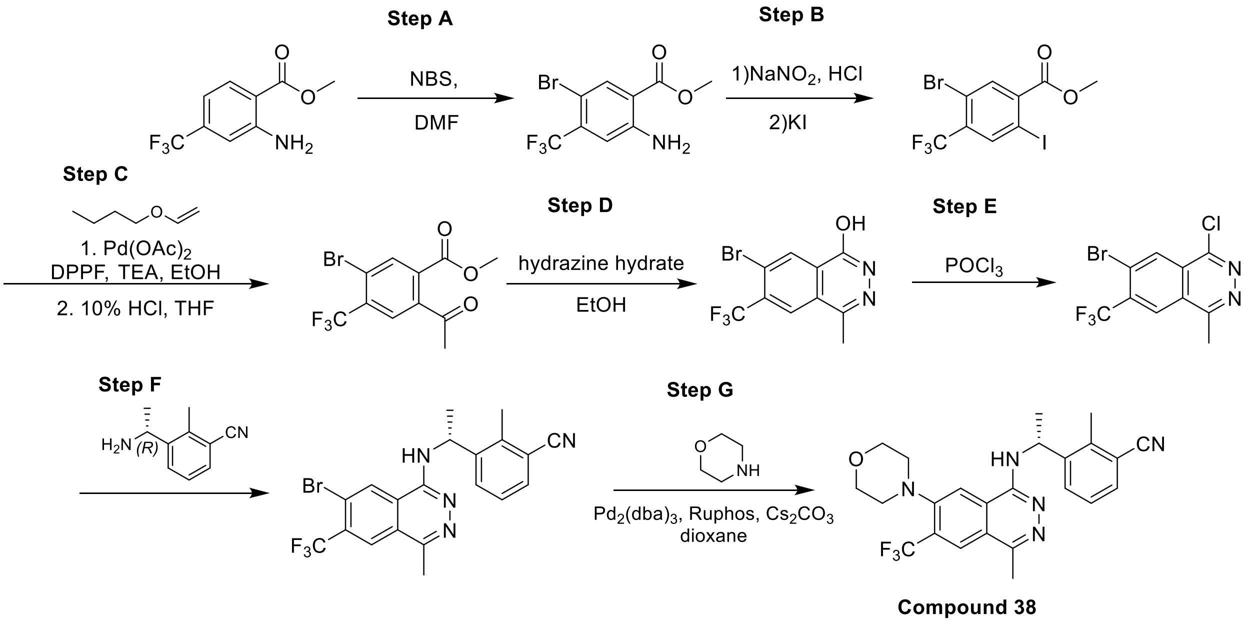

Step A: To a solution of methyl 2-amino-4-(trifluoromethyl)benzoate (3.00 g, 13.7 mmol, 1.00 *eq.*) in *N,N*-dimethylformamide (50.0 mL) was added NBS (2.68 g, 15.1 mmol, 1.10 *eq.*) under a nitrogen atmosphere. The reaction mixture was stirred at 20 °C for 12 hours under a nitrogen atmosphere, then poured into water (50.0 mL) and extracted with ethyl acetate (50.0 mL × 3). The

combined organic layers were washed with brine (40.0 mL), dried over sodium sulfate, filtered, and concentrated under reduced pressure to give a residue. The residue was purified by column chromatography (silica gel, petroleum ether/ethyl acetate = 50/1 to 10/1) to give methyl 2-amino-5-bromo-4-(trifluoromethyl)benzoate (3.30 g, 11.1 mmol, 80.9% yield) as yellow solid.

^{1}H NMR (400 MHz, DMSO-d_6): δ = 8.06 (s, 1H), 6.93 (s, 1H), 5.86 (s, 2H), 3.84 (s, 3H).

Step B: To a solution of methyl 2-amino-5-bromo-4-(trifluoromethyl)benzoate (3.30 g, 11.1 mmol, 1.00 *eq.*) in hydrochloric acid (4.00 M, 100 mL, 36.1 *eq.*) was added sodium nitrite (917 mg, 13.3 mmol, 1.20 *eq.*) at 0 °C and stirred for 1 hour under a nitrogen atmosphere. To this mixture was added potassium iodide (3.68 g, 22.1 mmol, 2.00 *eq.*) portionwise at 0 °C. The mixture was then heated to 90 °C and stirred for 11 hours under a nitrogen atmosphere. The reaction mixture was cooled 25 °C, extracted with ethyl acetate (100 mL × 3), and the combined organic layers were washed with brine (50.0 mL), dried over sodium sulfate, filtered, and concentrated under reduced pressure to give a residue. The residue was purified by column chromatography (silica gel, petroleum ether/ethyl acetate = 100/1 to 20/1) to give methyl 5-bromo-2-iodo-4-(trifluoromethyl)benzoate (4.10 g, 10.0 mmol, 90.5% yield) as a yellow solid.

^{1}H NMR (400 MHz, $CDCl_3$) δ = 8.16 (s, 2H), 8.01 (s, 1H), 3.90 (s, 3H).

Step C: To a solution of methyl 5-bromo-2-iodo-4-(trifluoromethyl)benzoate (3.60 g, 8.80 mmol, 1.00 *eq.*) and 1-(vinyloxy)butane (1.06 g, 10.6 mmol, 1.36 mL, 1.20 *eq.*) in *N,N*-dimethylformamide (10.0 mL) were added DPPF (244 mg, 440 µmol, 0.05 *eq.*), triethylamine (2.67 g, 26.4 mmol, 3.68 mL, 3.00 *eq.*) and $Pd(OAc)_2$ (59.3 mg, 264 µmol, 0.03 *eq.*) under a nitrogen atmosphere. The reaction mixture was stirred at 70 °C for 12 hours under a nitrogen atmosphere, then cooled to 25 °C, diluted with tetrahydrofuran (17.8 g, 247 mmol, 20.0 mL, 14.9 *eq.*), hydrochloric acid (4.00 M, 20.0 mL, 4.82 *eq.*), and stirred at 20 °C for 1 hour. The reaction mixture was poured into water (30.0 mL), and then extracted with ethyl acetate (30.0 mL × 3). The combined organic layers were washed with brine (30.0 mL), dried over sodium sulfate, filtered, and concentrated under reduced pressure to give a residue. The residue was purified by column chromatography (silica gel, petroleum ether/ethyl acetate = 50/1 to 10/1) to give methyl 2-acetyl-5-bromo-4-(trifluoromethyl)benzoate (250 mg, 769 µmol, 8.74% yield) as a yellow solid.

^{1}H NMR (400 MHz, $CDCl_3$) δ = 8.08 (s, 1H), 7.68 (s, 1H), 3.87 (s, 3H), 2.49 (s, 3H).

Step D: To a solution of methyl 2-acetyl-5-bromo-4-(trifluoromethyl)benzoate (250 mg, 769 µmol, 1.00 *eq.*) in ethanol (5.00 mL) was added hydrazine hydrate (46.2 mg, 923 µmol, 44.8 µL, 98%, 1.20 *eq.*) under a nitrogen atmosphere. The reaction mixture was stirred at 95 °C for 30 minutes under a nitrogen atmosphere. The reaction mixture was cooled to 25 °C, and concentrated under reduced pressure to give 7-bromo-4-methyl-6-(trifluoromethyl)phthalazin-1-ol (170 mg, crude) as a yellow solid which was used directly in the next step. LCMS $[M+3]^+$: 309.1.

Step E: A solution of 7-bromo-4-methyl-6-(trifluoromethyl)phthalazin-1-ol (50.0 mg, 162.8 µmol, 1.00 *eq.*) in $POCl_3$ (4.95 g, 32.3 mmol, 3.00 mL, 198 *eq.*) was stirred at 110 °C for 1 hour under a nitrogen atmosphere. The reaction mixture was then cooled to 25 °C, diluted with ethyl acetate (50.0 mL), and further diluted with saturated sodium bicarbonate aqueous solution (50.0 mL) at 25°C. The resulting mixture was extracted with ethyl acetate (50.0 mL × 3) and the combined organic layers were washed with brine (40.0 mL), dried over sodium sulfate, filtered, and concentrated under reduced pressure to give a residue. The residue was purified by prep-TLC (silica gel plate, petroleum ether/ethyl acetate = 1/1) to give 6-bromo-4-chloro-1-methyl-7-(trifluoromethyl)phthalazine (20.0 mg, 61.4 µmol, 37.7% yield) as a brown oil. LCMS $[M+3]^+$: 326.7.

Step F: To a solution of 6-bromo-4-chloro-1-methyl-7-(trifluoromethyl)phthalazine (120 mg, 369 µmol, 1.00 *eq.*) and (*R*)-3-(1-aminoethyl)-2-methylbenzonitrile (59.0 mg, 369 µmol, 1.00 *eq.*) in DMSO (3.00 mL) was added potassium fluoride (107 mg, 1.84 mmol, 43.2 µL, 5.00 *eq.*) and the mixture was stirred at 130 °C for 12 hours under a nitrogen atmosphere. The reaction mixture was cooled to 25 °C, poured into water (10.0 mL), and then extracted with ethyl acetate (10.0 mL × 3). The combined organic layers were washed with brine (10.0 mL), dried over sodium sulfate, filtered, and concentrated under reduced pressure to give a residue. The residue was purified by prep-TLC (silica gel plate, dichloromethane/methyl alcohol = 10/1) to give (*R*)-3-(1-((7-bromo-4-methyl-6-(trifluoromethyl)phthalazin-1-yl)amino)ethyl)-2-methylbenzonitrile (110 mg, 244 µmol, 66.4% yield) as a light yellow solid. LCMS $[M+3]^+$: 451.2.

Step G: To a solution of (*R*)-3-(1-((7-bromo-4-methyl-6-(trifluoromethyl)phthalazin-1-yl)amino)ethyl)-2-methylbenzonitrile (40.0 mg, 89.0 µmol, 1.00 *eq.*) and morpholine (22.0 mg, 178 µmol, 22.2 µL, 2.00 *eq.*) in dioxane (3.00 mL) was added cesium carbonate (58.0 mg, 178 µmol, 2.00 *eq.*), $Pd_2(dba)_3$ (8.15 mg, 8.90 µmol, 0.10 *eq.*) and Ruphos (8.31 mg, 17.8 µmol, 0.20

eq.) and the mixture was stirred at 105 °C for 12 hours under a nitrogen atmosphere. The reaction mixture was then cooled to 25 °C, poured into water (30.0 mL), and extracted with ethyl acetate (30.0 mL × 3). The combined organic layers were washed with water (20.0 mL), dried over sodium sulfate, filtered, and concentrated under reduced pressure to give a residue. The residue was purified by prep-HPLC (column: Waters xbridge 150 × 25 mm 10 um; mobile phase A: water(10mM NH_4HCO_3), mobile phase B: acetonitrile; B%: 37% - 67%) to give (*R*)-2-methyl-3-(1-((4-methyl-7-morpholino-6-(trifluoromethyl)phthalazin-1-yl)amino)ethyl)benzonitrile (2.57 mg, 5.33 μmol, 5.99% yield, 94.5% purity) as a white solid.

^{1}H NMR (400 MHz, DMSO-*d$_6$*) δ = 8.58 (s, 1H), 8.21 (s, 1H), 7.83 (d, *J* = 6.8 Hz, 1H), 7.75 (d, *J* = 7.6 Hz, 1H), 7.61 (d, *J* = 7.6 Hz, 1H), 7.32 (t, *J* = 7.6 Hz, 1H), 5.57 (quin, *J* = 6.4 Hz, 1H), 3.87 - 3.68 (m, 4H), 3.13 - 2.97 (m, 4H), 2.67 (s, 3H), 2.65 (s, 3H), 1.57 (d, *J* = 6.8 Hz, 3H). ^{13}C NMR (101 MHz, DMSO-*d$_6$*) δ = 153.98, 151.87, 148.36, 146.31, 139.17, 131.25, 121.71, 129.71, 129.59 (q, *J* = 29.3 Hz, 1C), 127.41, 125.55, (q, *J* = 5.1 Hz, 1C), 123.38, 123.83 (q, *J* = 274.9 Hz, 1C), 119.09, 118.96, 112.73, 87.03, 54.21, 47.35, 21.86, 19.15, 17.19. HRMS (m/ z): $[M + H]^{+\cdot}$ calcd for $C_{24}H_{24}F_3N_5O$, 456.1933; found, 456.2022. HPLC (A: 0.0375% TFA in water, B: 0.01875% TFA in Acetonitrile): t_R = 4.353 min (98.9% purity).

Compound 6

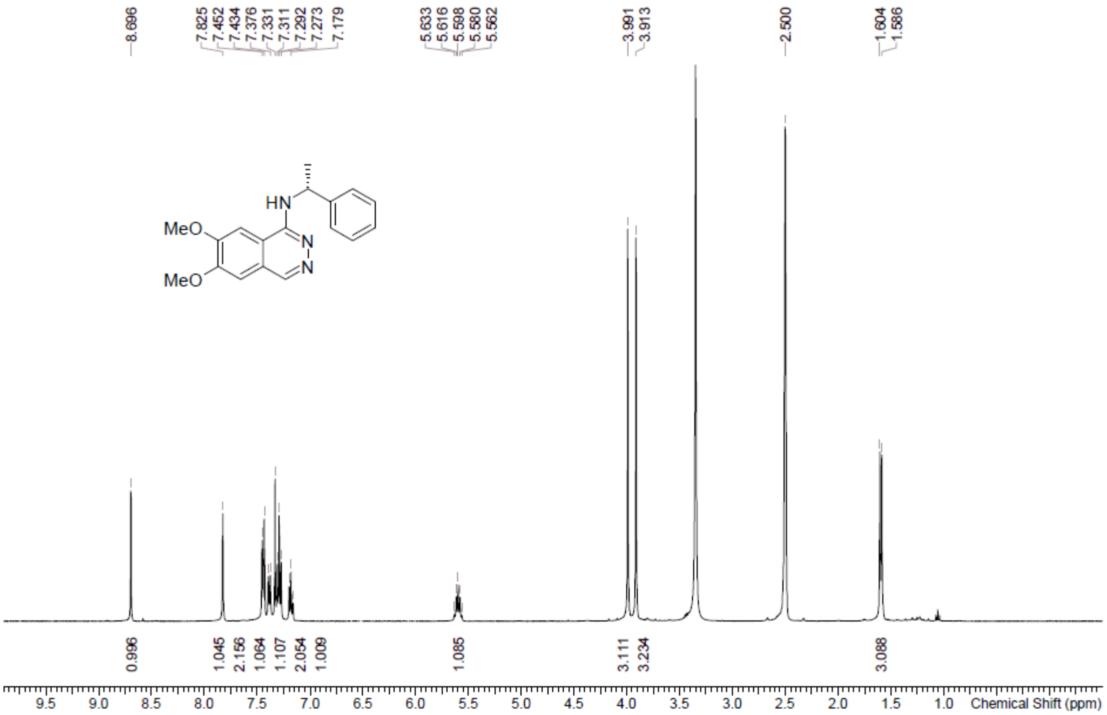

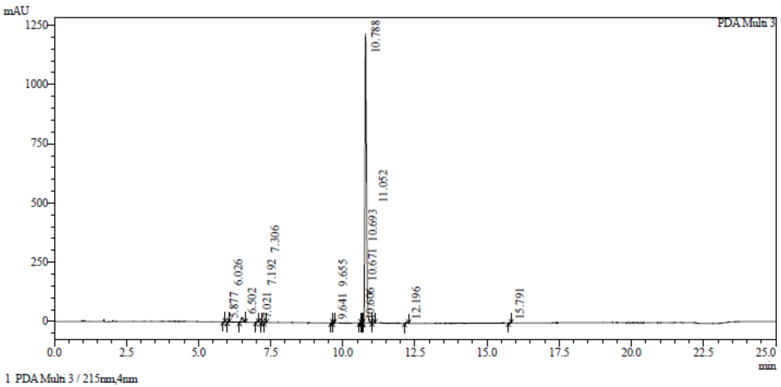

1 PDA Multi 3 / 215nm,4nm

Integration result

PeakTable

PDA Ch3 215nm

Peak#	Ret. Time	USP Width	Resolution	Height	Area	Area %
1	5.877	0.062	0.000	1174	2618	0.054
2	6.026	0.048	2.737	876	1700	0.035
3	6.502	0.120	5.681	20508	93821	1.944
4	7.021	0.074	5.350	4202	10966	0.227
5	7.192	0.070	2.390	666	1292	0.027
6	7.306	0.061	1.735	4542	10518	0.218
7	9.641	0.107	27.682	2632	5866	0.122
8	9.655	0.146	0.107	2466	3906	0.081
9	10.606	0.066	9.006	1258	2996	0.062
10	10.671	0.000	0.000	960	1885	0.039
11	10.693	0.000	0.000	1343	1108	0.023
12	10.788	0.099	0.000	1221095	4660367	96.551
13	11.052	0.085	2.865	2594	8188	0.170
14	12.196	0.096	12.608	4555	16580	0.344
15	15.791	0.106	35.520	1242	5035	0.104
Total				1270112	4826845	100.000

Compound 7

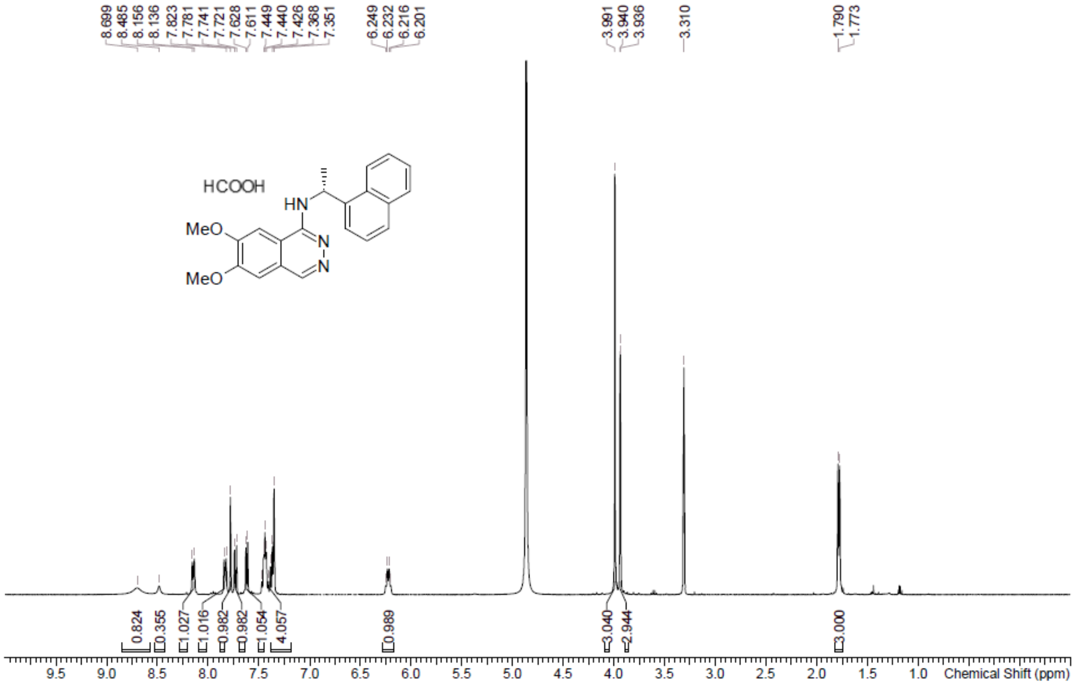

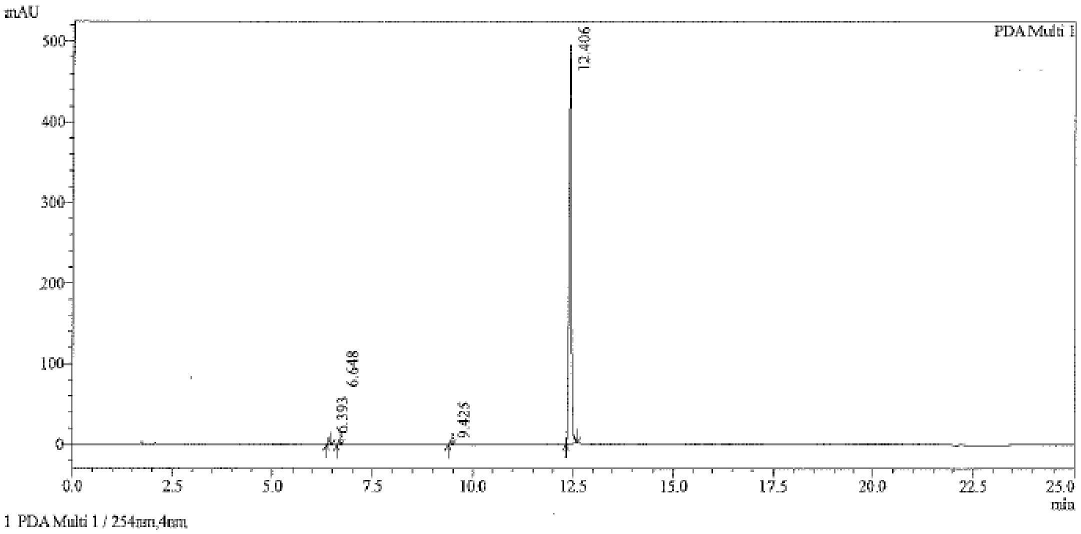

Integration result

PeakTable

PDA Ch1 254nm

Peak#	Ret. Time	USP Width	Resolution	Height	Area	Area %
1	6.393	0.082	0.000	9156	27988	1.353
2	6.648	0.087	3.022	3640	11502	0.556
3	9.425	0.092	31.057	2961	10043	0.486
4	12.406	0.104	30.357	496533	2018312	97.605
Total				512291	2067845	100.000

Compound 8

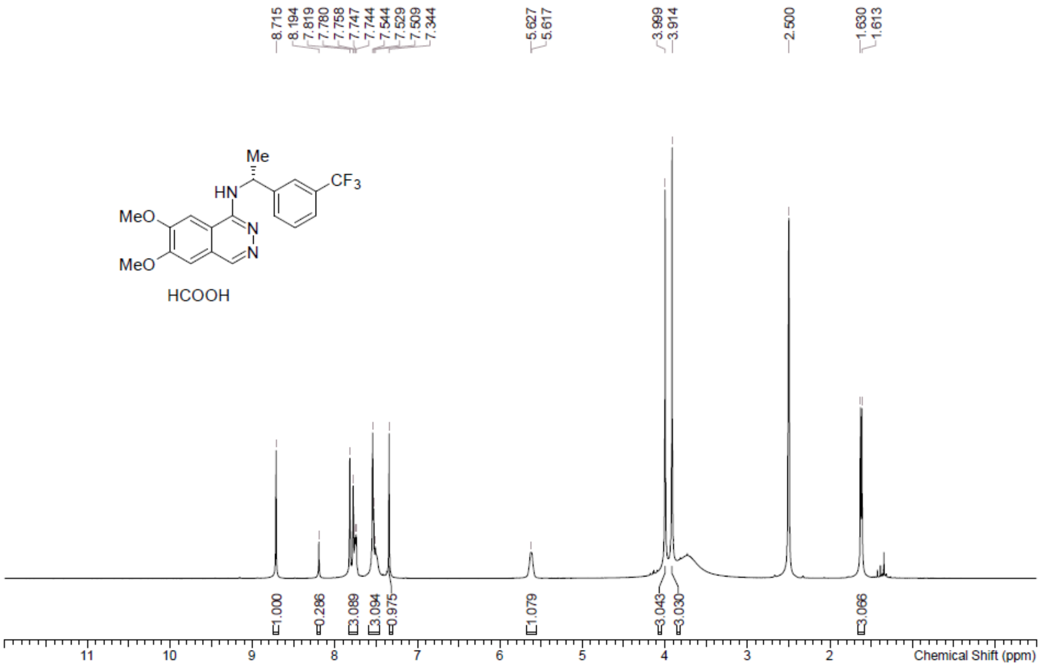

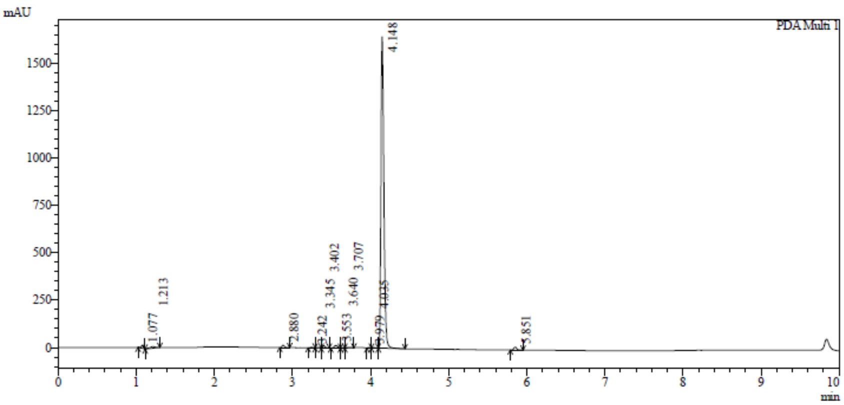

1 PDA Multi 1 / 220nm,4nm

Integration result

PeakTable

PDA Ch1 220nm

Peak#	Ret. Time	USP Width	Resolution	Height	Area	Area %
1	1.077	0.059	0.000	13821	30705	0.679
2	1.213	0.138	1.388	8160	43596	0.964
3	2.880	0.060	16.908	12525	28404	0.628
4	3.242	0.064	5.883	3796	9262	0.205
5	3.345	0.077	1.465	2062	5670	0.125
6	3.402	0.061	0.825	10775	24820	0.549
7	3.553	0.077	2.197	12857	39060	0.863
8	3.640	0.088	1.046	1158	2431	0.054
9	3.707	0.097	0.717	2170	8249	0.182
10	3.979	0.052	3.659	3799	7225	0.160
11	4.035	0.061	0.976	3840	8909	0.197
12	4.148	0.070	1.739	1627115	4276470	94.519
13	5.851	0.067	24.867	15306	39670	0.877
Total				1717383	4524470	100.000

Compound 9

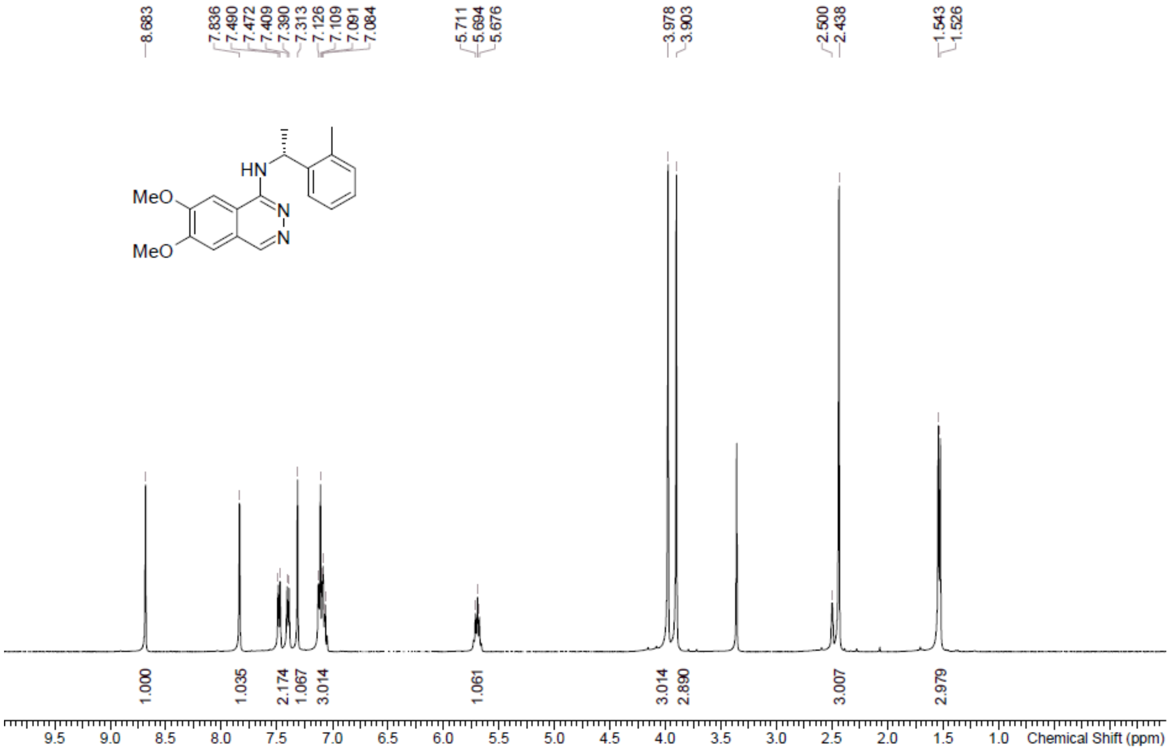

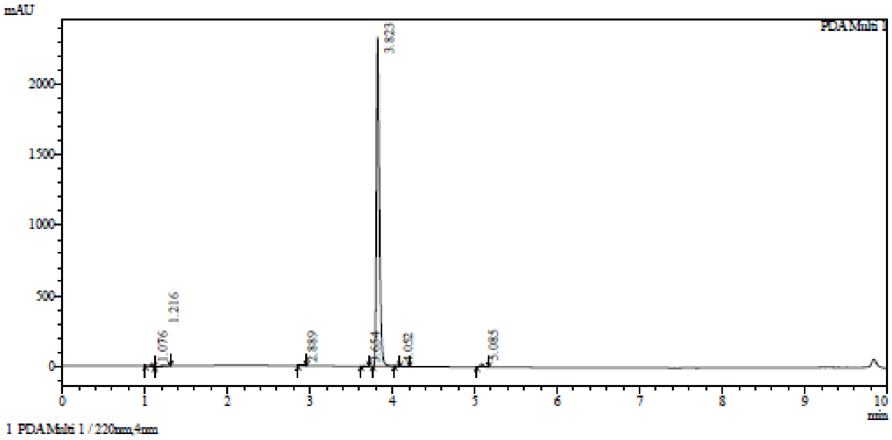

Integration result

PeakTable

PDA Ch1 220nm

Peak#	Ret. Time	USP Width	Resolution	Height	Area	Area %
1	1.076	0.074	0.000	11717	33221	0.550
2	1.216	0.138	1.320	7933	41393	0.685
3	2.889	0.062	16.774	2805	6406	0.106
4	3.654	0.060	12.620	3017	6956	0.115
5	3.823	0.068	2.641	2332796	5900162	97.644
6	4.052	0.056	3.695	1128	2102	0.035
7	5.085	0.062	17.523	22252	52276	0.865
Total				2381648	6042515	100.000

Compound 10

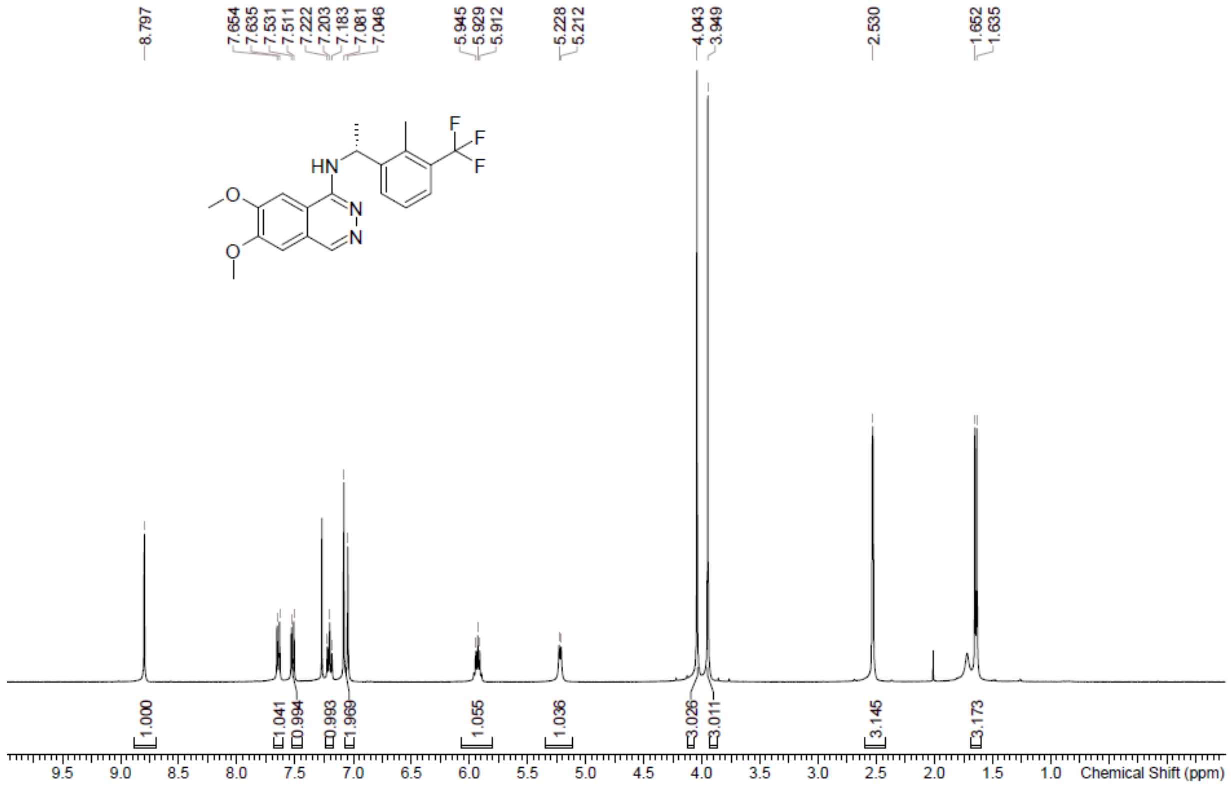

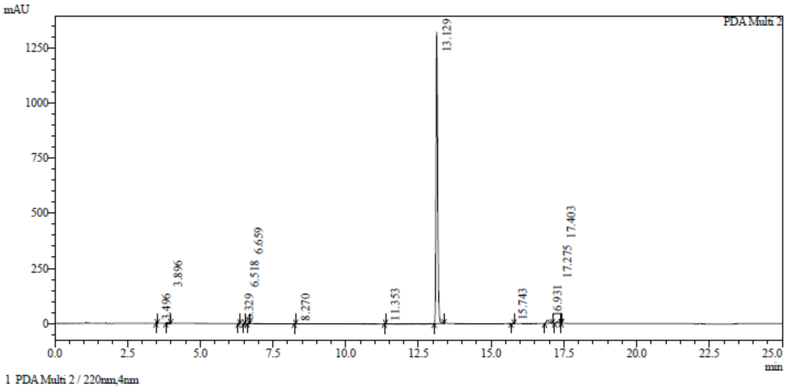

Integration result

PeakTable

PDA Ch2 220nm

Peak#	Ret. Time	USP Width	Resolution	Height	Area	Area %
1	3.496	0.066	0.000	940	2085	0.039
2	3.896	0.135	3.989	3815	19163	0.356
3	6.329	0.067	24.128	520	1094	0.020
4	6.518	0.059	3.003	936	1851	0.034
5	6.659	0.059	2.399	3513	7641	0.142
6	8.270	0.065	26.114	549	1061	0.020
7	11.353	0.061	49.137	553	1135	0.021
8	13.129	0.102	21.725	1322261	5193886	96.431
9	15.743	0.091	27.051	1131	3847	0.071
10	16.931	0.177	8.854	14653	99926	1.855
11	17.275	0.178	1.933	8032	53241	0.988
12	17.403	0.067	1.045	1025	1191	0.022
Total				1357928	5386123	100.000

Compound 11

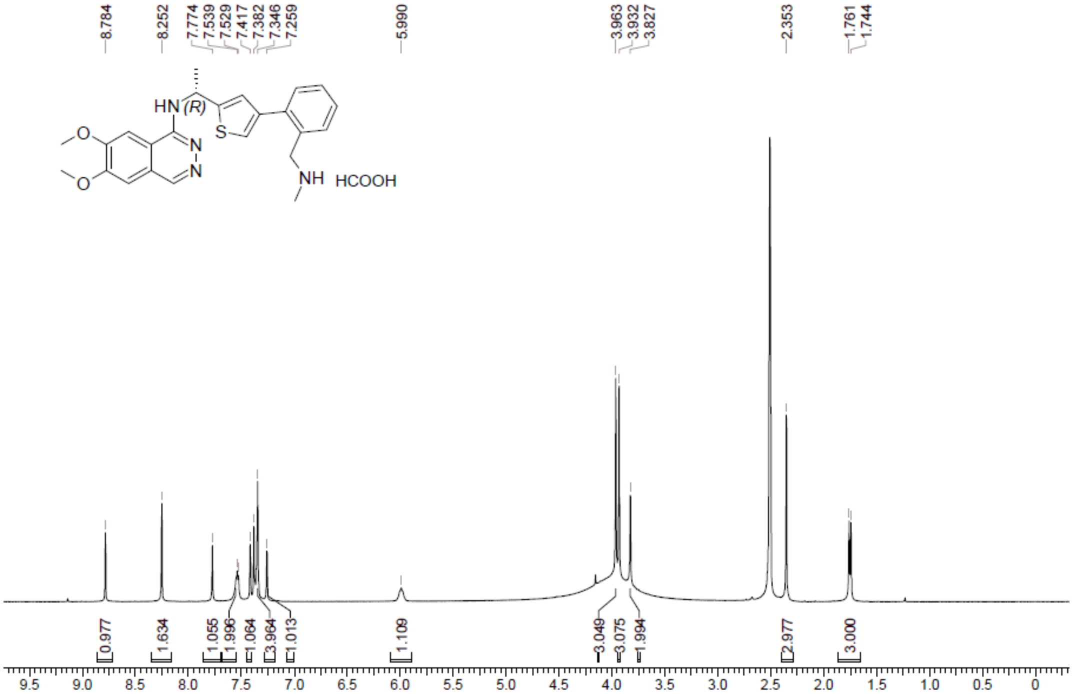

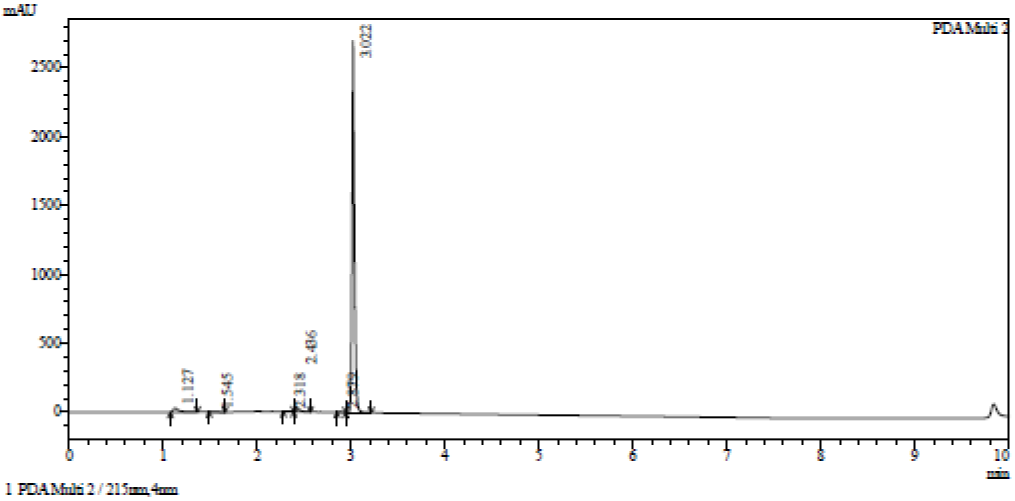

Integration result

PeakTable

PDA Ch2 215nm

Peak#	Ret. Time	USP Width	Resolution	Height	Area	Area %
1	1.127	0.101	0.000	31166	144943	2.795
2	1.545	0.115	3.868	1785	7498	0.145
3	2.318	0.065	8.588	1028	2310	0.045
4	2.436	0.063	1.859	28249	75389	1.454
5	2.879	0.054	7.593	2796	5243	0.101
6	3.022	0.047	2.837	2679410	4950133	95.461
Total				2744435	5185517	100.000

Compound 12

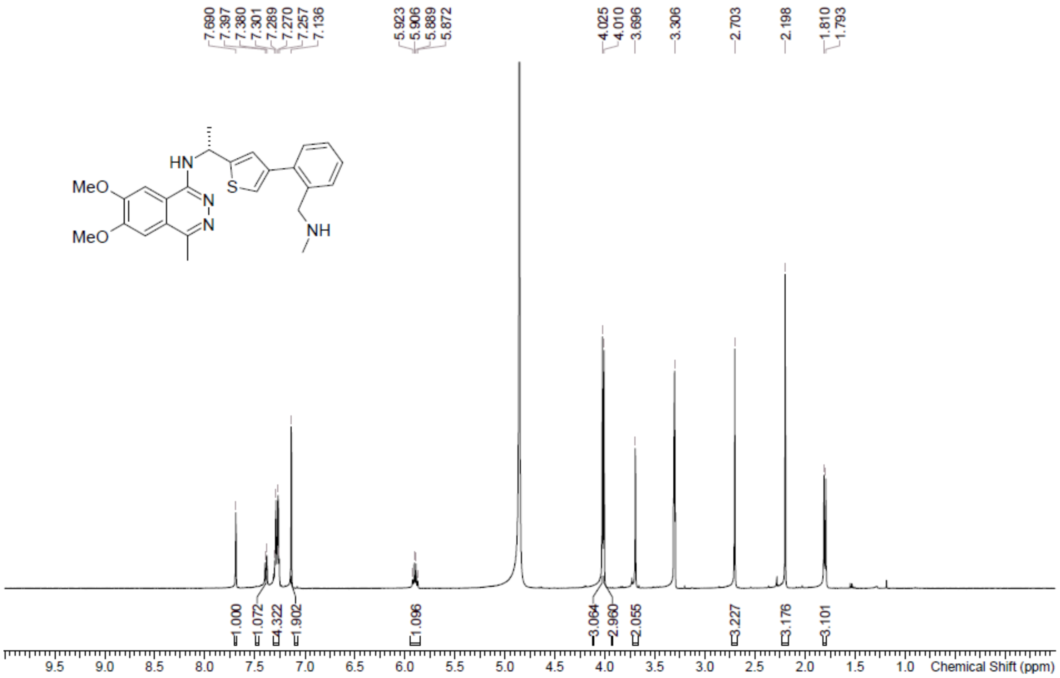

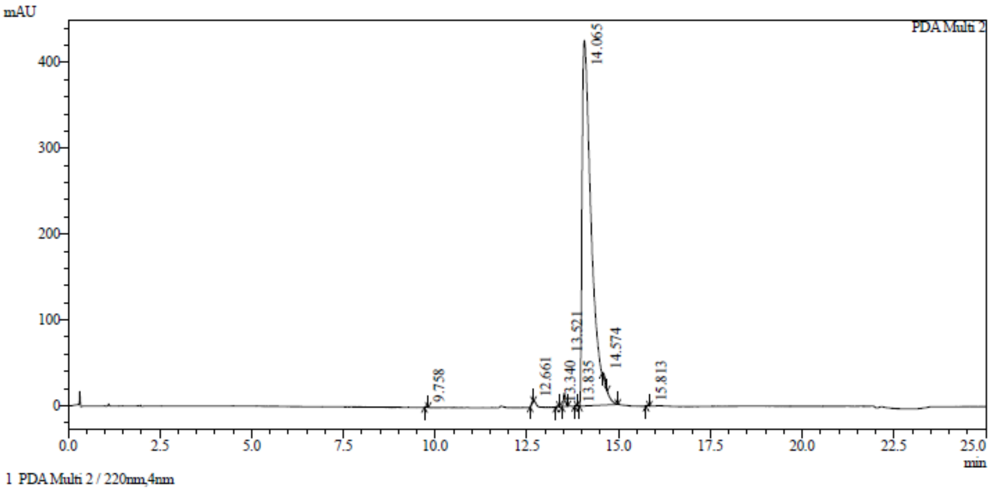

Integration result

PeakTable

PDA Ch2 220nm

Peak#	Ret. Time	USP Width	Resolution	Height	Area	Area %
1	9.758	0.084	0.000	1710	5648	0.074
2	12.661	0.073	36.883	2028	7355	0.096
3	13.340	0.128	6.737	1495	5569	0.073
4	13.521	0.101	1.580	15686	58913	0.772
5	13.835	0.064	3.820	754	1729	0.023
6	14.065	0.432	0.927	426098	7523836	98.616
7	14.574	0.113	1.868	3434	22368	0.293
8	15.813	0.121	10.618	1173	4015	0.053
Total				452378	7629432	100.000

Compound 13

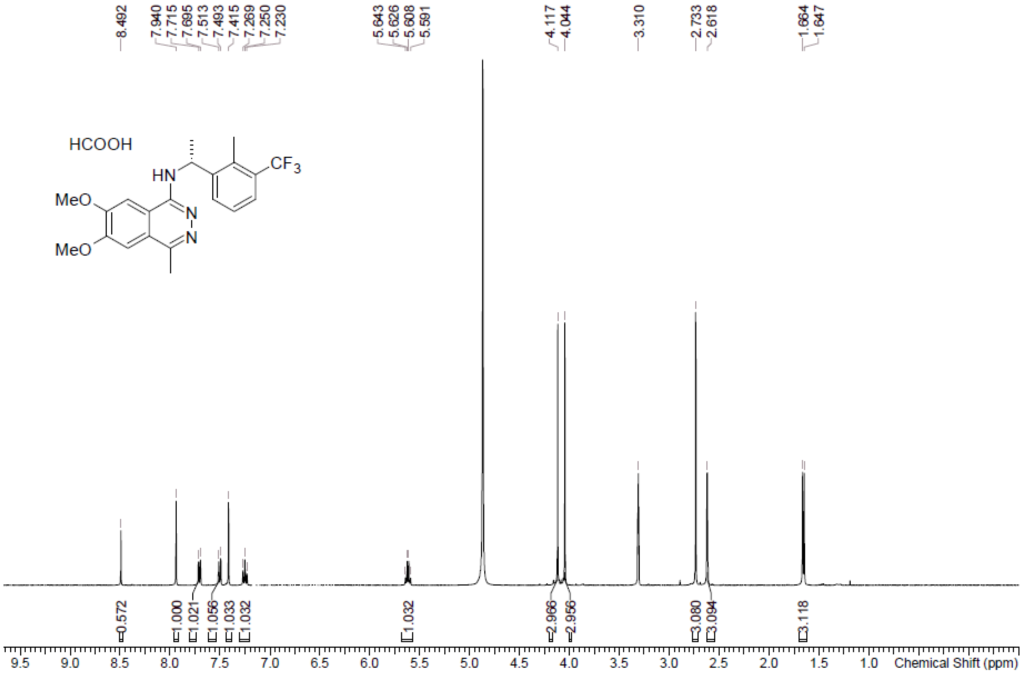

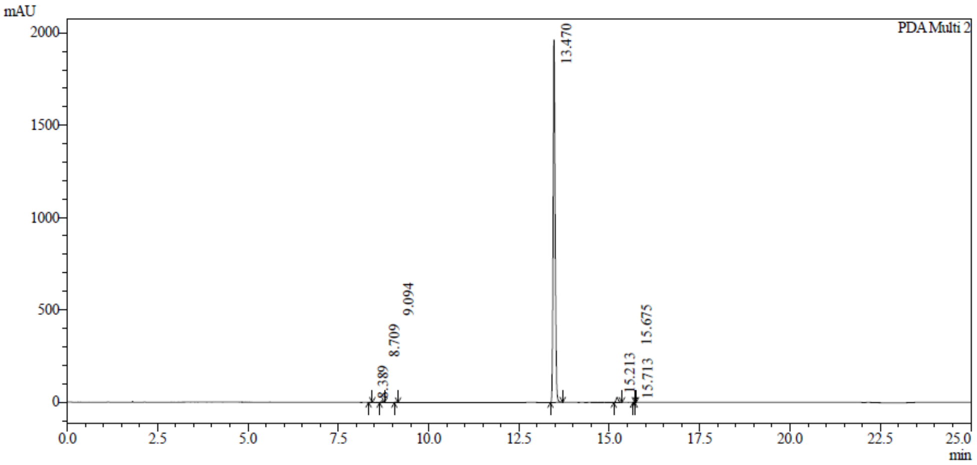

1 PDA Multi 2 / 220nm,4nm

Integration result

PeakTable

PDA Ch2 220nm

Peak#	Ret. Time	USP Width	Resolution	Height	Area	Area %
1	8.389	0.080	0.000	1502	4629	0.059
2	8.709	0.091	3.726	10039	34172	0.435
3	9.094	0.084	4.393	3704	11779	0.150
4	13.470	0.102	47.000	1964553	7680212	97.690
5	15.213	0.117	15.916	27774	126098	1.604
6	15.675	0.152	3.435	1359	3689	0.047
7	15.713	0.090	0.321	883	1272	0.016
Total				2009815	7861852	100.000

Compound 14

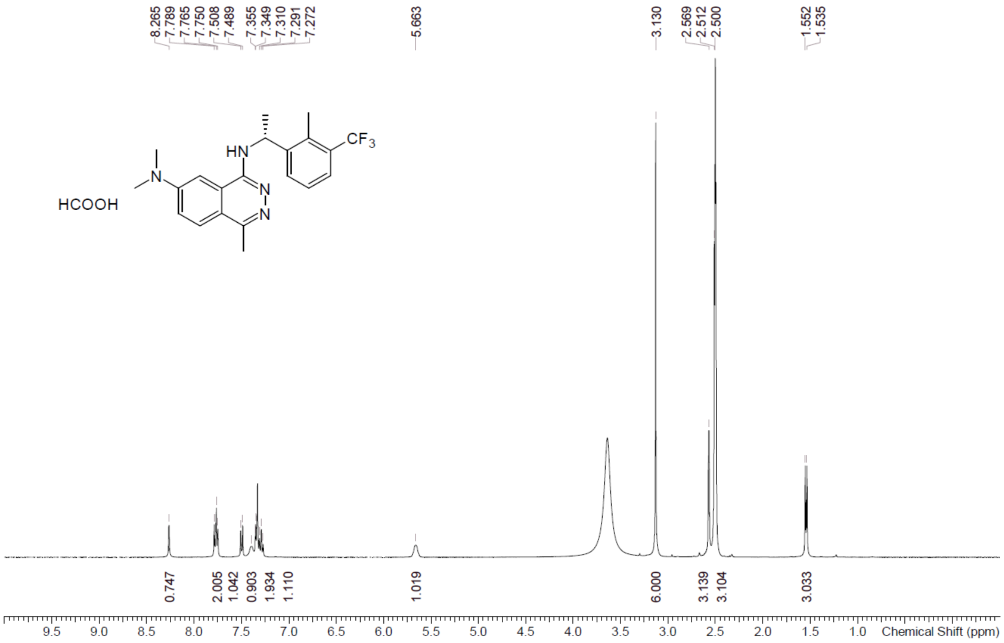

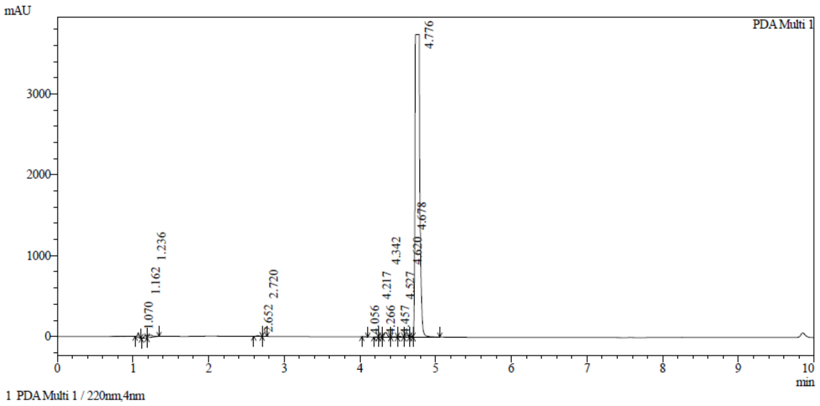

Integration result

PeakTable

PDA Ch1 220nm

Peak#	Ret. Time	USP Width	Resolution	Height	Area	Area %
1	1.070	0.040	0.000	58346	87149	0.515
2	1.162	0.103	1.290	29931	76874	0.454
3	1.236	0.581	0.216	31220	133403	0.789
4	2.652	0.072	4.340	8859	24199	0.143
5	2.720	0.065	1.004	821	1288	0.008
6	4.056	0.049	23.495	828	1537	0.009
7	4.217	0.052	3.201	2462	4557	0.027
8	4.266	0.055	0.917	3084	5779	0.034
9	4.342	0.071	1.214	55607	149307	0.883
10	4.457	0.088	1.441	3105	9654	0.057
11	4.527	0.053	0.989	7785	15421	0.091
12	4.620	0.053	1.761	25959	51594	0.305
13	4.678	0.093	0.782	6992	15309	0.091
14	4.776	0.079	1.143	3740923	16339524	96.594
Total				3975922	16915597	100.000

Compound 15

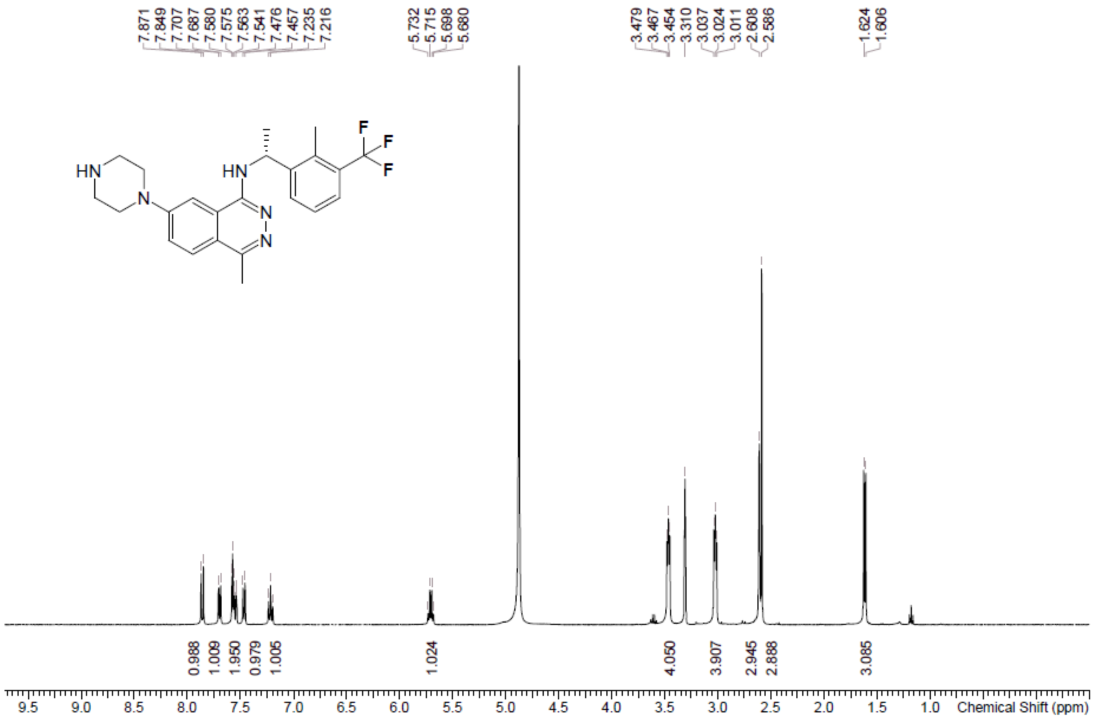

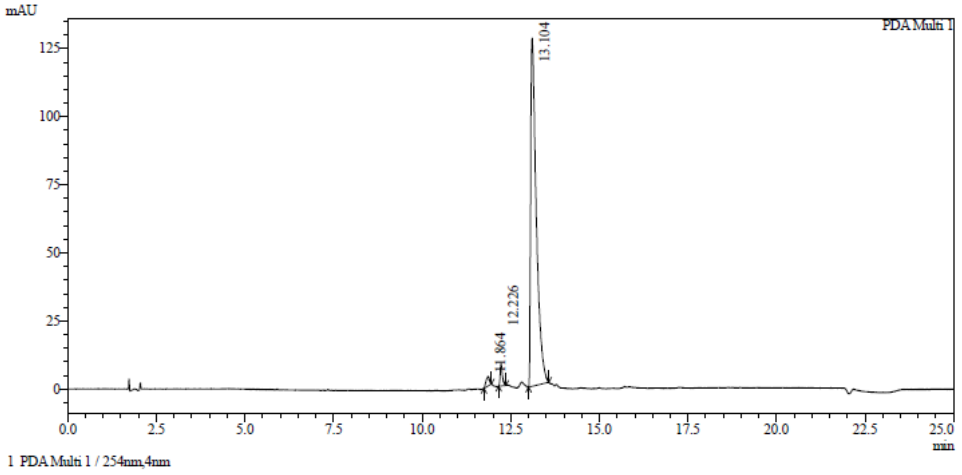

Integration result

PeakTable

PDA Ch1 254nm

Peak#	Ret. Time	USP Width	Resolution	Height	Area	Area %
1	11.864	0.183	0.000	3520	24300	1.646
2	12.226	0.112	2.457	8338	36272	2.457
3	13.104	0.282	4.450	127911	1415666	95.897
Total				139769	1476239	100.000

Compound 16

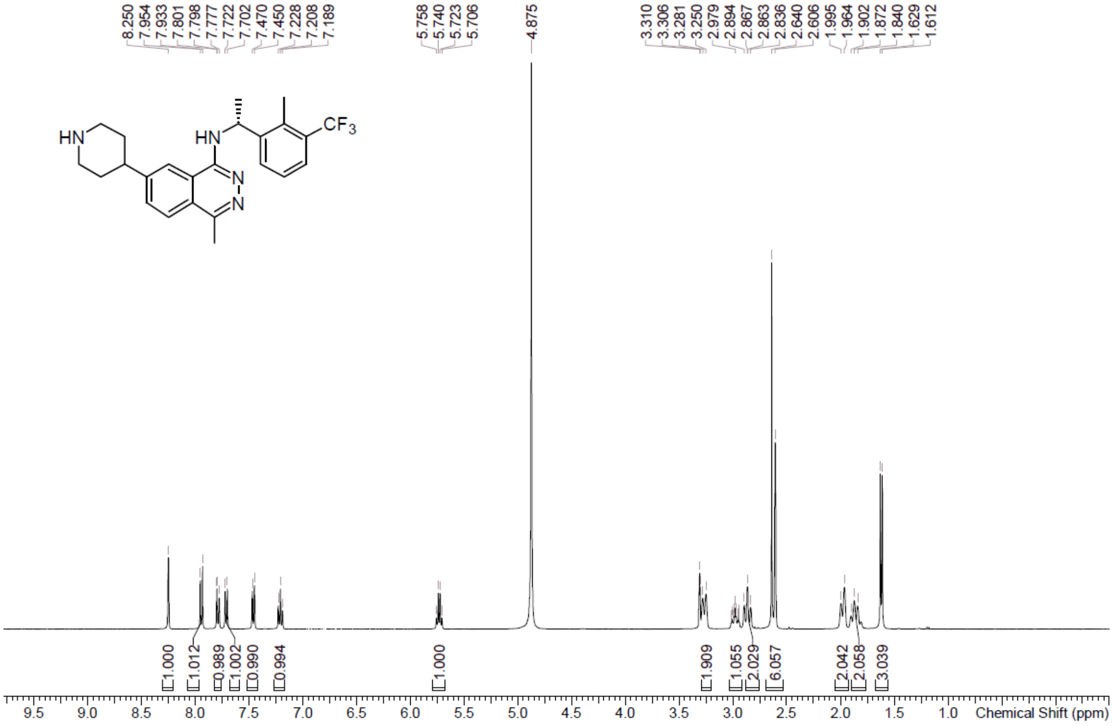

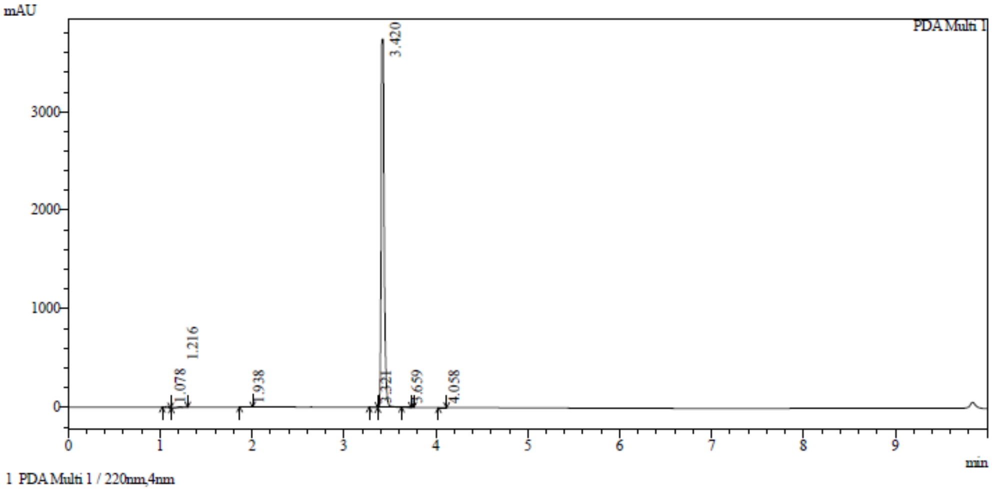

Integration result

PeakTable

PDA Ch1 220nm

Peak#	Ret. Time	USP Width	Resolution	Height	Area	Area %
1	1.078	0.068	0.000	10987	27903	0.323
2	1.216	0.129	1.404	7749	37539	0.434
3	1.938	0.108	6.092	1863	7552	0.087
4	3.321	0.084	14.373	1884	5999	0.069
5	3.420	0.048	1.497	3737930	8561618	98.991
6	3.659	0.056	4.616	3142	6497	0.075
7	4.058	0.057	7.099	902	1811	0.021
Total				3764456	8648920	100.000

Compound 17

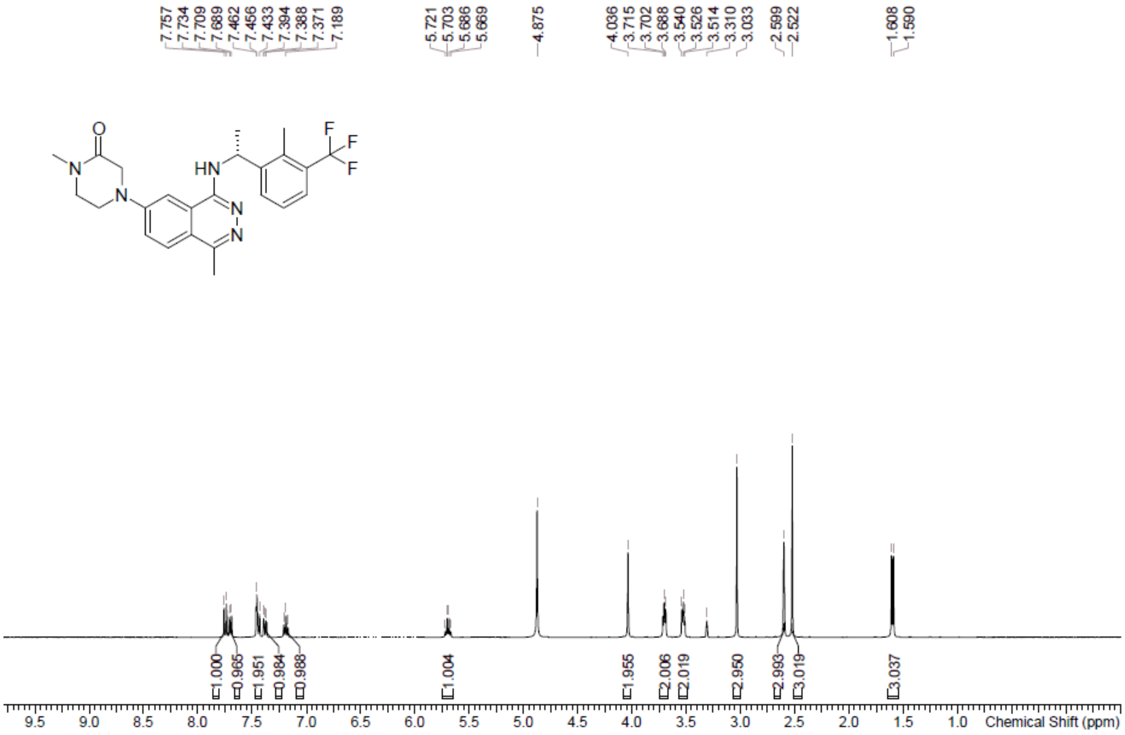

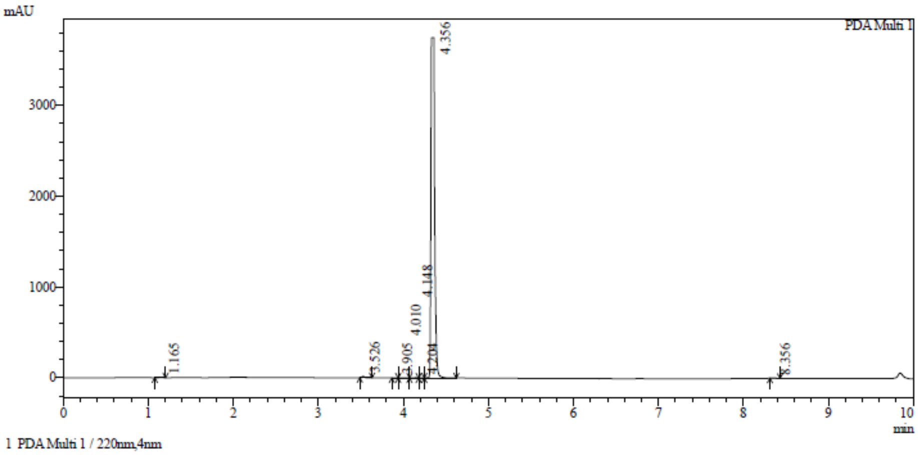

Integration result

PeakTable

PDA Ch1 220nm

Peak#	Ret. Time	USP Width	Resolution	Height	Area	Area %
1	1.165	0.119	0.000	3107	12357	0.107
2	3.526	0.053	27.572	15657	31460	0.273
3	3.905	0.053	7.148	1745	3412	0.030
4	4.010	0.076	1.625	1647	5930	0.051
5	4.148	0.054	2.107	4188	10987	0.095
6	4.204	0.097	0.738	1434	3713	0.032
7	4.356	0.058	1.962	3750488	11467464	99.372
8	8.356	0.075	60.033	1690	4567	0.040
Total				3779956	11539891	100.000

Compound 18

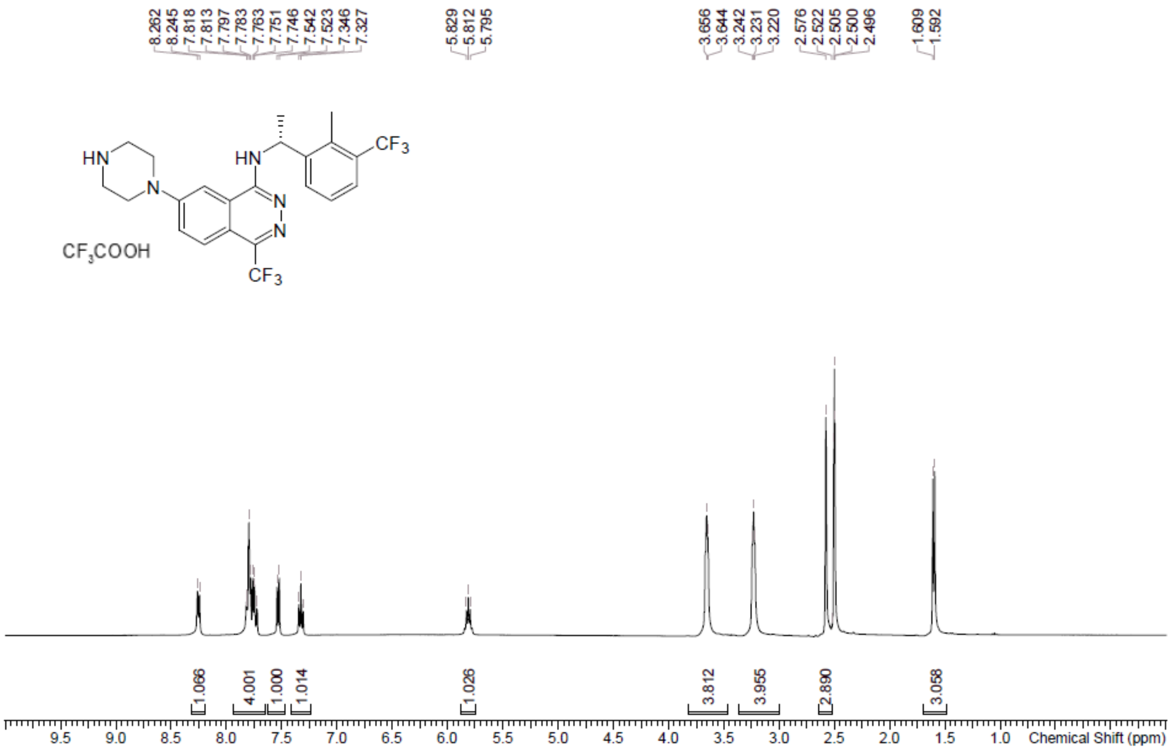

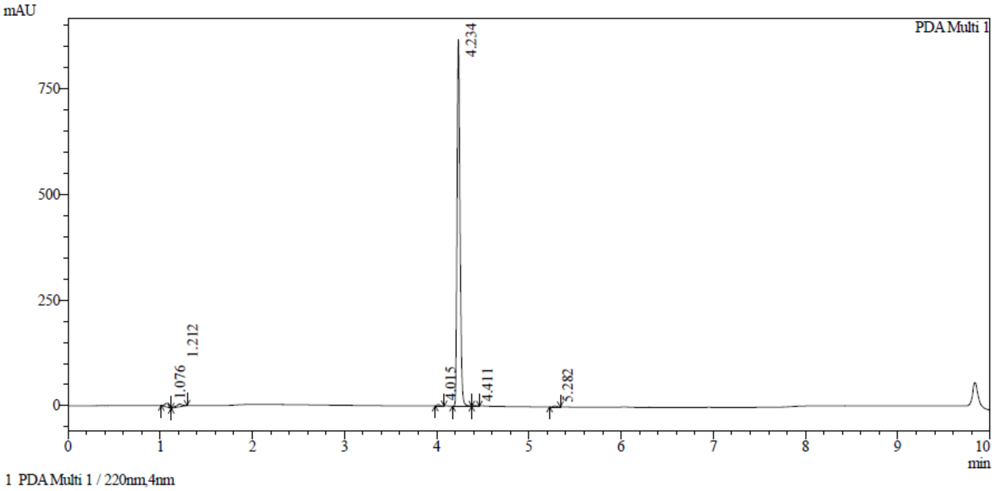

Integration result

PeakTable

PDA Ch1 220nm

Peak#	Ret. Time	USP Width	Resolution	Height	Area	Area %
1	1.076	0.076	0.000	8585	24687	1.235
2	1.212	0.116	1.427	6317	29141	1.458
3	4.015	0.054	32.970	3847	7782	0.389
4	4.234	0.059	3.870	865333	1926477	96.389
5	4.411	0.064	2.880	1764	4081	0.204
6	5.282	0.062	13.834	2668	6490	0.325
Total				888514	1998658	100.000

Compound 19

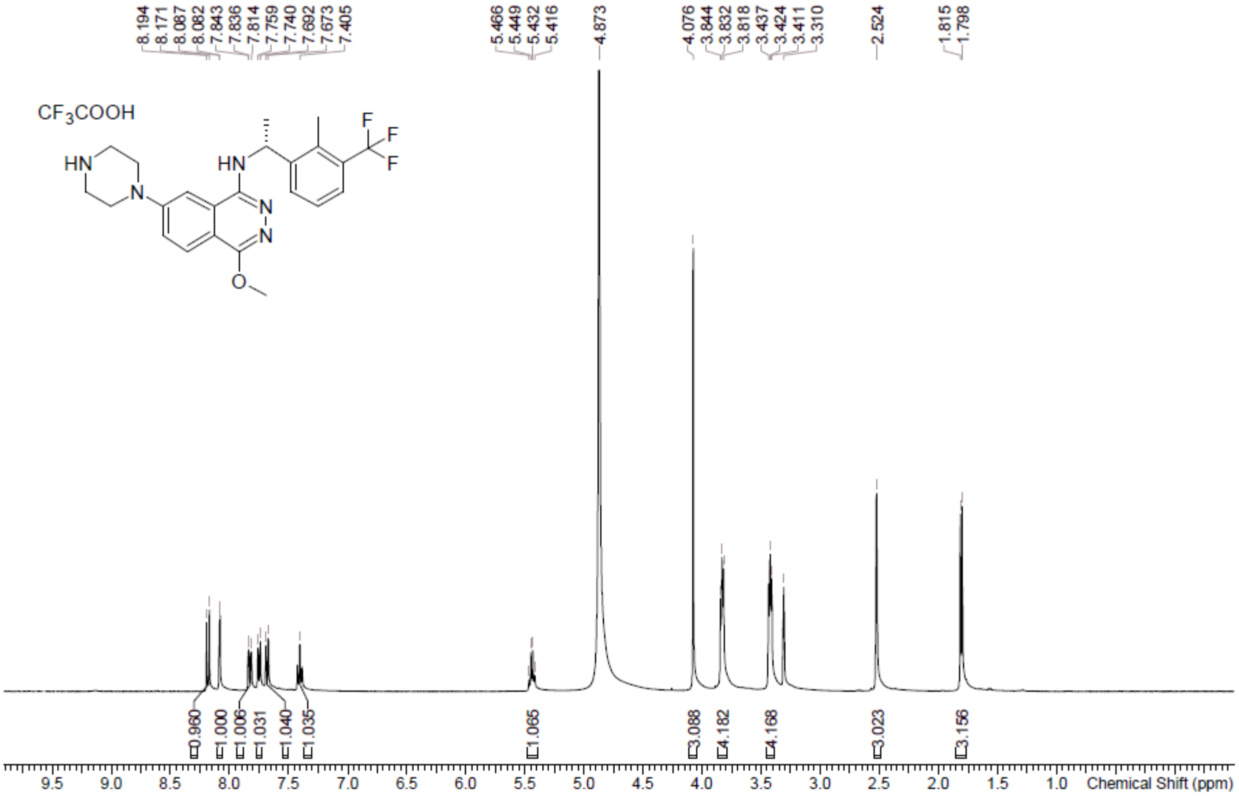

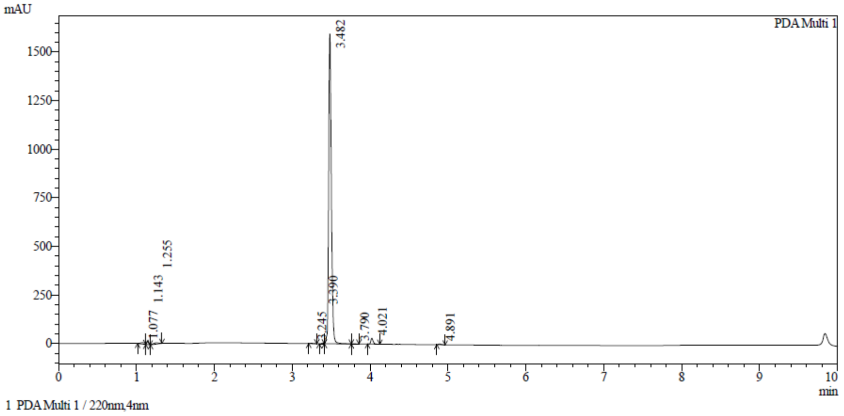

Integration result

PeakTable

PDA Ch1 220nm

Peak#	Ret. Time	USP Width	Resolution	Height	Area	Area %
1	1.077	0.288	0.000	7334	29941	0.748
2	1.143	0.046	0.392	18539	30130	0.752
3	1.255	0.135	1.242	7114	31562	0.788
4	3.245	0.057	20.726	1598	3459	0.086
5	3.390	0.057	2.547	2951	5426	0.135
6	3.482	0.064	1.541	1590572	3833504	95.718
7	3.790	0.053	5.310	1237	2410	0.060
8	4.021	0.054	4.347	29552	60316	1.506
9	4.891	0.055	16.007	4015	8232	0.206
Total				1662912	4004981	100.000

Compound 20

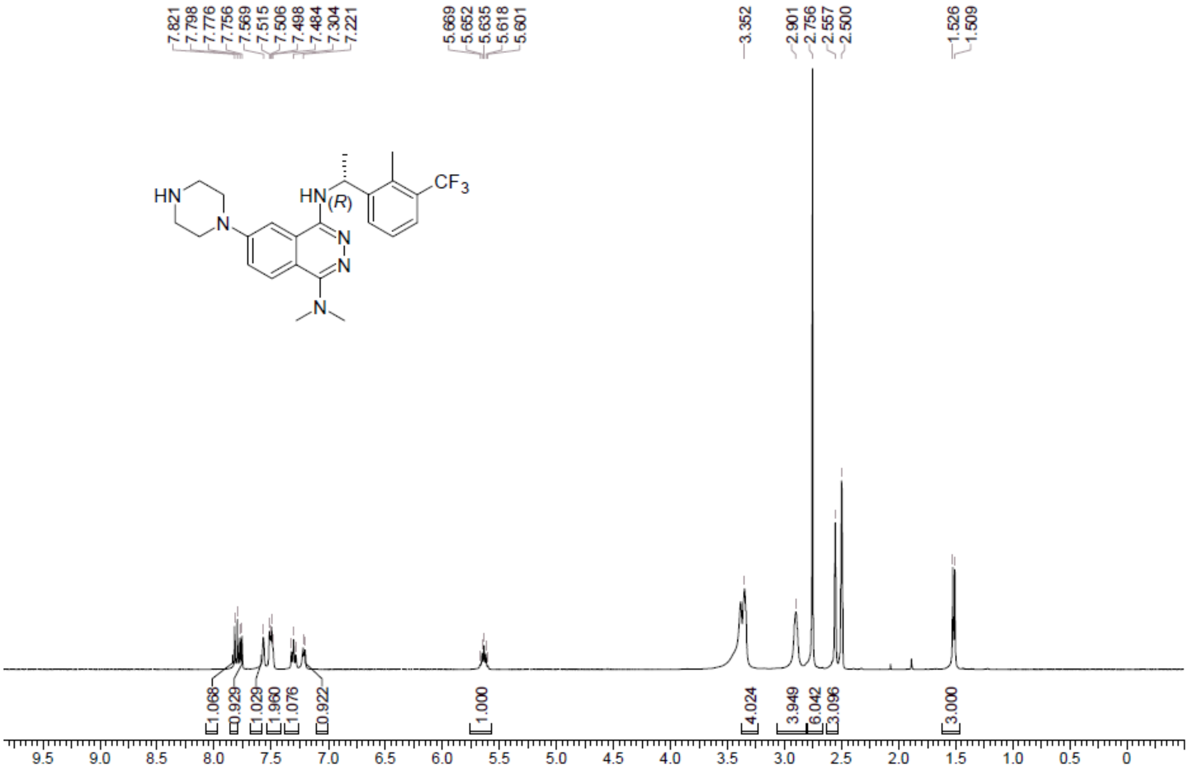

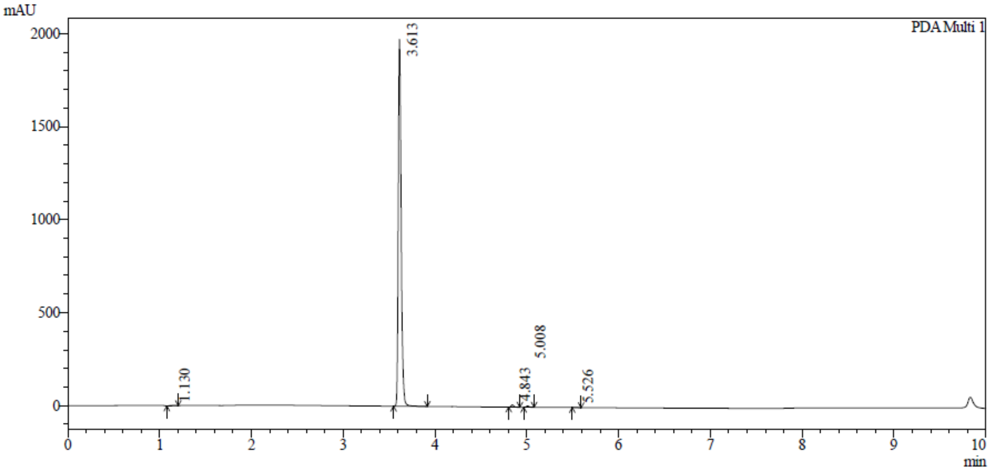

1 PDA Multi 1 / 220nm,4nm

Integration result

PeakTable

PDA Ch1 220nm

Peak#	Ret. Time	USP Width	Resolution	Height	Area	Area %
1	1.130	0.119	0.000	2554	10152	0.233
2	3.613	0.059	27.939	1961482	4301456	98.855
3	4.843	0.054	21.802	11571	23565	0.542
4	5.008	0.054	3.064	6980	14296	0.329
5	5.526	0.055	9.472	892	1830	0.042
Total				1983479	4351300	100.000

Compound 21

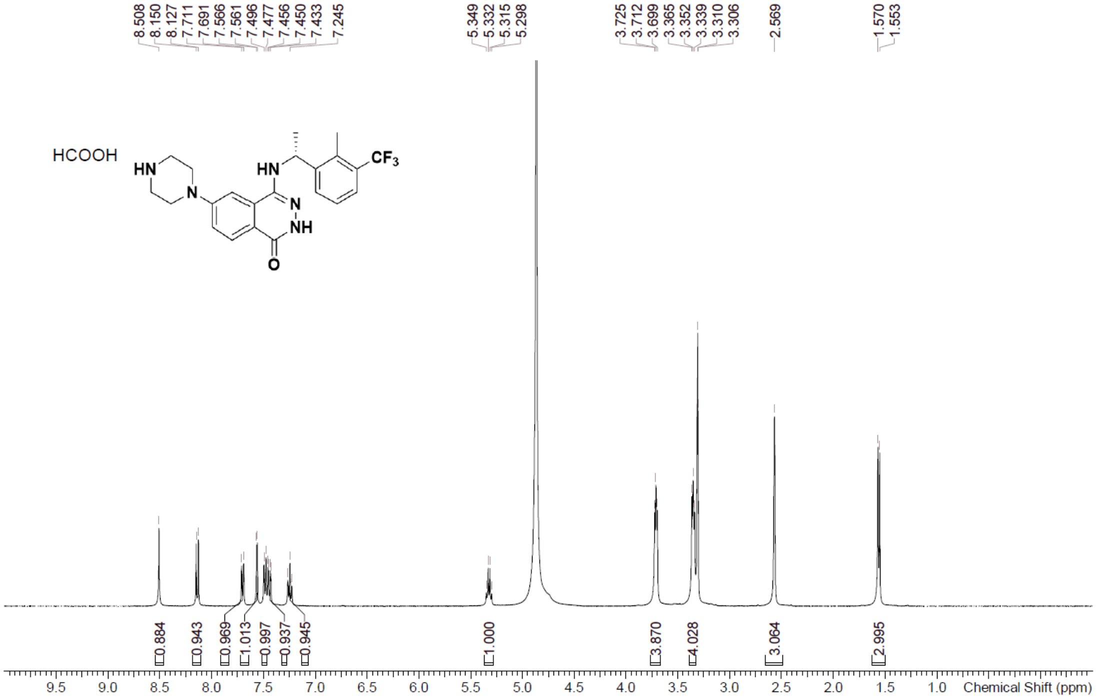

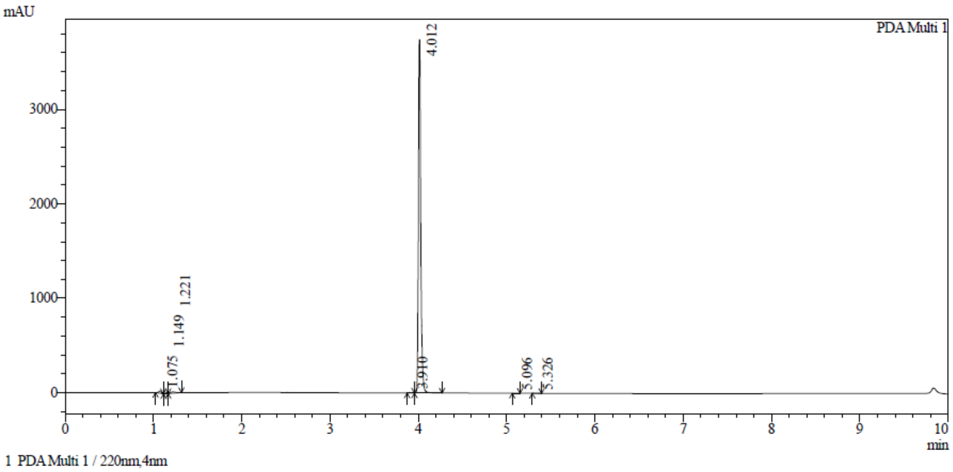

Integration result

PeakTable

PDA Ch1 220nm

Peak#	Ret. Time	USP Width	Resolution	Height	Area	Area %
1	1.075	0.055	0.000	24580	50678	0.725
2	1.149	0.094	1.000	10690	22494	0.322
3	1.221	0.171	0.545	14075	64648	0.925
4	3.910	0.049	24.448	1692	3145	0.045
5	4.012	0.042	2.249	3739749	6843415	97.926
6	5.096	0.055	22.485	790	1570	0.022
7	5.326	0.056	4.148	1156	2435	0.035
Total				3792732	6988384	100.000

Compound 22

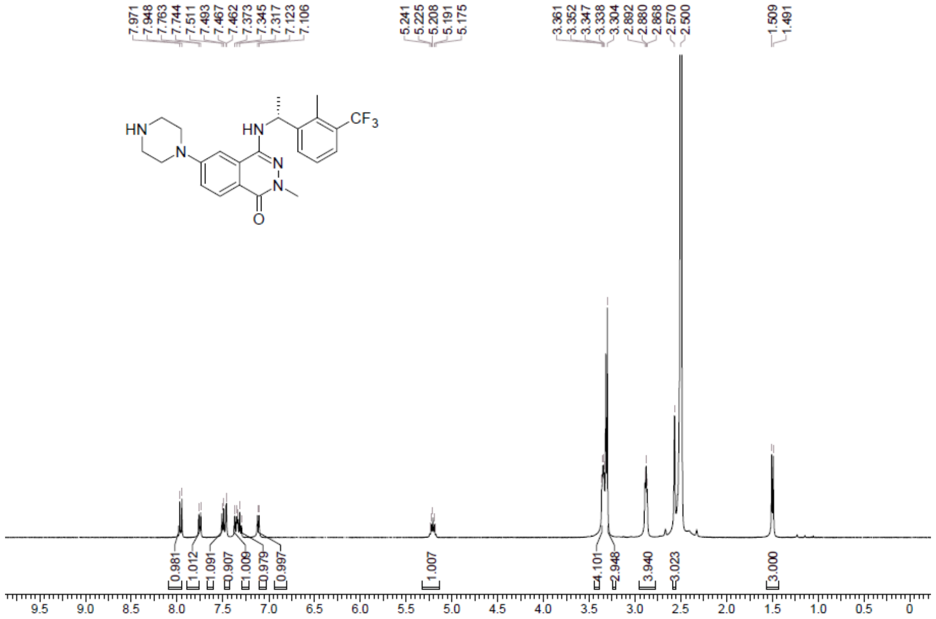

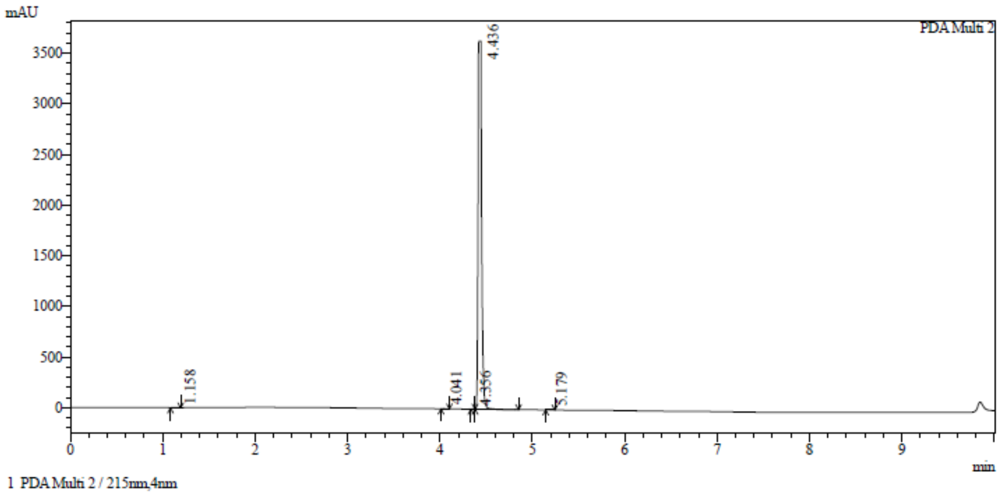

1 PDA Multi 2 / 215nm,4nm

Integration result

PeakTable

PDA Ch2 215nm

Peak#	Ret. Time	USP Width	Resolution	Height	Area	Area %
1	1.158	0.121	0.000	3470	14143	0.142
2	4.041	0.053	33.057	1361	2622	0.026
3	4.356	0.050	6.093	1105	1997	0.020
4	4.436	0.054	1.536	3634315	9938926	99.779
5	5.179	0.069	12.143	1260	3289	0.033
Total				3641511	9960977	100.000

Compound 23

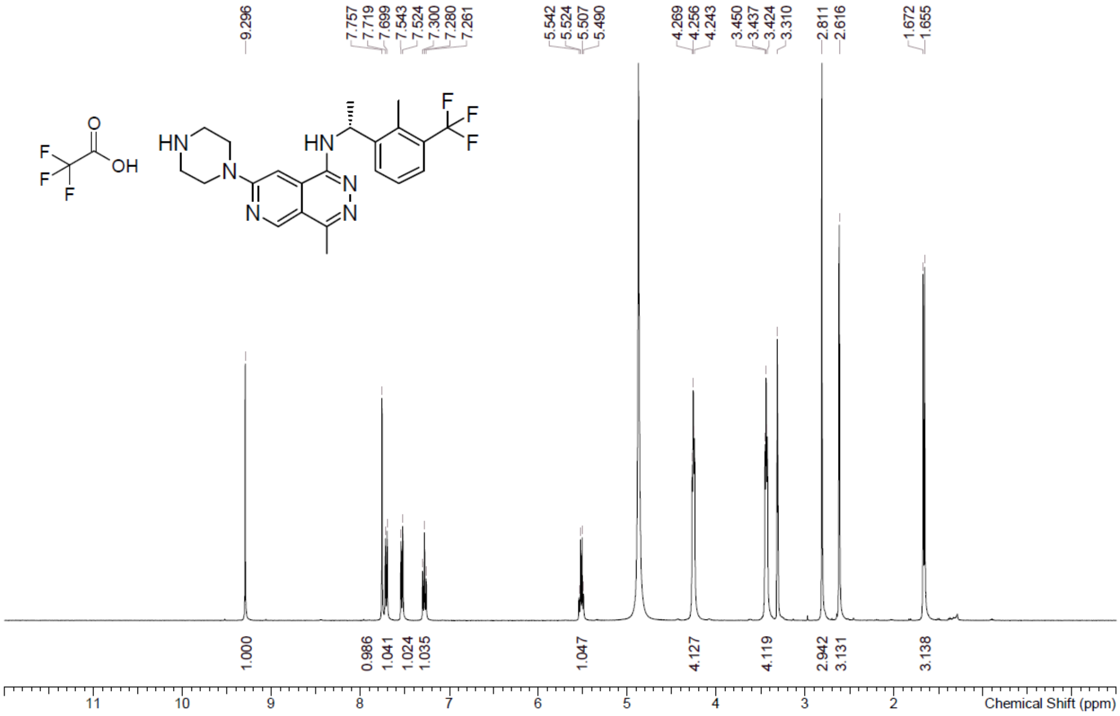

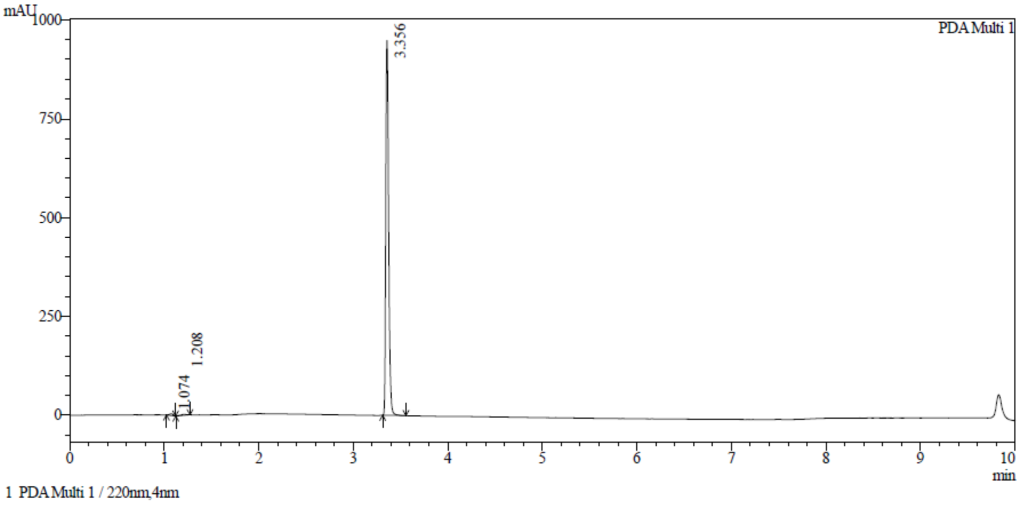

Integration result

PeakTable

PDA Ch1 220nm

Peak#	Ret. Time	USP Width	Resolution	Height	Area	Area %
1	1.074	0.071	0.000	3966	10673	0.530
2	1.208	0.101	1.556	2177	9275	0.460
3	3.356	0.056	27.416	937011	1995233	99.010
Total				943154	2015181	100.000

Compound 24

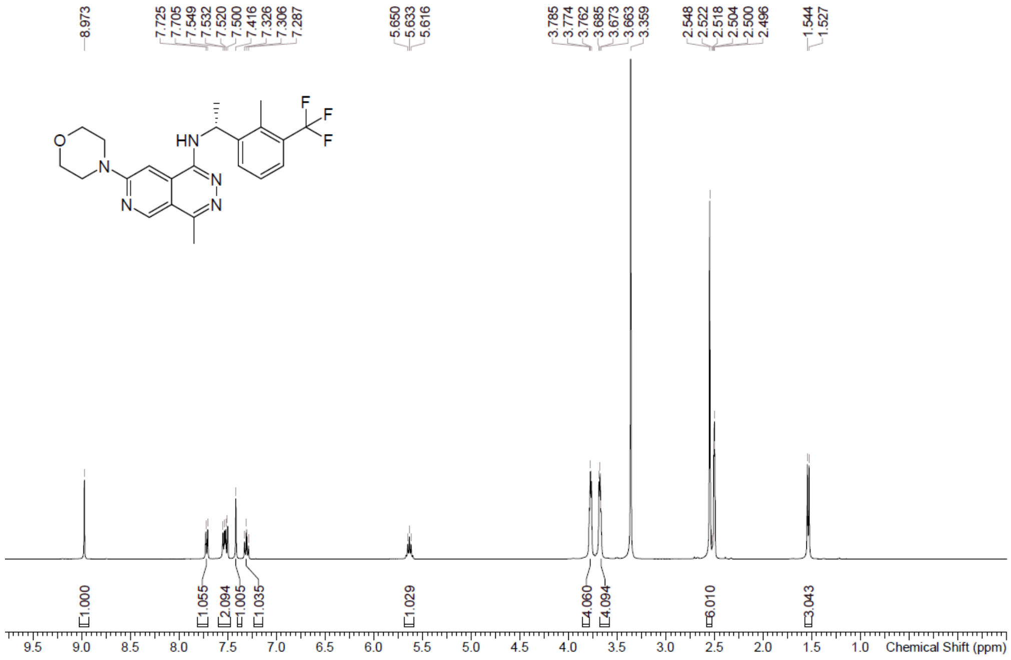

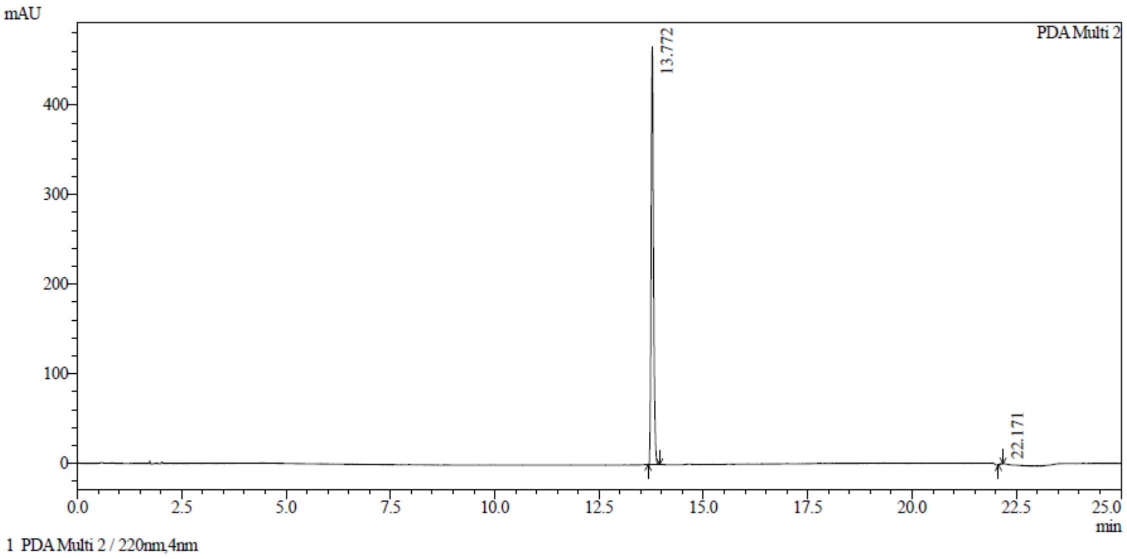

Integration result

PeakTable

PDA Ch2 220nm

Peak#	Ret. Time	USP Width	Resolution	Height	Area	Area %
1	13.772	0.103	0.000	466380	1838795	99.829
2	22.171	0.108	79.584	369	3150	0.171
Total				466749	1841945	100.000

Compound 25

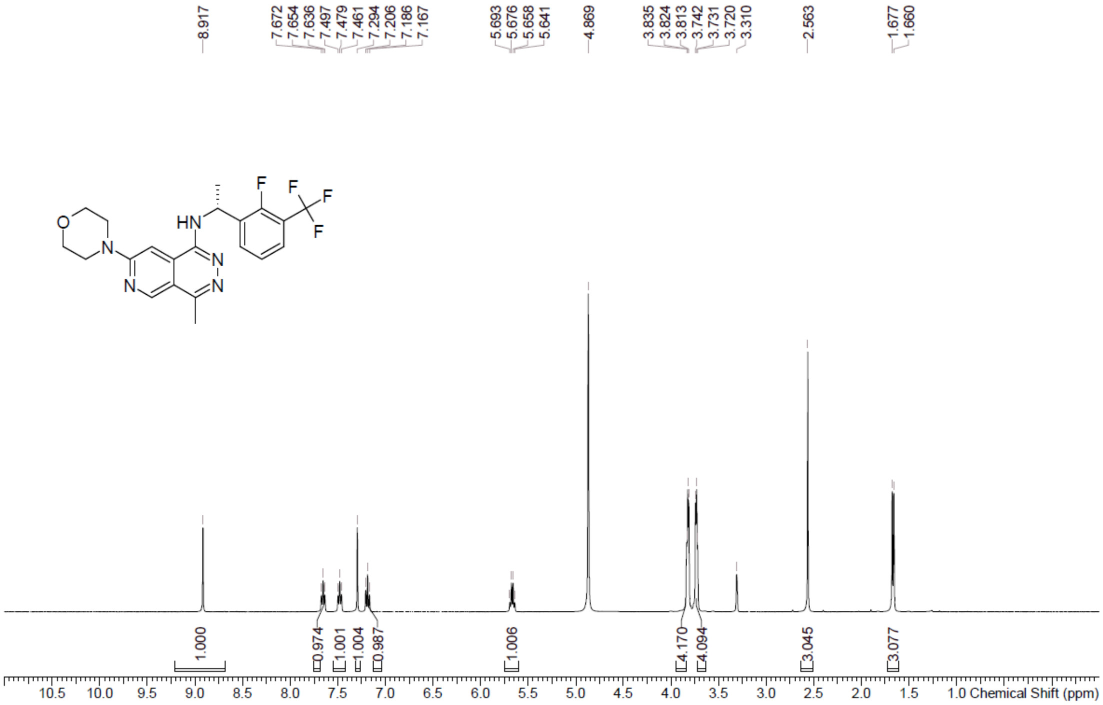

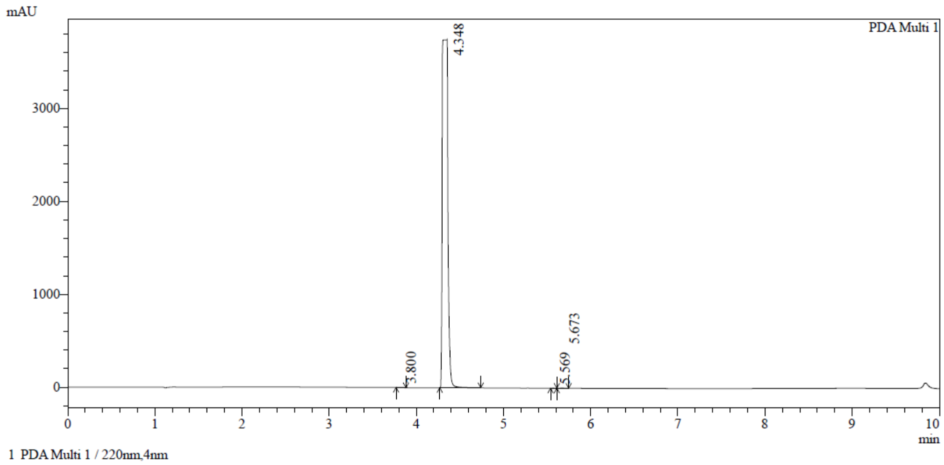

Integration result

PeakTable

PDA Ch1 220nm

Peak#	Ret. Time	USP Width	Resolution	Height	Area	Area %
1	3.800	0.059	0.000	1246	3142	0.019
2	4.348	0.080	7.860	3739969	16492583	99.921
3	5.569	0.051	18.619	1195	2263	0.014
4	5.673	0.067	1.759	2828	7553	0.046
Total				3745237	16505542	100.000

Compound 26

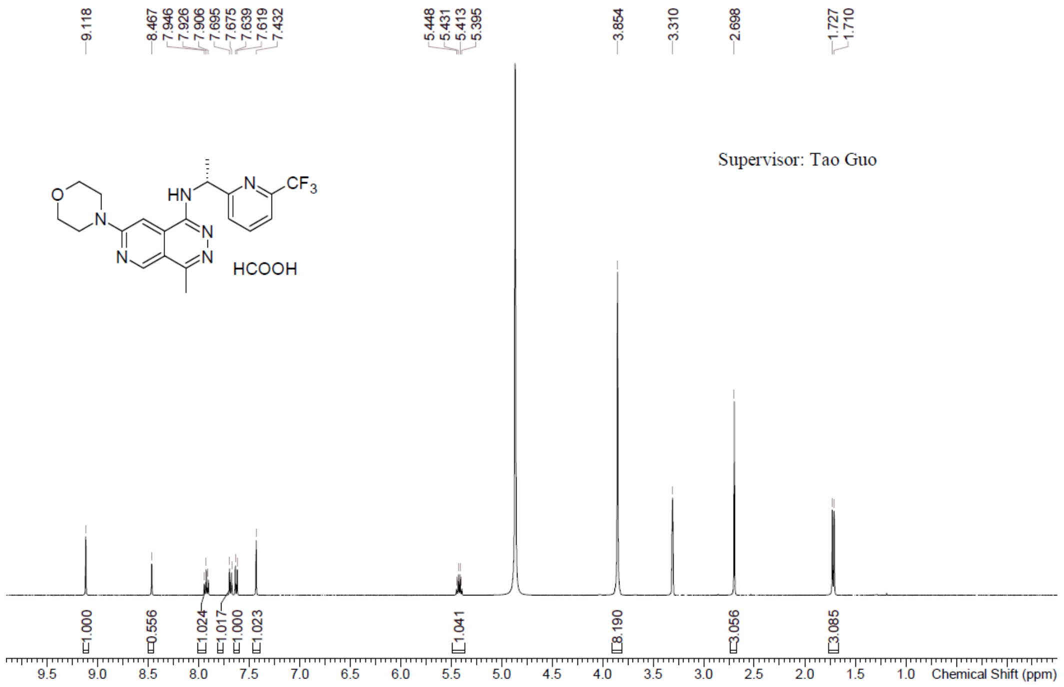

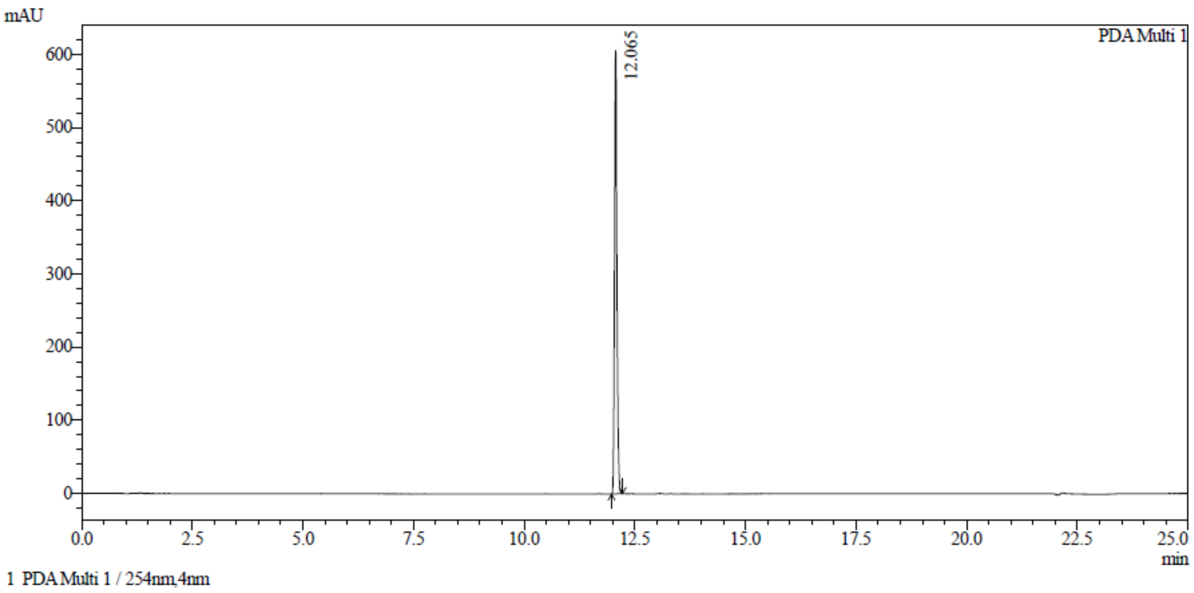

Integration result

PeakTable

PDA Ch1 254nm

Peak#	Ret. Time	USP Width	Resolution	Height	Area	Area %
1	12.065	0.099	0.000	606036	2294678	100.000
Total				606036	2294678	100.000

Compound 27

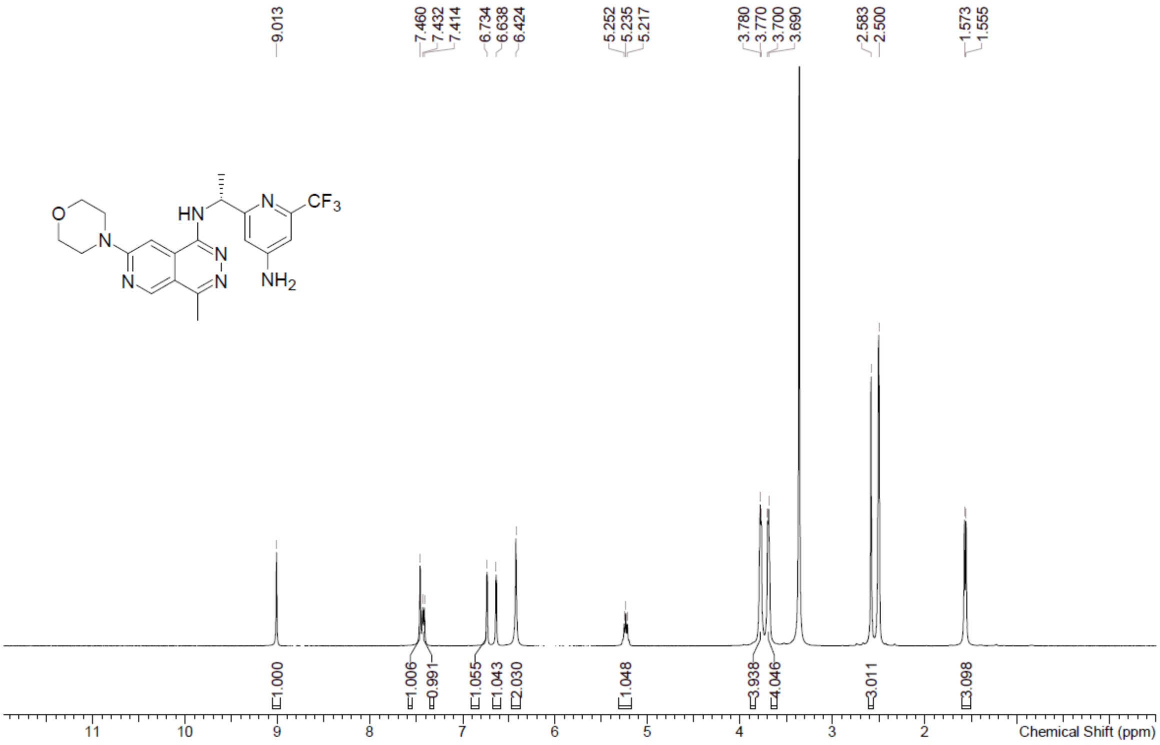

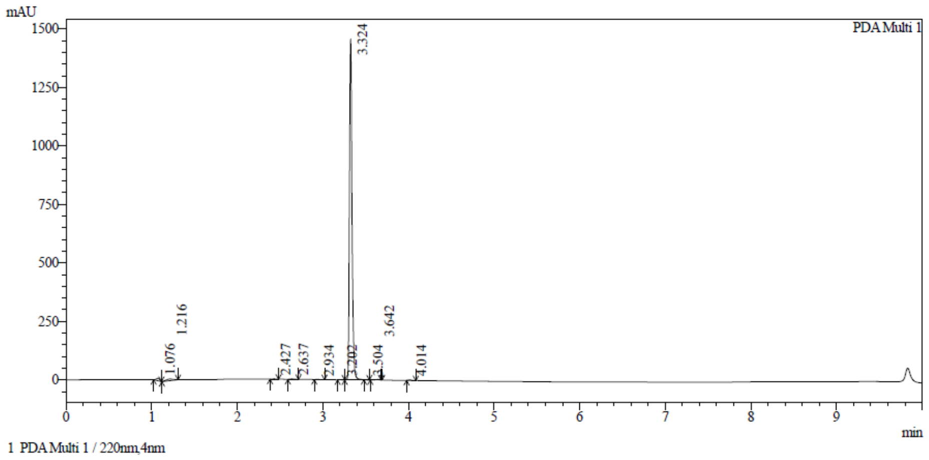

Integration result

PeakTable

PDA Ch1 220nm

Peak#	Ret. Time	USP Width	Resolution	Height	Area	Area %
1	1.076	0.068	0.000	10792	27724	0.882
2	1.216	0.144	1.318	7414	39059	1.243
3	2.427	0.057	12.034	1921	4000	0.127
4	2.637	0.074	3.217	2561	7086	0.226
5	2.934	0.056	4.578	1096	2570	0.082
6	3.202	0.059	4.664	808	1792	0.057
7	3.324	0.056	2.120	1438222	3048274	97.006
8	3.504	0.049	3.456	684	1103	0.035
9	3.642	0.078	2.184	1933	5581	0.178
10	4.014	0.058	5.503	2360	5161	0.164
Total				1467791	3142352	100.000

Compound 28

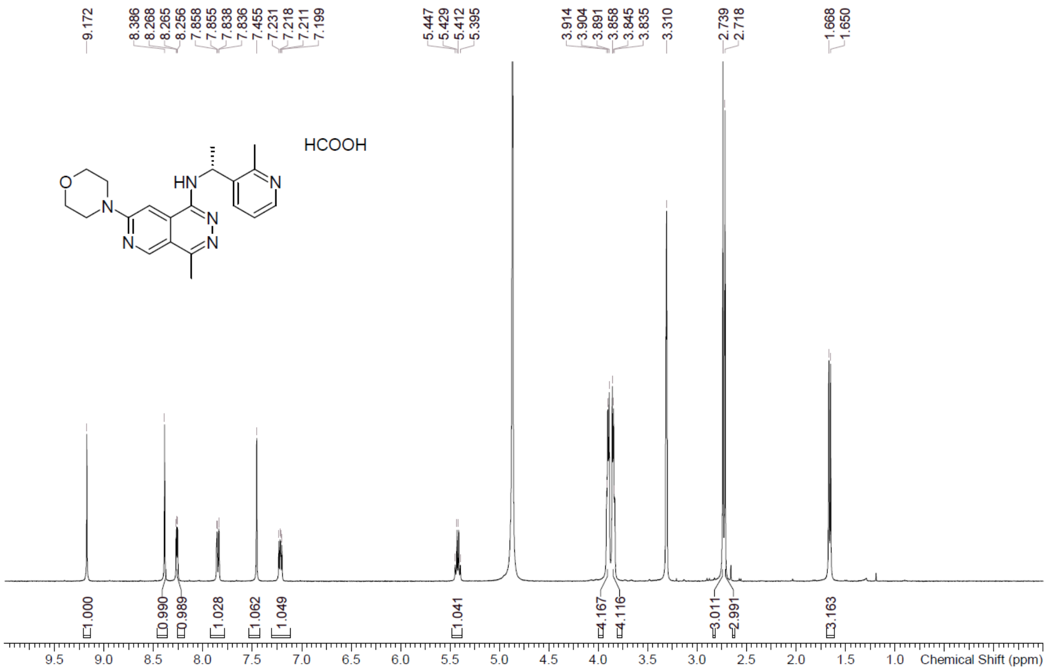

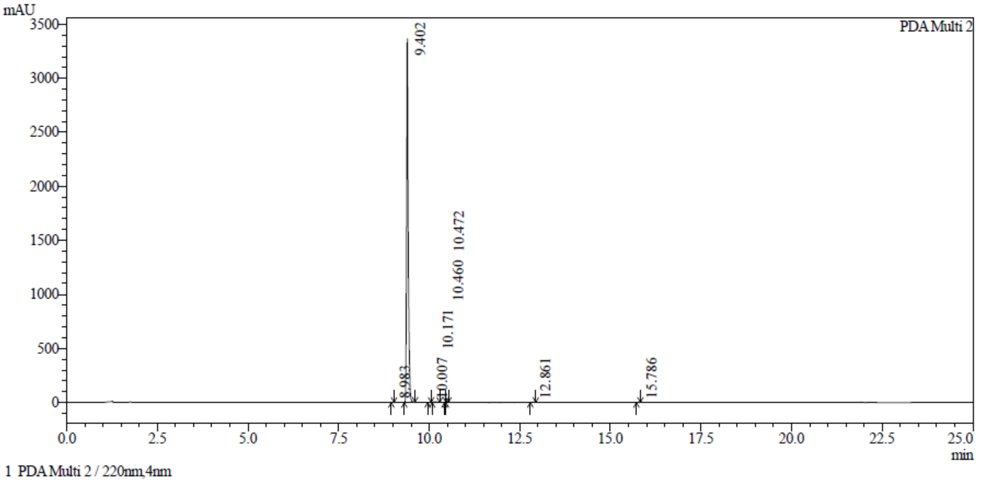

Integration result

PeakTable

PDA Ch2 220nm

Peak#	Ret. Time	USP Width	Resolution	Height	Area	Area %
1	8.983	0.075	0.000	1150	3073	0.028
2	9.402	0.064	6.047	3367751	11061172	99.339
3	10.007	0.094	7.621	1237	3902	0.035
4	10.171	0.134	1.441	6841	35260	0.317
5	10.460	0.069	2.835	1502	2068	0.019
6	10.472	0.090	0.145	1675	3509	0.032
7	12.861	0.128	21.928	4347	19514	0.175
8	15.786	0.109	24.684	1583	6319	0.057
Total				3386086	11134818	100.000

Compound 29

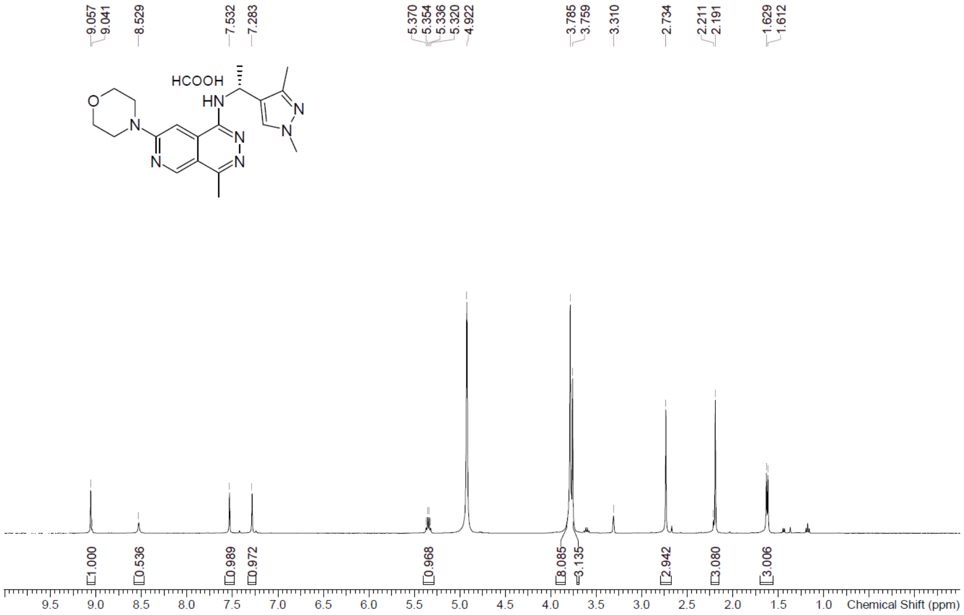

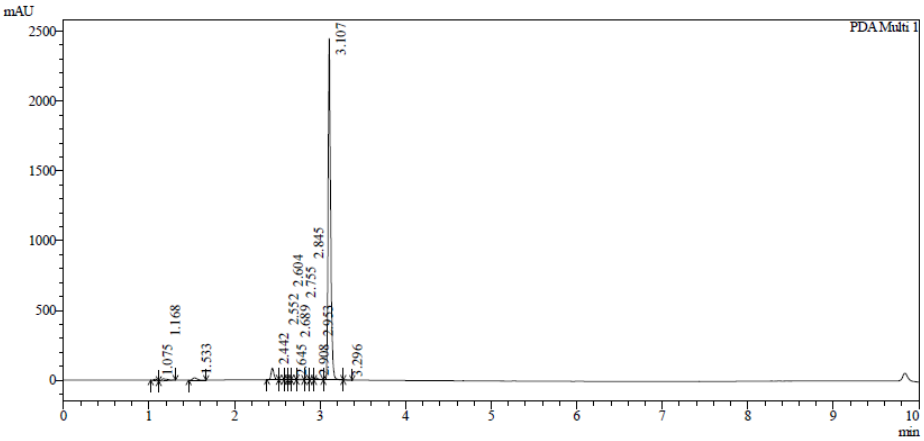

1 PDA Multi 1 / 220nm,4nm

Integration result

PeakTable

PDA Ch1 220nm

Peak#	Ret. Time	USP Width	Resolution	Height	Area	Area %
1	1.075	0.067	0.000	10351	25362	0.471
2	1.168	0.132	0.929	14372	67486	1.254
3	1.533	0.094	3.222	15922	58063	1.079
4	2.442	0.061	11.720	83447	192232	3.573
5	2.552	0.066	1.737	1005	2264	0.042
6	2.604	0.060	0.822	1040	1913	0.036
7	2.645	0.082	0.579	3138	5919	0.110
8	2.689	0.086	0.515	4157	10917	0.203
9	2.755	0.164	0.530	3862	18765	0.349
10	2.845	0.116	0.646	5420	16466	0.306
11	2.908	0.116	0.543	6638	15574	0.289
12	2.953	0.073	0.467	14549	42561	0.791
13	3.107	0.055	2.399	2421036	4918881	91.434
14	3.296	0.051	3.561	1639	3312	0.062
Total				2586578	5379716	100.000

Compound 30

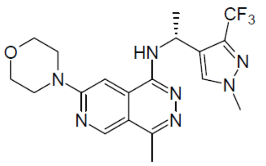

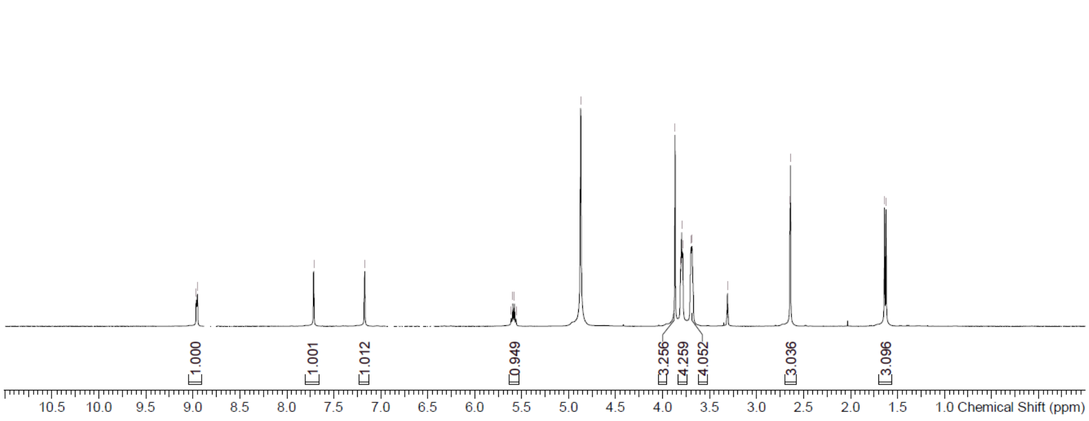

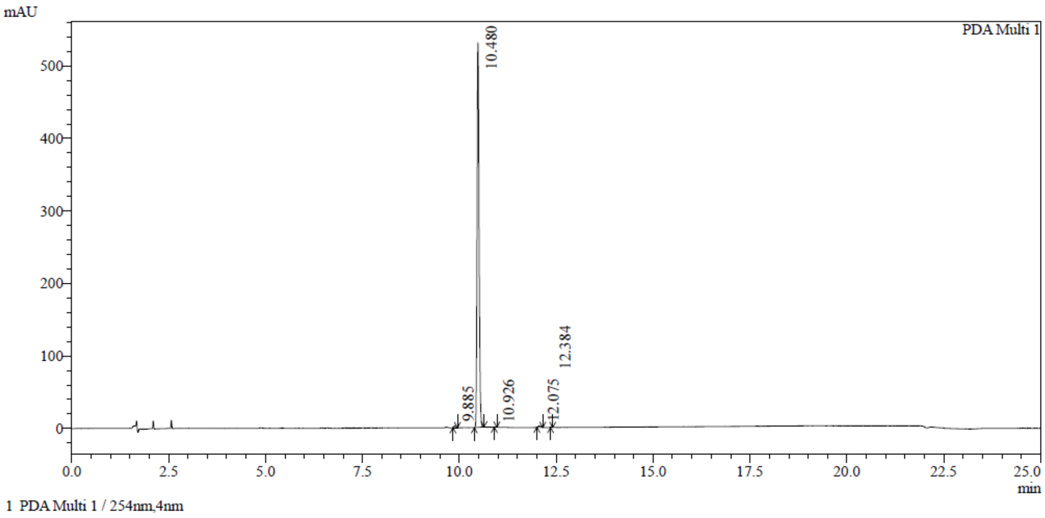

Integration result

PeakTable

PDA Ch1 254nm

Peak#	Ret. Time	USP Width	Resolution	Height	Area	Area %
1	9.885	0.088	0.000	2761	8941	0.454
2	10.480	0.097	6.441	530791	1949873	98.915
3	10.926	0.057	5.812	501	1126	0.057
4	12.075	0.130	12.307	2197	10278	0.521
5	12.384	0.131	2.374	500	1040	0.053
Total				536750	1971258	100.000

Compound 31

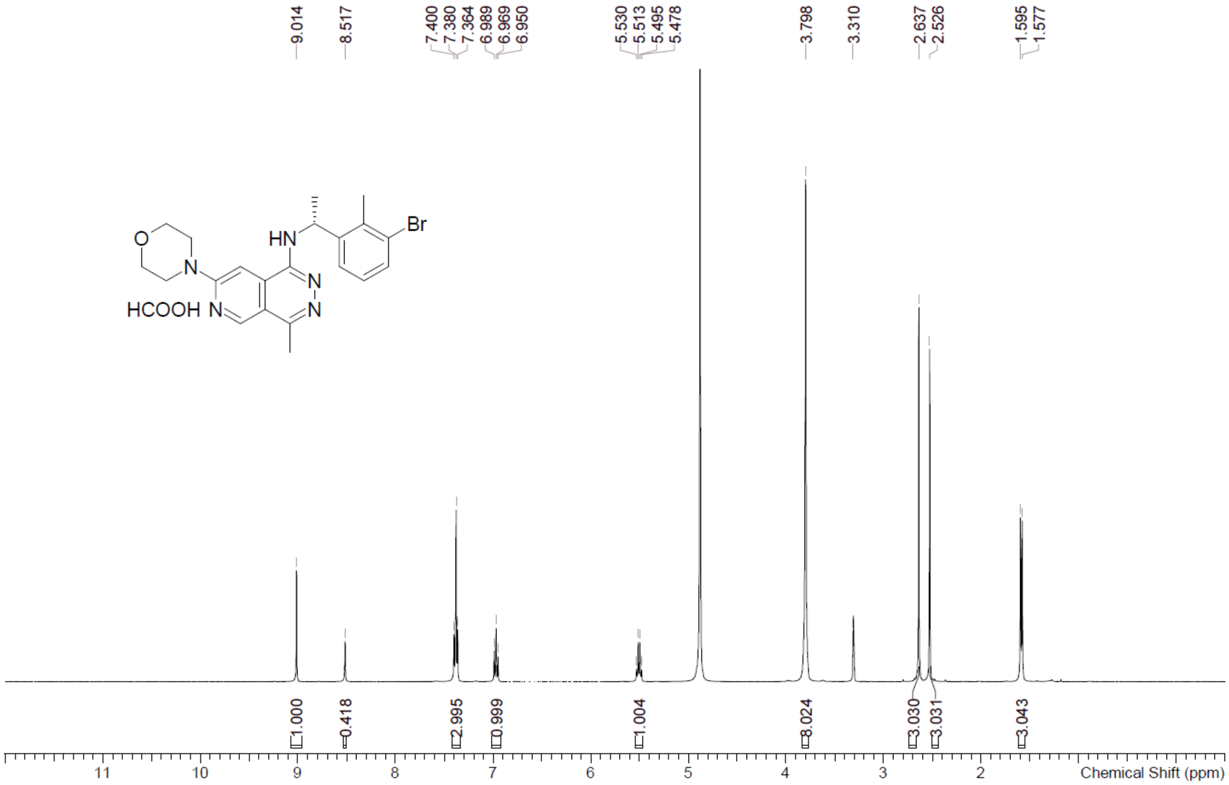

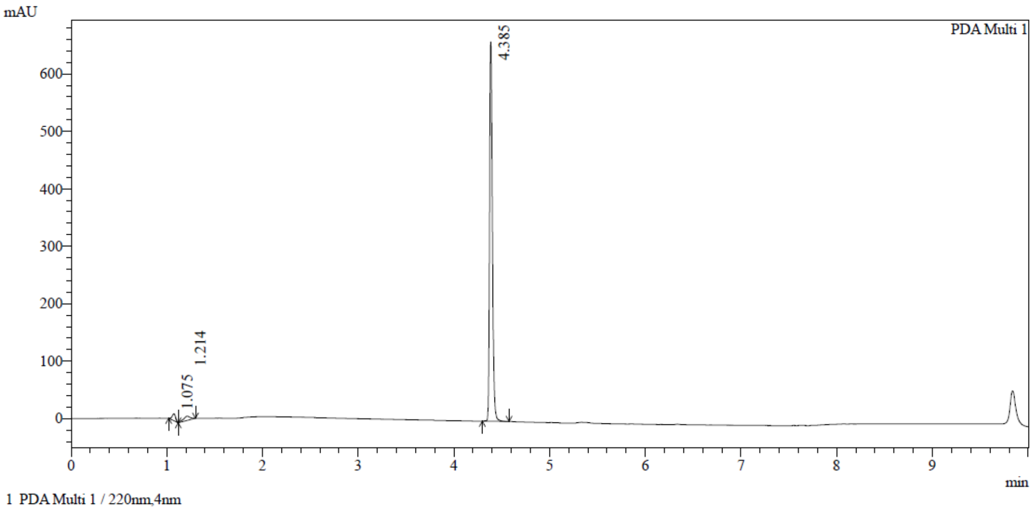

Integration result

PeakTable

PDA Ch1 220nm

Peak#	Ret. Time	USP Width	Resolution	Height	Area	Area %
1	1.075	0.065	0.000	12072	29534	2.054
2	1.214	0.128	1.437	7445	36933	2.569
3	4.385	0.055	34.784	657614	1371065	95.376
Total				677131	1437532	100.000

Compound 32

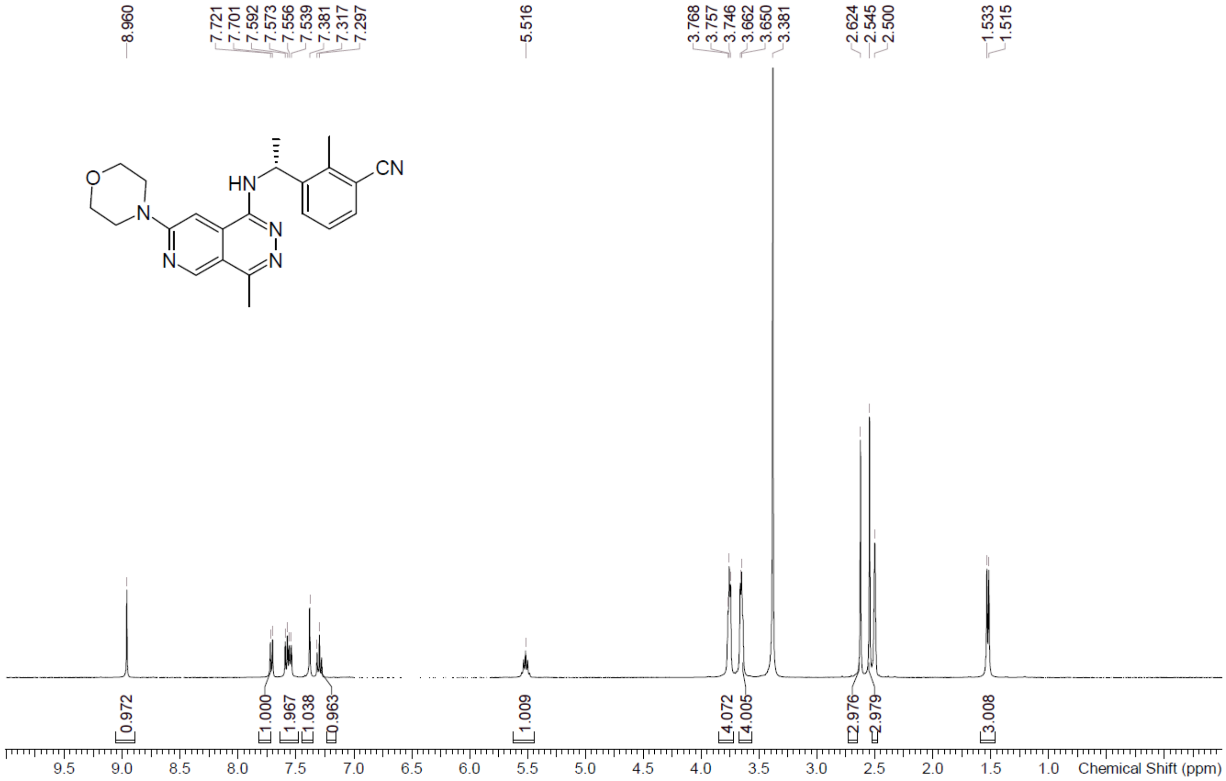

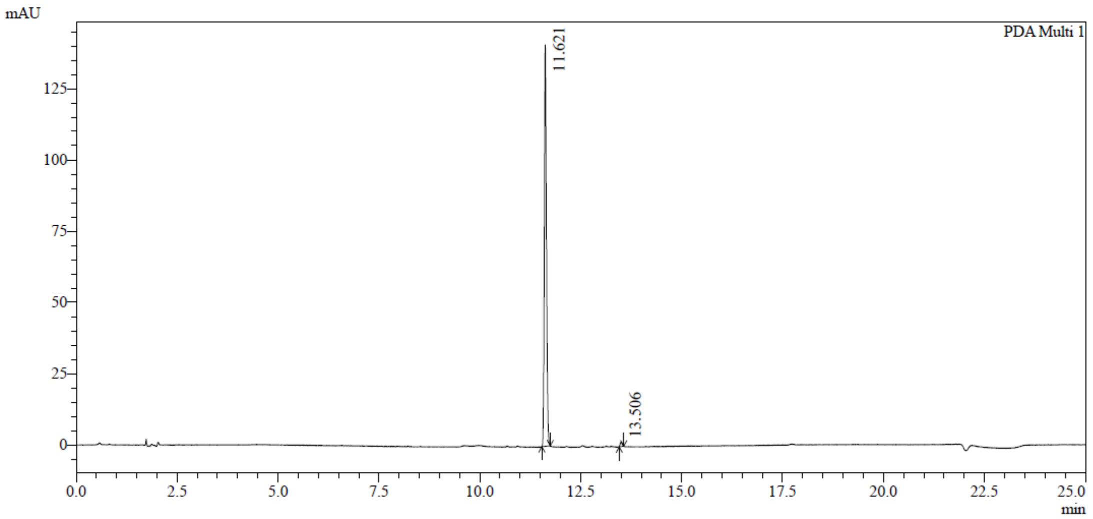

1 PDA Multi 1 / 254nm,4nm

Integration result

PeakTable

PDA Ch1 254nm

Peak#	Ret. Time	USP Width	Resolution	Height	Area	Area %
1	11.621	0.097	0.000	140892	518997	98.909
2	13.506	0.102	18.988	1621	5725	1.091
Total				142514	524723	100.000

Compound 33

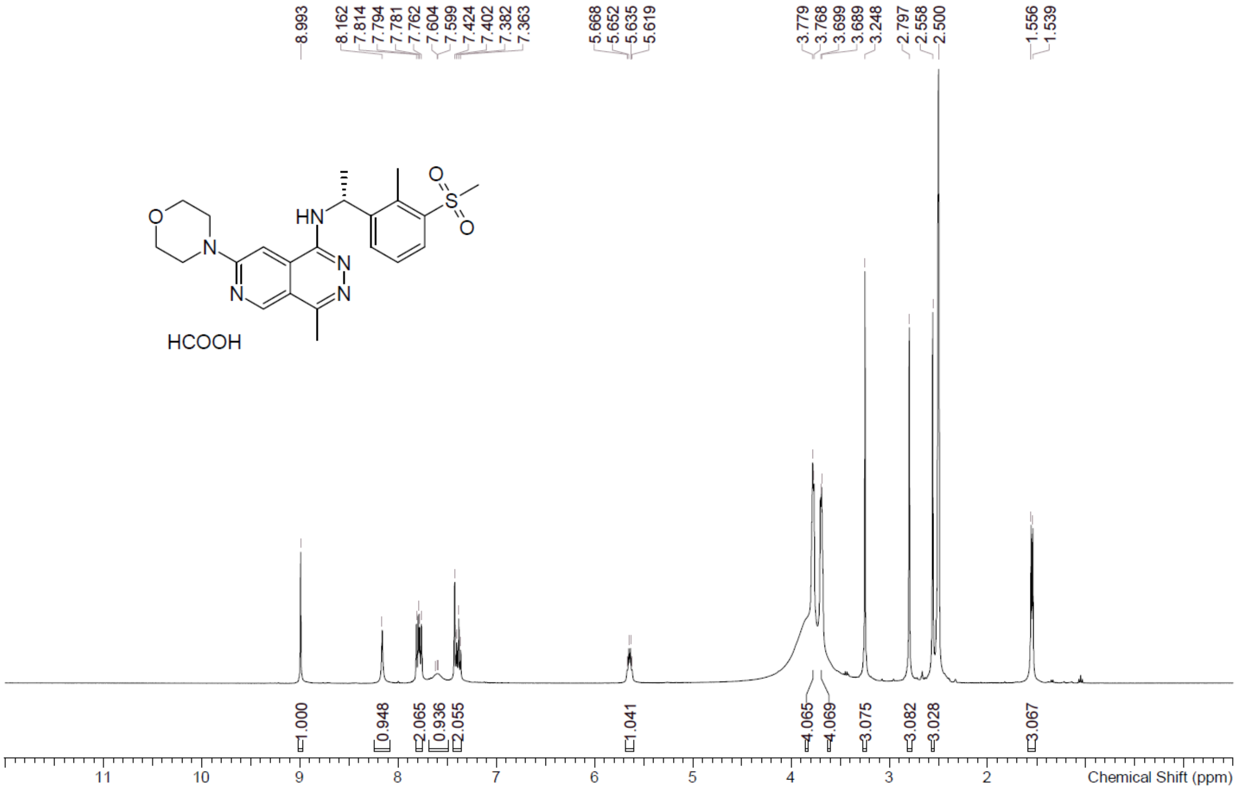

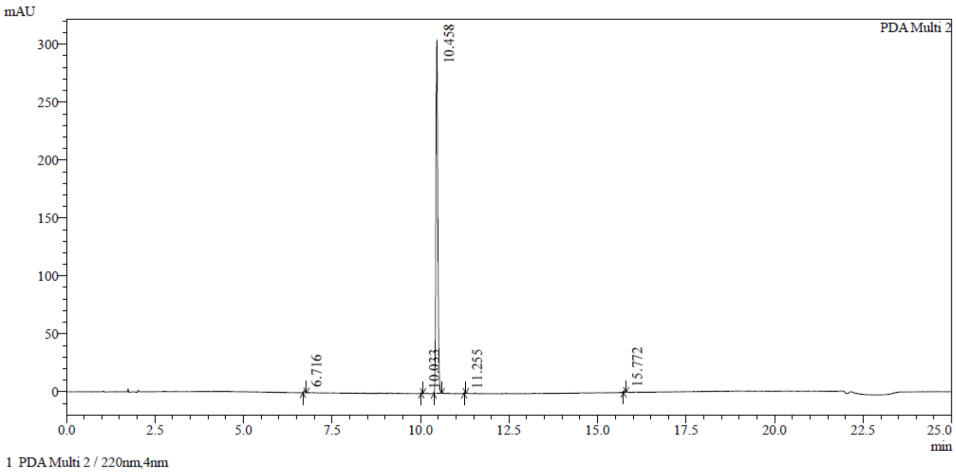

Integration result

PeakTable

PDA Ch2 220nm

Peak#	Ret. Time	USP Width	Resolution	Height	Area	Area %
1	6.716	0.077	0.000	1738	5184	0.467
2	10.033	0.048	53.139	661	1029	0.093
3	10.458	0.094	6.000	305296	1098316	98.866
4	11.255	0.078	9.269	676	1471	0.132
5	15.772	0.114	47.161	1465	4912	0.442
Total				309837	1110913	100.000

Compound 34

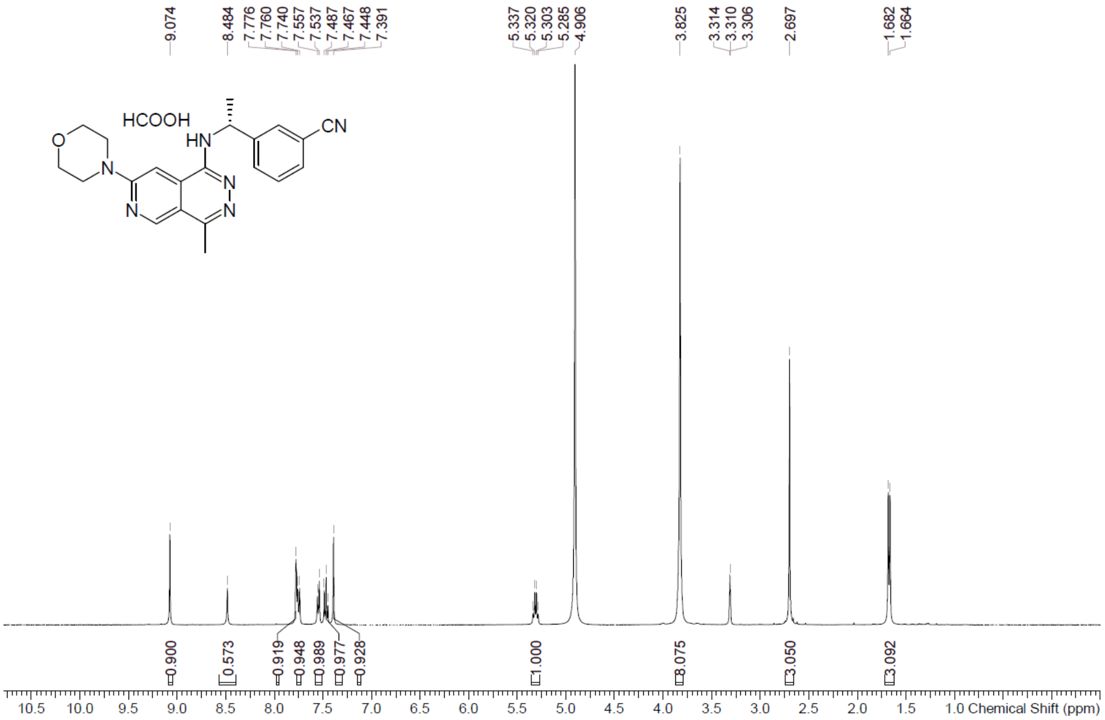

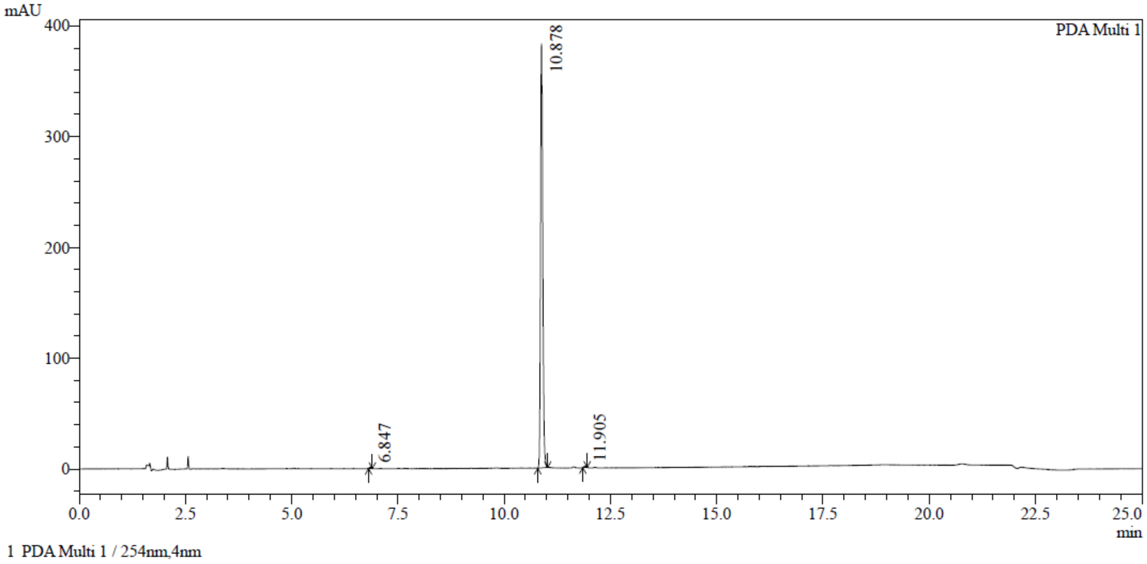

Integration result

PeakTable

PDA Ch1 254nm

Peak#	Ret. Time	USP Width	Resolution	Height	Area	Area %
1	6.847	0.073	0.000	1294	3392	0.239
2	10.878	0.097	47.577	382816	1411836	99.456
3	11.905	0.103	10.260	1184	4336	0.305
Total				385294	1419564	100.000

Compound 35

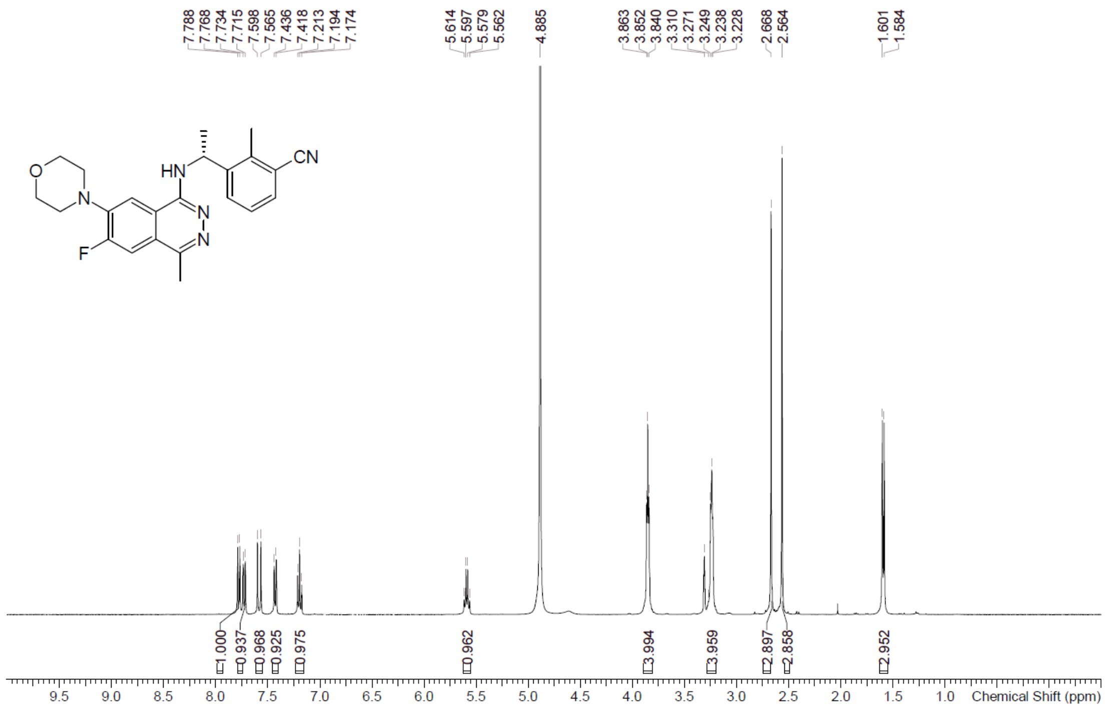

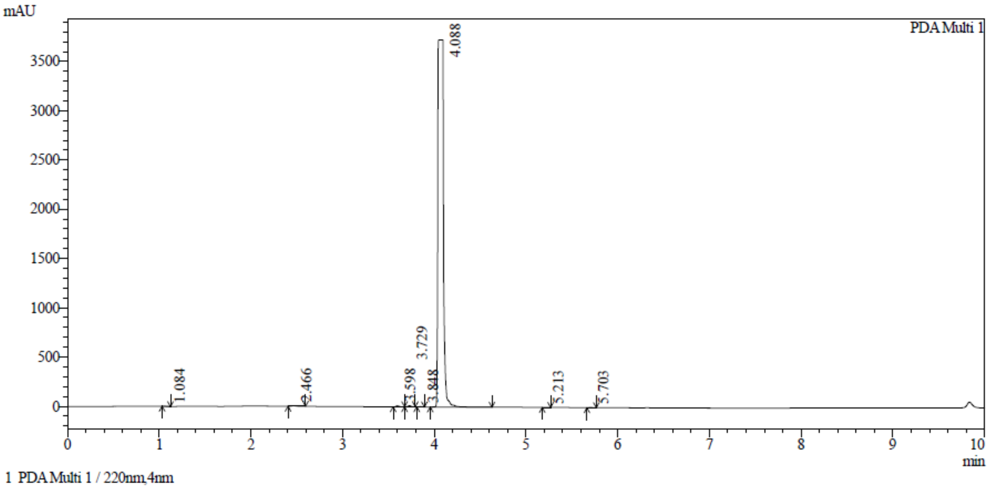

Integration result

PeakTable

PDA Ch1 220nm

Peak#	Ret. Time	USP Width	Resolution	Height	Area	Area %
1	1.084	0.074	0.000	3735	9336	0.061
2	2.466	0.089	16.965	5610	19017	0.123
3	3.598	0.057	15.485	9723	20851	0.135
4	3.729	0.060	2.238	11071	24901	0.162
5	3.848	0.054	2.101	3121	6136	0.040
6	4.088	0.074	3.755	3723057	15316518	99.438
7	5.213	0.060	16.745	1198	2578	0.017
8	5.703	0.071	7.477	1446	3770	0.024
Total				3758963	15403107	100.000

Compound 36

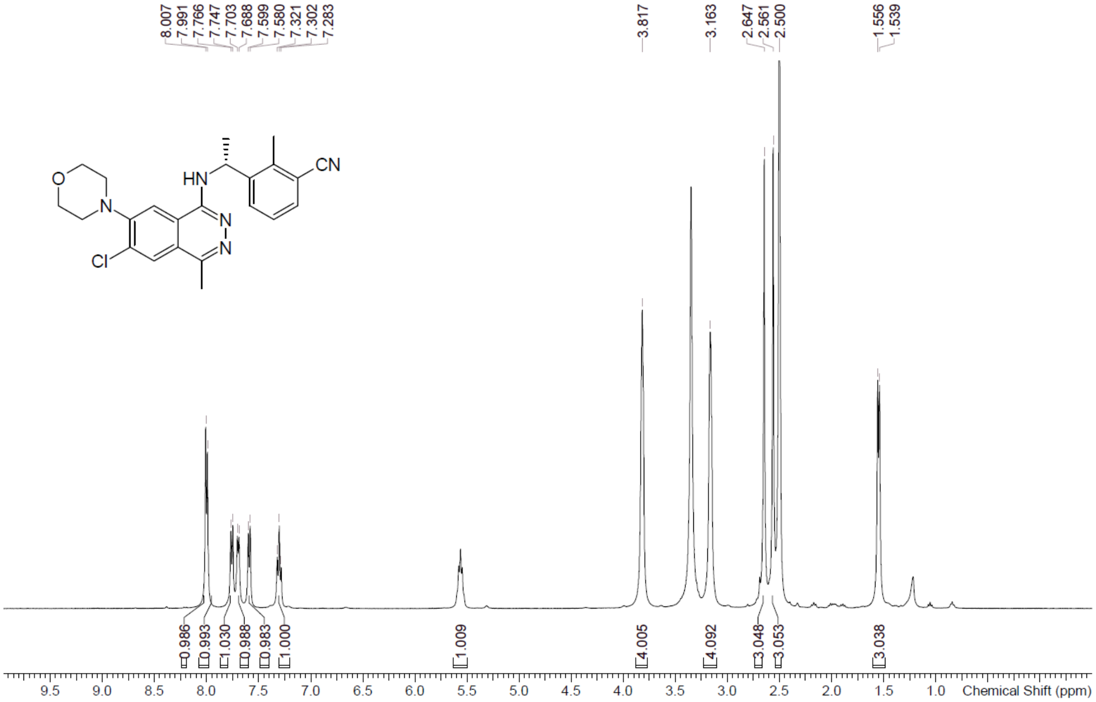

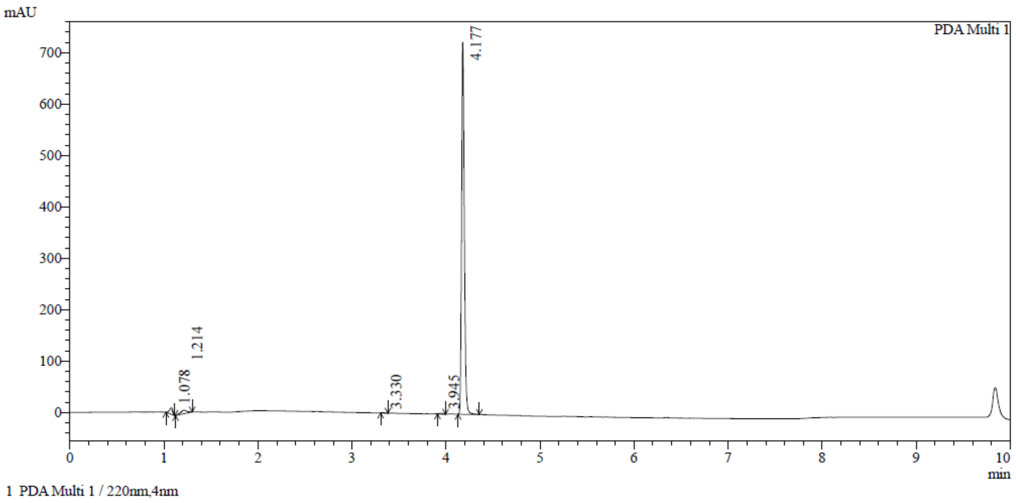

Integration result

PeakTable

PDA Ch1 220nm

Peak#	Ret. Time	USP Width	Resolution	Height	Area	Area %
1	1.078	0.066	0.000	12252	30358	1.955
2	1.214	0.125	1.424	7042	34290	2.208
3	3.330	0.047	24.529	804	1436	0.092
4	3.945	0.064	11.054	1520	3394	0.219
5	4.177	0.054	3.940	717797	1483688	95.527
Total				739416	1553165	100.000

Compound 37

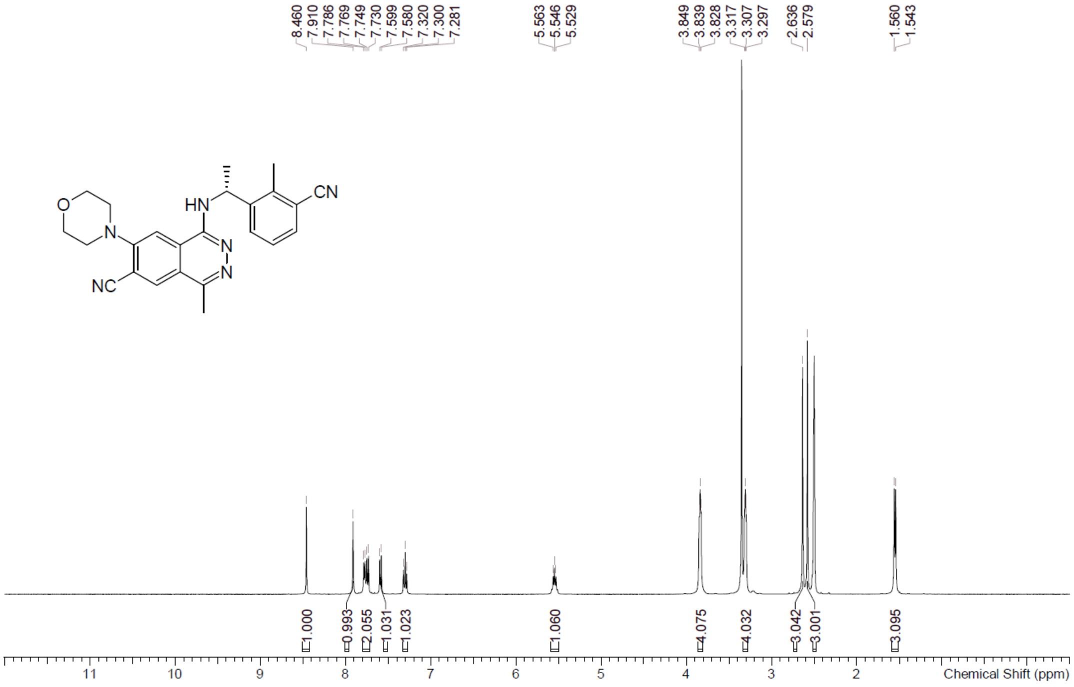

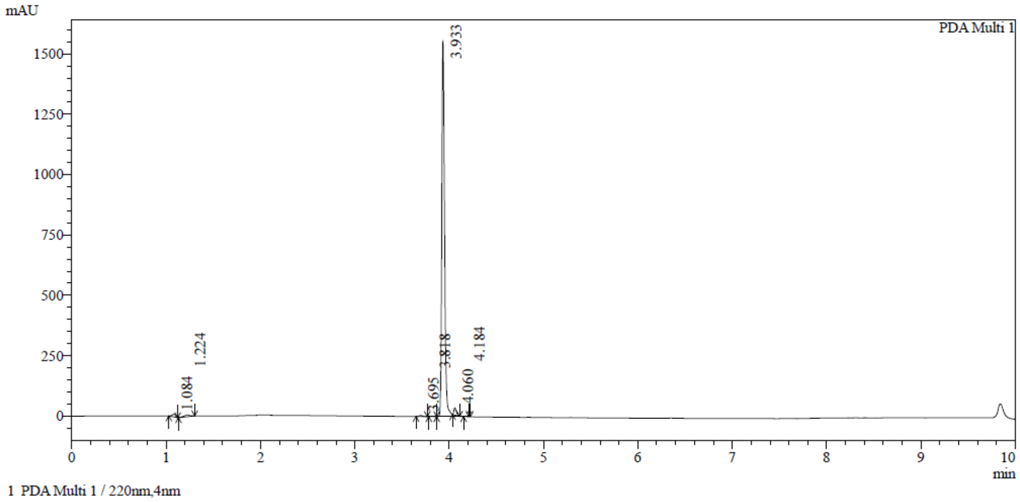

Integration result

PeakTable

PDA Ch1 220nm

Peak#	Ret. Time	USP Width	Resolution	Height	Area	Area %
1	1.084	0.070	0.000	10804	28713	0.855
2	1.224	0.121	1.470	7153	33736	1.005
3	3.695	0.059	27.519	4804	10788	0.321
4	3.818	0.052	2.226	1011	2011	0.060
5	3.933	0.054	2.180	1551055	3227152	96.134
6	4.060	0.049	2.457	29774	53470	1.593
7	4.184	0.040	2.782	581	1047	0.031
Total				1605182	3356917	100.000

Compound 38

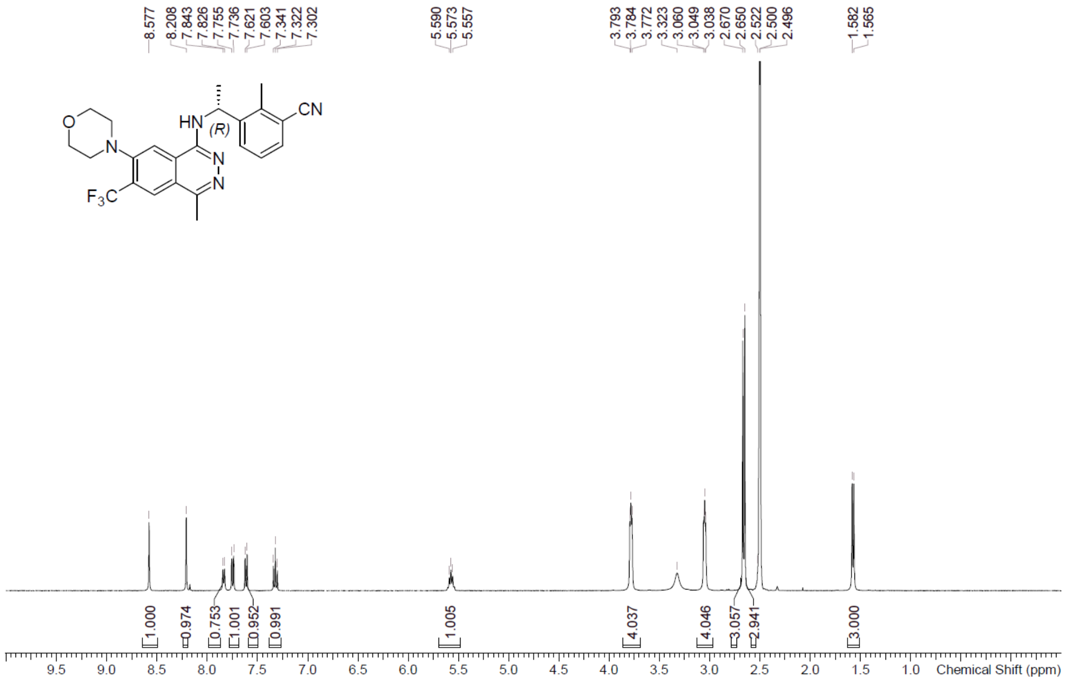

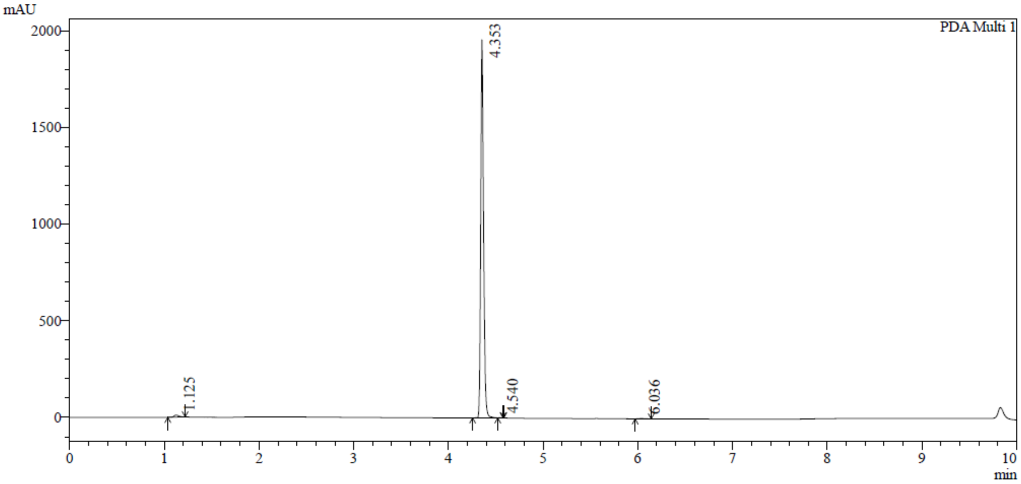

1 PDA Multi 1 / 220nm,4nm

Integration result

PeakTable

PDA Ch1 220nm

Peak#	Ret. Time	USP Width	Resolution	Height	Area	Area %
1	1.125	0.098	0.000	9774	37384	0.890
2	4.353	0.057	41.648	1947583	4154915	98.895
3	4.540	0.045	3.636	930	1459	0.035
4	6.036	0.108	19.473	1774	7572	0.180
Total				1960061	4201331	100.000

HTRF Displacement Assay to Determine K*i*

Measured the ability of a compound to bind to SOS1 was measured using a HTRF displacement assay. A recombinant human SOS1 polypeptide (corresponding to amino acids 560-1049, expressed in E. Coli with N-terminal His-TEV-AviTag-SOS1 (MW=59.4 kDa) and lanthanide labeled streptavidin (CisBio) was incubated with an exemplary compound (in a DMSO stock solution) in buffer (25 mM HEPES pH 7.5, 25 mM NaCl, 1 mM DTT, 0.01% Brij 35, 0.02% BSA, 0.1% DMSO). After a 10-15 minute incubation at room temperature, a solution comprised of a custom-made Cy5 labelled tracer and MAb Anti-6HIS Tb cryptate Gold (Cisbio 61HI2TLA) in buffer was added to the solution containing the SOS1 polypeptide and exemplary compound. After a 1-hour incubation at room temperature, the HTRF signal was measured using Clairostar plate reader (BMG Labtech) according to the manufacturer's instructions. Excitation filter EX-TR was used, and emission 1 was detected at 650-610 nm and emission 2 detected at 620-610 nm. The HTRF ratio was calculated using the formula: [emission 1/emission 2]*10000. Background signals were calculated from well with a 10μM inhibitor, known to inhibit 100% at that concentration. The background subtracted signals were converted to % binding relative to DMSO controls. Data were analyzed using XLFIT software (IDBS) using a Morrison equation for competitive binding and Ki's were generated.

MKN1 pERK ICW Assay

MKN1 cells (15,000/w) were seeded in a black clear flat bottom 96-well cell culture plate (Corning, #3904) and incubated at 37°C overnight. Assay day 1, cells were dosed with compounds with a 10 μm starting concentration and serially diluted 3x for a total of 9 concentrations. The cells were incubated for approximately 0.5-1 hour with the compounds solubilized in DMSO at 37 °C. Cells were immediately fixed by adding 50 μL of 4% formaldehyde to all wells in a fume hood and the plates were incubated for 20 minutes at room temperature. The formaldehyde was discarded from the plates and 150 μL of ice-cold methanol was added to permeabilize the cells for 10 minutes at -20 °C. The methanol was discarded from each of the plates and any liquid remaining in the plate by tapping the plate against paper towels. Cells were then blocked with 150 μL of Odyssey blocking buffer (LI-COR Biosciences #927-50010) using 0.05% Tween for 1 hour at room temperature on a shaker. The blocking buffer was discarded and 50 μL of primary antibodies pERK (cell signaling Technology #9101L; Rabbit, 1:500) and GapDH (Millipore #MAB34;

Mouse,1:5000) diluted in Odyssey blocking buffer was added. The plates were incubated overnight at 4 °C on a shaker.

On Assay day 2, the primary antibody solution was removed. Each plate was washed 3x times with 150 µL of 1x PBST (PBS + 0.1 % Tween 20) and incubated with 50 µL of secondary antibodies: Anti-Rabbit (LI-COR Biosciences #926-32211) and Anti-Mouse (LI-COR Biosciences #68070) at 1:800 dilution in Odyssey blocking buffer with Tween at room temperature on a shaker for 2 hours (protected from light). The secondary antibody solution as removed and each plate was washed with PBST 3x times. Any liquid remaining was discarded, and the plate was imaged using the Licor Odyssey machine according to the manufacturer's instruction, using a set focus length at 3mm and both 800nm and 700nm filters. The GAPDH normalized scan values for each well were divided by the average of vehicle wells to get the % of pERK inhibition. The IC_{50} values were then calculated with the Graph pad Prism software.

EGFR Selectivity Assay

EGFR selectivity was profiled using Reaction Biology's radiometric HotSpot kinase assay with human EGFR. Compounds were sent as a powder then suspended as a 10 mM DMSO stock solution. Compounds were tested in 10-dose IC_{50} mode with a 3-fold serial dilution starting at 10 µM. Control compound, staurosporine, was tested in 10-dose IC_{50} mode with 4-fold serial dilution starting at 20 µM. Reactions were carried out at 10 µM ATP.

SOS2 KRAS WT GDP Exchange Assay

The final conditions for the Functional SOS2 Assay are as follows: 50 mM HEPES 7.5, 2nM SOS2, 50nM GTP-CY5, 30nM KRAS, 0.5 nM Tb-SA, 5 mM $MgCl_2$, 1 mM TCEP, 0.2 mg/mL BSA, ~1.0% DMSO. Recombinant human SOS2 polypeptide (Accelagen Inc., corresponding to amino acids 558-1047, expressed in *E. coli* with a N-terminal HIS-tag MW=59.3 kDa) and Cy5-GTP (Jena Bioscience Inc) were added to an exemplary compound (in a DMSO stock solution) at room temperature for 15 minutes. Then recombinant human KRAS polypeptide (Accelagen Inc., amino acids 2-169, expressed in *E. coli* with a C-terminal Avi-biotinylated tag MW 22.0 kDa) was added with Terbium-StrepAvidin (Cisbio Inc.) and the Ratio metric data is collected after 30 minutes using a BMG LABTECH CLARIOstar Plus via TR-FRET. 100 percent of control (POC) is determined by using a DMSO control and 0 POC is determined using a concentration of control

compound that completely inhibits activity of SOS2. The POC values were fit to an IC_{50} with Hill equation and the IC_{50} value reported.

$$\text{Equation 1: } Vmax/(1+((x/IC_{50})\text{\^{}}Hill)))+BKD)$$

AO Metabolism Assay

Pooled human liver S9 fraction with high level of AO lot 1710129 were obtained from Xenotech. Human liver S9 was incubated at a final protein concentration of 0.5 mg/ml in phosphate buffer (100 mM, pH 7.4) with 1 µM test compounds or controls (phthalazine and zaleplon). Incubations were conducted with and without raloxifene (25 µM final concentration), a known AO inhibitor. No cofactors were added to any incubations. Incubations (200 µL total volume) were conducted in 96 deep well plates (Thomson Instruments, Oceanside, CA) using a thermomixer (Eppendorf, Hamburg, Germany) at 37°C with a shaking speed of 600 rpm. Aliquots (25 µL) were removed at 0, 2, 4, 8, 15, and 30 minutes and quenched with 100 µL of chilled acetonitrile containing 50 nM labetalol as internal standard. All collected samples were centrifuged at 4000 g for 10 minutes, and supernatants were diluted 10-fold with water before injected for liquid chromatography mass spectroscopy/mass spectroscopy (LC-MS/MS) analysis. For each incubation, a plot of percent parent drug remaining versus time was constructed. For some experiments, data points were removed from curve fitting if <25% of the parent drug was remaining. The $T_{1/2}$ was calculated using Equation 1 below, where A is the initial concentration (100%), k is the first-order rate constant, and t is the time in minutes. To determine k, data points were fit to an exponential decay model (model 500) in XLfit 5.5.0.5 as an add-in to Microsoft Excel for Microsoft 365 MSO (16.0.13127.21624) 64-bit (Redmond, WA). For all experiments, regression was performed with freely-floating t = 0 minute data points. The $T_{1/2}$ was calculated to be the time when percent remaining = 50%.

$$\text{Equation 1:} \qquad T_{(1/2)} = Ae^{(-kt)}$$

Tumor Pharmacodynamic and Tumor Xenograft Studies

Dulbecco's Modified Eagle Medium (#10566-016), penicillin and streptomycin (#15070-063), HEPES [(4-(2-hydroxyethyl)-1-piperazineethanesulfonic, acid); #15630-080], and Dulbecco's

Phosphate-Buffered Saline (#14190-136) were obtained from Gibco/Thermo Fisher Scientific. Fetal Bovine Serum (FBS) was obtained from Corning (#35-011-CV).

The MIA PaCa-2 cell line (ATCC Cat.# CRL-1420) was cultured in Dulbecco's Modified Eagle Medium with 10% FBS and 1% Penicillin-Streptomycin (5,000 U/mL) Solution and maintained at 37 °C in a humidified incubator at 5% CO_2. The MiaPaca-2 cell line was cultured 1:10 biweekly and flasks of cells were carried for no more than 10-15 cell passages in this work. Cells were harvested from confluent T225 flasks (Fisher Scientific, #10-126-63) for implants. Prior to implant for *in vivo* efficacy studies, the MIA PaCa-2 cell line was tested and confirmed negative for *Mycoplasma* and pathogens by IMPACT 1 assessment (IDEXX BioAnalytics).

The following antibodies were used at the indicated dilution: phospho-p44/42 MAPK (ERK1/2 Thr202/Tyr204) (1:500; Cell Signaling Technologies #9101L), p44/42 MAPK (ERK1/2) (1:500; Cell Signaling Technologies #9102L), GAPDH (1:5000; EMD Millipore MAB374), α-Tubulin (DM1A) Mouse mAb #3873 (1:10000; Cell Signaling Technologies #3873S), Beta Actin (1:2000, Abcam #AB8227), IRDye® 680RD (1:10,000; LI-COR Biosciences Catalog #926-68070), and IRDye® 800CW (1:10,000; LI-COR Biosciences Catalog #926-32211)

All mouse studies were conducted in compliance with all applicable regulations and guidelines of the Institutional Animal Care and Use Committee (IACUC) from the National Institutes of Health (NIH). Mice were maintained under pathogen-free conditions, and food and water was provided *ad libitum*. 6 – 8-week-old female Hsd:Athymic Nude-*Foxn1^{nu}* mice (Envigo, San Diego) were injected subcutaneously with tumor cells in 100 μl of PBS and Matrigel matrix (Corning #356237; Discovery Labware, MA) in the right hind flank of each mouse with 5.0 x 10^6 MIA PaCa-2 cells at a ratio of 1:1 in PBS and Matrigel (Corning #356237). Mouse health was monitored daily, and caliper measurements began when tumors were palpable. Tumor volume measurements were determined utilizing the formula 0.5 x L x W^2 in which L refers to length and W refers to width of each tumor. When tumors reached the desired average study start tumor volume of 150 mm^3 or 200 mm^3 for 21–28-day TGI efficacy and 6-day studies, respectively, mice were randomized into treatment groups. MRTX0902 was formulated in 0.5% Methylcellulose (4000cps) + 0.2% Tween80 in water once per week and dosing solution was stored protected from light at 4 °Celsius. MRTX849 was formulated once per week in 10% Captisol in 50 mM Citrate Buffer pH 5.0 and

dosing solution was stored protected from light at 4 °Celsius. Mice were orally administered vehicle, MRTX0902, MRTX849 orally (PO) at the indicated doses and schedules. Mice were monitored daily, tumors and body weights were measured 2 or 3 times per week. Percent Tumor Growth Inhibition (% TGI) was calculated using the following formula: (1-(Final Drug Treated Tumor Volume – Initial Drug Treated Tumor Volume) / (Final Vehicle Treated Tumor Volume – Initial Vehicle Treated Tumor Volume))*100. Percent Tumor regression was calculated when the average tumor volume of final treated tumors was less than initial treated tumor volume using the following equation: (-100%)*(1-(Final treated tumor volume)/(Initial treated tumor volume)). Statistical analysis of differences in mean tumor volume between vehicle- and drug-treated cohorts was run using a two-tailed Student t test in GraphPad Prism version 8.2.0. A *p* value of less than 0.05 was considered to be statistically significant.

In studies where tumor collection was performed, mice were humanely sacrificed, and tumors were surgically removed and immediately cut into two pieces. One half piece was transferred to a pre-filled homogenizer tube with ceramic beads (Fisher Scientific, #15-340-154) and the other half piece was transferred to an Eppendorf tube. Both tubes were immediately submerged in liquid nitrogen to snap freeze the tissue and stored at -80°C until further processing was performed.

Immunoblotting and Densitometric Analysis

To the tumor samples frozen with ceramic beads, an equal volume of ice cold 1X Lysis/Binding/Wash Buffer from Active Motif's GTPase Ras ELISA kit (Cat. #52097, Active Motif) was added, and tumors were homogenized using the MP FastPrep-24 homogenizer (MP Biomedicals) with high-speed shaking 3 – 5 times for 20 seconds while keeping the tumor lysate on ice between cycles. After homogenization, tubes were spun at 15,000 rpm for 10 minutes at 4°C and supernatant was collected. Protein concentrations of each lysate sample were determined using a Pierce BCA protein assay kit (#PI23227; Thermo Fisher Scientific) per the manufacturer's instructions.

Approximately 30 μg of total protein was added to 1X Lysis/Binding/Wash Buffer (Cat. # 52097; Active Motif) with 10X reducing agent (Cat. #NP0009; Invitrogen) and 4X XT Sample Buffer (Cat. #1610791; Bio-Rad) and boiled for 5 minutes. Processed samples were then loaded onto a 12% Criterion™ XT Precast Gel (Cat. #345-0118; Bio-Rad) using MOPS 1X Running Buffer

(#161-0788; Bio-Rad). Proteins were transferred from the gels to a nitrocellulose membrane using the iBlot 2 Dry Blotting System (Cat. #IB23001; Thermo Fisher Scientific). Afterwards, membranes were blocked with LI-COR Odyssey TBS Blocking Buffer (Cat. #927-50000; LI-COR) for 1 hour at room temperature on a rocking platform. Primary antibodies were diluted in LI-COR Blocking Buffer and incubated (on a rocking platform) overnight with the blot at 4°C. Membranes were then washed with Tris-buffered saline-Tween 20 (TBS-T), incubated with LI-Cor IR Dye secondary antibodies for 1 hour at room temperature, and subsequently washed for a final time (3X, 10 minutes) with TBS-T.

Images were acquired from probed nitrocellulose membranes using the LI-COR Odyssey CLx Imaging system (LI-COR, Lincoln, NE) set to the AutoScan channel for both the 700 and 800 wavelength channels to measure the signal intensity from the IRDye 680RD goat anti-rabbit and IRDye 800CW goat anti-mouse secondary antibodies, respectively. Images were imported into LiCor's Image Studio software version 4.0 and then .tif files were exported for annotation. To quantify the pixel intensity for each selected protein band, the "Add Rectangle" tool in the image viewer was used to identify a consistently sized area of interest for each band of a given target protein as well as a representative background region of the immunoblot. The signal output column from the software subtracts background pixel intensity and was used to determine the target pixel intensity for each protein band. This corrected signal intensity was determined for each target protein of interest and data were exported to Excel.

Drug treatment is defined as either single agent administration of MRTX0902 or dual administration of MRTX0902 and MRTX849. Target protein sample loading normalization of each sample was determined by dividing the signal output of the target protein (pERK) by the signal output of the loading control protein (β-actin, α-tubulin, or ERK1/2). Each target protein was also averaged within each vehicle or drug treatment group. The vehicle value was normalized to "1" by dividing all average values by the vehicle value and standard deviation was calculated from the normalized values. To measure the degree of pERK phosphorylation, the normalized pERK signal for each sample was determined by dividing the pERK signal output by the signal output of the total ERK1/2 protein. Percent inhibition of normalized pERK in drug-treated tumors compared to vehicle-treated control tumors was calculated by dividing the average drug-treated tumor normalized pERK signal by the average vehicle-treated normalized pERK signal and

multiplying by 100. GraphPad Prism 8 was used to graph the data and determine statistical significance between the vehicle- and drug-treated cohorts using a two-tailed Student's t test, where a p value of less than 0.05 was considered to be statistically significant.

Co-crystal structure determination

Protein purification. The gene encoding His6-Tev-SOS1 (Uniprot Q07889, amino acids 564-1049) was codon optimized, synthesized, cloned into a pET24a expression vector and transformed into BL(21)DE3 *E*. coli cells. Protein expression was induced by addition of 0.4 mM IPTG for 4 hours at 30 °C. Cells were harvested and lysed with a microfluidizer in 25 mM Tris-HCL, pH 8.0, 500 mM NaCl, 1 mM TCEP with protease inhibitor tablets (Roche) at 4 °C. Cell debris was removed by centrifugation. SOS1 was purified from the supernatant using a HisTrap HP column and eluted with lysis buffer plus an imidazole gradient. Fractions containing SOS1 were combined, treated with TEV protease, and dialyzed overnight at 4 °C. SOS1 was further purified by using a HisTrap flow through step followed by a HiTrapQ column. Gel filtration was performed as a final purification step using an S200 (26/600) SEC column (Cytiva, Marlborough MA) equilibrated in 20 mM HEPES, pH 7.8, 100 mM NaCl, 1 mM TCEP. SOS1 was concentrated to 1.5 mg/mL, aliquoted, flash frozen, and stored at -80 C for to use for crystallization. Purity was confirmed by SDS-page analysis.

Crystallography. Purified SOS1 was defrosted, incubated with a 10-fold molar excess of inhibitor, and co-concentrated to 5 mg/mL. Initial co-crystals of SOS1 grew by vapor diffusion using a 1:1 mixture of protein and reservoir solutions containing 5-15% PEG 8000, 5-15% Ethanol, 100 mM Tris pH 8 at 8 C. Subsequent SOS1 crystals were grown from the same reservoir conditions with seeding. Crystals were harvested, cryo-protected in a solution of reservoir plus 20% Ethylene Glycol, and flash frozen in liquid nitrogen. Synchrotron x-ray diffraction data was collected on frozen crystals at the Advanced Photon Source (NE-Cat beamlines 23-ID-C and 23-ID-E). Data was indexed, processed, and scaled using XDS and aimless (Table S1). Structures were determined by molecular replacement (Phaser[1]) and refined over iterative rounds of automated refinement (Phenix refine[2]) and manual refitting in Coot.[3]

**Table S1**. SOS1 X-ray data collection and refinement statistics		
**Compound**	**Compound 15**	**MRTX0902 Compound 32**
**PDB ID**	7UKS	7UKR
**Data Collection**		
Beamline	APS-24-ID	APS-24-ID-E
Wavelength (Å)	0.97918	0.97918
Resolution (Å)[a]	50.0-2.29 (2.37-2.29)	50.0-2.5 (2.54-2.50)
Space Group	$P2_12_12_1$	$P2_1$
Unit Cell a, b, c (Å)	40.8, 89.1, 172.3	77.8, 148.2, 52.4
α, β, γ (°)	90, 90, 90	90, 108, 90
Unique Reflections	28762 (2766)	35219 (1179)
Redundancy	4.4 (4.3)	3.0 (2.6)
Completeness (%)	98.8 (98.6)	91.4 (61.9)
$< I/\sigma_I >$	13.8 (0.6)	7.2 (1.0)
R_{pim}[b]	0.04 (2.6)	0.09 (0.40)
$CC_{1/2}$[c]	0.99 (0.45)	0.99 (0.85)
**Refinement**		
R (%)[d] / R_{free} (%)[e]	24.4 / 27.5	24.1 / 28.9
B Average ($Å^2$)	75	57
RMSD Bond Lengths (Å)	0.001	0.002
RMSD Bond Angles (°)	0.4	0.6
**Ramachandran (%)**		
Favored	98.5	95.8
Outliers	0	0.6

[a]Numbers in parentheses refer to the highest resolution shell.

[b]$R_{pim} = \sqrt{\Sigma_{hkl} \frac{1}{(N_{hkl}-1)}} \times \Sigma_i |I_{hkl,i} - <I_{hkl}>| / \Sigma_{hkl}\Sigma_i I_{hk,i}$,

where $I_{hkl,i}$ is the scaled intensity of the ith measurement of reflection hkl and $<I_{hkl}>$ is the average intensity of that reflection.

[c]$CC_{1/2}$ = Pearson Correlation Coefficient between two random half datasets.

[d]$R = \Sigma_{hkl} | F_o - F_c | / \Sigma_{hkl} | F_o | \times 100$

[e]R_{free} was calculated as for R, but on a test set comprising 5% of the data excluded from refinement.

Table S2. MRTX0902 inhibition profile across DiscoverX (Eurofins) Safetyscan Panel

Target Class	Assay Name	Assay Target	Mode	Result Type	Value Prefix	RC50 (uM)
GPCR	Calcium Flux	ADORA2A	Agonist	EC50	>	10
GPCR	Calcium Flux	ADRA1A	Agonist	EC50	>	10
GPCR	Calcium Flux	AVPR1A	Agonist	EC50	>	10
GPCR	Calcium Flux	CCKAR	Agonist	EC50	>	10
GPCR	Calcium Flux	CHRM1	Agonist	EC50	>	10
GPCR	Calcium Flux	CHRM3	Agonist	EC50	>	10
GPCR	Calcium Flux	EDNRA	Agonist	EC50	>	10
GPCR	Calcium Flux	HRH1	Agonist	EC50	>	10
GPCR	Calcium Flux	HTR2A	Agonist	EC50	>	10
GPCR	Calcium Flux	HTR2B	Agonist	EC50	>	10
GPCR	Calcium Flux	ADORA2A	Antagonist	IC50	>	10
GPCR	Calcium Flux	ADRA1A	Antagonist	IC50	>	10
GPCR	Calcium Flux	AVPR1A	Antagonist	IC50	>	10
GPCR	Calcium Flux	CCKAR	Antagonist	IC50	>	10
GPCR	Calcium Flux	CHRM1	Antagonist	IC50	=	6.63988
GPCR	Calcium Flux	CHRM3	Antagonist	IC50	>	10
GPCR	Calcium Flux	EDNRA	Antagonist	IC50	>	10
GPCR	Calcium Flux	HRH1	Antagonist	IC50	>	10
GPCR	Calcium Flux	HTR2A	Antagonist	IC50	>	10
GPCR	Calcium Flux	HTR2B	Antagonist	IC50	>	10
GPCR	cAMP	ADRA2A	Agonist	EC50	>	10
GPCR	cAMP	ADRB1	Agonist	EC50	>	10
GPCR	cAMP	ADRB2	Agonist	EC50	>	10
GPCR	cAMP	CHRM2	Agonist	EC50	>	10
GPCR	cAMP	CNR1	Agonist	EC50	>	10
GPCR	cAMP	CNR2	Agonist	EC50	>	10
GPCR	cAMP	DRD1	Agonist	EC50	>	10
GPCR	cAMP	DRD2S	Agonist	EC50	>	10
GPCR	cAMP	HRH2	Agonist	EC50	>	10
GPCR	cAMP	HTR1A	Agonist	EC50	>	10
GPCR	cAMP	HTR1B	Agonist	EC50	>	10
GPCR	cAMP	OPRD1	Agonist	EC50	>	10
GPCR	cAMP	OPRK1	Agonist	EC50	>	10
GPCR	cAMP	OPRM1	Agonist	EC50	>	10
GPCR	cAMP	ADRA2A	Antagonist	IC50	>	10
GPCR	cAMP	ADRB1	Antagonist	IC50	>	10
GPCR	cAMP	ADRB2	Antagonist	IC50	>	10

GPCR	cAMP	CHRM2	Antagonist	IC50	>	10
GPCR	cAMP	CNR1	Antagonist	IC50	=	3.42989
GPCR	cAMP	CNR2	Antagonist	IC50	>	10
GPCR	cAMP	DRD1	Antagonist	IC50	>	10
GPCR	cAMP	DRD2S	Antagonist	IC50	=	4.03144
GPCR	cAMP	HRH2	Antagonist	IC50	>	10
GPCR	cAMP	HTR1A	Antagonist	IC50	>	10
GPCR	cAMP	HTR1B	Antagonist	IC50	>	10
GPCR	cAMP	OPRD1	Antagonist	IC50	>	10
GPCR	cAMP	OPRK1	Antagonist	IC50	>	10
GPCR	cAMP	OPRM1	Antagonist	IC50	>	10
Ion Channel	Ion Channel	CAV1.2	Blocker	IC50	>	10
Ion Channel	Ion Channel	GABAA	Blocker	IC50	>	10
Ion Channel	Ion Channel	hERG	Blocker	IC50	>	10
Ion Channel	Ion Channel	HTR3A	Blocker	IC50	>	10
Ion Channel	Ion Channel	KvLQT1/minK	Blocker	IC50	>	10
Ion Channel	Ion Channel	nAChR(a4/b2)	Blocker	IC50	>	10
Ion Channel	Ion Channel	NAV1.5	Blocker	IC50	>	10
Ion Channel	Ion Channel	NMDAR (1A/2B)	Blocker	IC50	>	10
Ion Channel	Ion Channel	GABAA	Opener	EC50	>	10
Ion Channel	Ion Channel	HTR3A	Opener	EC50	>	10
Ion Channel	Ion Channel	KvLQT1/minK	Opener	EC50	>	10
Ion Channel	Ion Channel	nAChR(a4/b2)	Opener	EC50	>	10
Ion Channel	Ion Channel	NMDAR (1A/2B)	Opener	EC50	>	10
Kinases	Binding	INSR	Inhibitor	IC50	>	10
Kinases	Binding	LCK	Inhibitor	IC50	>	10
Kinases	Binding	ROCK1	Inhibitor	IC50	>	10
Kinases	Binding	VEGFR2	Inhibitor	IC50	>	10
NHR	NHR Nuclear Translocation	AR	Agonist	EC50	>	10
NHR	NHR Nuclear Translocation	AR	Antagonist	IC50	>	10
NHR	NHR Protein Interaction	GR	Agonist	EC50	>	10
NHR	NHR Protein Interaction	GR	Antagonist	IC50	>	10
Non-Kinase Enzymes	Enzymatic	AChE	Inhibitor	IC50	>	10
Non-Kinase Enzymes	Enzymatic	COX1	Inhibitor	IC50	>	10
Non-Kinase Enzymes	Enzymatic	COX2	Inhibitor	IC50	>	10

Non-Kinase Enzymes	Enzymatic	MAOA	Inhibitor	IC50	=	8.88403
Non-Kinase Enzymes	Enzymatic	PDE3A	Inhibitor	IC50	>	10
Non-Kinase Enzymes	Enzymatic	PDE4D2	Inhibitor	IC50	>	10
Transporter	Transporter	DAT	Blocker	IC50	>	10
Transporter	Transporter	NET	Blocker	IC50	>	10
Transporter	Transporter	SERT	Blocker	IC50	>	10

pKa Determination

Sirius T3Dt (Sirius Analytical Instruments Ltd) fitted with combination Ag/AgCl pH electrode was used for the determination of dissociation constants. The pKa and psKa values were calculated by Sirius T3Dt software (Sirius Analytical Instruments Ltd, Version 1.0.12.120). There are two methods available on the Sirius T3 for pKa measurement: potentiometric titration and spectroscopic (UV) titration.

UV metric method: Pipetted 5 µL of sample stock solution (10 mM in DMSO) and 25 µL fast UV buffer into a sample vial, and 1.5 mL of 80%v/v MeOH was added into the sample vial automatically. Pre-acidified the sample solution with 0.5 M HCl by the instrument automatically, and then titrated three times from low to high pH. Refined the data to get aqueous pKa value.

pH metric method: About 1 mg of sample was weighed into a sample vial. 1.50 mL of 80%v/v MeOH was added into the vial automatically. The sample was solution pre-acidified with 0.5 M HCl by the instrument automatically, and then titrated three times from low to high pH. Refined the data to get aqueous pKa value.

Sirius T3 software always fits Yasuda-Shedlovsky data linearly, according to the equation.

Where:

- Yi is calculated as psKa + log [H_2O]
- psKa represents apparent pKa values of compounds measured in water/co-solvent mixtures
- [H_2O] represents the concentration of H_2O in mixtures solution
- Xi calculated as 1/ε i.
- ε i represents dielectric constant of water/co-solvent mixture

Table S3. pKa of MRTX0902

Cmpd	pKa (UV metric)	pKa (pH metric)	Final pKa result
MRTX0902	6.78	6.62	6.70

Caco-2 Assay:

Caco-3 cells (clone C2BBe1) were obtained from the American Type Culture Collection (Manassas, VA). Cell monolayers were grown to confluence on collagen-coated, microporous membranes in 12- well assay plates. The permeability assay buffer was Hanks' balanced salt solution containing 10 mM HEPES and 15

mM glucose at a pH of 7.4. The buffer in the received chamber also contained 1% bovine serum albumin. The dosing solution concentration was 10 µM of test article in buffer. Cell monolayers were dose on the apical side (A-to-B) or basolateral side (B-to-A) and incubated at 37 °C with 5% CO_2 in a humidified incubator. Samples were taken from the donor and receiver chambers at 120 minutes. Each determination was performed in duplicate. The flux of lucifer yellow was also measure post-experimentally for each monolayer to ensure no damage was inflicted to the cell monolayers during the flux period. All samples were assayed by LC-MS/MS using electrospray ionization. The apparent permeability (Papp) and percent recovery were calculated as follows:

$$P_{app} = (dC_r/dt) \times V_r/(A \times C_A) \quad (1)$$

$$\text{Percent Recovery} = 100 \times ((V_r \times C_r^{final}) + (V_d \times C_d^{final}))/(V_d \times C_N)$$

Where:

- dC_r/dt is the slope of the cumulative receiver concentration versus time in µM s^{-1};
- V_r is the volume of the receiver compartment in cm^3;
- V_d is the volume of the donor compartment in cm^3;
- A is the area of the insert (1.13 cm^2 for 12-well);
- C_A is the average of the nominal dosing concentration and the measured 120-minute
- donor concentration in µM;
- C_N is the nominal concentration of the dosing solution in µM;
- C_r^{final} is the cumulative receiver concentration in µM at the end of the incubation period;
- C_d^{final} is the concentration of the donor in µM at the end of the incubation period.

Efflux ratio (ER) is defined as P_{app} (B-to-A) / P_{app} (A-to-B).

References:

1. McCoy, A. J.; Grosse-Kunstleve, R. W.; Adams, P. D.; Winn, M. D.; Storoni, L. C.; Read, R. J. Phaser Crystallographic Software. *J. Appl. Cryst.* **2007**, *40*, 658-674.
2. Liebschner, D.; Afonine, P. V. ; Baker, M. L.; Bunkóczi, G.; Chen, V. B.; Croll, T. I.; Hintze, B.; Hung, L. W.; Jain, S.; McCoy, A. J.; Moriarty, N. W.; Oeffner, R. D.; Poon, B. K.; Prisant, M. G.; Read, R. J.; Richardson, J. S.; Richardson, D. C.; Sammito, M. D.; Sobolev, O. V.; Stockwell, D. H.; Terwilliger, T. C.; Urzhumtsev, A. G.; Videau, L. L.; Williams, C. J.; Adams, P. D. Macromolecular structure determination using x-rays, neutrons and electrons: recent developments in phenix. *Acta Crystallogr. D. Struct. Biol.* **2009**, *75*, 861–877.
3. Emsley, P.; Lohkamp, B.; Scott, W. G.; Cowtan, K. Features and Development of Coot *Acta Crystallographica Section D - Biological Crystallography* **2010**, *66*, 486-501.
